# The impact of rate heterogeneity on inference of phylogenetic models of trait evolution

**DOI:** 10.1111/jeb.12979

**Published:** 2016-10-04

**Authors:** A. M. Chira, G. H. Thomas

**Affiliations:** ^1^ Department of Animal and Plant SciencesUniversity of SheffieldSheffieldUK; ^2^ Department of Life SciencesThe Natural History MuseumLondonUK

**Keywords:** absolute adequacy, body mass evolution, evolutionary models, rate‐heterogeneity, relative fit, trait evolution

## Abstract

Rates of trait evolution are known to vary across phylogenies; however, standard evolutionary models assume a homogeneous process of trait change. These simple methods are widely applied in small‐scale phylogenetic studies, whereas models of rate heterogeneity are not, so the prevalence and patterns of potential rate variation in groups up to hundreds of species remain unclear. The extent to which trait evolution is modelled accurately on a given phylogeny is also largely unknown because studies typically lack absolute model fit tests. We investigated these issues by applying both rate‐static and variable‐rates methods on (i) body mass data for 88 avian clades of 10–318 species, and (ii) data simulated under a range of rate‐heterogeneity scenarios. Our results show that rate heterogeneity is present across small‐scaled avian clades, and consequently applying only standard single‐process models prompts inaccurate inferences about the generating evolutionary process. Specifically, these approaches underestimate rate variation, and systematically mislabel temporal trends in trait evolution. Conversely, variable‐rates approaches have superior relative fit (they are the best model) and absolute fit (they describe the data well). We show that rate changes such as single internal branch variations, rate decreases and early bursts are hard to detect, even by variable‐rates models. We also use recently developed absolute adequacy tests to highlight misleading conclusions based on relative fit alone (e.g. a consistent preference for constrained evolution when isolated terminal branch rate increases are present). This work highlights the potential for robust inferences about trait evolution when fitting flexible models in conjunction with tests for absolute model fit.

## Introduction

Phenotypic diversity represents a fundamental axis of biodiversity, alongside variation in species richness. Species diversify into a multitude of forms, and significant differences in the magnitude and disparity of phenotypic traits occur across the tree of life. The speed at which traits change (i.e. the rate of evolution) may vary in numerous ways, including between groups of species (e.g. Hawaiian honeycreepers vs. Hawaiian thrushes, Lovette *et al*., 2002), across habitats (e.g. reef vs. nonreef, Price *et al*., 2011) and between distinct speciation regimes (Rabosky & Adams, 2012; Hipsley *et al*., 2014). Evolutionary rate heterogeneity has been attributed to a multitude of factors that are often taxon and/or trait specific; for example, piscivorus sunfishes experience higher rates of evolution in jaw morphology than nonpiscivorous relatives (*Centrarchidae*, Collar *et al*., 2009), forests promote faster rates of avian song divergence compared with open grassland areas (Weir *et al*., 2012), and among shorebirds, offspring developmental mode is associated with increased rates of evolution for parental care and mating systems (Thomas *et al*., 2006). At broader scales, geographic distributions (e.g. islands vs. mainland, Thomas *et al*., 2009; temperate vs. tropical areas, Martin *et al*., 2010) and geologic events (impacts of the K‐Pg mass extinction, Slater, 2013) have also been shown to influence evolutionary rates.

**Figure 1:**
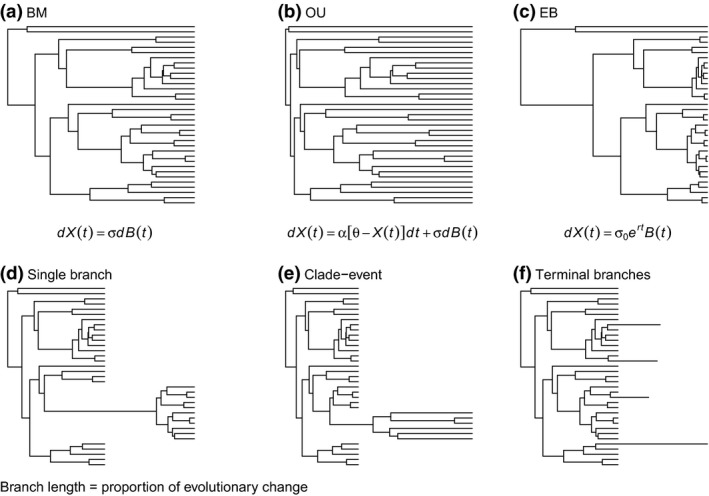
Tree transformations showing how trait evolution is modelled by single‐process approaches: the (a) Brownian motion (BM), (b) Ornstein–Uhlenbeck (OU) and (c) early burst (EB; exemplified by a constant rate‐deceleration process from root to tips) models. The equations describe the process of trait change inferred by models, where *dX*(*t*) represents the change in the trait of interest, *σ* is the rate of change, *dB*(*t*) quantifies random noise by time *t*,* α* represents the ‘rubber band’ parameter acting to pull back the trait values to an optimum phenotype *θ* (OU‐specific), *σ*_0_ is the initial rate of trait change, and *r* is the constant rate‐change parameter (EB‐specific). Hypothetical rate‐heterogeneity scenarios captured by variable‐rates models: rate changes (d) on a single, internal branch, (e) across a whole clade and (f) on isolated tip branches.

Although it is clear that rates of trait evolution vary across phylogenetic, temporal and spatial contexts, the prevalence of different forms of heterogeneity, especially within small clades, is not known. The most commonly used models on clades up to hundreds of species assume that trait evolution can be described by a single process across the whole group of interest. The earliest and most straightforward such approach is the Brownian motion or random walk model (BM) of trait evolution. Under the BM process, evolutionary rates are constant, the mean expected trait change is 0, and variance accumulates linearly in time (Fig. 1a, Cavalli‐Sforza & Edwards, 1967; Felsenstein, 1985). The BM model can describe processes including both genetic drift and adaptation (Hansen & Martins, 1996). Several other approaches build on the BM model, with added parameters aimed to capture the complexities of trait evolution (i.e. deviations from a simple BM process). The Ornstein–Uhlenbeck (OU) model accounts for constrained trait evolution and nonindependence between trait changes at each node in the phylogeny of interest (e.g. when species share similar selection regimes, Butler & King, 2004). Under the simplest version of the OU model (the single stationary peak model), evolutionary rates are constant, but traits are always pulled towards a single optimum value, so that, in time, the phenotype is constrained (Fig. 1b). Other models relax the assumption of a constant rate of evolution, for example by allowing trait change to accelerate or decelerate through time across the whole phylogeny (e.g. ACDC method, Blomberg *et al*., 2003 and *δ*, Pagel, 1999). The most frequently used ACDC approach is the early burst (EB) model, which is a derivation of the BM approach with an extra parameter that models a constant rate‐decrease through time. Under an early burst model, evolution peaks early in the phylogenetic history of the group of interest, after which the mean trait change exponentially decreases (e.g. expected across adaptive radiations, Harmon *et al*., 2010; Fig. 1c).

If evolutionary rate heterogeneity is prevalent, and potentially unpredictable across phylogenies, can we still use single‐process approaches to make inferences about the underlying tempo of evolutionary processes for a specific trait? The interpretation of single‐process models of evolution is apparently appealing and straightforward, but fitting only these models may mask complexity and may not adequately describe variation in the data. The prevailing current approach when studying trait evolution is to fit several models to the data, and then choose the best relative fit based on maximum likelihood or Akaike information criterion (AIC, Burnham & Anderson, 2004). As the absolute adequacy of models is not accounted for, one cannot detect whether all alternative models are deficient. Further, models cannot always differentiate between alternative processes leading to the same trait distribution at the end of the phylogeny (Boettiger *et al*., 2012; Kaliontzopoulou & Adams, 2016). Therefore, the pattern of trait evolution can easily be misidentified. This problem has been recognized (e.g. Freckleton & Harvey, 2006; Pennell *et al*., 2015), and more recently, models have been developed that account for heterogeneity in the tempo of evolution in flexible ways. Several approaches, including Eastman *et al*. (2011) and Venditti *et al*. (2011), use reversible‐jump MCMC to search rate shifts across the phylogeny of interest, assuming a BM mode of evolution between potential transitions (Huelsenbeck *et al*., 2001), whereas others use parametric methods to model distributions of rates (e.g. Elliot & Mooers, 2014). Such methods reveal that rate changes can occur on isolated branches (Fig. 1d), throughout the phylogeny or across whole clades (e.g. Fig. 1e,f; also Baker *et al*., 2016). Changes in the rate of trait evolution can also be modelled as heterogeneity in rate regimes that are temporally variable, as implemented in the Bayesian analysis of macroevolutionary mixture model (BAMM, Rabosky *et al*., 2013; Grundler & Rabosky, 2014; Rabosky, 2014; Rabosky *et al*., 2014a; Shi & Rabosky, 2015).

Although the use of single‐process models has tended to focus on smaller scales (e.g. clade size in Harmon *et al*. (2010) ranges from 6 to 179 species), to date most applications of rate‐variable models have been at relatively large scales on phylogenies including thousands of species (e.g. Venditti *et al*., 2011; Rabosky *et al*., 2013; Baker *et al*., 2015). Consequently, the prevalence of rate heterogeneity and its potential role in misleading single‐process model inferences on trees of the order of hundreds of species is unknown. The aim of this study was to address this knowledge gap by resolving the following issues: (i) how prevalent is rate heterogeneity at relatively small phylogenetic scales, (ii) does the form of rate heterogeneity lead to predictable biases in favour of particular single‐process evolutionary models, and (iii) does accounting for rate heterogeneity improve model fit and provide an adequate description for the data?

To address the first question, we use single‐process and variable‐rates approaches to examine body mass evolution within 88 bird groups, summing up to a total of over 6500 species. Heterogeneity in the rate of evolution for several traits has been previously recorded between avian clades (e.g. Lovette *et al*., 2002) and sister species (Martin *et al*., 2010; Weir & Wheatcroft, 2011). Early bursts of rapid evolution have also been identified in some groups such as ovenbirds (Derryberry *et al*., 2011), vangas (Reddy *et al*., 2012) and Hawaiian honeycreepers (Lovette *et al*., 2002). Avian phylogeny is resolved at the species level (Jetz *et al*., 2012; recent discussions also in Jarvis *et al*., 2014; Prum *et al*., 2015); moreover, body mass data are readily available for most species (Dunning, 2008; Wilman *et al*., 2014), making this system appealing when investigating the prevalence of rate heterogeneity. We further investigate in more detail when and how different forms of rate heterogeneity incapacitate evolutionary models, using simulated rate‐variation scenarios informed by empirical observations. We anticipate that the extent and form of evolutionary rate variability will mislead the patterns of trait evolution quantified by single‐process methods and model choice, leading to spurious inferences of macroevolutionary processes. Conversely, variable‐rates approaches should perform better both in relative fit and in absolute adequacy.

## Materials and methods

### Models of trait evolution

We used the BM (Cavalli‐Sforza & Edwards, 1967), OU (Butler & King, 2004) and EB (Harmon *et al*., 2010) models as representatives of popular single‐process approaches. The models were fitted using fitContinuous() in the R package GEIGER (Pennell *et al*., 2014), using 100 iterations. For some clades (the accentors, olive warbler and woodpeckers in the empirical analyses), the likelihood surface for the OU alpha parameter consisted of a flat ridge (similar to Harmon *et al*., 2010) and could not be estimated reliably; therefore, we excluded the OU analyses on these clades. The relative fit of models was determined using the AICw selection criteria (Burnham & Anderson, 2004). We are aware that AIC can be biased towards models with increasing number of parameters and provide a flawed relative hierarchy between nested methods (e.g. Kaliontzopoulou & Adams, 2016); however, our objective was to replicate and assess the common approach when studying trait evolution, and for the BM, OU and EB models, the number of parameters differs by a maximum of 1.

The Variable‐Rates Model for Continuous Traits in BayesTraits V2 (further referred to as BayesTraits for simplicity; http://www.evolution.rdg.ac.uk/BayesTraits.html) was used as a first representative of variable‐rates models. BayesTraits implements changes in the rate of evolution using two scaling mechanisms that can be added at any location in the tree: a single‐branch modification (modifies the rate on a target branch) and a clade modification (adjusts a target branch and all its descendants; Venditti *et al*., 2011). The model outputs posterior configurations of rate shifts that best predict the tip trait data on the phylogeny of interest. Uniform (default) priors with no restrictions were used for alpha (phylogenetic mean) and sigma (Brownian variance) parameters. Four chains were run to ensure convergence between independent runs. Within‐ and between‐chains convergence was assessed using trace and auto‐correlation plots, effective sample size and the Gelman–Rubin diagnostic, all tested in the R package CODA (details in the supporting information; Plummer *et al*., 2006). We further used BAMM version 2.3.0 (http://bamm-project.org/) as a second example of methods allowing for variation in the rate of trait evolution. Under BAMM, the process of rate change is dependent on time, following:

$$\sigma \left(t\right)=\sigma_{0}\;\text{exp}\;\left(zt\right),$$

where *σ*(*t*) represents the rate of gradual trait change in time *t*,* t* is the elapsed time from the start of the regime, *σ*_0_ is the initial regime rate, and *z* is a rate parameter that controls for the magnitude of trait change in time. BAMM thus models multiple time‐dependent, gradual rate changes, giving an approximation of continuous rate‐variation processes with occasional jumps. For each tree and associated tip data, the priors for the Poisson rate (in BAMM 2.5.0, this is equivalent with the inverse of the expected number of shifts), initial evolutionary rate and rate‐change parameter in each regime were calculated in R, using the function setBAMMpriors (Rabosky *et al*., 2014b). Throughout, the function set the poissonRatePrior = 1, whereas values for the betaInitPrior and betaShiftPrior varied between trees. The model also put a uniform prior density on the distribution of ancestral states, with bounds depending on the range of the observed data (useObservedMinMaxAsTraitPriors = 1). BAMM offers the possibility to switch between time‐constant and time‐varying processes of trait evolution when modelling rate variation via the time‐flip proposal. We performed BAMM analyses: (i) with the time‐flip proposal to allow both time‐varying and time‐constant processes (betaIsTimeVariablePrior = 0.5 and updateRateBetaTimeMode = 1), and (ii) limiting the model to time‐varying rate‐heterogeneity processes (betaIsTimeVariablePrior = 1 and updateRateBetaTimeMode = 0, the default in BAMM 2.3.0). Four chains were run and convergence between and within chains was assessed in CODA (details in the supporting information).

### Empirical data

We used maximum clade credibility trees for 88 avian clades from the Jetz *et al*. (2012) stage 1 distribution (trees include genetic data only; accessed via Birdtree.org). Tree size ranged from 10 to 318 species, covering a total of 6656 extant bird species. Bird body mass data was taken from EltonTraits 1.0 (Wilman *et al*., 2014). EltonTraits comprises specific body estimates based on (i) the geometric mean of average values for both sexes from Dunning (2008), and (ii) genus average from other sources. Body mass estimates (in grams) for each species were log‐transformed. We calculated the median scaled trees from the outputs of BayesTraits and BAMM, in which each branch length is stretched and shortened proportional to the median rate of evolution across the posterior scaled tree distribution for that particular branch. Posterior scaled trees are readily available in the output of BayesTraits. For BAMM, we modified the function getMeanBranchLengthTree() in R (package BAMMtools, Rabosky *et al*., 2014b), so that it computed the per‐branch median rates across the posterior tree distribution (instead of the mean; code deposited at doi: 10.5061/dryad.qj367). Median scaled trees were used to visualize and describe patterns of trait evolution, and further as input for absolute model fit analyses (across both the empirical and simulated data). For the avian data sets, we also compared the fit of alternative models with various number of supported shifts given by BAMM‐flip using BayesFactors (calculated with computeBayesFactors() in BAMMtools, Rabosky *et al*., 2014b).

### Simulations

We simulated trees with 100 tips under a pure birth model using TreeSim (Stadler, 2011), with a speciation rate set to 1. We chose this specific tree size because standard trait evolutionary models are typically applied on relatively small phylogenies with 50–200 tips. The root‐to‐tip distance was standardized to 1 in all trees. Rate‐heterogeneity scenarios were simulated by changing the length for specific branches of interest (discrete shifts), or by generating gradual processes using the function rescale() in GEIGER. Brownian motion trait evolution with a variance rate of 1 was further simulated on these transformed trees. The original tree and the simulated trait data were used as input data for alternative models of trait evolution. We simulated rate variation as (i) a single, internal branch shift not passed to descendants (Fig. 1d), (ii) a clade event, in which all members of a particular group record a change in the rate of evolution (Fig. 1e), (iii) rate shifts on nonclustered terminal branches (Fig. 1f), (iv) a constant rate‐deceleration process from the root to tips (Fig. 1c) and (v) a case when a single clade goes through an initial increase in the rate of evolution (×5) followed by a constant‐rate decay (same process as Fig. 1c, but constrained to a clade). The number of terminal branches and the size of clades that recorded rate shifts were set to 15–30 species. Combinations of the first three scenarios were also added. All code used for the simulations is deposited at doi: 10.5061/dryad.qj367. Parameter choices for the simulations were informed by the rate‐heterogeneity patterns observed on the empirical data and also by inference to the literature (discrete branch shifts: Venditti *et al*., 2011; Revell *et al*., 2012; Thomas & Freckleton, 2012; Puttick *et al*., 2014; Baker *et al*., 2016; gradual rate decreases: Harmon *et al*., 2010; Rabosky *et al*., 2014a; Slater & Pennell, 2014). Discrete shifts were given magnitudes of ×0.05, ×0.1, ×0.2, ×0.5, ×2, ×5, ×10 and ×20. Gradual rate decreases were set under a rate‐deceleration parameter (*a*) of ln(0.5), ln(0.2), ln(0.1) and ln(0.05). Each heterogeneity scenario with its respective magnitude was simulated on 100 trees, resulting in a total of 6400 trees and trait data. We used an additional 1000 constant‐rate trees, that is trees with a simulated BM process of trait evolution, and associated tip data, to assess model fit in the absence of rate heterogeneity. We also investigated whether the size of trees influences the ability of variable‐rates models (BayesTraits and BAMM‐flip) to detect heterogeneity. To do this, we simulated additional 400 trees with 25, 50, 100 and 200 tips (100 trees for each size), and we repeated the discrete rate‐variation scenarios. The size of clades and number of terminal branches that recorded rate changes were set to 10–15, in order to accommodate for trees of only 25 tips.

The probability of internal branch shifts, clade events and terminal branch shifts to be detected by models was also quantified using the simulated data. We fitted the BM model: (i) on the simulated trees, that is trees with incorporated rate heterogeneity, alongside the simulated trait data, and (ii) on trees before applying rate changes, alongside the simulated trait data. The differences in log‐likelihood between (ii) and (i) were calculated; small differences in log‐likelihood indicate that a particular heterogeneity scenario does not leave much signal in the tip data.

### Absolute model fit

Freckleton & Harvey (2006) proposed bootstrapping approaches to assess the adequacy of the Brownian model as a descriptor of the data. More recently, Pennell *et al*. (2015) extended this approach with a series of parametric tests of the absolute adequacy of trait evolutionary models implemented in the R package ARBUTUS (Pennell *et al*., 2015). Briefly, the algorithm works as follows: (i) an evolutionary model is fitted to the data, (ii) a unit tree is built by transforming the original tree according to the model parameters, (iii) Felsenstein's independent contrasts (Felsenstein, 1985) are calculated on this unit tree, making up the ‘observed data’, (iv) trait evolution is simulated on the unit tree, following a BM process with variance = 1, and the contrasts are calculated again (i.e. the ‘simulated data’), and (iv) the observed and simulated distribution of contrasts are compared. ARBUTUS takes a phylogeny and the associated tip trait distribution as input; therefore, for the variable‐rates models, a BM model was run on the median scaled tree at step (i), and the unit tree was built according to the BM parameters on the scaled tree.

ARBUTUS provides six diagnostics that test model fit: (i) the coefficient of variation of the absolute value of contrasts (C.VAR) tests whether the candidate model underestimates (C.VAR_obs_ > C.VAR_sim_) or overestimates (C.VAR_obs_ < C.VAR_sim_) total rate heterogeneity, (ii) the mean of the squared contrasts (M.SIG) assesses model ability to quantify the overall rate of evolution, (iii) the D statistic (Kolmolgorov–Smirnov test) compares the distribution of the contrasts with the expected $X∼N\left(0,\sigma^{2}\right)$; D.CDF tests for deviations from the expected normal distribution of contrasts. The last three diagnostics represent the slopes of several linear models fitted to the absolute value of contrasts (iv) against node heights (S.HGT), which assesses model ability to account for temporal variation (positive slopes show rate overestimations late in the phylogeny and underestimations early on), (v) against the variances of contrasts (S.VAR), signalling if models account for variation related to branch lengths (positive slopes show rate underestimation on long branches and overestimation on short ones), and (vi) against the weighted average values at each node (S.ASR), which tests whether the model accounts for variation related to ancestral states (positive slopes show overestimates at smaller nodes and underestimates at larger nodes). A candidate model is considered inadequate for a particular test when the observed and simulated test statistics are significantly different (*P *<* *0.05). We used the *P*‐values to calculate the frequency of inadequate trees and associated trait data (referred as inadequacy levels) given by each candidate model across our simulated scenarios. The ability of variable‐rates models to detect rate shifts on simulated trees of different sizes was assessed by calculating (i) the posterior probability for the simulated branch and clade rate shifts (for BayesTraits), and (ii) the relative odds of a clade shift (i.e. marginal odds ratio) for BAMM‐flip; currently, a protocol for assessing the probability of individual branch shifts is not formally described for this model.

We used the simulated trees and data under various heterogeneity scenarios to compare the rate estimates from variable‐rates models with the true, simulated ones. Specifically, for each branch where a rate change was simulated, we calculated the natural logarithm for the proportion between the estimated and true rate of evolution. Positive values indicated that models overestimated the evolutionary rate on branches. These differences were calculated for the branches without simulated rate changes as well.

We also used constant‐rate trees and associated trait data to evaluate potential tendencies of variable‐rates models to infer false rate heterogeneity. BayesTraits has revealed a wealth of rate changes in body mass evolution across the mammalian tree (Venditti *et al*., 2011); therefore, we first calculated the prevalence of branch rate changes inferred in constant‐rate trees by BayesTraits that could potentially be interpreted as shifts in the rate of evolution. Secondly, BAMM has been used to identify time‐varying evolution within clades (Grundler & Rabosky, 2014; Rabosky *et al*., 2014a). We thus tested whether the default BAMM model (where all rate regimes are modelled as time‐varying) infers false gradual rate‐change processes, particularly early in the phylogeny. We further tested whether any such biases are alleviated by using BAMM's time‐flip proposal that allows both time‐varying and time‐constant rates to be modelled. We used the function getEventData() in BAMMtools to extract the rate‐change parameter (*β*) for the root process. These *β* parameters should distribute normally around 0 if no rate‐change regime characterizes the root. We also plotted the *β* distribution for the simulations involving rate‐discrete shifts, to test for a potential link between specific rate‐heterogeneity scenarios and falsely inferred gradual processes at the root.

## Results

### Avian groups

**Figure 2:**
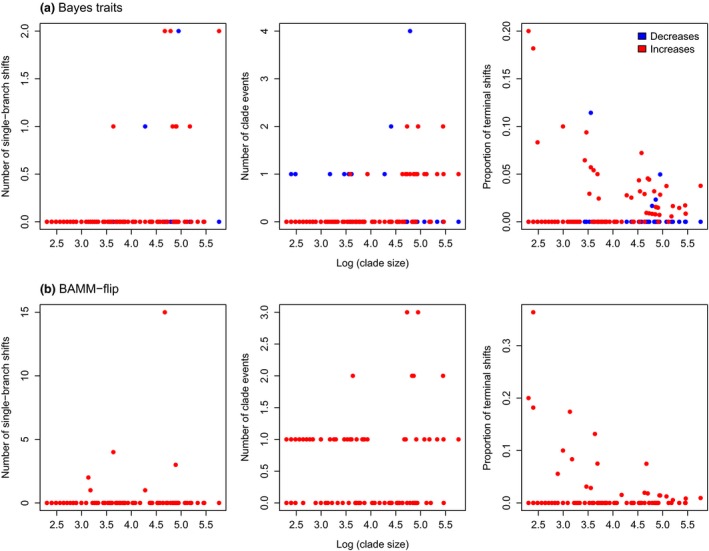
Patterns of rate heterogeneity in avian body mass evolution given by (a) BayesTraits and (b) BAMM‐flip, plotted against clade size. Rate variation is measured as: number of single‐branch rate changes, number of clade events and proportion of isolated changes at the tips. Rate decreases and increases are represented in blue and red, respectively.

Heterogeneity in the rate of body mass evolution was prevalent across bird phylogenies (Fig. 2, considering per‐branch rate changes more substantial than ×2 or ×0.5 as evidence for rate variation), and the intensity and patterns of rate changes varied across clades. Several recurrent forms of rate heterogeneity stood out (Fig. 2): rate changes affecting whole clades (e.g. *Paradoxornis* genus, Fig. S71; *Geospiza* and *Camarhynchus* genera, Fig. S78; *Cinclodes* genus, Fig. S84), rate increases on isolated terminal branches (e.g. Figs S76, S81, S83) and evolutionary rate increases on an internal branch not passed to descendants (referred to as ‘single‐lineage ancestral bursts’ in Venditti *et al*., 2011 and Baker *et al*., 2016; e.g. Fig. S99a). There was also evidence of time‐dependent declining rates of evolution within groups, and BAMM revealed fast rates early in the phylogeny followed by declining rates in few cases (e.g. *Pachycephalidae* Fig. S36b; *Procellariidae,* Fig. S99b). Further, BAMM detected 35 groups that had strong evidence for at least one regime shift (Bayes factors for one or more shifts relative to the null model > 20), and in 43 groups, there was at least some effect for one or more rate regime changes (Bayes factors > 12; Table S5). Highest extents of rate variation were typically inferred in the large clades, but there was no clear relationship between the prevalence of heterogeneity and clade size (Fig. 2). Rate shifts were found across small groups (e.g. pheasants, quail, guineafowl, 11 species, Fig. S91; orioles, allies, 32 species, Fig. S67), and also some larger clades had little to no rate variation throughout (e.g. cuckoos, 128 species, Fig. S53; buntings, American sparrows, brush–finches, 127 species, Fig. S57). Typically, rate shifts did not exceed a 30‐fold increase or a five‐fold decrease, but there were a limited number of exceptions (e.g. the *Platysteiridae* family undergoes a 14‐fold decrease in the amount of body mass change relative to the length of the identical branch in the input phylogeny, Fig. S100).

**Figure 3:**
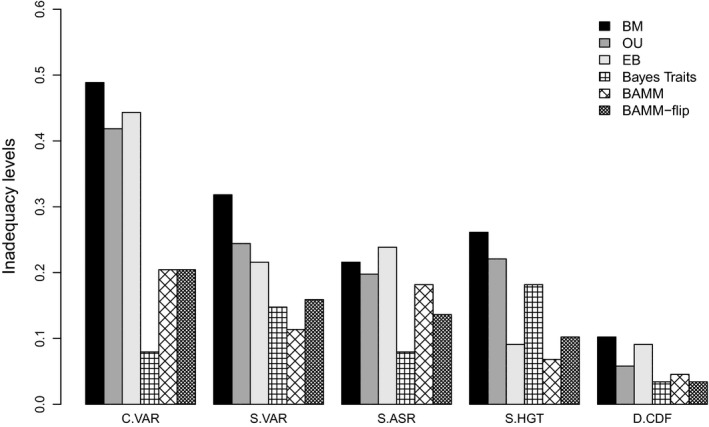
Inadequacy levels (quantified as the frequency of trees and associated trait data where the focal model was inadequate) for evolutionary models across avian clades, showing model inability to account for total variation in the rate of evolution (C.VAR), variation related to branch lengths (S.VAR), ancestral states (S.ASR) and node heights (S.HGT). D.CDF inadequacy refers to deviations in the distribution of independent contrasts from the expected normality under a BM. Single‐process (BM, OU and EB) and variable‐rates models (BayesTraits and BAMM with time‐flip proposal) are considered.

Variable‐rates models generally represented an adequate approach to model body mass evolution across avian clades (Fig. 3). Conversely, single‐process models underestimated the total amount of rate variation in almost 50% of the groups included in the analyses. Further, the inadequacy of single‐process approaches was predominant across phylogenies that showed high rate heterogeneity (as described by rate‐variable models, Fig. S15). Most important, variable‐rates models were not just better at capturing the evolutionary process relative to single‐process approaches (expected, as absolute fit does not penalize complexity), but they also recorded high levels of absolute adequacy. Therefore, such methods provide robust descriptions of the statistical patterns in the data, whereas single‐process models frequently do not. BAMM and the EB model described the temporal aspect of evolution best (best adequacy in the S.HGT diagnostic), as the rest of the models tended to underestimate the rate of evolution early in the phylogeny, and/or overestimate it towards the tips (positive S.HGT, Table S3). The BAMM version constrained to time‐varying processes typically produced stronger rate‐deceleration processes at the root compared with the BAMM‐flip alternative (Fig. S14), mostly in small clades (< 50 tips).

The BM model had highest AICw in 54% of trees (Figs S17–S63), followed by the OU (24%; Figs S64–S86) and EB models (22%; Figs S87–S104). The relative and absolute adequacies of single‐process models were not tightly related. Rather, the prevalence of highest AICw for the OU model increased as models missed more and more sources of variation (Fig. 4). Thus often a superior relative fit of the OU model was not a result of best absolute fit, but of alternative evolutionary processes that were not accounted for by any of the single‐process models included. We found 11 clades in which the OU model had over 90% support from the AICw over the BM and EB, but all three models had poor absolute adequacy (select *Pellorneidae* and *Sylviidae*, Fig. 71c; *Alaudidae*, Fig. S72c; select *Anatidae*, Fig. S74c; *Pycnonotidae*, Fig. S76c; *Lari*, Fig. S77c; select *Thraupidae*, Fig. S78c and Fig. S81c; *Psittacidae*, Fig. S79c; *Fringillidae*, Fig. S80c; *Muscicapidae*, Fig. S83c; *Furnariidae*, Fig. S84c); within these groups, variable‐rates models typically identified rate increases late in the phylogeny, in the form of clade events and/or increases on isolated terminal branches. Absolute adequacy levels also helped distinguishing between the relative fit of models with similar AICw. We found 12 clades in which the BM and EB models were not clearly separated by their AICw, but were assigned different adequacy levels by ARBUTUS (*Trogonidae*, Fig. S35c; select *Acanthizinae*, Fig. S42c; *Conopophagidae*, Fig. S87c; *Melanocharitidae* and *Cnemophilidae*, Fig. S88c; *Maluridae*, Fig. S90c; *Petroicidae*, Fig. S92c; *Cardinalidae*, Fig. S94c; *Vireonidae*, Fig. S95; *Procellaridae*, Fig. S99c; select *Psittacidae*, Fig. S101c; *Numididae*, Fig. S102c; *Meliphagidae*, Fig. S103c). Within these groups, the BM (and BayesTraits) failed to account for temporal variation, and underestimated rates late in the phylogeny; conversely, the EB (and BAMM) was adequate across all diagnostics.

**Figure 4:**
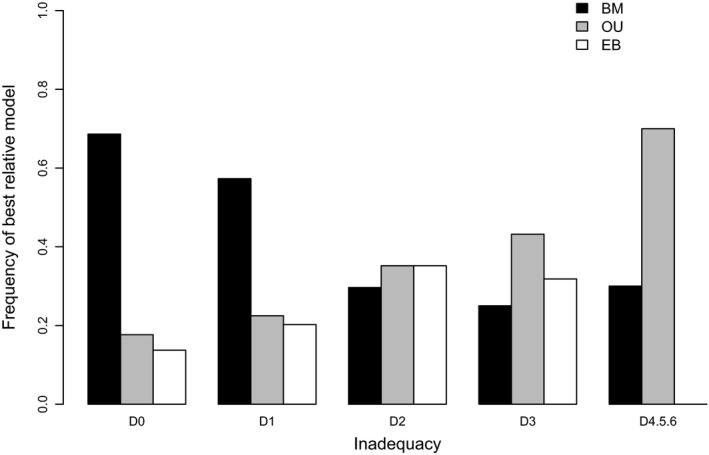
Frequency of the best relative single‐process model (highest AICw) for increasing levels of inadequacy across avian clades. Inadequacy levels are quantified as the number of model fit diagnostics failed across all three models (from D0 – no adequacy tests failed, to D4.5.6 – four or more failed tests).

### Model fit in the presence of simulated rate heterogeneity

**Figure 5:**
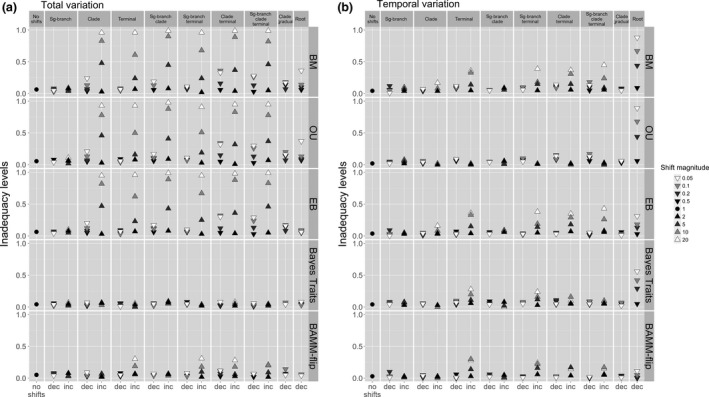
Model inadequacy levels (quantified as the frequency of trees and associated trait data where the focal model was inadequate) across a simulated Brownian motion process (no shifts, i.e. shift magnitude = 1) and rate‐heterogeneity scenarios: internal branch shift; clade event; rate changes on isolated, terminal branches; rate burst followed by gradual decreases within a clade, and constant rate‐deceleration process from root to tips. Single‐process (BM, OU and EB) and variable‐rates models (BayesTraits and BAMM with time‐flip proposal) are considered. Inadequacy levels measure model ability to account for (a) total rate variation and (b) temporal variation. Inadequacy is quantified separately for rate increases (inc, up‐pointing triangles) and decreases (dec, down‐pointing triangles), and the exact magnitude of each shift is highlighted by the white–black colour scheme. For scenarios involving gradual rate changes, the natural logarithm of the shift magnitude represents the constant rate‐change parameter.

**Figure 6:**
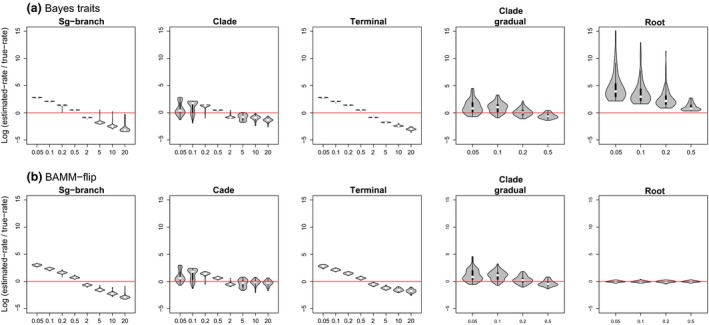
Distributions of log‐proportions between rates estimated by variable‐rates models and the true (simulated) rate changes on the identical branches. Distributions are shown for various shift magnitudes (*x*‐axis) and heterogeneity scenarios: internal branch shift; clade event; rate changes on isolated, terminal branches; rate burst followed by gradual decreases within a clade, and constant rate‐deceleration process from root to tips. Results for BayesTraits (a) and BAMM‐flip (b).

In the absence of rate heterogeneity (constant‐rate trees), all models perform adequately. However, the single‐process models vary in their ability to capture evolution on heterogeneous trees (Fig. 5). Similar to results on the empirical data, variable‐rates models generally performed better than single‐process models, and also recorded low levels of inadequacy overall. The magnitude of rate changes affected the absolute fit of models consistently across all simulated rate‐heterogeneity scenarios. Specifically, the fit of single‐process models was better on simulations involving decreases in the rate of evolution compared with rate increases. On branches with simulated rate changes, variable‐rates models typically underestimated the magnitude of rate changes (Fig. 6; also Fig. S3b). This effect was stronger with increasing magnitudes of rate shifts, and ARBUTUS diagnostics also detected a poorer model fit as the magnitude of rate shifts became bigger for both rate increases and rate decreases (Fig. 5). The mean of the squared contrasts (M.SIG) was very rarely inadequate across our analyses, and this particular diagnostic has been previously identified as having low power to detect model inadequacy (Pennell *et al*., 2015). We therefore do not report or discuss M.SIG further. Also, we did not specifically model directional trends of rate variation in relation to ancestral states or branch lengths. Accordingly, these ARBUTUS diagnostics do not reveal any specific problems related to the models fitted; rather, inadequacy levels follow the trends predicted by the tests related to temporal and total rate variation (Fig. S1).

#### Model ability to account for overall rate heterogeneity

Single‐process models recorded particularly high levels of inadequacy when heterogeneity is simulated as rate increases on isolated terminal branches or on several branches forming a clade (Fig. 5a). In addition, and as expected, the BM and OU models frequently fail to account for rate‐deceleration processes across the whole tree. Although designed to model rate heterogeneity, BAMM also tended to underestimate total rate variation (mostly positive C.VAR differences, Fig. S5a), and especially missed the rate increases on isolated terminal branches. However, the inadequacy levels for BAMM were typically lower than the single‐process models. Further, the time‐flip proposal improved absolute adequacy relative to the fixed time‐varying prior in BAMM (Fig. S4a). Overall, model adequacy in terms of capturing rate heterogeneity was highest for BayesTraits; however, it was also the only model that regularly overinflated estimates of the total rate variation (negative C.VAR differences, Fig. S5a; also slightly higher differences between true and estimated rates of evolution compared with BAMM; Fig. 6).

#### Model ability to account for temporal rate variation

Not surprisingly, the BM and EB models described the temporal aspect of rate variation poorly when rate increases were simulated on terminal isolated branches (Fig. 5b), as they underestimated these late shifts (negative S.HGT, Fig. S5b). BAMM also showed a ubiquitous tendency to overestimate rates early on and underestimate the late increases (all negative S.HGT; Fig. S5). All models except BAMM were unable to accurately account for rate‐deceleration processes across the whole phylogeny (Fig. 5b), as they underestimate high initial rates and overestimate terminal rates (all positive S.HGT, Table S1). The EB model performed better than the BM and OU models (as expected), and BayesTraits, but still tended to miss fast decelerating processes. Early bursts also led to the highest inadequacy levels for BayesTraits compared with all other heterogeneity scenarios (Figs 5 and 6).

#### The influence of tree size on model ability to detect rate shifts; tendency of variable‐rates models to overfit; likelihood tests

The ability of BayesTraits to detect a rate shift on individual branches or across a whole clade was not influenced by the size of the simulated trees (Figs S6–S8). The ability of BAMM‐flip to detect a clade rate shift did not differ between trees of different sizes although on average the model recovered rate increases better in bigger trees (Fig. S9). Further, the ability of BayesTraits to detect a clade shift in trees of 100 tips was little influenced by the size of the heterogeneous clade (Fig. S10). Similarly, BAMM‐flip recovered clade rate changes similarly well across different clade sizes (Fig. S11). Universally, the main factor affecting model ability to detect rate shifts was the shift magnitude, and models recovered big shifts better than smaller ones, in respect of both increases and decreases in the rate of evolution.

BayesTraits commonly inferred rate increases up to two‐fold when fitted on constant‐rate trees and associated data (26–33% frequency of trees with rate shifts); however, the frequencies of trees with shifts dropped considerably when considering rate changes bigger than ×5 (8.5%), ×10 (0.5%) and ×20 (0%, Table S2). Further, the vast majority of rate increases occurred on terminal branches. There was no clear tendency for BAMM to infer false early rate‐decelerating processes when fitted on constant‐rate trees and trait data, using either a time‐flip proposal (*β* distributions average around a mean = −0.14 ± 0.18 SD, and a median = −0.08) or not (*β* mean = −0.12 ± 0.43 SD, and a median = −0.07, Fig. S12). Per‐branch comparisons between the estimated and true rates of evolution across constant‐rate trees also show no worrying amount of overfit from variable‐rates models; however, rates inferred by BAMM‐flip show more noise around the true values compared with BayesTraits (Fig. S2).

When considering the data simulated with single branch, clade and terminal shifts, *β* values also distributed normally, but the central points and deviations differed across heterogeneity scenarios (Fig. S13). When evolution was constrained to time‐varying processes (Fig. S13b), *β* distributions were slightly shifted right towards positive values for simulated rate increases; that is, BAMM infers processes of slight gradual rate increases at the root when some late rate increases are present. This trend was, however, corrected by BAMM‐flip (Fig. S13a). Using the time‐varying constrained BAMM alternative also resulted in many weak deceleration processes at the root, rectified by BAMM‐flip (*β* much narrowly distributed along the 0 line). Both BAMM versions approximated slightly steeper rate‐deceleration processes as a response to discrete rate decreases late in the clade (wider ranged *β* distributions). Per‐branch differences between estimated and simulated rates of evolution also showed a small tendency for BAMM‐flip to overestimate rates of evolution on nonchanged branches as a response to big rate increases at the tips (Fig. S2b). Conversely, BayesTraits underestimated rates on nonchanged branches in these trees (Fig. S2a).

**Figure 7:**
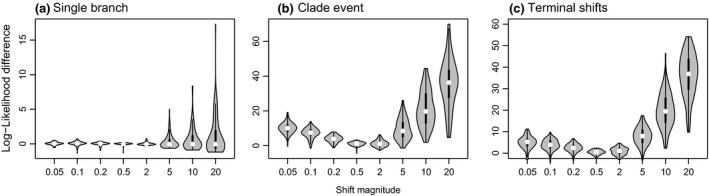
Log‐likelihood differences between runs on original and transformed trees (with incorporated rate changes) for three rate‐heterogeneity scenarios: (a) internal branch shift, (b) clade event, and (c) multiple nonclustered rate changes at the tips. The magnitudes of shifts in each category are represented on the *x*‐axis.

As expected, single‐branch shifts do not leave much signal in the tip data, whereas clade events and shifts on multiple isolated terminal branches have a high likelihood of being detected by models. Similarly, rate decreases are much less detectable compared with rate increases, and, as the magnitude of a shift increases, so does its signal in the tip data (Fig. 7).

#### Absolute vs. relative model fit selection criteria in the presence of rate heterogeneity

**Figure 8:**
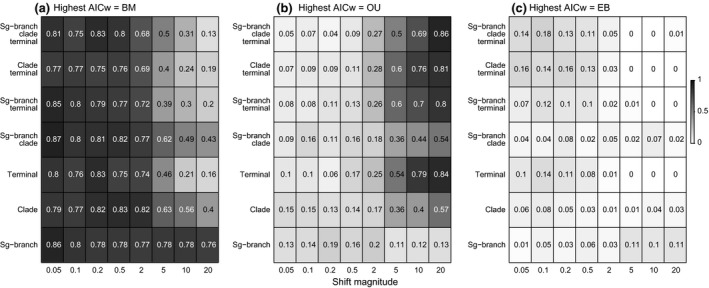
Frequency of best relative model (highest AICw) for the BM, OU and EB models across simulated heterogeneity scenarios: internal branch shift, clade events, isolated terminal changes and combinations. Columns correspond to specific shift magnitudes for each scenario. The colour scheme highlights low (white) to high (black) frequencies of best relative AICw.

**Figure 9:**
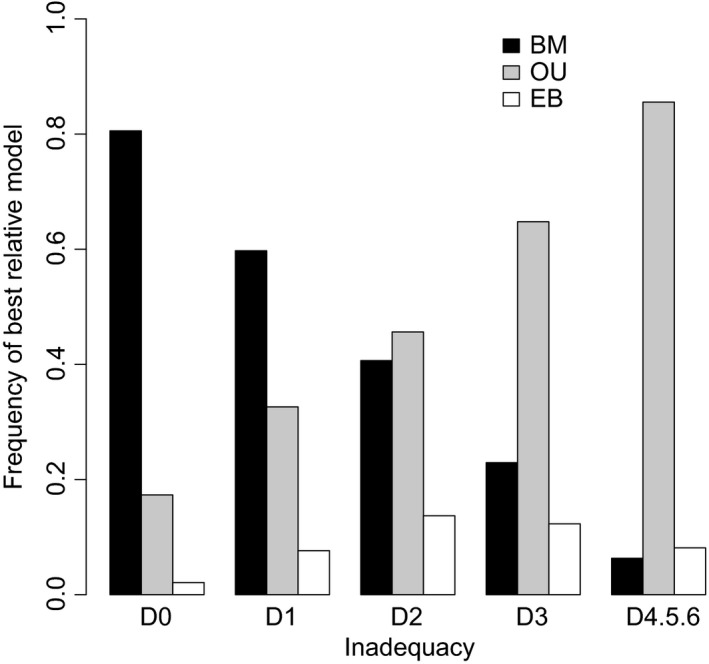
Frequency of the best relative single‐process model (highest AICw) for increasing levels of inadequacy across all simulated discrete rate‐heterogeneity scenarios. Inadequacy levels are quantified as the number of model fit diagnostics failed across all three models (from D0 – no adequacy tests failed, to D4.5.6 – four or more failed tests).

Across scenarios simulated under a BM process with discrete shifts (internal branch shift, clade event and terminal rate shifts), the BM model was expectedly most often favoured by model selection criteria, followed by the OU and EB processes (Fig. 8). Similar to the empirical data, the relative preference for the OU model was not spread randomly across the heterogeneity scenarios considered; rather, the OU model was particularly favoured in scenarios involving big rate increases on branches late in the phylogeny (Fig. 8b). Further, relative model selection criteria did not reflect the absolute fit of models, and the cases in which the OU model was picked up as best across these simulations were clearly linked with a high inadequacy of all three single‐process models fitted (Fig. 9).

## Discussion

### Patterns of rate heterogeneity in avian body mass evolution and consequences to model fit

Generally, variable‐rates models performed well in capturing the phylogenetic distribution of the data, as highlighted by their low levels of inadequacy across ARBUTUS diagnostics, on both simulated and empirical data sets. Allowing for rate heterogeneity when modelling trait evolution can thus provide a robust approach to understanding trait evolution, both in the presence and absence of variability in rates. Conversely, assuming a constant process can misguide the choice of best model and generate poor inferences about the evolutionary process across groups of interest. The intensity of body mass rate variation fluctuated across avian phylogenetic groups, but rate heterogeneity was prevalent. As a consequence, single‐process models commonly gave poor estimates on the total amount of rate variation present in these data sets and were highly inadequate compared with the more flexible variable‐rates approaches. In general, evolutionary models recorded similar inadequacy tendencies across simulated and empirical data sets, ruling out biases such as phylogenetic or measurement error as determinants of inadequacy differences between models in favour of rate heterogeneity. Observations on model inadequacy specific to the empirical data sets likely signalled attributes of avian body mass evolution.

Several clades (e.g. albatrosses, shearwaters, petrels, Fig. S99b; Whistlers, Fig. S36b) showed a characteristic of high rates early in the phylogeny followed by rate‐decelerating processes, identified by BAMM and the EB model. The simulation step highlighted the tendency of BM, OU and BayesTraits to miss such patterns. Therefore, where inferred, early bursts are likely an accurate description of body mass evolution. Accordingly, the distribution of the BAMM rate‐decay parameters at the root (*β*) across the empirical data was fat‐tailed, with the outliers signalling the burst processes (Fig. S14). BAMM without the time‐flip algorithm recorded more powerful decelerating processes at the root (i.e. smaller *β* values), alerting on a potential bias for this strict time‐varying alternative to infer false extreme early rate‐decay processes (especially in clades with < 50 species). Additionally, variable‐rates models identified rate heterogeneity in the form of branch rate decreases or increases that are not passed to descendants making up a monophyletic group, recurrent whole‐clade events and changes on nonclustered, terminal branches. Both BayesTraits and BAMM reveal a similar prevalence of rate variation in avian clades (Fig. 2). We are aware that quantifying the extent of this variation based on per‐branch shifts is not particularly suitable for BAMM, as it can miss or misinterpret gradual processes. However, the algorithm was generally robust, and there was only one extreme case in our analyses: the fast rate‐deceleration process in albatrosses, shearwaters, petrels was quantified as a BAMM‐flip output of 15 single‐branch bursts (Fig. 2b). Some of the avian clades identified in our analyses with a high degree of rate heterogeneity in body mass evolution have also been associated with high diversification rates and rapid radiations (e.g. ovenbirds, select gulls, hummingbirds, ant birds and tyrants; Jetz *et al*., 2012).

The forms of rate heterogeneity we report are most likely not a statistical artefact, given the high prevalence of consistent rate‐variation patterns and the general low inadequacy levels of variable‐rates models. Moreover, similar patterns have also been reported across a variety of phylogenetic groups: clade rate increases (Pacific minnows, Martin & Bonett, 2015) and decreases (*Taphozous* bats, Venditti *et al*., 2011), similar group events, but involving a basal shift, propagated then throughout the clade of interest (*Ctenotus* lizards, Rabosky *et al*., 2014a), single‐lineage internal bursts restricted to the branches leading to *Hominidae* (great apes), *Chiroptera* (bats, Venditti *et al*., 2011) or *Mysticeti* (baleen whales, Baker *et al*., 2016). Such phylogenetic distributions of rates reinforce the importance of allowing for lineage‐specific rate changes when modelling trait evolution, in order to avoid inaccurate inferences about the evolutionary process. As presented, even for phylogenetic scales up to hundreds of species one could attribute differences in the rate of evolution between groups to a general clade event rather than to considerable changes on a single or restricted number of lineages.

We used the output of variable‐rates models in conjunction with adequacy checks to clarify the conclusions on the tempo of trait evolution in some problematic avian groups. For example, across tanagers and allies, the OU model had a clear superior relative fit. However, all single‐process models were inadequate, and variable‐rates models further showed an exceptional burst of evolution within the clade consisting of Galapagos finches (Fig. S78). Thus, based on relative fit only, an interpretation of constrained evolution could have been preferred to a completely different, limited island radiation hypothesis. We identified the same issue even when the number of radiating species was very small (like the case of steamer ducks, a genus of only four flightless ducks, Fig. S74). Absolute adequacy checks also guided output interpretation for variable‐rates models. For example, in the clade *Procelariidae*, BayesTraits inferred a single‐branch shift increase ancestral to albatrosses, evolving towards a big body size (Fig. S99a). BAMM, however, inferred this ancestral increase as part of an early burst process spanning across the whole phylogeny (Fig. S99b). ARBUTUS signalled that BayesTraits inadequately described the temporal variation in this group and missed early fast rates, thus favouring the BAMM interpretation of rate variation on this tree. The EB model also modelled temporal heterogeneity accurately but missed the complexities of rate variation across the whole clade (positive C.VAR, Fig. S99c).

We only used trees containing species where sequence data was available, ruling out a potential over inflation of rate heterogeneity (especially towards the tips) or biased model preference towards an OU model due to incorporating species based on taxonomic information only (Rabosky, 2015). We did not, however, incorporate measurement error into our empirical analyses, which could potentially cause an overestimation of rate heterogeneity across the body mass data (Silvestro *et al*., 2015). From the two variable‐rates models included in our analyses, BayesTraits can account for measurement error by modelling many rate increases on isolated terminal branches, but it cannot be distinguished whether the presence of such increases in the outputted scaled trees is caused by noisy data or real rate changes at the tips. However, our analyses on simulated data sets showed that the model rarely gives false substantial rate changes at the tips. Still, we argue that some rate variation across empirical data sets should be interpreted with caution, if at all, and the above‐mentioned considerations led us to not take into account rate shifts smaller than ×2 when quantifying patterns of avian trait evolution (Fig. 2).

### Heterogeneity patterns that mislead models

As a general rule, specific forms of heterogeneity and not the general complexity of rate variation caused problems for evolutionary models. That is, when a specific rate‐heterogeneity pattern caused a model to fit poorly, the effect occurred frequently across all simulations. For example, data simulated with a shift in rate across a whole clade led to poor performance of candidate models, regardless of whether other types of shifts were also simulated. Having simulated under a range of scenarios and magnitudes enabled us to mark how models approximate trait evolution in response to various heterogeneity cases, and also highlight which and to what extent rate‐variation scenarios mislead model inference.

There was a clear difference between how models handled increases and decreases in the rate of evolution. Single‐process models came out as more adequate in the presence of rate decreases compared with increases. This difference in model fit is probably a consequence of the small likelihood that discrete branch rate decreases leave any signal in the data (Fig. 7). That is, single‐process approaches do not approximate rate decreases better; rather, this form of rate variation is hardly tractable in the data, and many different processes alongside rate shifts can theoretically lead to that particular tip trait distribution. Similar to rate decreases, single internal branch shifts were typically not flagged up as being inadequately described across models, because a single internal branch has little impact on the likelihood of the model (except when the shifts have a large magnitude, Fig. 7a). Variable‐rates methods also showed good absolute fit when ran on trees and tip data simulated under single‐branch shifts and rate‐decreases scenarios; however, models estimated these rate changes with a similar true accuracy as other heterogeneity scenarios (Fig. 6).

Multiple branch increases had a high negative impact on model adequacy. Isolated terminal increases were particularly troublesome compared with whole‐clade events, potentially because single‐process models accommodate rate variation by changing estimated *σ*² on several branches adjacent to the ones presenting rate shifts. Thus, changes on nonclustered branches can cause a wide spread of falsely inferred rates. Similarly, BAMM shapes rate heterogeneity as a process across multiple branches, and it is less able to capture single‐branch shifts (Rabosky & Huang, 2016). In BAMM, detection of single‐branch shifts requires two events (i.e. nested rate shifts with modelling of an increase at the start of a branch followed by a subsequent decrease). In contrast, BayesTraits explicitly allows changes on single branches with one event. Accordingly, BAMM had poorer ARBUTUS diagnostics in the presence of isolated tip increases (Fig. 5) and overestimated rates of evolution on the untransformed branches in trees with simulated terminal rate changes (Fig. S2). However, the method accurately described heterogeneity in the form of whole‐clade rate increases. Also, the accuracy of estimates improved when using the more flexible BAMM‐flip version.

The root‐to‐tip rate‐decelerating process caused most spurious results across all models except BAMM. Even the EB model missed these processes in almost 20% of cases, particularly when a steep decrease was involved [*a *= log(0.05) or log(0.1)]. BayesTraits was also largely unable to describe early bursts (Figs 5 and 6). The lack of strength in modelling early bursts by models (except BAMM) was highlighted in the empirical data sets as well, and the EB was often not separated clearly from the BM in terms of relative fit, despite its superior adequacy in modelling temporal rate variation. These results add to the body of ideas advocating that early bursts are often not identified across data sets (Harmon *et al*., 2010; Slater *et al*., 2010; Venditti *et al*., 2011; Alhajeri *et al*., 2015) not necessarily because such scenarios are scarce in nature, but because current models do not have the power to detect them, and early shifts leave little signal in the tip data (Slater & Pennell, 2014).

The size of simulated trees did not generally affect the ability of variable‐rates models to recover rate shifts, and these methods were similarly robust for trees of 25 to 200 species. The detectability of rate shifts was largely influenced by the shift magnitude, and by whether a shift was on isolated branches or as part of a clade (for BayesTraits, grouped events were more easily detected). These results hence mirror the patterns of absolute adequacy seen throughout the main analyses, and variable‐rates models prove suitable for detecting heterogeneity even when the group of interest is fairly small. Similarly, we did not find the number of species involved in a clade event to affect the shift detectability; however, we only had data for clades ranging between 10 and 30 species. Conversely, the magnitude of the regime shift had a substantial effect on the model ability to recover the event, and most likely potential effects of a larger variability in clade sizes wane when the shift magnitude is taken into account; that is, small clades with a big magnitude shift will be successfully recovered by models (e.g. body mass evolution in the steamer ducks, Fig. S74), but for small magnitudes, a bigger clade might be needed. Of the two variable‐rates models included, BAMM‐flip showed some sensitivity to both tree and clade size, specifically regarding its ability to detect the larger rate shifts.

### Other limitations of variable‐rates models

BayesTraits generally approximated trait evolution with low inadequacy levels; however, the model did tend to overestimate total rate heterogeneity, mostly because it inferred multiple false terminal rate increases. We repeated the adequacy analyses on the simulated heterogeneity scenarios using the mean (rather than the median) branch lengths to summarize the posterior scaled trees from the variable‐rates models. Following this approach, BayesTraits clearly registered higher inadequacy levels (Table S4, Fig. S16), mostly determined by cases of extreme terminal increases with a low probability in the posterior that caused additional untrue terminal branch shifts in the averaged scaled trees. Approaches such as BayesTraits have been accused of overinflating rate variation before (Ho *et al*., 2014), mainly because of the relaxed/permissive nature of (default) priors. Further, our analyses on trees and trait data simulated with no rate shifts showed that although considerable rate shifts (i.e. > five‐fold) inferred using BayesTraits are probably supported by the data, more caution is needed when making inferences about smaller (< two‐fold) rate changes at the tips.

BAMM was prone to underestimations of total rate variation and an inability to account for isolated tip increases, expected as heterogeneity is modelled in a less flexible framework compared with BayesTraits (Rabosky & Huang, 2016). Allowing the model to flip between time‐varying and time‐constant processes did, however, improve fit in comparison with the constrained time‐varying version (Fig. S4a). Further, BAMM showed an inclination towards rate‐decelerating processes, as shown by (i) a negative S.HGT ubiquitously across the analyses, (ii) the distributions of the rate‐change parameters governing the root regime (*β*) and (iii) the comparison between estimated and true rates on branches with no simulated rate shifts. Therefore, BAMM tends to infer some false early bursts in both the presence and absence of rate heterogeneity, but the intensity and prevalence of these erroneous inferences is low. Using a BAMM‐flip alternative also reduces the occurrence of false rate bursts; however, BayesTraits still showed best true fit under the assumption of homogeneity in rates (Fig. S2).

There are several other approaches to rate heterogeneity in trait evolution, and a notable body of such models use parametric methods to model a distribution of evolutionary rates that allows jumps (e.g. Landis *et al*., 2013; Elliot & Mooers, 2014). Elliot & Mooers (2014) method is readily available in StableTraits; however, the outputted scaled tree (i.e. a tree with branches scaled by the rate of trait evolution) cannot be equated with a parameterized global transformation of the branch lengths. Hence, we could not use the output of StableTraits to build the unit tree in ARBUTUS. Pennell *et al*. (2015) also warn that jump methods are not (yet) compatible with the ARBUTUS framework. Further, BayesTraits is a nonparametric approach, and the single‐lineage bursts are likely a good approximation of a rate jump. Thus, we believe that jump methods would produce similar patterns in the evolutionary process, and record similar adequacy levels with BayesTraits.

### Absolute vs. relative model fit in the presence of rate heterogeneity

A relative preference for the OU model (and derivatives) over other single‐process models is widespread in the literature (e.g. Collar *et al*., 2009; Harmon *et al*., 2010; Blackburn *et al*., 2013; Knope & Scales, 2013; Price & Hopkins, 2015), but there are many challenges attributed to estimation and interpretation of this model (Ho *et al*., 2014; Cooper *et al*., 2016). Pennell *et al*. (2015) found the OU method is largely inadequate even though it predominantly scored highest AICw over the BM and EB models on angiosperm data sets. Our adequacy analyses also linked high relative fit for OU methods with cases of high inadequacy for all single‐process models included, across both simulated and empirical data sets. Particularly, when species record very high rates of evolution late in the phylogeny (especially nonclustered species), the OU model is favoured by relative selection criteria over other approaches. The link between inadequacy levels and model relative fit was stronger across the simulated compared with the empirical data, likely due to the existence of other evolutionary processes besides rate shifts that affect relative fit across avian data sets. Nonetheless, often a high relative fit for the OU model was a consequence of rate heterogeneity, and not of body mass evolution under an OU‐type process. Not accounting for measurement error could have also caused a biased preference for the OU model across the empirical data sets (Silvestro *et al*., 2015); however, the link between late rate heterogeneity and a bias for the OU model clearly emerges from the results on the simulated data sets, ruling out the possibility that measurement error is solely responsible for the biased selection criteria across the avian data sets.

## Conclusions

Evolutionary models continue to be developed to approximate the macroevolutionary process with a higher degree of realism, by dealing with increasingly complex deviations from a simple process. Here we used a large data set of avian body mass to show that variation in the rate at which traits change can be a common event in relatively small phylogenetic clades (up to hundreds of species). We further used both empirical data and simulated rate‐heterogeneity scenarios to show that allowing rates of evolution to vary in the absence of *a priori* assumptions about the magnitude or location of shifts represents a reliable method to pattern trait evolution. Variable‐rates approaches do have limitations; heterogeneity in the form of rate decreases and single‐branch changes is hard to detect and generates poor method fit. Further, rate increases on terminal branches can be poorly approximated even when allowing for rate variation, and early bursts in particular are often misquantified by BayesTraits. However, we show that interpretation can be guided by the use of absolute adequacy tests. We also underline the potential for misleading inferences when using relative model selection criteria only, for example missing early bursts or favouring OU‐type processes when late rate variation is present. This work does not invalidate the concepts behind standard single‐process methods; rather, we advise using the more flexible applications of these approaches (e.g. implementation of EB and OU models in a Bayesian framework; Pennell *et al*., 2014; Uyeda & Harmon, 2014).

## Supporting information


**Appendix S1** Implementation of BayesTraits and BAMM models.
**Table S1** Model inadequacy levels across a simulated constant rate‐deceleration process from root to tips, and a simulated rate‐burst followed by a gradual decrease within a clade.
**Table S2** Frequency at which BayesTraits infers rate shifts in the absence of rate‐heterogeneity (i.e. on trees and associated tip‐data simulated under a BM mode of evolution).
**Table S3** Frequency of positive significant differences (P < 0.05) between test statistics across key ARBUTUS diagnostics; results on the empirical data.
**Table S4** Model inadequacy levels across a simulated constant rate‐deceleration process from root to tips, and a simulated rate‐burst followed by a gradual decrease within a clade. Results when models are fitted using mean scaled trees.
**Table S5** BayesFactor (BF) evidence for alternative models with various numbers of rate‐shifts given by BAMM‐flip across the empirical datasets.
**Figures S1–S5** Model fit in the presence of simulated rate‐heterogeneity.
**Figures S6–S11** The influence of tree size on model ability to detect rate shifts.
**Figures S12–S14** Tendency of variable‐rates models to overfit.
**Figure S15** Rate heterogeneity and general absolute adequacy on empirical data.
**Figure S16** Absolute Adequacy on Simulated datasets – results on mean scaled trees.
**Figures S17–S104** Avian trees scaled by the rate of body mass evolution as described by BayesTraits and BAMM.
