# Supplementary material for: The impact of rate heterogeneity on inference of phylogenetic models of trait evolution
Source: J Evol Biol. 2016 Oct 4;29(12):2502–18. doi: 10.1111/jeb.12979 (PMC5217074; doi:10.1111/jeb.12979)
Supplement: Supplementary file 1 — Appendix S1 Implementation of BayesTraits and BAMM models. Table S1 Model inadequacy levels across a simulated constant rate‐deceleration process from root to tips, and a simulated rate‐burst followed by a gradual decrease within a clade. Table S2 Frequency at which BayesTraits infers rate shifts in the absence of rate‐heterogeneity (i.e. on trees and associated tip‐data simulated under a BM mode of evolution). Table S3 Frequency of positive significant differences (P < 0.05) between test statistics across key ARBUTUS diagnostics; results on the empirical data. Table S4 Model inadequacy levels across a simulated constant rate‐deceleration process from root to tips, and a simulated rate‐burst followed by a gradual decrease within a clade. Results when models are fitted using mean scaled trees. Table S5 BayesFactor (BF) evidence for alternative models with various numbers of rate‐shifts given by BAMM‐flip across the empirical datasets. Figures S1–S5 Model fit in the presence of simulated rate‐heterogeneity. Figures S6–S11 The influence of tree size on model ability to detect rate shifts. Figures S12–S14 Tendency of variable‐rates models to overfit. Figure S15 Rate heterogeneity and general absolute adequacy on empirical data. Figure S16 Absolute Adequacy on Simulated datasets – results on mean scaled trees. Figures S17–S104 Avian trees scaled by the rate of body mass evolution as described by BayesTraits and BAMM. [file JEB-29-2502-s001.pdf]

SUPPLEMENTARY INFORMATION

TABLE OF CONTENTS

Implementation of BayesTraits and BAMM models 2

Tables S1-S4 3

Model fit in the presence of simulated rate-heterogeneity: Figures S1-S5 7

The influence of tree size on model ability to detect rate shifts: Figures S6-S11 22

Tendency of variable-rates models to overfit: Figures S12-S14 28

Rate heterogeneity and general absolute adequacy on empirical data: Figure S15 32

Absolute Adequacy on Simulated datasets – results on mean scaled trees: Figure S16 34

BayesFactor (BF) evidence for alternative models with various numbers of rate-shifts given by BAMM-flip across the empirical datasets: Table S5 36

Avian trees scaled by the rate of body mass evolution as described by BayesTraits and BAMM: Figures S17-S104 42

Implementation of BayesTraits and BAMM models

The Variable Rates model in BayesTraits was used with 25 million iterations, sampling every 20000 generations and discarding the 20% iterations as burn in. This left a sample of 1000 scaled-trees. MCMC chains were re-run if the effective sample size (ESS) for the likelihood, alpha, sigma or number of variable rates was smaller than 200. Further, trace and autocorrelation plots were visualised to ensure convergence. Lastly, we checked the multivariate and individual potential scale reduction factor for each variable, and values of psrf > 1.1 were taken as evidence that the runs had not mixed. Four independent chains were used for each tree, and, if converged, estimates from all four were put together. For BAMM, the MCMC chain was run with 30 million iterations, sampling every 15000 generations, so that 2000 samples remained recorded. The burn in period was set for 25%. MCMC chains were re-run if the ESS for the number of shifts, logPrior, log likelihood, event or acceptance rates was under 200. In a limited number of simulated trees, within chain convergence was not successful even after four repeated runs; these trees were further discarded from the analyses (2 cases in the discrete simulations, 2 cases for the gradual deceleration within a clade, and 1 case for the constant-rate deceleration process from root to tips). Similar to BayesTraits, between chain convergence was checked using the potential scale reduction factor, and, if the runs had successfully mixed, estimates for four independent chains were combined.

Table S1. Model inadequacy levels (quantified as frequency of inadequate trees and associated trait data) across (a) a simulated constant rate-deceleration process from root to tips, and (b) a simulated rate-burst followed by gradual decreases within a clade. *a* represents the rate-change parameter. Inadequacy levels measure model ability to account for total rate variation (C.VAR), variation related to time (S.HGT), branch lengths (S.VAR), and ancestral states (S.ASR). A majority of positive or negative significant ($P < 0.05$) differences between test statistics is signalled by (+) and (-), respectively.

Model	*a*	Inadequacy levels			
		C.VAR	S.HGT	S.VAR	S.ASR
a) Root-phylogeny process					
BM	ln(0.05)	0.36 (+)	0.88 (+)	0.76 (+)	0.02 (+)
	ln(0.1)	0.14 (+)	0.67 (+)	0.46 (+)	0.02 (+)
	ln(0.2)	0.1 (+)	0.44 (+)	0.29 (+)	0.02 (+)
	ln(0.5)	0.06 (+)	0.09 (+)	0.06 (+)	0
OU	ln(0.05)	0.37 (+)	0.89 (+)	0.77 (+)	0
	ln(0.1)	0.13 (+)	0.68 (+)	0.43 (+)	0.02 (+)
	ln(0.2)	0.09 (+)	0.44 (+)	0.28 (+)	0.01 (+)
	ln(0.5)	0.07 (-)	0.06 (+)	0.09 (+)	0
EB	ln(0.05)	0.06 (+)	0.31 (+)	0.26 (+)	0.02 (+)
	ln(0.1)	0.09 (-)	0.18 (+)	0.23 (+)	0.04 (+)
	ln(0.2)	0.08 (+)	0.13 (+)	0.13 (+)	0.03 (+)
	ln(0.5)	0.05 (-)	0.03 (+)	0.02 (+)	0.01 (+)
	ln(0.05)	0.08 (+)	0.56 (+)	0.75 (+)	0.02 (+)
BayesTraits	ln(0.05)	0.08 (+)	0.56 (+)	0.75 (+)	0.02 (+)
	ln(0.1)	0.03 (+)	0.42 (+)	0.38 (+)	0
	ln(0.2)	0.04 (+)	0.29 (+)	0.25 (+)	0.02 (+)
	ln(0.5)	0.05 (-)	0.05 (+)	0.09 (+)	0
BAMM	ln(0.05)	0.04 (+)	0.11 (+)	0.16 (+)	0.01 (+)
	ln(0.1)	0.04 (-)	0.04 (+)	0.06 (+)	0.02 (+)
	ln(0.2)	0.07 (-)	0	0.03 (+)	0.03 (+)
	ln(0.5)	0.05 (-)	0	0.01 (-)	0
BAMM-flip	ln(0.05)	0.04 (+)	0.11 (+)	0.14 (+)	0.01 (+)
	ln(0.1)	0.06 (+)	0.05(+)	0.10 (+)	0.02 (+)
	ln(0.2)	0.06 (+)	0.01 (+)	0.02 (+)	0.02 (+)
	ln(0.5)	0.06 (-)	0	0.02 (+)	0
b) Clade process					
BM	ln(0.05)	0.17 (+)	0.05 (+)	0.03 (+)	0.14 (+)
	ln(0.1)	0.18 (+)	0.05 (+)	0.11 (+)	0.02 (-)
	ln(0.2)	0.07 (+)	0.07 (+)	0.1 (+)	0.11 (-)
	ln(0.5)	0.14 (+)	0.08 (-)	0.05 (-)	0.17 (+)

OU	ln(0.05)	0.16 (+)	0.06 (+)	0.06 (+)	0.09 (+)
	ln(0.1)	0.2 (+)	0.06 (+)	0.13 (+)	0.02 (-)
	ln(0.2)	0.07 (+)	0.05 (+)	0.09 (+)	0.07 (-)
	ln(0.5)	0.14 (+)	0.03 (+)	0.02 (+)	0.09 (+)
EB	ln(0.05)	0.16 (+)	0.01 (+)	0.04 (+)	0.16 (+)
	ln(0.1)	0.17 (+)	0.03 (+)	0.09 (+)	0.03 (-)
	ln(0.2)	0.08 (+)	0.03 (-)	0.08 (+)	0.12 (-)
	ln(0.5)	0.14 (+)	0.06 (-)	0.05 (+)	0.16 (+)
BayesTraits	ln(0.05)	0.04 (+)	0.02 (+)	0.07 (+)	0.03 (+)
	ln(0.1)	0.07 (+)	0.07 (+)	0.13 (+)	0.01 (-)
	ln(0.2)	0.04 (-)	0.07 (+)	0.09 (+)	0.03 (-)
	ln(0.5)	0.07 (-)	0.05 (-)	0.07 (+)	0.05 (+)
BAMM	ln(0.05)	0.05 (+)	0.04 (-)	0	0.06 (+)
	ln(0.1)	0.07 (+)	0.02 (-)	0.04 (+)	0.02 (+)
	ln(0.2)	0.04 (+)	0.03 (-)	0.02 (+)	0.06 (-)
	ln(0.5)	0.08 (-)	0.05 (-)	0.02 (-)	0.05 (+)
BAMM-flip	ln(0.05)	0.04 (+)	0.11 (-)	0.14 (+)	0.01 (+)
	ln(0.1)	0.06 (+)	0.05 (+)	0.10 (+)	0.02 (+)
	ln(0.2)	0.06 (+)	0.01 (+)	0.02 (+)	0.02 (-)
	ln(0.5)	0.06 (-)	0	0.02 (+)	0

Table S2. Frequency at which BayesTraits infers rate shifts in the absence of rate-heterogeneity (i.e. on trees and associated tip-data simulated under a BM mode of evolution). Results for various thresholds at which a rate change (i.e. the ratio between the branch length estimated by BayesTraits and length of the identical branch in the input phylogeny) is considered a rate shift.

Shift threshold	Proportion of trees with rate shifts	Proportion of trees with terminal rate shifts	Number of terminal branch shifts / tree
< 0.05	0 %	NA	NA
< 0.1	0 %	NA	NA
< 0.2	0.1 %	0 %	NA
< 0.5	0.2 %	0 %	NA
< 0.7	5.8 %	0 %	NA
all shifts in [0.5,1.5]	70.5 %		
> 1.5	29.3 %	24.7 %	1-15
> 2	26.2 %	21.4 %	1-10
> 5	8.5 %	5.6 %	1-6
> 10	0.5 %	0.2 %	1
> 20	0 %	0 %	NA

Table S3. Frequency of positive significant differences (P < 0.05) between test statistics across key ARBUTUS diagnostics; results on the empirical data.

Model	C.VAR	S.HGT	S.VAR	S.ASR
BM	1	0.74	0.36	0.69
OU	0.94	0.95	0.48	0.88
EB	0.87	0.5	0.16	0.76
BayesTraits	1	0.88	1	1
BAMM	1	0.17	0.2	0.88
BAMM-flip	1	0.56	0.57	1

Table S4. Model inadequacy levels (quantified as frequency of inadequate trees and associated trait data) across (a) a simulated constant rate-deceleration process from root to tips, and (b) a simulated rate-burst followed by gradual decreases within a clade. *a* represents the rate-change parameters. Inadequacy levels measure model ability to account for total rate variation (C.VAR), and further, variation related to time (S.HGT), branch lengths (S.VAR), and ancestral states (S.ASR). A majority of positive or negative significant (P < 0.05) differences between test statistics is signalled by (+) and (-), respectively. Results on the mean scaled trees from the output of BayesTraits.

Model	*a*	Inadequacy levels			
		C.VAR	S.HGT	S.VAR	S.ASR
a) Root-phylogeny process					
Bayes Traits	ln(0.05)	0.11 (-)	0.40 (+)	0.92 (+)	0 (+)
	ln(0.1)	0.10 (-)	0.31 (+)	0.76 (+)	0
	ln(0.2)	0.20 (-)	0.30 (+)	0.62 (+)	0
	ln(0.5)	0.25 (-)	0.08 (+)	0.37 (+)	0
b) Clade process					
Bayes Traits	ln(0.05)	0.1 (-)	0.06 (+)	0.51 (+)	0.01 (+)
	ln(0.1)	0.14 (-)	0.04 (+)	0.48 (+)	0
	ln(0.2)	0.21 (-)	0.09 (+)	0.34 (+)	0.02 (+)
	ln(0.5)	0.23 (-)	0	0.28	0.02 (+)

Figure S1. Model inadequacy levels (quantified as the frequency of trees and associated tip-data where the focal model was inadequate) across a simulated Brownian Motion processes (no shifts i.e. shift magnitude = 1) and rate-heterogeneity scenarios: internal branch shift; clade event; rate-changes on isolated, terminal branches; rate-burst followed by gradual decreases within a clade, and constant rate-deceleration process from root to tips. Single-process (BM, OU and EB) and variable-rates models (BayesTraits and BAMM with time-flip proposal) are considered. Inadequacy levels measure model ability to account for variation related to (a) branch lengths, and (b) ancestral states. Inadequacy is quantified separately for rate increases (inc, up-pointing triangles) and decreases (dec, down-pointing triangles), and the exact magnitude of each shift is highlighted by the white-black colour scheme. For scenarios involving gradual rate-changes, the natural logarithm of the shift magnitude represents the constant rate-change parameter.

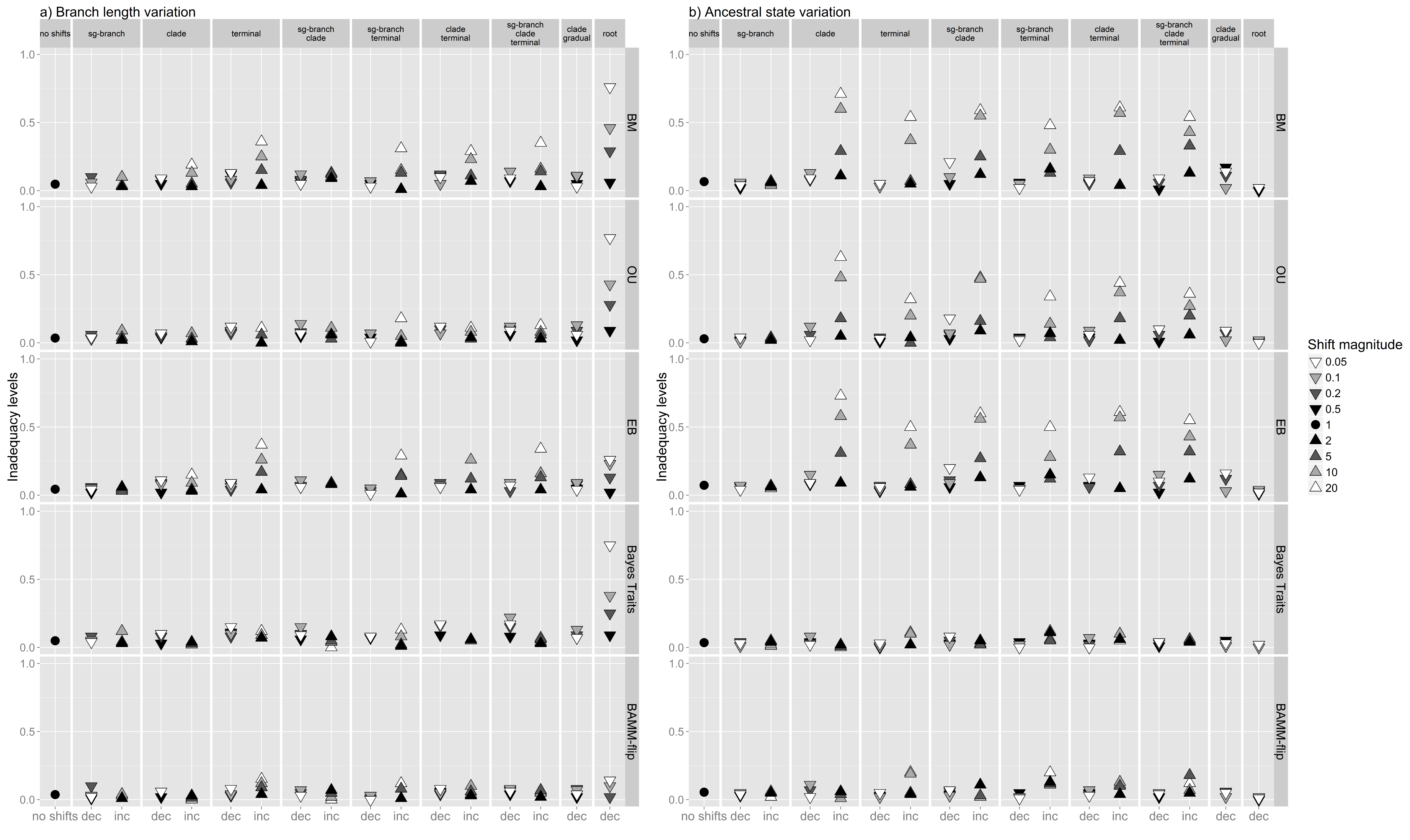

a) Branch length variation
b) Ancestral state variation
no shifts
sg-branch
clade
terminal
sg-branch clade
sg-branch terminal
clade terminal
sg-branch clade terminal
clade gradual
root
BM
OU
EB
Bayes Traits
BAMM-flip
Inadequacy levels
dec
inc
Shift magnitude
0.05
0.1
0.2
0.5
1
2
5
10
20

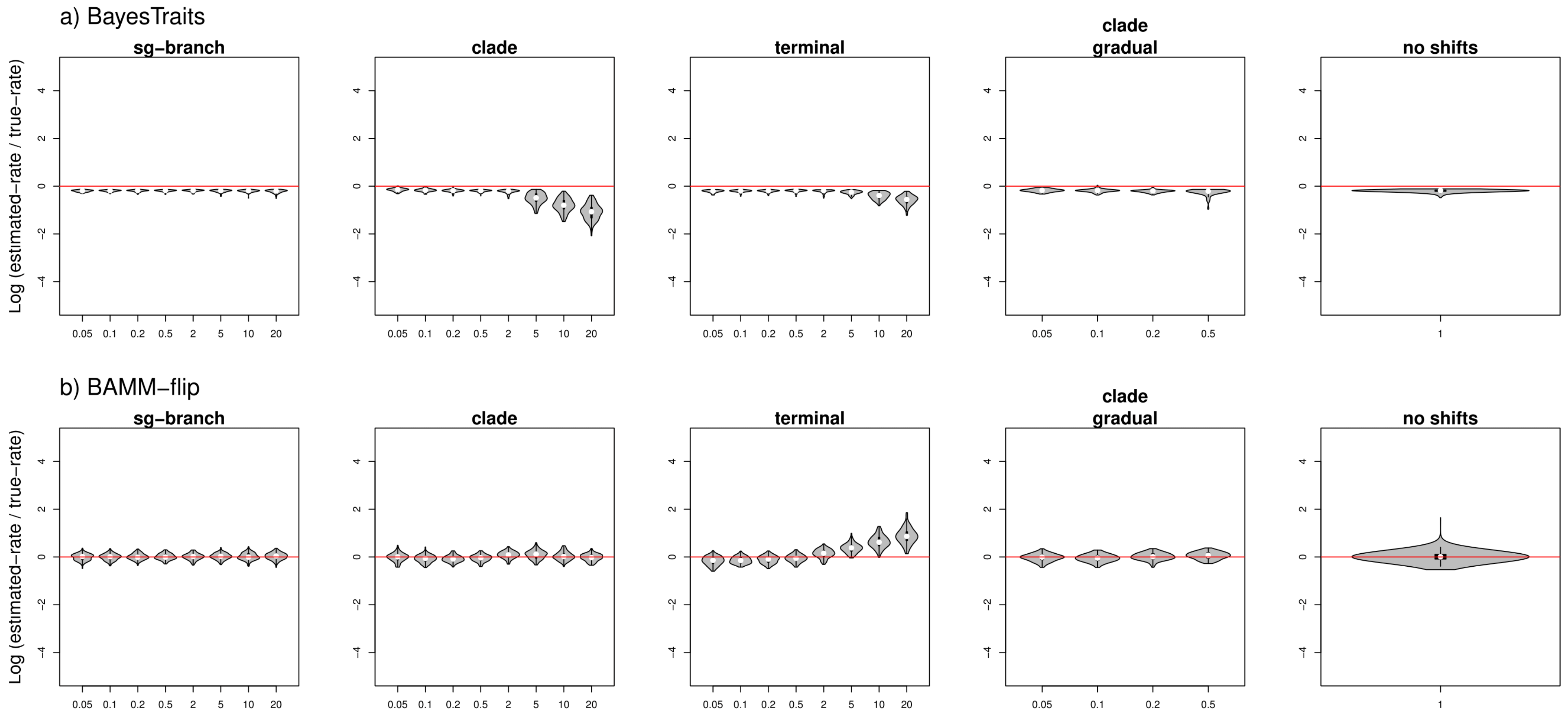

Figure S2. Distributions of log-proportions between rates estimated by variable-rates models and the true (simulated) rates of evolution. Only branches with no simulated rate-changes are considered (i.e. true $\sigma^2 = 1$). Distributions are shown for various shift magnitudes (x-axis) and heterogeneity scenarios: internal branch shift; clade event; rate-changes on isolated, terminal branches; rate-burst followed by gradual decreases within a clade. Per-branch comparisons within trees with no simulated rate-shifts are also shown. Results for BayesTraits (a) and BAMM-flip (b).

Figure. S3. (a) Distributions of log-proportions between rates estimated by variable-rates models and the true (simulated) rate-changes on the identical transformed branches. Distributions are shown for various shift magnitudes (x-axis) and combinations of heterogeneity scenarios: internal branch shift; clade event; rate-changes on isolated, terminal branches. (b) Per-branch comparisons shown for the non-transformed branches in each rate-heterogeneity scenario. Results for BayesTraits and BAMM-flip.

BayesTraits

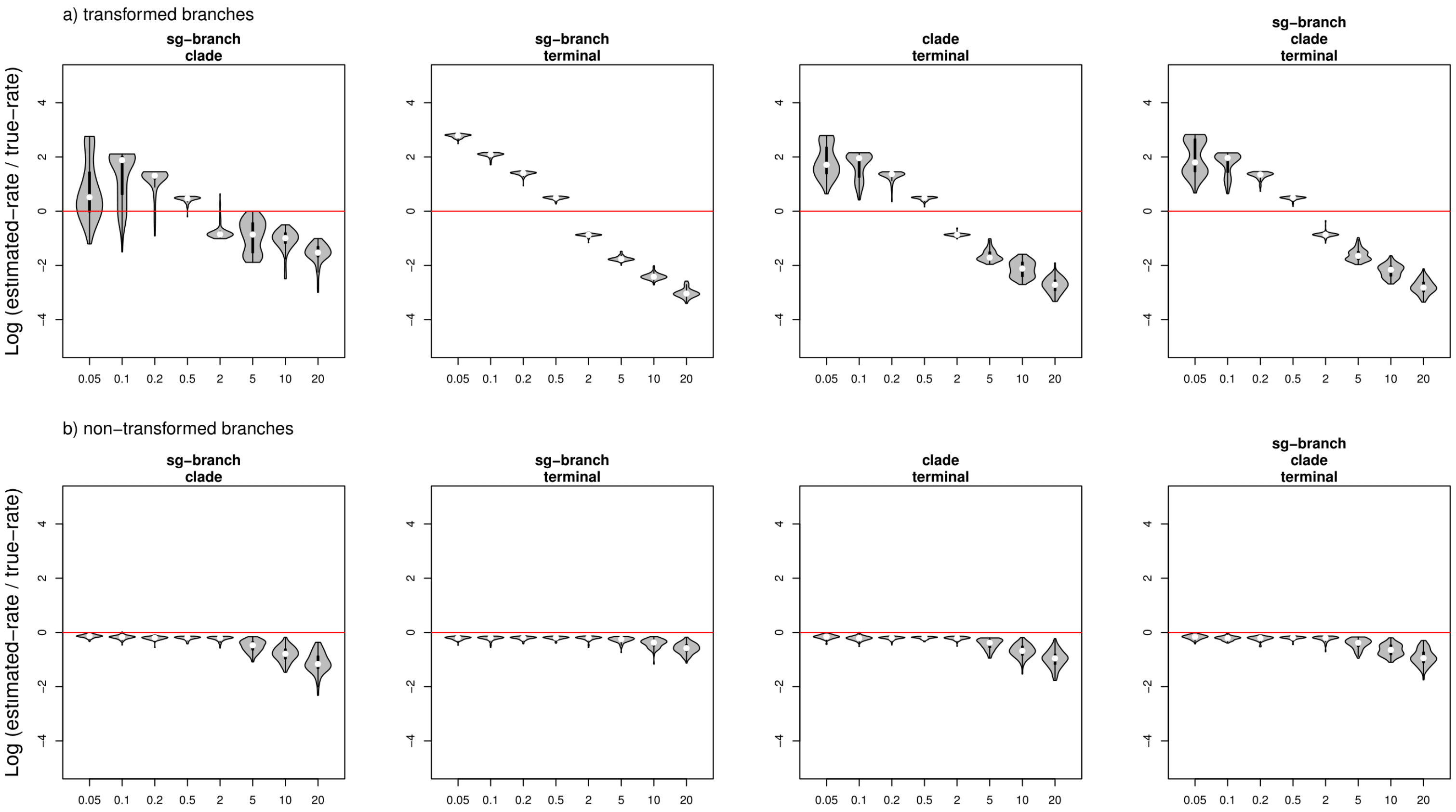

BAMM_flip

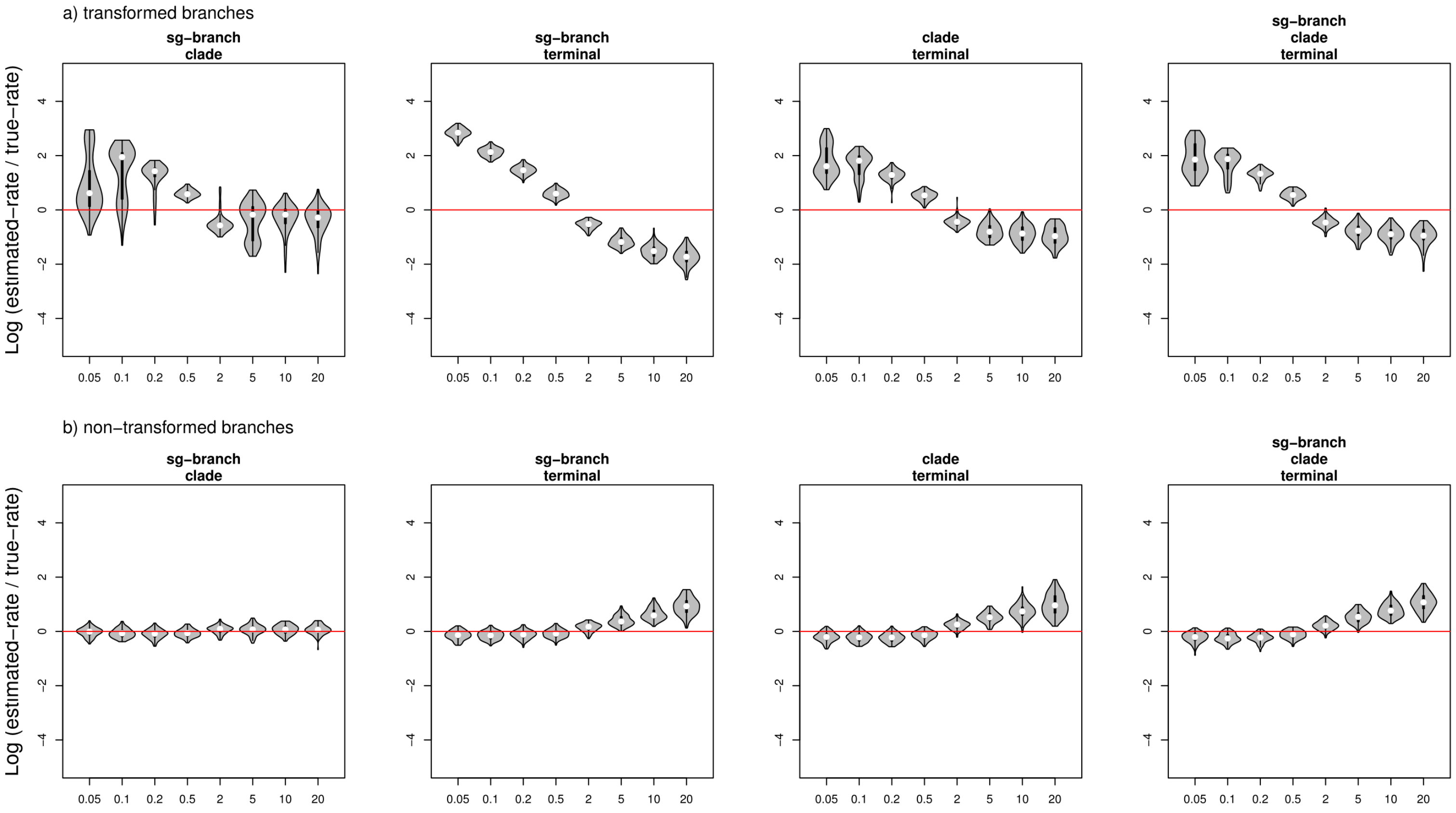

Figure S4. Model inadequacy levels (quantified as frequency of inadequate trees and associated trait data) across simulated rate-heterogeneity scenarios: internal branch shift, clade event, rate-change on isolated, terminal branches, and combinations. The following models are considered: BM, OU, EB, BayesTraits, and two BAMM alternatives: constrained to time-varying processes only (BAMM), and allowed to flip between time-varying and time-constant trait-change (BAMM-flip). Inadequacy levels measure model ability to account for (a) total variation, and further, variation related to (b) time, (c) branch lengths, and (d) ancestral states. Each column corresponds to set shift-magnitudes. Inadequacy levels for each specific model are also highlighted by the blue-red colour scheme.

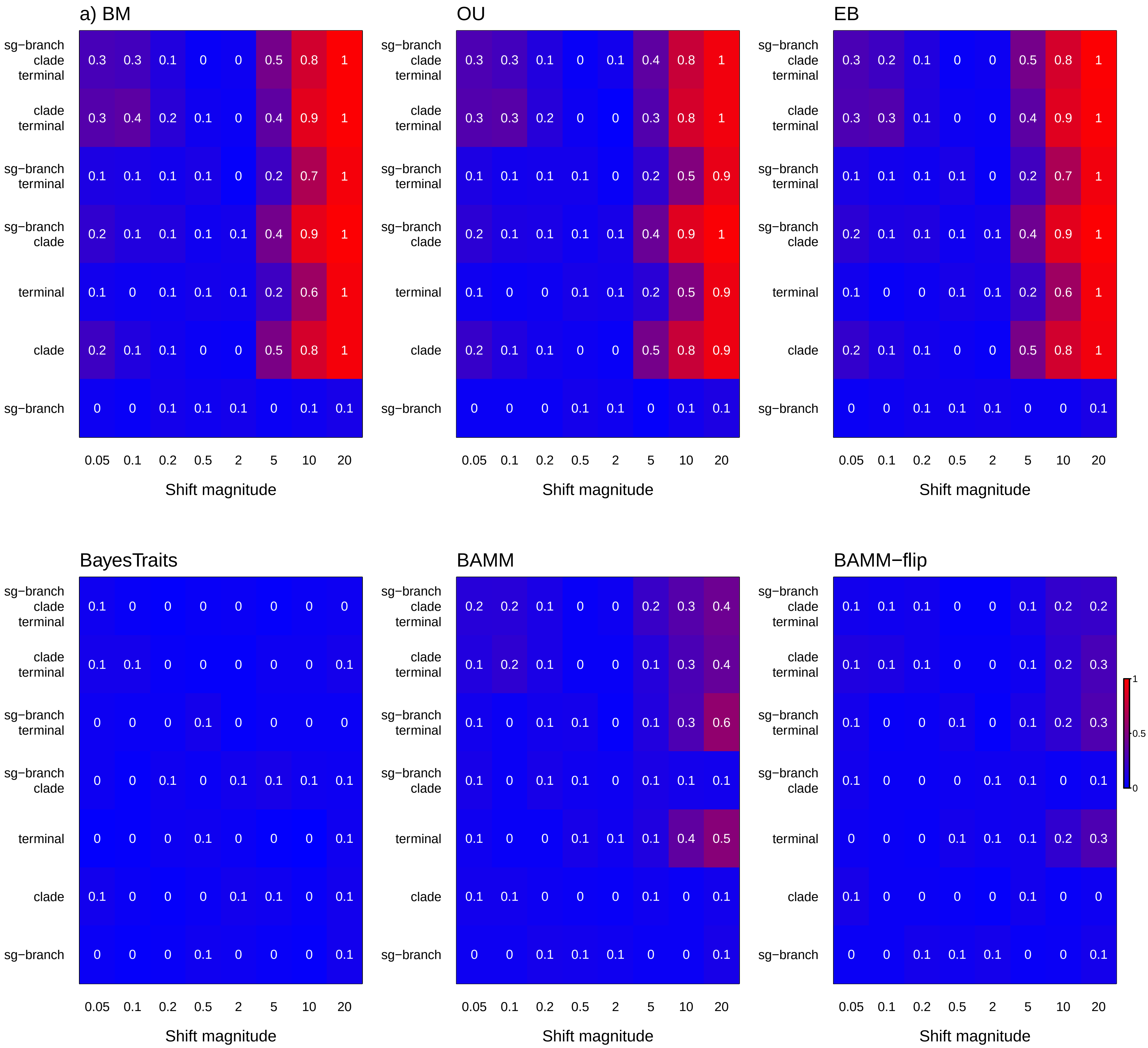
a) BM
sg−branch clade terminal 0.3 0.3 0.1 0 0 0.5 0.8 1
clade terminal 0.3 0.4 0.2 0.1 0 0.4 0.9 1
sg−branch terminal 0.1 0.1 0.1 0.1 0 0.2 0.7 1
sg−branch clade 0.2 0.1 0.1 0.1 0.1 0.4 0.9 1
terminal 0.1 0 0.1 0.1 0.1 0.2 0.6 1
clade 0.2 0.1 0.1 0 0 0.5 0.8 1
sg−branch 0 0 0.1 0.1 0.1 0 0.1 0.1
0.05 0.1 0.2 0.5 2 5 10 20
Shift magnitude
OU
sg−branch clade terminal 0.3 0.3 0.1 0 0.1 0.4 0.8 1
clade terminal 0.3 0.3 0.2 0 0 0.3 0.8 1
sg−branch terminal 0.1 0.1 0.1 0.1 0 0.2 0.5 0.9
sg−branch clade 0.2 0.1 0.1 0.1 0.1 0.4 0.9 1
terminal 0.1 0 0 0.1 0.1 0.2 0.5 0.9
clade 0.2 0.1 0.1 0 0 0.5 0.8 0.9
sg−branch 0 0 0 0.1 0.1 0 0.1 0.1
0.05 0.1 0.2 0.5 2 5 10 20
Shift magnitude
EB
sg−branch clade terminal 0.3 0.2 0.1 0 0 0.5 0.8 1
clade terminal 0.3 0.3 0.1 0 0 0.4 0.9 1
sg−branch terminal 0.1 0.1 0.1 0.1 0 0.2 0.7 1
sg−branch clade 0.2 0.1 0.1 0.1 0.1 0.4 0.9 1
terminal 0.1 0 0 0.1 0.1 0.2 0.6 1
clade 0.2 0.1 0.1 0 0 0.5 0.8 1
sg−branch 0 0 0.1 0.1 0.1 0 0 0.1
0.05 0.1 0.2 0.5 2 5 10 20
Shift magnitude
BayesTraits
sg−branch clade terminal 0.1 0 0 0 0 0 0 0
clade terminal 0.1 0.1 0 0 0 0 0 0.1
sg−branch terminal 0 0 0 0.1 0 0 0 0
sg−branch clade 0 0 0.1 0 0.1 0.1 0.1 0.1
terminal 0 0 0 0.1 0 0 0 0.1
clade 0.1 0 0 0 0.1 0.1 0 0.1
sg−branch 0 0 0 0.1 0 0 0 0.1
0.05 0.1 0.2 0.5 2 5 10 20
Shift magnitude
BAMM
sg−branch clade terminal 0.2 0.2 0.1 0 0 0.2 0.3 0.4
clade terminal 0.1 0.2 0.1 0 0 0.1 0.3 0.4
sg−branch terminal 0.1 0 0.1 0.1 0 0.1 0.3 0.6
sg−branch clade 0.1 0 0.1 0.1 0 0.1 0.1 0.1
terminal 0.1 0 0 0.1 0.1 0.1 0.4 0.5
clade 0.1 0.1 0 0 0 0.1 0 0.1
sg−branch 0 0 0.1 0.1 0.1 0 0 0.1
0.05 0.1 0.2 0.5 2 5 10 20
Shift magnitude
BAMM−flip
sg−branch clade terminal 0.1 0.1 0.1 0 0 0.1 0.2 0.2
clade terminal 0.1 0.1 0.1 0 0 0.1 0.2 0.3
sg−branch terminal 0.1 0 0 0.1 0 0.1 0.2 0.3
sg−branch clade 0.1 0 0 0 0.1 0.1 0 0.1
terminal 0 0 0 0.1 0.1 0.1 0.2 0.3
clade 0.1 0 0 0 0 0.1 0 0
sg−branch 0 0 0.1 0.1 0.1 0 0 0.1
0.05 0.1 0.2 0.5 2 5 10 20
Shift magnitude
1
0.5
0

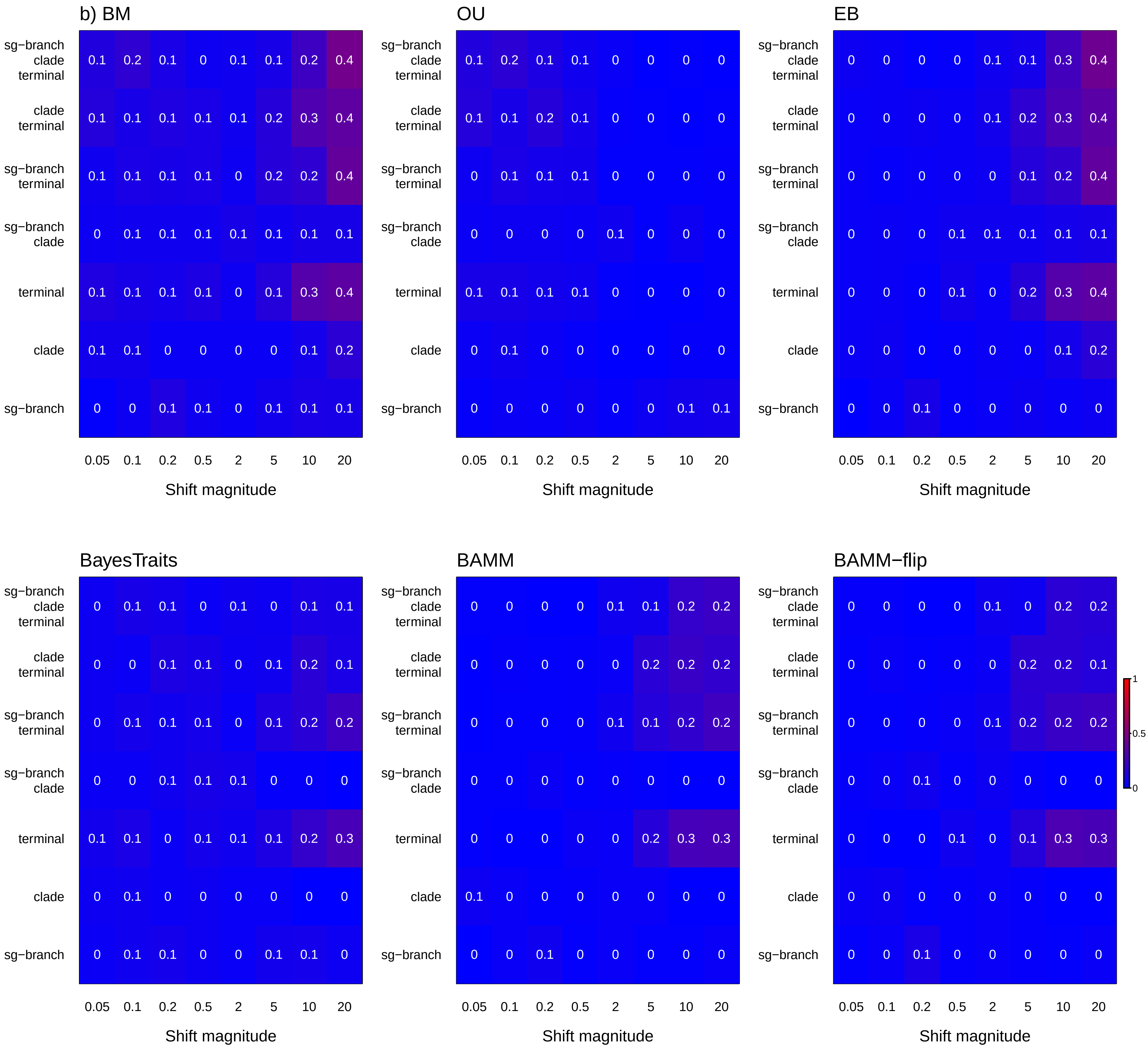
b) BM
sg−branch clade terminal 0.1 0.2 0.1 0 0.1 0.1 0.2 0.4
clade terminal 0.1 0.1 0.1 0.1 0.1 0.2 0.3 0.4
sg−branch terminal 0.1 0.1 0.1 0.1 0 0.2 0.2 0.4
sg−branch clade 0 0.1 0.1 0.1 0.1 0.1 0.1 0.1
terminal 0.1 0.1 0.1 0.1 0 0.1 0.3 0.4
clade 0.1 0.1 0 0 0 0 0.1 0.2
sg−branch 0 0 0.1 0.1 0 0.1 0.1 0.1
0.05 0.1 0.2 0.5 2 5 10 20
Shift magnitude
OU
sg−branch clade terminal 0.1 0.2 0.1 0.1 0 0 0 0
clade terminal 0.1 0.1 0.2 0.1 0 0 0 0
sg−branch terminal 0 0.1 0.1 0.1 0 0 0 0
sg−branch clade 0 0 0 0 0.1 0 0 0
terminal 0.1 0.1 0.1 0.1 0 0 0 0
clade 0 0.1 0 0 0 0 0 0
sg−branch 0 0 0 0 0 0 0.1 0.1
0.05 0.1 0.2 0.5 2 5 10 20
Shift magnitude
EB
sg−branch clade terminal 0 0 0 0 0.1 0.1 0.3 0.4
clade terminal 0 0 0 0 0.1 0.2 0.3 0.4
sg−branch terminal 0 0 0 0 0 0.1 0.2 0.4
sg−branch clade 0 0 0 0.1 0.1 0.1 0.1 0.1
terminal 0 0 0 0.1 0 0.2 0.3 0.4
clade 0 0 0 0 0 0 0.1 0.2
sg−branch 0 0 0.1 0 0 0 0 0
0.05 0.1 0.2 0.5 2 5 10 20
Shift magnitude
BayesTraits
sg−branch clade terminal 0 0.1 0.1 0 0.1 0 0.1 0.1
clade terminal 0 0 0.1 0.1 0 0.1 0.2 0.1
sg−branch terminal 0 0.1 0.1 0.1 0 0.1 0.2 0.2
sg−branch clade 0 0 0.1 0.1 0.1 0 0 0
terminal 0.1 0.1 0 0.1 0.1 0.1 0.2 0.3
clade 0 0.1 0 0 0 0 0 0
sg−branch 0 0.1 0.1 0 0 0.1 0.1 0
0.05 0.1 0.2 0.5 2 5 10 20
Shift magnitude
BAMM
sg−branch clade terminal 0 0 0 0 0.1 0.1 0.2 0.2
clade terminal 0 0 0 0 0 0.2 0.2 0.2
sg−branch terminal 0 0 0 0 0.1 0.1 0.2 0.2
sg−branch clade 0 0 0 0 0 0 0 0
terminal 0 0 0 0 0 0.2 0.3 0.3
clade 0.1 0 0 0 0 0 0 0
sg−branch 0 0 0.1 0 0 0 0 0
0.05 0.1 0.2 0.5 2 5 10 20
Shift magnitude
BAMM−flip
sg−branch clade terminal 0 0 0 0 0.1 0 0.2 0.2
clade terminal 0 0 0 0 0 0.2 0.2 0.1
sg−branch terminal 0 0 0 0 0.1 0.2 0.2 0.2
sg−branch clade 0 0 0.1 0 0 0 0 0
terminal 0 0 0 0.1 0 0.1 0.3 0.3
clade 0 0 0 0 0 0 0 0
sg−branch 0 0 0.1 0 0 0 0 0
0.05 0.1 0.2 0.5 2 5 10 20
Shift magnitude
1
0.5
0

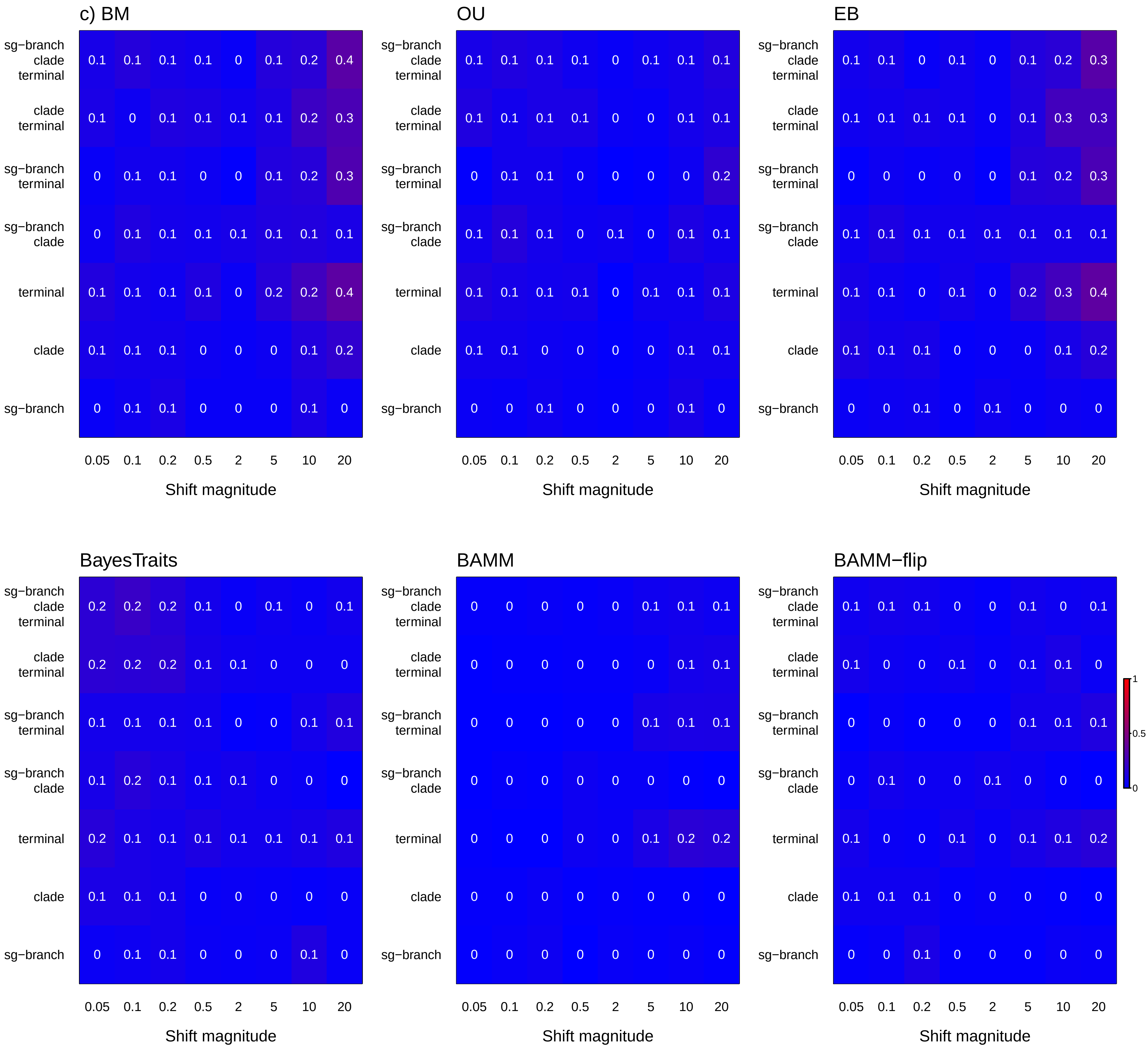

c) BM
sg−branch clade terminal 0.1 0.1 0.1 0.1 0 0.1 0.2 0.4
clade terminal 0.1 0 0.1 0.1 0.1 0.1 0.2 0.3
sg−branch terminal 0 0.1 0.1 0 0 0.1 0.2 0.3
sg−branch clade 0 0.1 0.1 0.1 0.1 0.1 0.1 0.1
terminal 0.1 0.1 0.1 0.1 0 0.2 0.2 0.4
clade 0.1 0.1 0.1 0 0 0 0.1 0.2
sg−branch 0 0.1 0.1 0 0 0 0.1 0
0.05 0.1 0.2 0.5 2 5 10 20
Shift magnitude
OU
sg−branch clade terminal 0.1 0.1 0.1 0.1 0 0.1 0.1 0.1
clade terminal 0.1 0.1 0.1 0.1 0 0 0.1 0.1
sg−branch terminal 0 0.1 0.1 0 0 0 0 0.2
sg−branch clade 0.1 0.1 0.1 0 0.1 0 0.1 0.1
terminal 0.1 0.1 0.1 0.1 0 0.1 0.1 0.1
clade 0.1 0.1 0 0 0 0 0.1 0.1
sg−branch 0 0 0.1 0 0 0 0.1 0
0.05 0.1 0.2 0.5 2 5 10 20
Shift magnitude
EB
sg−branch clade terminal 0.1 0.1 0 0.1 0 0.1 0.2 0.3
clade terminal 0.1 0.1 0.1 0.1 0 0.1 0.3 0.3
sg−branch terminal 0 0 0 0 0 0.1 0.2 0.3
sg−branch clade 0.1 0.1 0.1 0.1 0.1 0.1 0.1 0.1
terminal 0.1 0.1 0 0.1 0 0.2 0.3 0.4
clade 0.1 0.1 0.1 0 0 0 0.1 0.2
sg−branch 0 0 0.1 0 0.1 0 0 0
0.05 0.1 0.2 0.5 2 5 10 20
Shift magnitude
BayesTraits
sg−branch clade terminal 0.2 0.2 0.2 0.1 0 0.1 0 0.1
clade terminal 0.2 0.2 0.2 0.1 0.1 0 0 0
sg−branch terminal 0.1 0.1 0.1 0.1 0 0 0.1 0.1
sg−branch clade 0.1 0.2 0.1 0.1 0.1 0 0 0
terminal 0.2 0.1 0.1 0.1 0.1 0.1 0.1 0.1
clade 0.1 0.1 0.1 0 0 0 0 0
sg−branch 0 0.1 0.1 0 0 0 0.1 0
0.05 0.1 0.2 0.5 2 5 10 20
Shift magnitude
BAMM
sg−branch clade terminal 0 0 0 0 0 0.1 0.1 0.1
clade terminal 0 0 0 0 0 0 0.1 0.1
sg−branch terminal 0 0 0 0 0 0.1 0.1 0.1
sg−branch clade 0 0 0 0 0 0 0 0
terminal 0 0 0 0 0 0.1 0.2 0.2
clade 0 0 0 0 0 0 0 0
sg−branch 0 0 0 0 0 0 0 0
0.05 0.1 0.2 0.5 2 5 10 20
Shift magnitude
BAMM−flip
sg−branch clade terminal 0.1 0.1 0.1 0 0 0.1 0 0.1
clade terminal 0.1 0 0 0.1 0 0.1 0.1 0
sg−branch terminal 0 0 0 0 0 0.1 0.1 0.1
sg−branch clade 0 0.1 0 0 0.1 0 0 0
terminal 0.1 0 0 0.1 0 0.1 0.1 0.2
clade 0.1 0.1 0.1 0 0 0 0 0
sg−branch 0 0 0.1 0 0 0 0 0
0.05 0.1 0.2 0.5 2 5 10 20
Shift magnitude
1
0.5
0

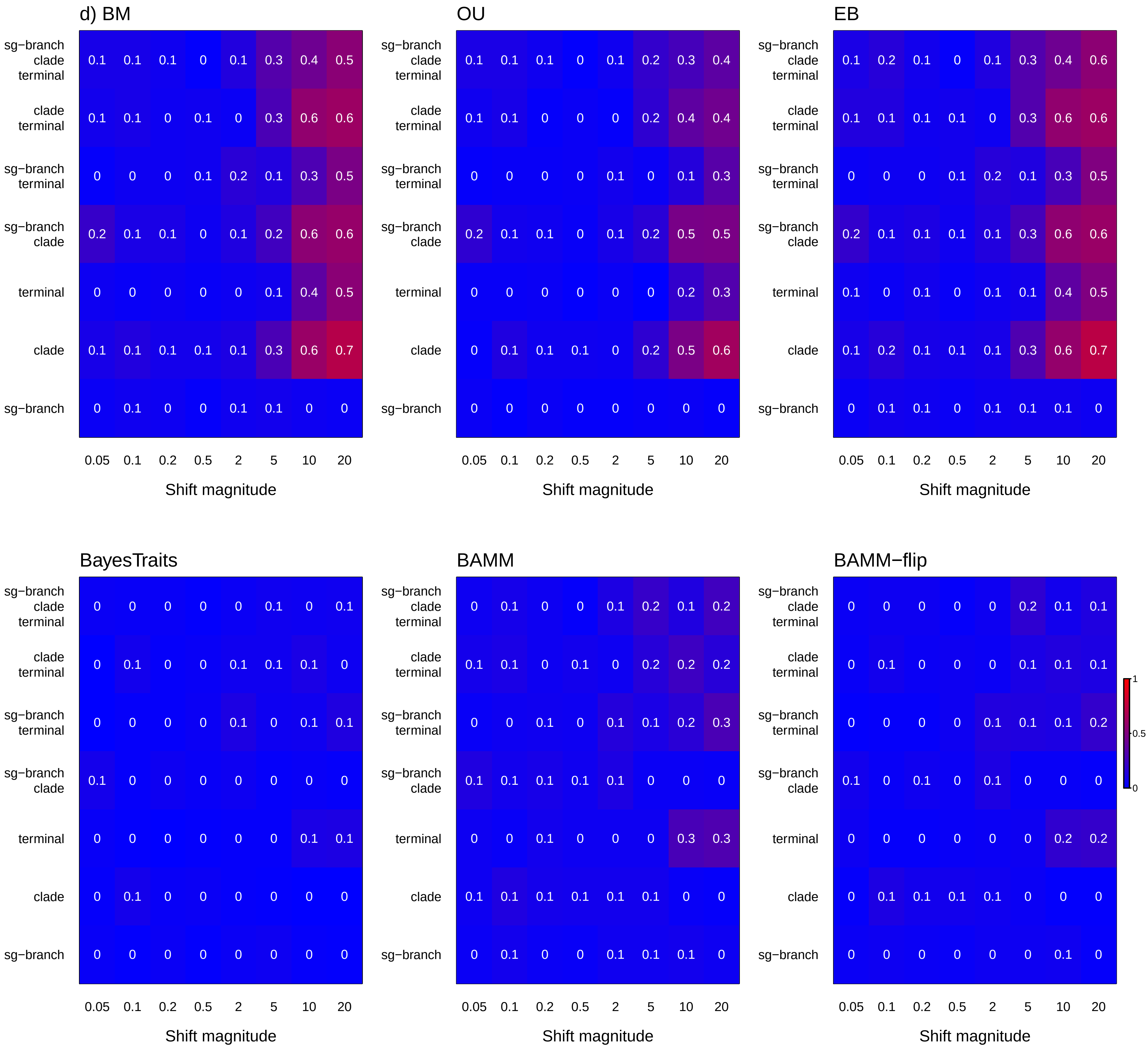
d) BM
sg−branch clade terminal
0.1 0.1 0.1 0 0.1 0.3 0.4 0.5
clade terminal
0.1 0.1 0 0.1 0 0.3 0.6 0.6
sg−branch terminal
0 0 0 0.1 0.2 0.1 0.3 0.5
sg−branch clade
0.2 0.1 0.1 0 0.1 0.2 0.6 0.6
terminal
0 0 0 0 0 0.1 0.4 0.5
clade
0.1 0.1 0.1 0.1 0.1 0.3 0.6 0.7
sg−branch
0 0.1 0 0 0.1 0.1 0 0
0.05 0.1 0.2 0.5 2 5 10 20
Shift magnitude
OU
sg−branch clade terminal
0.1 0.1 0.1 0 0.1 0.2 0.3 0.4
clade terminal
0.1 0.1 0 0 0 0.2 0.4 0.4
sg−branch terminal
0 0 0 0 0.1 0 0.1 0.3
sg−branch clade
0.2 0.1 0.1 0 0.1 0.2 0.5 0.5
terminal
0 0 0 0 0 0 0.2 0.3
clade
0 0.1 0.1 0.1 0 0.2 0.5 0.6
sg−branch
0 0 0 0 0 0 0 0
0.05 0.1 0.2 0.5 2 5 10 20
Shift magnitude
EB
sg−branch clade terminal
0.1 0.2 0.1 0 0.1 0.3 0.4 0.6
clade terminal
0.1 0.1 0.1 0.1 0 0.3 0.6 0.6
sg−branch terminal
0 0 0 0.1 0.2 0.1 0.3 0.5
sg−branch clade
0.2 0.1 0.1 0.1 0.1 0.3 0.6 0.6
terminal
0.1 0 0.1 0 0.1 0.1 0.4 0.5
clade
0.1 0.2 0.1 0.1 0.1 0.3 0.6 0.7
sg−branch
0 0.1 0.1 0 0.1 0.1 0.1 0
0.05 0.1 0.2 0.5 2 5 10 20
Shift magnitude
BayesTraits
sg−branch clade terminal
0 0 0 0 0 0.1 0 0.1
clade terminal
0 0.1 0 0 0.1 0.1 0.1 0
sg−branch terminal
0 0 0 0 0.1 0 0.1 0.1
sg−branch clade
0.1 0 0 0 0 0 0 0
terminal
0 0 0 0 0 0 0.1 0.1
clade
0 0.1 0 0 0 0 0 0
sg−branch
0 0 0 0 0 0 0 0
0.05 0.1 0.2 0.5 2 5 10 20
Shift magnitude
BAMM
sg−branch clade terminal
0 0.1 0 0 0.1 0.2 0.1 0.2
clade terminal
0.1 0.1 0 0.1 0 0.2 0.2 0.2
sg−branch terminal
0 0 0.1 0 0.1 0.1 0.2 0.3
sg−branch clade
0.1 0.1 0.1 0.1 0.1 0 0 0
terminal
0 0 0.1 0 0 0 0.3 0.3
clade
0.1 0.1 0.1 0.1 0.1 0.1 0 0
sg−branch
0 0.1 0 0 0.1 0.1 0.1 0
0.05 0.1 0.2 0.5 2 5 10 20
Shift magnitude
BAMM−flip
sg−branch clade terminal
0 0 0 0 0 0.2 0.1 0.1
clade terminal
0 0.1 0 0 0 0.1 0.1 0.1
sg−branch terminal
0 0 0 0 0.1 0.1 0.1 0.2
sg−branch clade
0.1 0 0.1 0 0.1 0 0 0
terminal
0 0 0 0 0 0 0.2 0.2
clade
0 0.1 0.1 0.1 0.1 0 0 0
sg−branch
0 0 0 0 0 0 0.1 0
0.05 0.1 0.2 0.5 2 5 10 20
Shift magnitude
1
0.5
0

Figure S5. Frequency of positive (red spectrum) and negative (blue spectrum) significant differences ($P < 0.05$) between test statistics across key ARBUTUS diagnostics: (a) C.VAR, (b) S.HGT, (c) S.VAR, and (d) S.ASR. Results across simulated heterogeneity scenarios: internal branch shift, clade even, isolated terminal changes, and combinations. The magnitude of rate shifts appears on the x-axis. Black signals 0 inadequacy levels for a particular rate-shifts scenario. The following models are considered: BM, OU, EB, BayesTraits, and two BAMM alternatives: constrained to time-varying evolution (BAMM) and allowed to flip between time-varying and time-constant trait-change (BAMM-flip). Frequency levels for each specific model are also highlighted by the blue-red colour scheme.

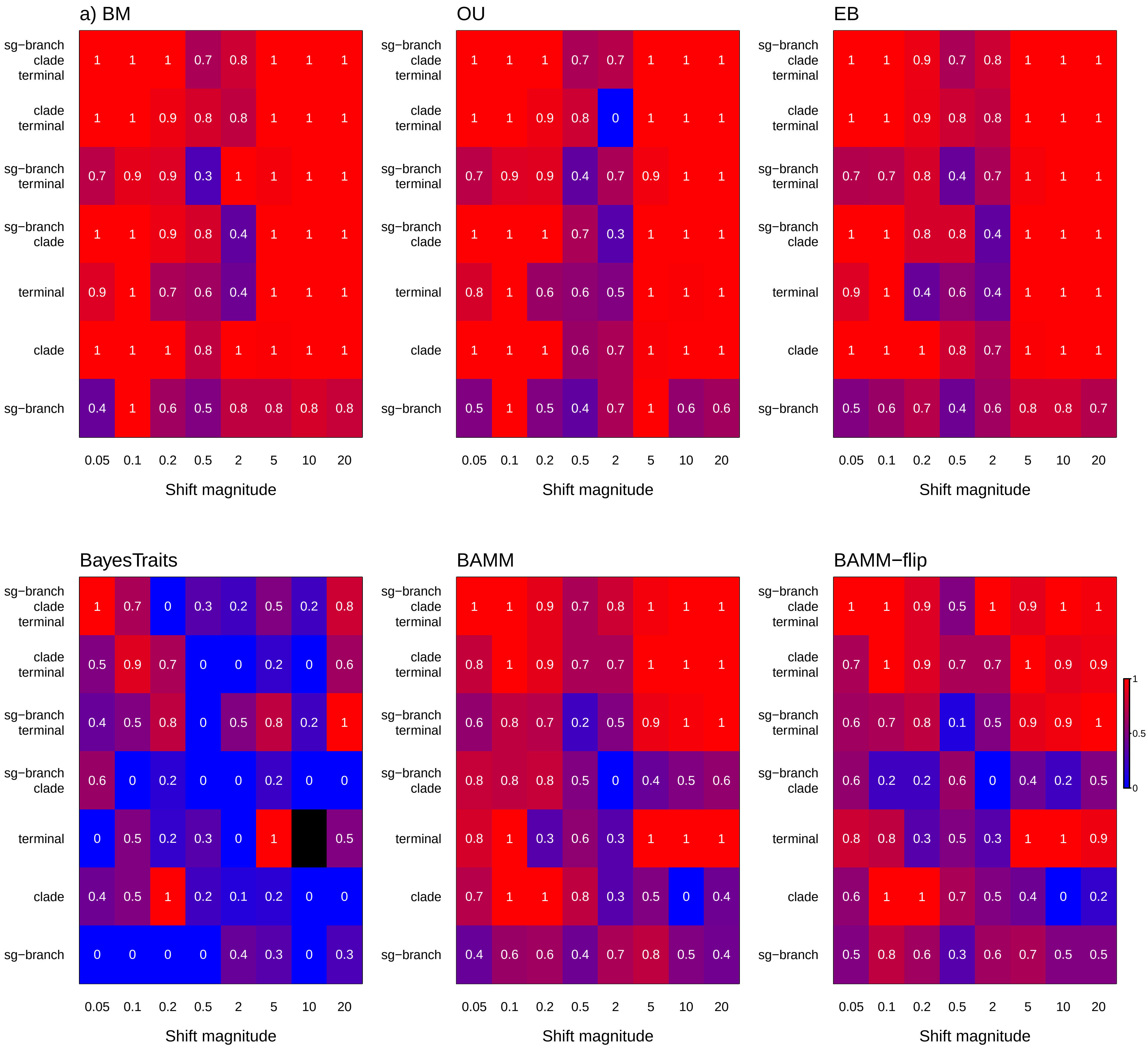
a) BM
sg−branch clade terminal
clade terminal
sg−branch terminal
sg−branch clade
terminal
clade
sg−branch
1 1 1 0.7 0.8 1 1 1
1 1 0.9 0.8 0.8 1 1 1
0.7 0.9 0.9 0.3 1 1 1 1
1 1 0.9 0.8 0.4 1 1 1
0.9 1 0.7 0.6 0.4 1 1 1
1 1 1 0.8 1 1 1 1
0.4 1 0.6 0.5 0.8 0.8 0.8 0.8
0.05 0.1 0.2 0.5 2 5 10 20
Shift magnitude
OU
sg−branch clade terminal
clade terminal
sg−branch terminal
sg−branch clade
terminal
clade
sg−branch
1 1 1 0.7 0.7 1 1 1
1 1 0.9 0.8 0 1 1 1
0.7 0.9 0.9 0.4 0.7 0.9 1 1
1 1 1 0.7 0.3 1 1 1
0.8 1 0.6 0.6 0.5 1 1 1
1 1 1 0.6 0.7 1 1 1
0.5 1 0.5 0.4 0.7 1 0.6 0.6
0.05 0.1 0.2 0.5 2 5 10 20
Shift magnitude
EB
sg−branch clade terminal
clade terminal
sg−branch terminal
sg−branch clade
terminal
clade
sg−branch
1 1 0.9 0.7 0.8 1 1 1
1 1 0.9 0.8 0.8 1 1 1
0.7 0.7 0.8 0.4 0.7 1 1 1
1 1 0.8 0.8 0.4 1 1 1
0.9 1 0.4 0.6 0.4 1 1 1
1 1 1 0.8 0.7 1 1 1
0.5 0.6 0.7 0.4 0.6 0.8 0.8 0.7
0.05 0.1 0.2 0.5 2 5 10 20
Shift magnitude
BayesTraits
sg−branch clade terminal
clade terminal
sg−branch terminal
sg−branch clade
terminal
clade
sg−branch
1 0.7 0 0.3 0.2 0.5 0.2 0.8
0.5 0.9 0.7 0 0 0.2 0 0.6
0.4 0.5 0.8 0 0.5 0.8 0.2 1
0.6 0 0.2 0 0 0.2 0 0
0 0.5 0.2 0.3 0 1 0.5
0.4 0.5 1 0.2 0.1 0.2 0 0
0 0 0 0 0.4 0.3 0 0.3
0.05 0.1 0.2 0.5 2 5 10 20
Shift magnitude
BAMM
sg−branch clade terminal
clade terminal
sg−branch terminal
sg−branch clade
terminal
clade
sg−branch
1 1 0.9 0.7 0.8 1 1 1
0.8 1 0.9 0.7 0.7 1 1 1
0.6 0.8 0.7 0.2 0.5 0.9 1 1
0.8 0.8 0.8 0.5 0 0.4 0.5 0.6
0.8 1 0.3 0.6 0.3 1 1 1
0.7 1 1 0.8 0.3 0.5 0 0.4
0.4 0.6 0.6 0.4 0.7 0.8 0.5 0.4
0.05 0.1 0.2 0.5 2 5 10 20
Shift magnitude
BAMM−flip
sg−branch clade terminal
clade terminal
sg−branch terminal
sg−branch clade
terminal
clade
sg−branch
1 1 0.9 0.5 1 0.9 1 1
0.7 1 0.9 0.7 0.7 1 0.9 0.9
0.6 0.7 0.8 0.1 0.5 0.9 0.9 1
0.6 0.2 0.2 0.6 0 0.4 0.2 0.5
0.8 0.8 0.3 0.5 0.3 1 1 0.9
0.6 1 1 0.7 0.5 0.4 0 0.2
0.5 0.8 0.6 0.3 0.6 0.7 0.5 0.5
0.05 0.1 0.2 0.5 2 5 10 20
Shift magnitude
1
0.5
0

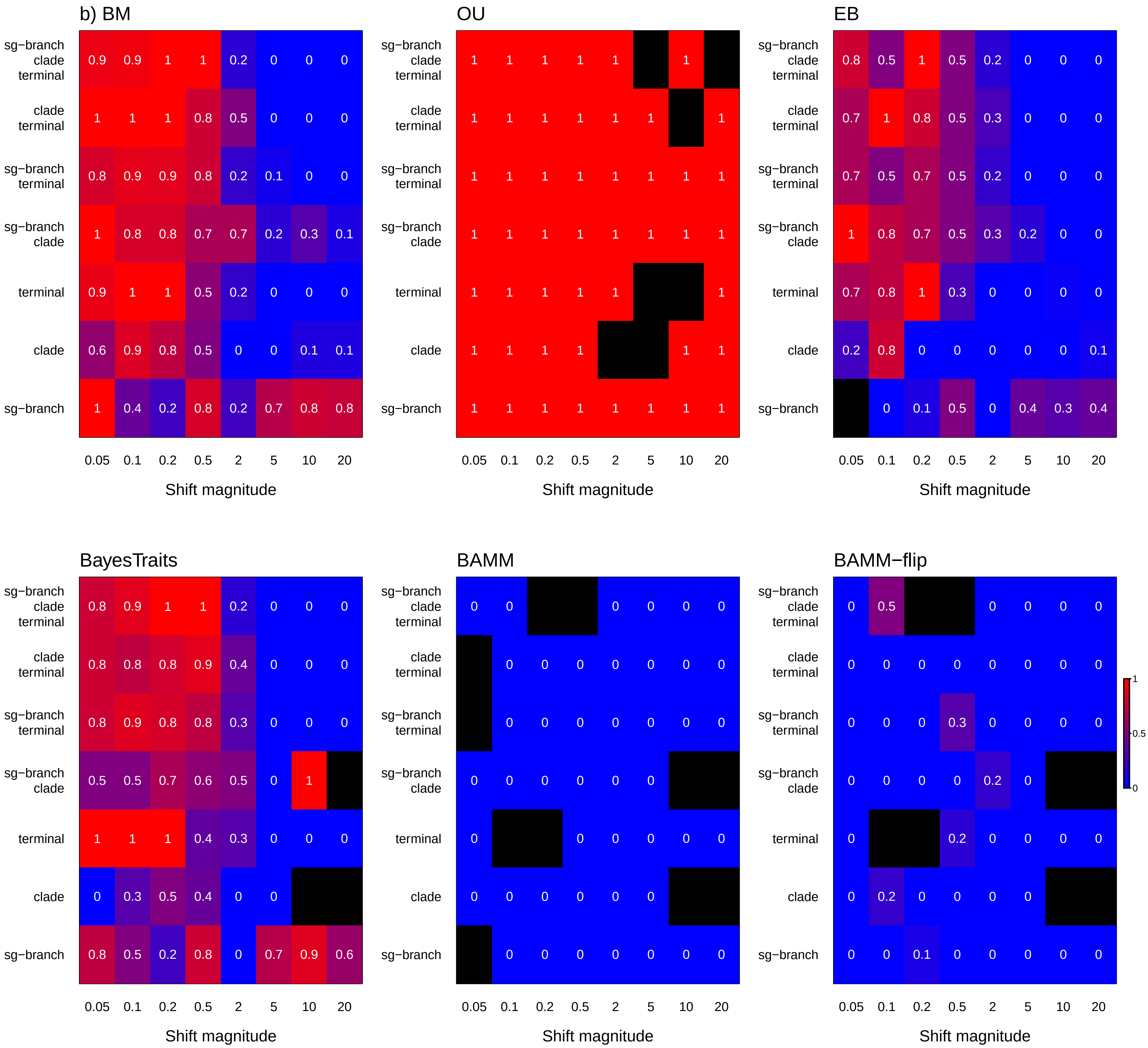
b) BM
sg−branch clade terminal
clade terminal
sg−branch terminal
sg−branch clade
terminal
clade
sg−branch
0.9 0.9 1 1 0.2 0 0 0
1 1 1 0.8 0.5 0 0 0
0.8 0.9 0.9 0.8 0.2 0.1 0 0
1 0.8 0.8 0.7 0.7 0.2 0.3 0.1
0.9 1 1 0.5 0.2 0 0 0
0.6 0.9 0.8 0.5 0 0 0.1 0.1
1 0.4 0.2 0.8 0.2 0.7 0.8 0.8
0.05 0.1 0.2 0.5 2 5 10 20
Shift magnitude
OU
1 1 1 1 1 1
1 1 1 1 1 1 1
1 1 1 1 1 1 1 1
1 1 1 1 1 1 1 1
1 1 1 1 1 1
1 1 1 1 1 1
1 1 1 1 1 1 1 1
0.05 0.1 0.2 0.5 2 5 10 20
Shift magnitude
EB
0.8 0.5 1 0.5 0.2 0 0 0
0.7 1 0.8 0.5 0.3 0 0 0
0.7 0.5 0.7 0.5 0.2 0 0 0
1 0.8 0.7 0.5 0.3 0.2 0 0
0.7 0.8 1 0.3 0 0 0 0
0.2 0.8 0 0 0 0 0 0.1
0 0.1 0.5 0 0.4 0.3 0.4
0.05 0.1 0.2 0.5 2 5 10 20
Shift magnitude
BayesTraits
0.8 0.9 1 1 0.2 0 0 0
0.8 0.8 0.8 0.9 0.4 0 0 0
0.8 0.9 0.8 0.8 0.3 0 0 0
0.5 0.5 0.7 0.6 0.5 0 1
1 1 1 0.4 0.3 0 0 0
0 0.3 0.5 0.4 0 0
0.8 0.5 0.2 0.8 0 0.7 0.9 0.6
0.05 0.1 0.2 0.5 2 5 10 20
Shift magnitude
BAMM
0 0 0 0 0 0
0 0 0 0 0 0 0
0 0 0 0 0 0 0
0 0 0 0 0 0
0 0 0 0 0 0
0 0 0 0 0 0
0 0 0 0 0 0 0
0.05 0.1 0.2 0.5 2 5 10 20
Shift magnitude
BAMM−flip
0 0.5 0 0 0 0
0 0 0 0 0 0 0 0
0 0 0 0.3 0 0 0 0
0 0 0 0 0.2 0
0 0.2 0 0 0 0
0 0.2 0 0 0 0
0 0 0.1 0 0 0 0 0
0.05 0.1 0.2 0.5 2 5 10 20
Shift magnitude
1
0.5
0

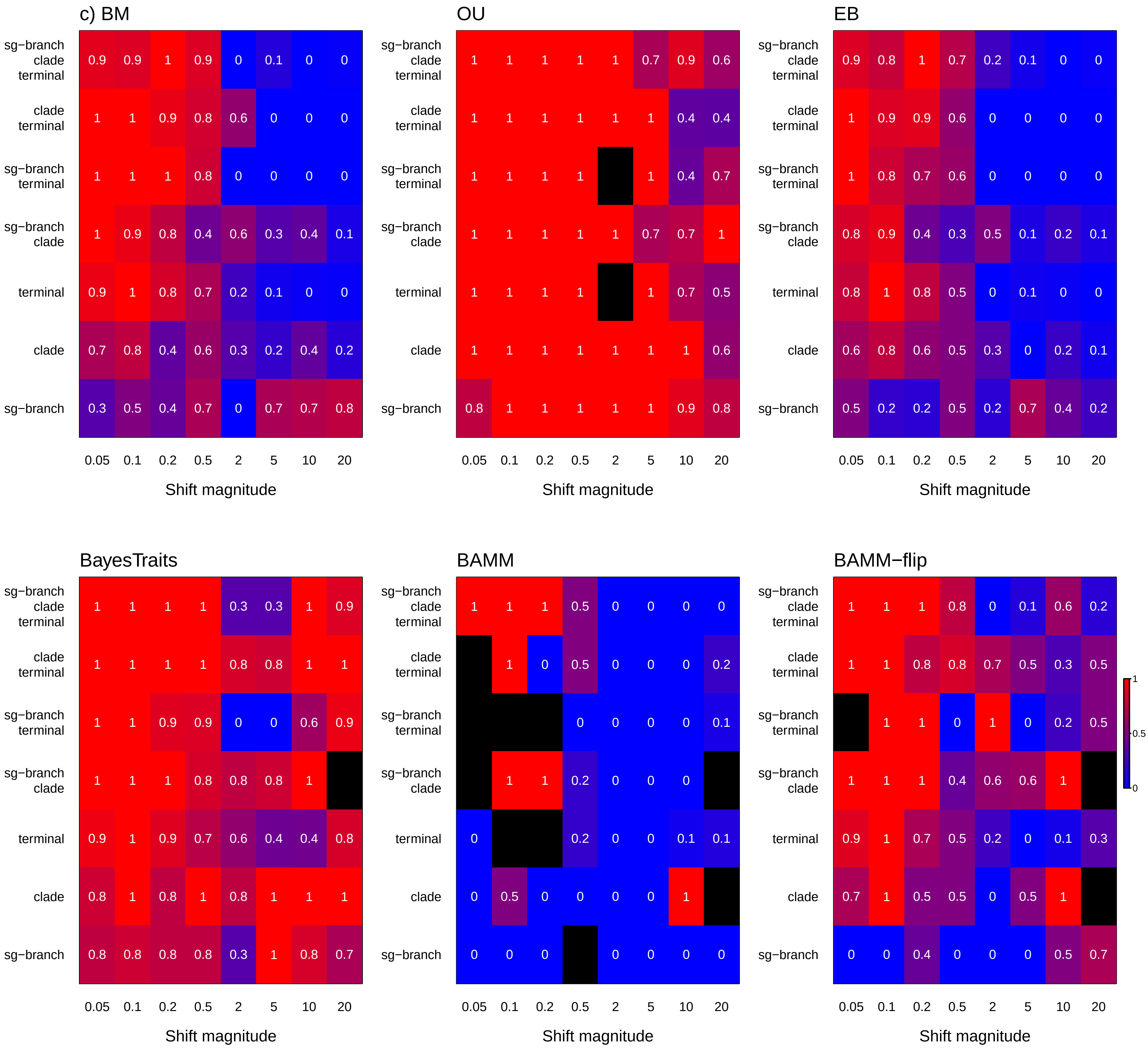

c) BM
OU
EB
BayesTraits
BAMM
BAMM−flip
sg−branch clade terminal
clade terminal
sg−branch terminal
sg−branch clade
terminal
clade
sg−branch
0.05 0.1 0.2 0.5 2 5 10 20
Shift magnitude

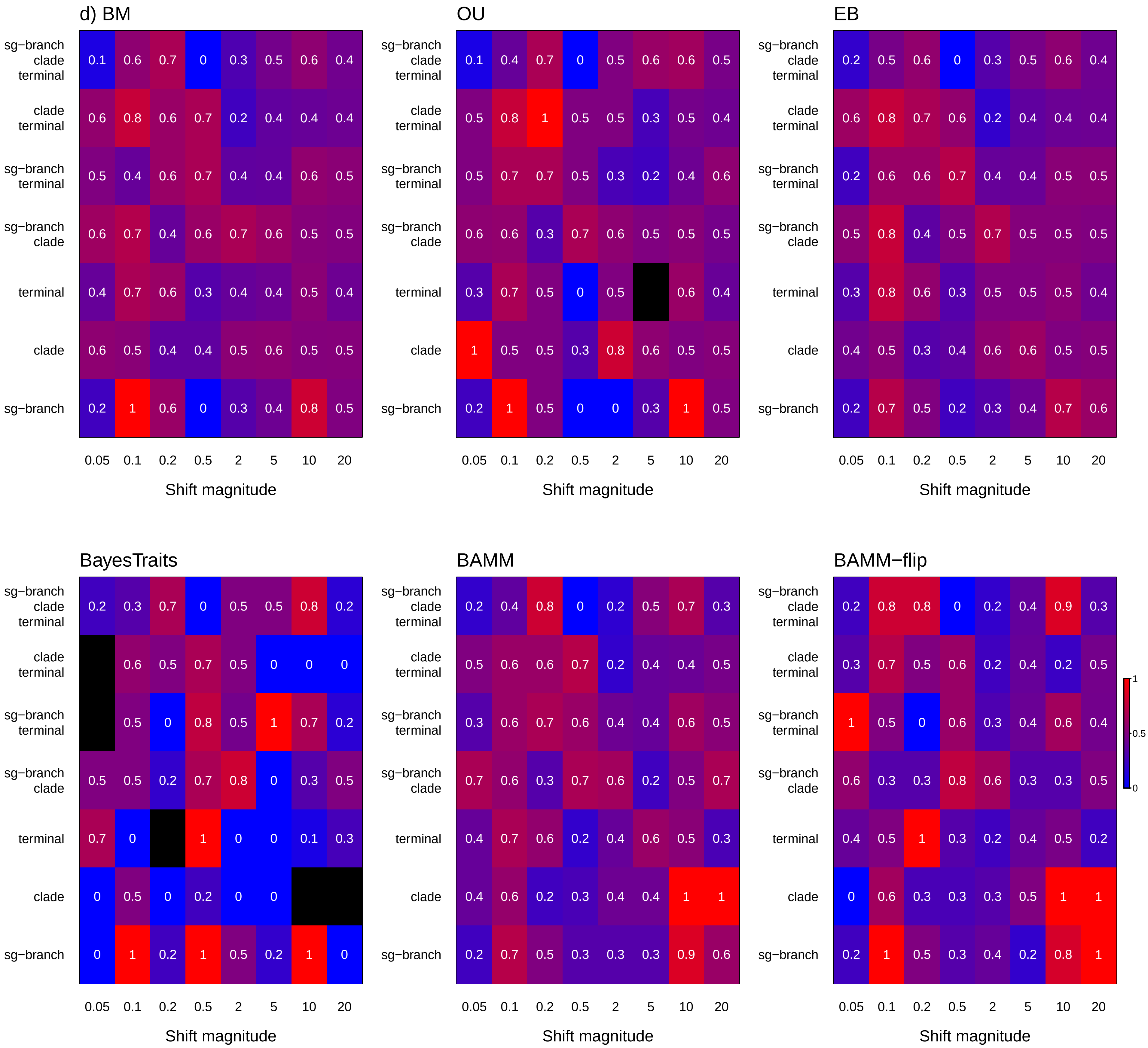
d) BM
OU
EB
BayesTraits
BAMM
BAMM−flip
sg−branch clade terminal
clade terminal
sg−branch terminal
sg−branch clade
terminal
clade
sg−branch
0.05
0.1
0.2
0.5
2
5
10
20
Shift magnitude

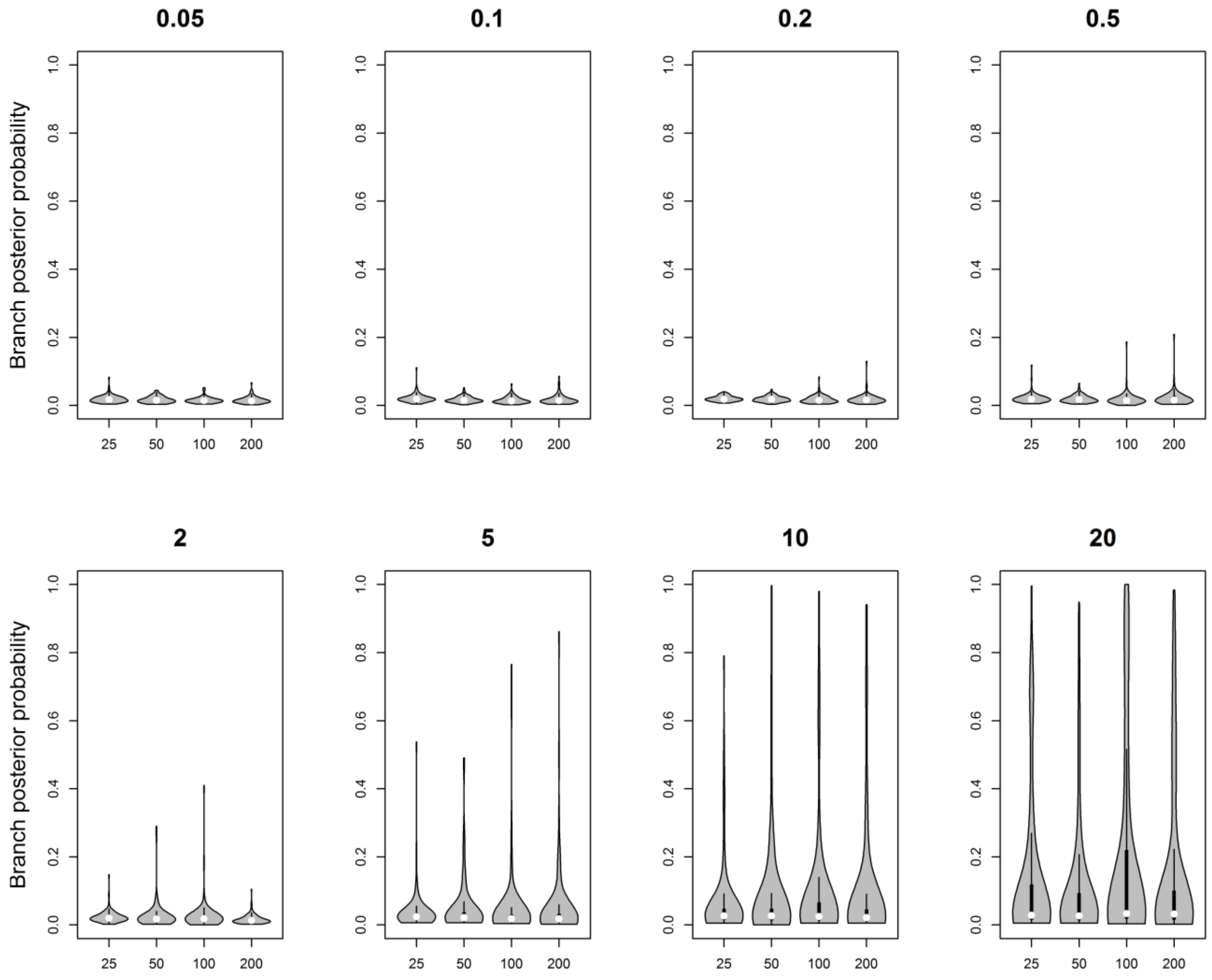

Figure S6. Posterior probabilities for a shift in the rate of evolution on the branch with a simulated rate-change compared between trees of various sizes (x-axis). Each panel corresponds to a different shift magnitude.

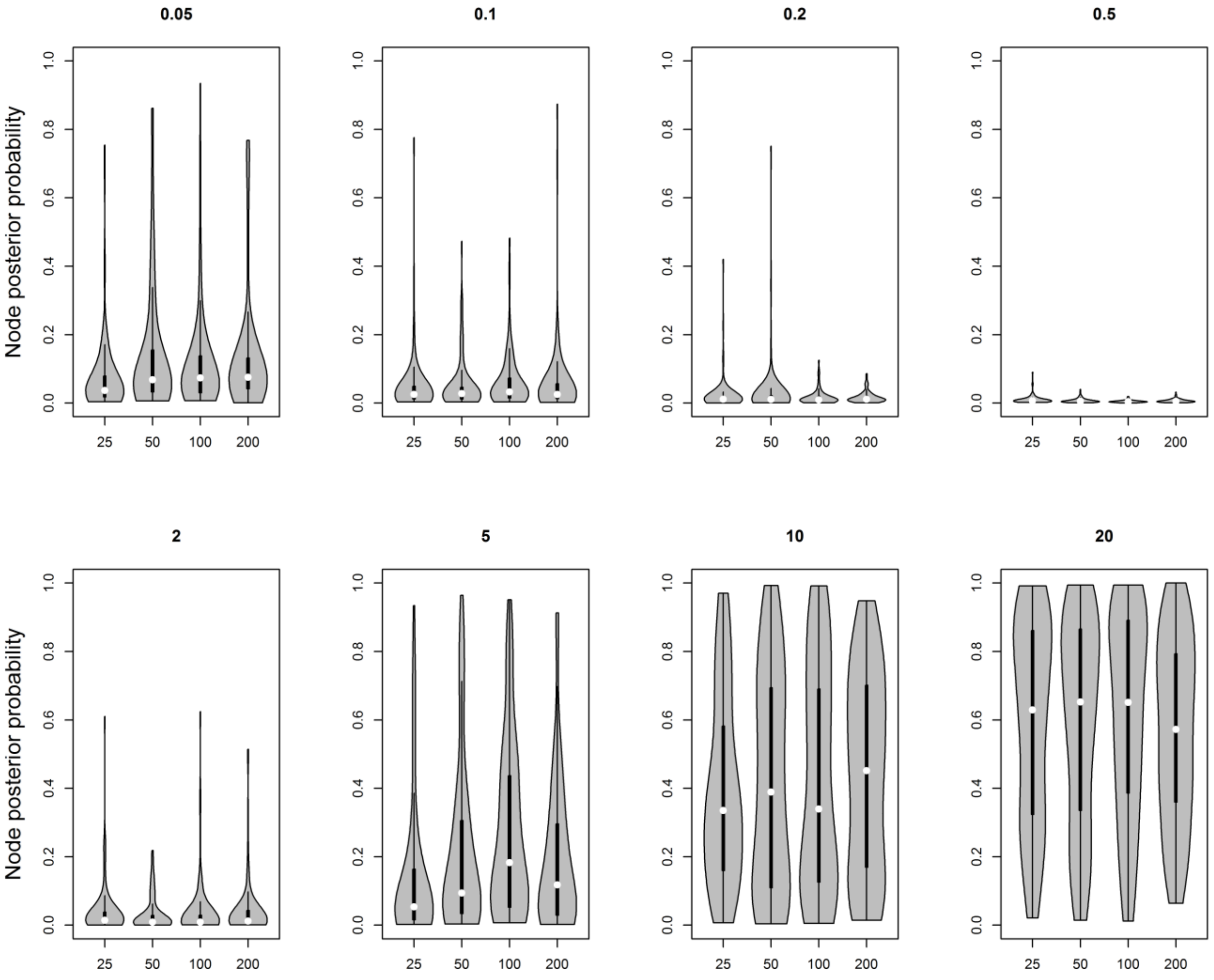

Figure S7. Posterior probabilities for a shift in the rate of evolution in a whole clade (node posterior probabilities) with a simulated rate-change compared between trees of various sizes (x-axis). Each panel corresponds to a different shift magnitude.

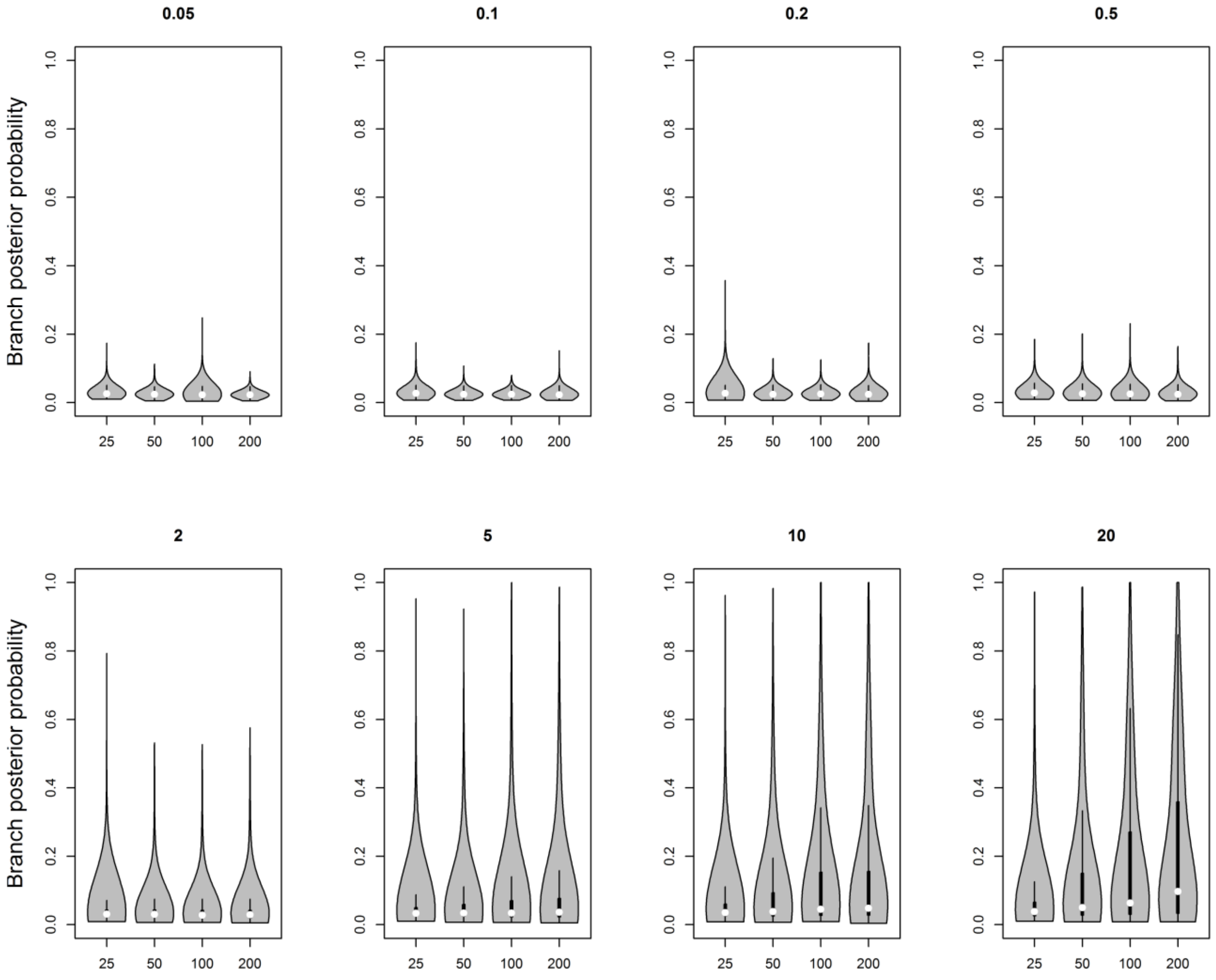

Figure S8. Posterior probabilities for shifts in the rate of evolution on branches with simulated rate-changes compared between trees of various sizes (x-axis). Each panel corresponds to a different shift magnitude.

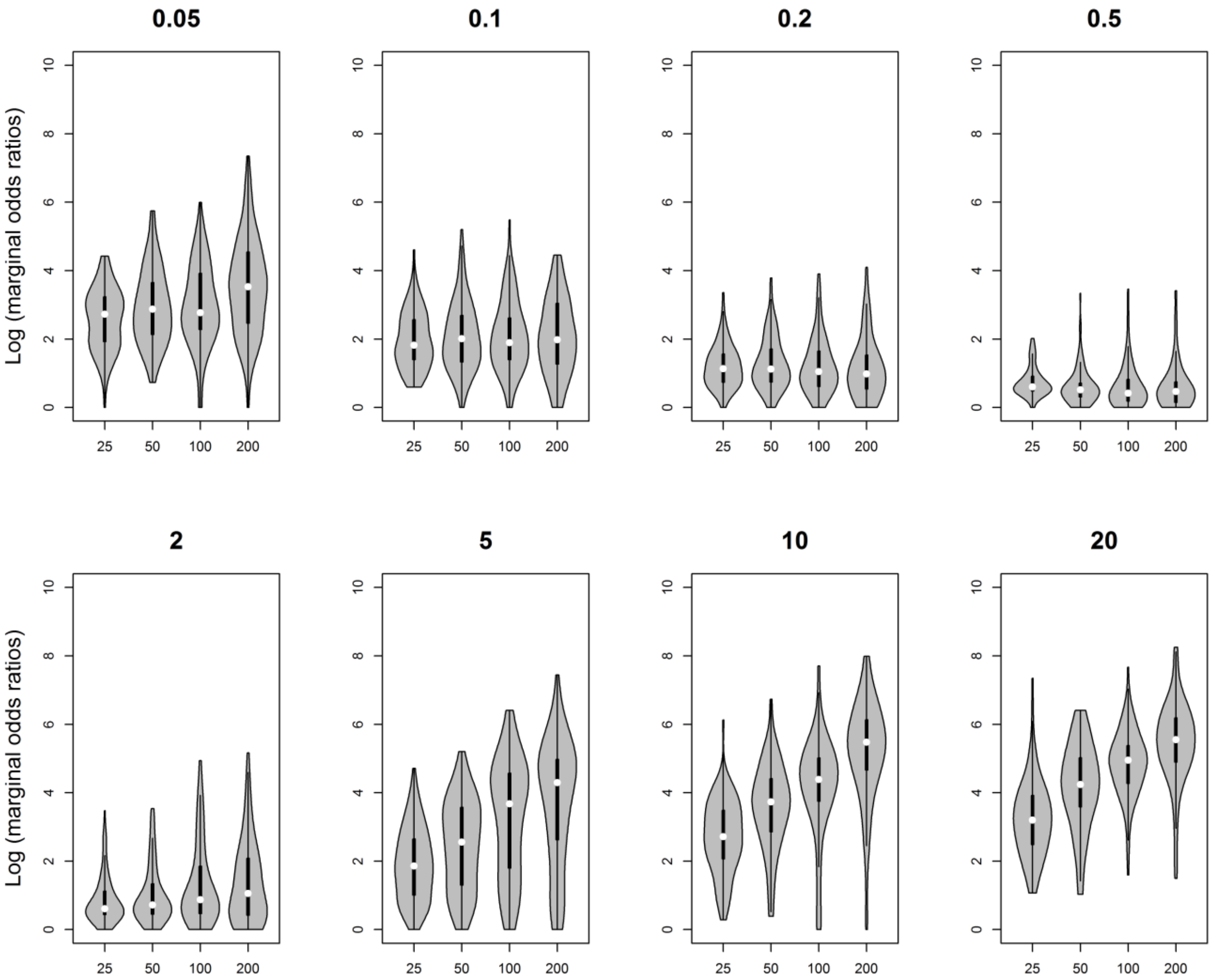

Figure S9. Log-marginal odds ratios (i.e. relative odds of a clade-shift) for a shift in the rate of evolution in a whole clade compared between trees of various sizes (x-axis). Each panel corresponds to a different shift magnitude.

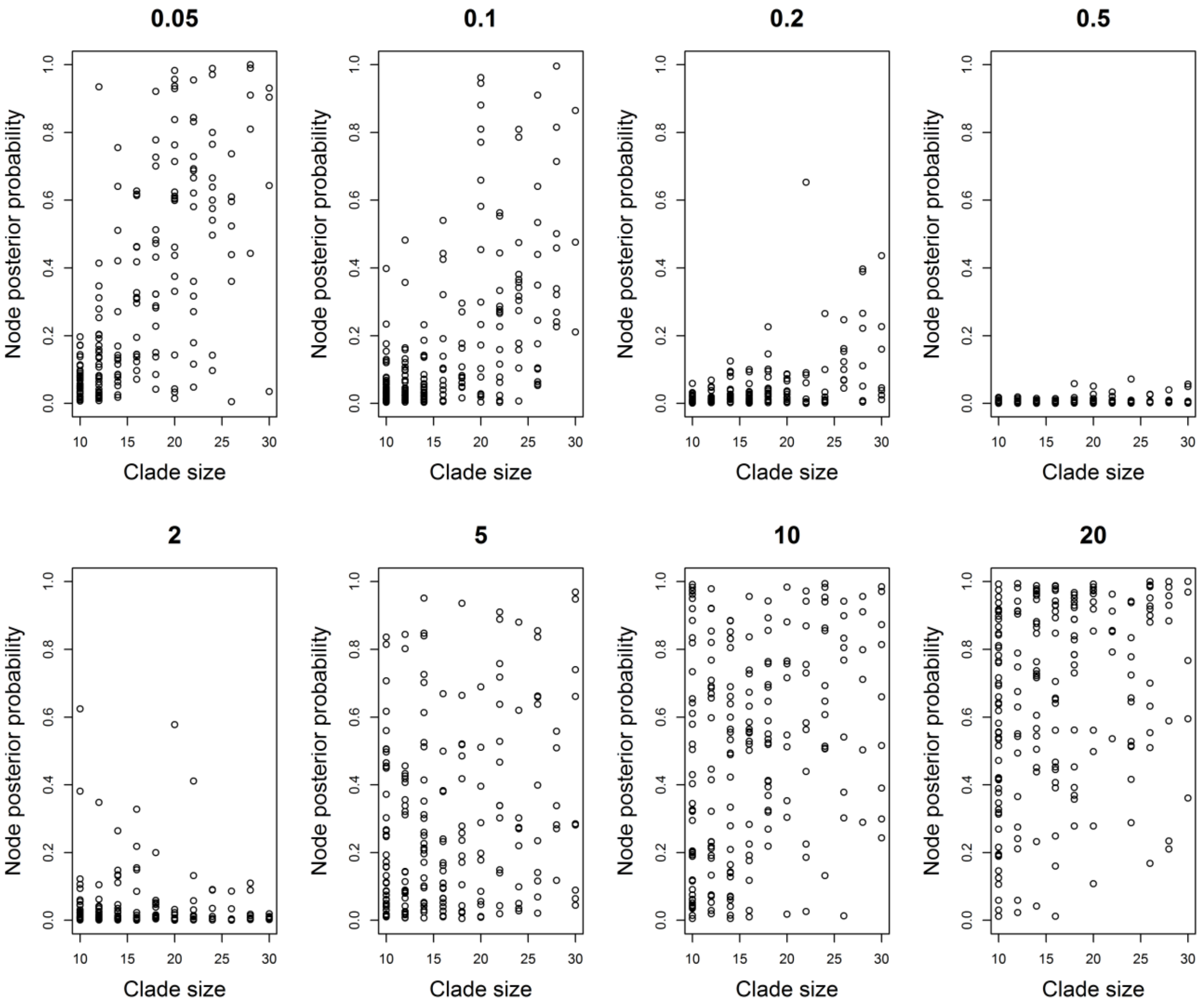

Figure S10. Posterior probabilities for a shift in the rate of evolution in a whole clade (node posterior probabilities) with a simulated rate-change for different sizes of the heterogeneous clade (x-axis). Each panel corresponds to a different shift magnitude.

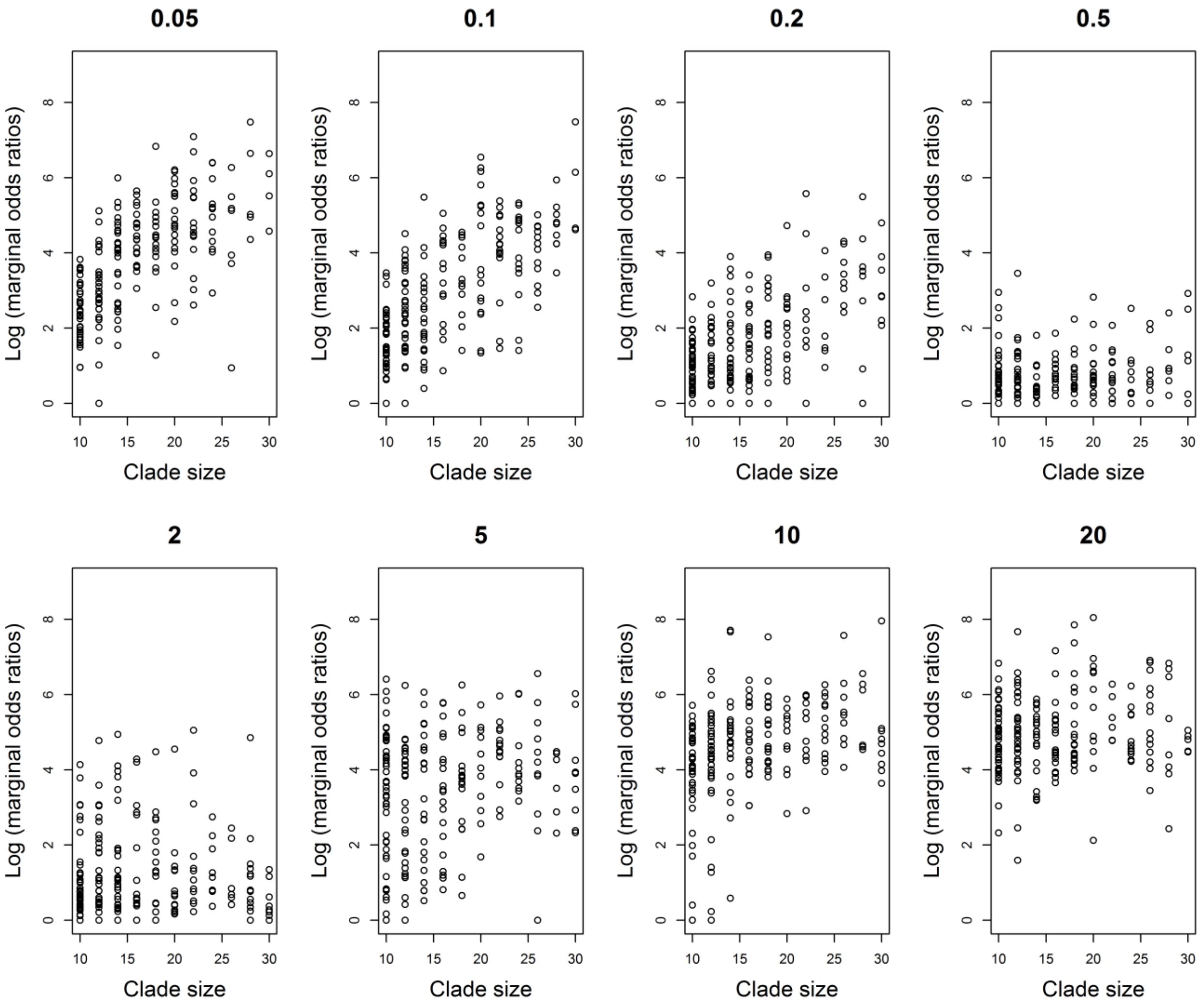

Figure S11. Log-marginal odds ratios (i.e. relative odds of a clade-shift) for a shift in the rate of evolution in a whole clade with a simulated rate-change for different sizes of the heterogeneous clade (x-axis). Each panel corresponds to a different shift magnitude.

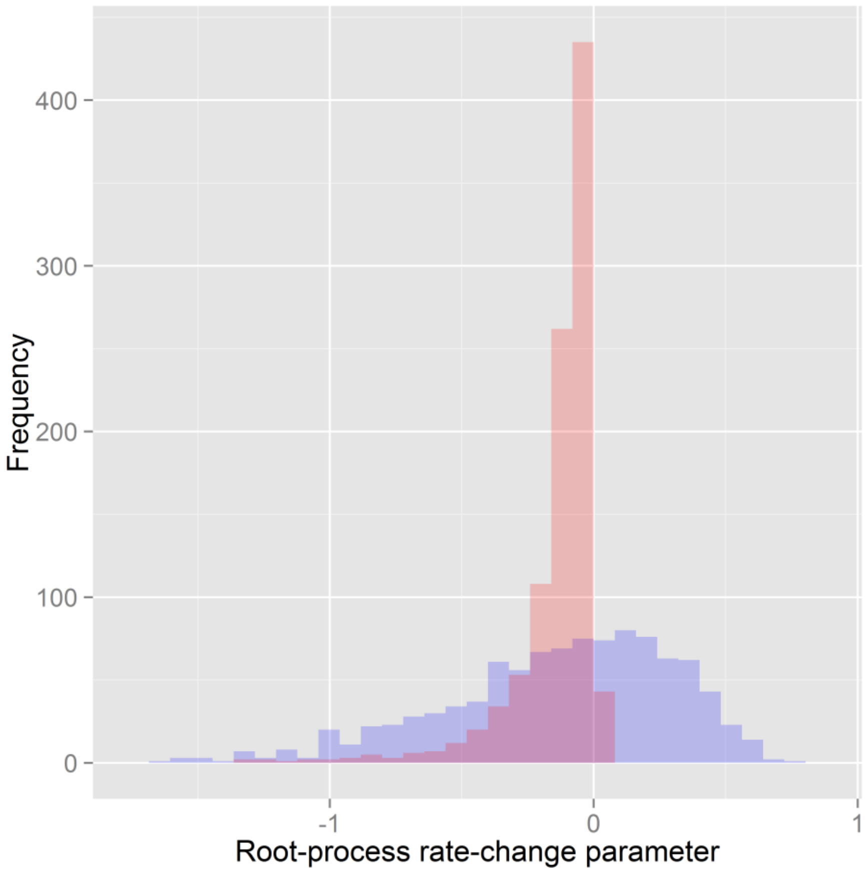

Figure S12. Frequency of rate-change parameters for the root process across constant-rate trees, estimated using BAMM with (red) and without (blue) a time-flip proposal. The expected mean for no rate-changes at the root is at x = 0.

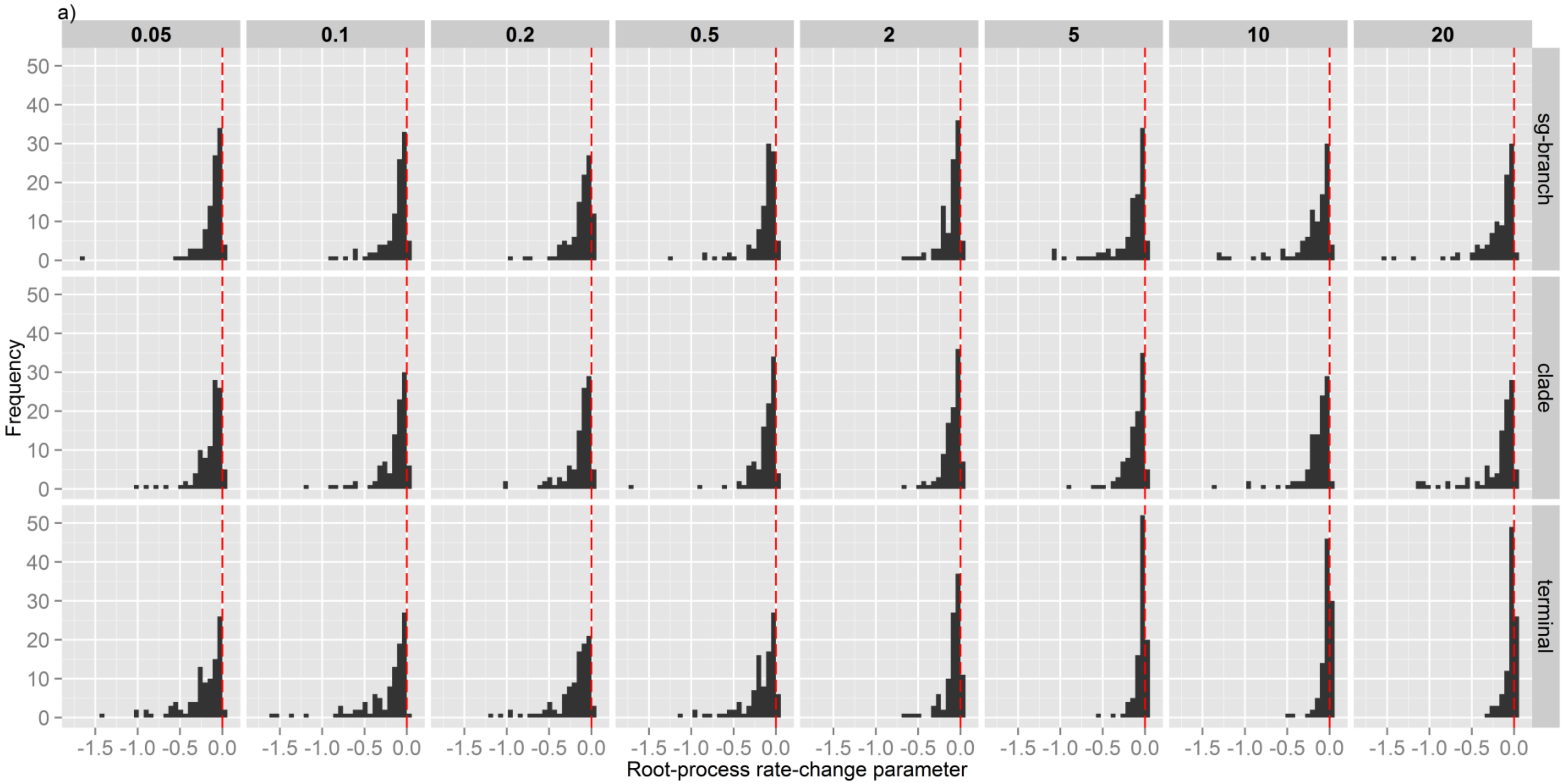

Figure S13a). Frequency of rate-change parameters for the root process across simulated heterogeneity scenarios: single branch shift, clade event, and changes on isolated terminal branches. The magnitudes of shifts in each scenario are recorded on the vertical panel. The expected mean for no rate-changes at the root is the 0 line (dashed red). Rate change parameters are estimated by a BAMM model with a time-flip proposal.

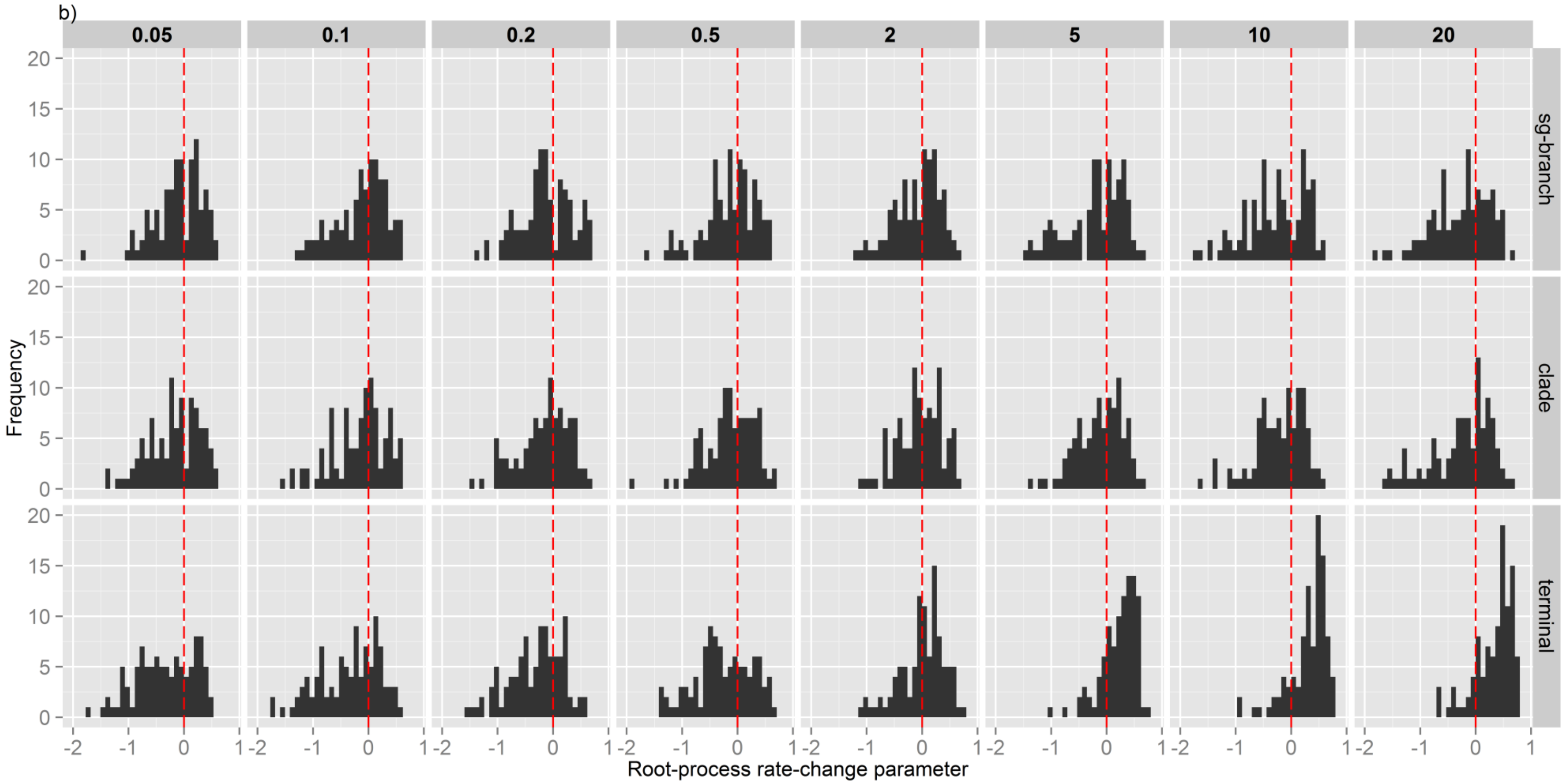

Figure S13b). Frequency of rate-change parameters for the root process across simulated heterogeneity scenarios: single branch shift, clade event, and changes on isolated terminal branches. The magnitudes of shifts in each scenario are recorded on the vertical panel. The expected mean for no rate-changes at the root is the 0 line (dashed red). Rate change parameters are estimated by a BAMM model constrained to time-varying processes only.

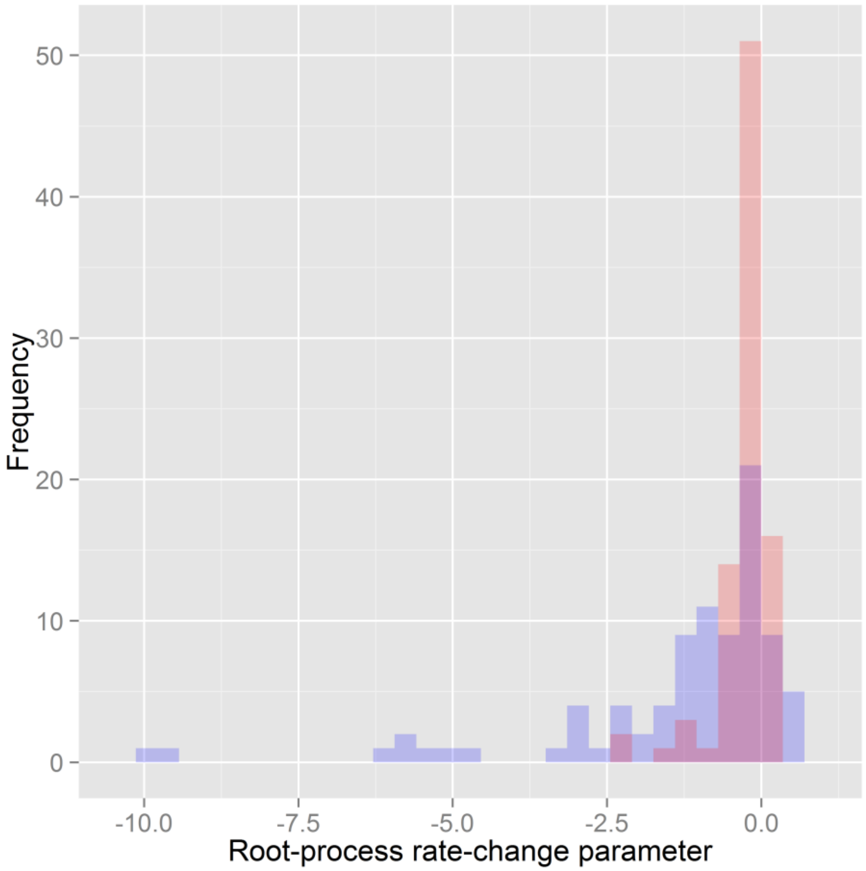

Figure S14. Frequency of rate-change parameters for the root process across empirical datasets, estimated using BAMM with (red) and without (blue) a time-flip proposal. The expected mean for no rate-changes is at x = 0.

Empirical Data – Rate heterogeneity and general absolute adequacy

The median branch lengths across all scaled-trees in the posterior were used to build the median scaled tree for each group of species. Clades were catalogued as heterogeneous if they had per-branch rate changes more substantial than x2 or x0.5 (i.e. the proportion between the rate-scaled branch length and the identical branch length in the input phylogeny was bigger than x2 or smaller than 1/2). This crude rate-variation criterion has used to investigate whether single-process models show higher levels of absolute inadequacy in relation to higher levels of rate heterogeneity across empirical datasets (Fig. S15).

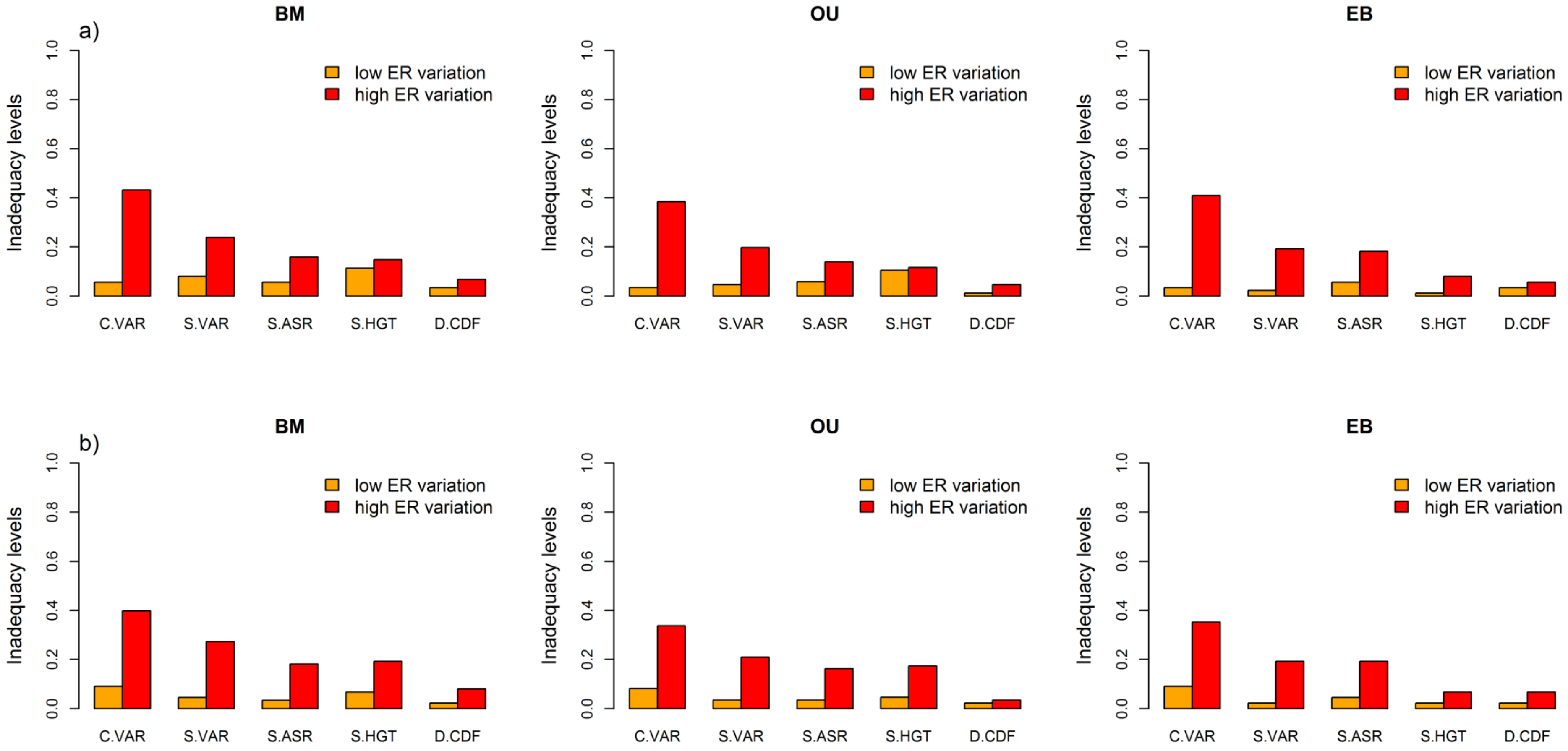

Figure S15. Model inadequacy levels (measured as frequency of inadequate trees and associated trait data) for single-process models in clades with low and high ER heterogeneity, quantified using the median scaled trees provided by (a) BAMM-flip and (b) BayesTraits.

Figure S16. Model inadequacy levels (quantified as the frequency of trees and associated trait data where the focal model was inadequate) across simulated rate-heterogeneity scenarios: internal branch shift; clade event; rate-changes on isolated, terminal branches; rate-burst followed by gradual decreases within a clade, and constant rate-deceleration process from root to tips. Results on the mean scaled trees from the output of BayesTraits. Inadequacy levels measure model ability to account for (a) total rate variation, and further, variation related to (b) time, (c) branch lengths, and (d) ancestral states. Inadequacy is quantified separately for rate increases (inc, up-pointing triangles) and decreases (dec, down-pointing triangles), and the exact magnitude of each shift is highlighted by the white-black colour scheme. For scenarios involving gradual rate-changes, the natural logarithm of the shift magnitude represents the constant rate-change parameter.

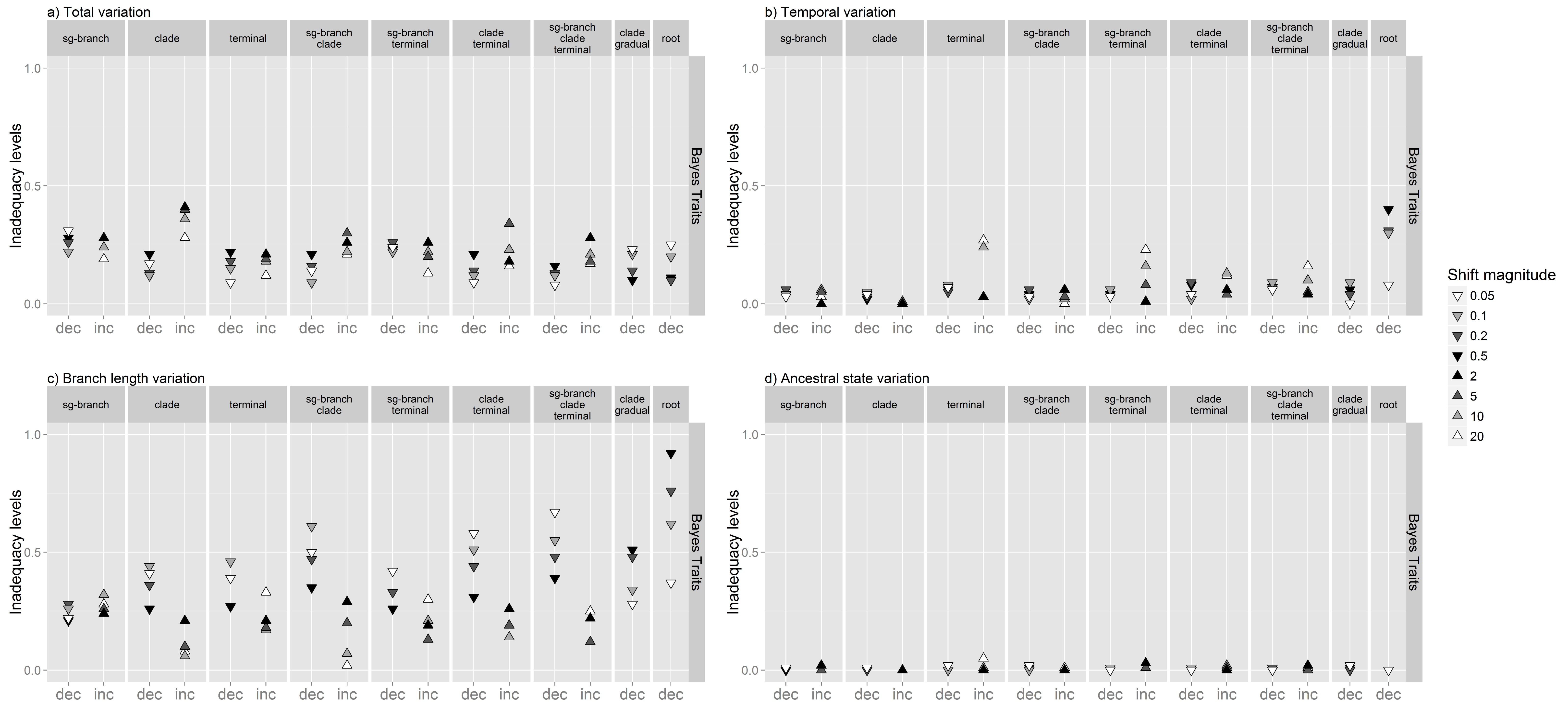
a) Total variation
b) Temporal variation
c) Branch length variation
d) Ancestral state variation
sg-branch
clade
terminal
sg-branch clade
sg-branch terminal
clade terminal
sg-branch clade terminal
clade gradual
root
Bayes Traits
Inadequacy levels
0.0
0.5
1.0
dec
inc
Shift magnitude
0.05
0.1
0.2
0.5
2
5
10
20

Table S5. Bayes factor (BF) evidence for alternative models with various numbers of rate-shifts given by BAMM-flip relative to the model with the lowest number of supported shifts (here, always 0). Where there was strong support for at least one rate regime shift (i.e. BF for one or more shifts relative to the null model > 20) the number of shifts with greatest support is reported (Most supported #shifts). Results on the empirical datasets.

Group name	Number of rate-shifts														Most supported #shifts
	0	1	2	3	4	5	6	7	8	9	10	11	12	13	
Australasian Babblers, Logrunners	1.00	3.20	10.80	23.12	36.71	48.78	45.45	40.78	44.18	23.79	9.06	9.06	9.06	18.12	5.00
Owlet-Nightjars	1.00	2.57	6.78	12.86	20.82	23.03	26.78	19.98	18.74	11.31	2.83	5.66	NA	NA	6.00
Ducks I, Geese I, Other Waterfowl, Screamers I	1.00	1.67	2.01	2.03	1.69	1.01	0.64	0.59	0.23	NA	NA	NA	NA	NA	0.00
Waxwings, Allies	1.00	8.29	28.77	66.51	116.31	157.54	190.77	198.56	175.59	141.13	65.64	78.77	52.51	52.51	7.00
Cormorants, Anhingas, Gannets, Frigatebirds	1.00	2.31	4.16	5.44	6.17	5.08	3.16	2.20	1.34	0.38	NA	NA	NA	NA	0.00
Accentors, Olive Warbler	1.00	36.08	62.80	60.92	43.45	26.09	14.77	4.92	NA	NA	NA	NA	NA	NA	2.00
Gnateaters, Allies	1.00	3.64	17.90	35.67	49.90	54.67	50.67	44.19	25.90	12.19	9.14	6.10	NA	NA	5.00
Cranes, Trumpeters, Limpkin	1.00	1.57	2.06	1.69	1.08	0.88	0.36	0.11	0.11	NA	NA	NA	NA	NA	0.00
Berrypeckers, Satinbirds, Allies	1.00	3.95	12.28	30.55	57.98	93.25	112.37	122.93	105.61	81.11	94.63	20.28	54.07	NA	7.00
Grosbeaks, Saltators, Allies	1.00	2.45	5.30	8.52	10.12	9.37	6.19	2.91	3.88	3.88	0.97	3.88	NA	NA	0.00
Whipbirds, Quail-Thrushes,	1.00	1.93	3.84	5.18	6.28	5.40	4.52	2.89	2.17	1.09	0.72	2.89	NA	NA	0.00

Allies															
African Warblers	1.00	2.94	8.09	16.93	27.10	39.05	44.50	37.62	39.46	26.61	20.19	25.69	7.34	NA	6.00
Penguins	1.00	1.88	2.85	3.30	2.99	1.80	1.21	0.57	0.57	0.28	NA	NA	NA	NA	0.00
Australian Treecreepers, Bowerbirds	1.00	2.57	6.47	10.13	14.75	14.92	13.45	9.99	6.17	5.29	7.05	4.70	4.70	NA	0.00
Storks	1.00	1.73	2.86	3.47	3.44	2.62	1.46	0.78	0.86	0.29	0.57	NA	NA	NA	0.00
Grebes	1.00	2.72	6.67	10.67	13.44	16.70	13.64	11.61	7.24	4.22	4.82	2.41	NA	NA	0.00
Parrots I	1.00	1.66	2.02	1.57	1.40	0.68	0.28	0.06	0.11	NA	NA	NA	NA	NA	0.00
Megapodes	1.00	2.36	5.28	7.96	10.36	9.70	9.61	6.29	3.63	3.38	0.97	NA	NA	NA	0.00
Turacos	1.00	22.30	70.48	153.01	221.57	255.59	224.41	158.78	90.73	38.89	38.89	NA	NA	NA	5.00
Ducks II, Geese II, Other Waterfowl, Screamers II	1.00	1.66	2.14	2.01	1.55	0.99	0.68	0.24	0.47	0.24	NA	NA	NA	NA	0.00
Bustards	1.00	1.56	2.16	2.26	2.03	1.16	0.65	0.41	NA	NA	NA	NA	NA	NA	0.00
Bee-eaters	1.00	1.53	1.85	1.71	1.37	1.10	0.60	0.38	0.33	NA	NA	NA	NA	NA	0.00
Australasian Wrens	1.00	3.93	11.31	20.33	29.32	33.66	29.38	25.55	15.84	12.26	8.18	16.35	NA	NA	5.00
Pheasants, Quail, Guineafowl	1.00	5.42	15.22	20.83	20.51	13.38	9.09	3.33	3.07	1.02	2.05	NA	NA	NA	3.00
Cormorants, Anhingas, Gannets, Frigatebirds	1.00	3.16	5.68	6.26	5.73	4.02	2.04	1.59	0.23	NA	NA	NA	NA	NA	0.00
Orioles, Allies	1.00	46.93	287.00	492.00	537.71	489.14	342.86	219.43	137.14	36.57	NA	NA	NA	NA	4.00
Ibises	1.00	3.19	7.93	13.17	17.25	16.39	17.01	13.15	5.96	4.21	2.81	2.81	NA	NA	0.00
Sparrows, Snowfinches, Allies	1.00	3.96	9.37	12.05	11.67	8.20	7.05	4.25	2.38	1.36	NA	NA	NA	NA	0.00
Trogons	1.00	2.62	4.52	5.66	6.00	4.83	2.78	2.53	0.61	1.62	0.81	NA	NA	NA	0.00

Whistlers, Allies	1.00	8.58	36.85	152.15	353.54	727.38	1044.92	1393.23	1462.15	1201.23	1260.31	827.08	472.62	315.08	8.00
Australasian Robins	1.00	6.26	20.59	28.06	30.32	24.25	17.18	8.42	4.04	1.35	NA	NA	NA	NA	4.00
Antpittas	1.00	1.87	3.08	3.80	3.29	2.66	1.91	1.05	0.60	1.20	NA	NA	NA	NA	0.00
Curassows, Chacalacas, Guans	1.00	2.63	3.74	3.90	2.85	2.09	1.11	0.94	0.34	NA	NA	NA	NA	NA	0.00
Cardinals, Allies	1.00	2.55	4.67	6.21	6.47	5.32	3.27	2.18	1.24	0.83	NA	NA	NA	NA	0.00
Manakins	1.00	1.64	2.32	2.43	1.93	1.26	0.61	0.61	0.12	NA	NA	NA	NA	NA	0.00
Broadbills, Pittas, Asities, Allies	1.00	2.98	8.30	17.64	26.60	30.86	29.87	25.51	26.17	20.06	13.96	3.49	NA	NA	5.00
Tapaculos	1.00	9.78	37.25	90.72	168.37	263.63	325.87	348.77	317.06	258.35	140.92	56.37	37.58	75.16	7.00
Hornbills	1.00	3.55	7.41	9.62	10.38	8.69	7.14	3.98	1.77	1.18	1.18	NA	NA	NA	0.00
Vireos, Allies	1.00	2.12	3.22	3.40	3.51	2.31	1.31	1.00	0.31	NA	NA	NA	NA	NA	0.00
Wagtails, Pipits	1.00	2.85	5.54	7.52	8.95	7.52	5.01	3.14	2.42	0.97	NA	NA	NA	NA	0.00
Herons	1.00	1.77	2.65	3.01	2.87	1.66	1.05	0.61	0.41	1.09	NA	NA	NA	NA	0.00
Thornbills, Gerygones	1.00	2.58	4.42	5.19	5.24	4.37	3.12	1.86	1.17	0.78	1.56	NA	NA	NA	0.00
Falcons, Caracaras	1.00	1.55	1.89	1.67	1.27	0.86	0.52	0.05	0.11	NA	NA	NA	NA	NA	0.00
Cotingas	1.00	1.69	2.43	2.45	2.00	1.32	0.78	0.38	0.38	NA	NA	NA	NA	NA	0.00
Babblers II, Old World Warblers II, Allies	1.00	154.18	537.09	1163.64	1578.18	1719.27	1512.73	1093.82	581.82	279.27	372.36	372.36	NA	NA	5.00
Tits, Chickadees, Penduline-Tits, Allies	1.00	2.26	3.68	4.22	4.76	3.13	2.27	1.72	0.52	0.69	NA	NA	NA	NA	0.00
Cuckoo-Shrikes	1.00	2.78	5.24	6.75	7.32	6.32	5.15	2.61	2.04	0.45	NA	NA	NA	NA	0.00
Swallows, Martins	1.00	3.31	8.15	13.87	18.44	18.80	20.41	11.40	7.36	6.62	5.89	NA	NA	NA	6.00
Nightjars	1.00	1.48	1.91	1.68	1.18	1.14	0.41	0.33	0.22	0.22	NA	NA	NA	NA	0.00
Larks	1.00	8.06	16.45	19.53	17.99	14.47	6.81	2.84	2.84	1.14	2.27	NA	NA	NA	0.00

Kingfishers, Motmots, Todies	1.00	1.71	2.38	2.70	1.92	1.28	1.01	0.32	0.13	2.03	NA	NA	NA	NA	0.00
Waders, Allies	1.00	2.42	4.24	4.89	5.02	3.91	2.14	1.68	0.93	NA	NA	NA	NA	NA	0.00
New World Blackbirds	1.00	5.06	12.09	17.60	22.89	21.21	17.74	10.79	4.79	2.88	3.84	3.84	NA	NA	4.00
Swifts, Treeswifts	1.00	1.75	2.15	2.25	1.59	1.28	0.82	0.55	NA	NA	NA	NA	NA	NA	0.00
Old World Warblers I	1.00	1.78	2.36	2.46	1.98	1.40	0.98	0.38	0.38	0.51	NA	NA	NA	NA	0.00
Ducks III, Geese II, Other Waterfowl, Screamers III	1.00	19.63	33.90	34.29	26.60	17.73	8.03	6.10	1.11	2.22	NA	NA	NA	NA	3.00
Toucans, Barbets	1.00	1.68	1.64	1.29	0.77	0.37	0.24	NA	NA	NA	NA	NA	NA	NA	0.00
New World Warblers	1.00	3.90	17.59	35.32	48.14	51.39	40.81	32.28	23.00	13.36	5.94	5.94	NA	NA	5.00
Albatrosses, Shearwaters, Petrels	1.00	18.72	87.85	170.07	196.15	165.93	147.75	66.37	41.09	25.28	12.64	NA	NA	NA	4.00
Bulbuls, Allies	1.00	4.21	14.78	40.30	83.65	110.97	96.12	70.57	43.80	21.41	11.68	NA	NA	NA	5.00
Wrens, Gnatcatchers, Nuthatches, Wallcreeper, Treecreepers	1.00	1.75	2.29	2.31	1.74	1.52	0.65	0.25	NA	NA	NA	NA	NA	NA	0.00
Rails, Finfoots	1.00	1.87	2.65	2.56	2.38	1.61	1.17	0.47	0.27	NA	NA	NA	NA	NA	0.00
Cuckoos	1.00	1.64	2.33	2.68	1.97	1.83	0.91	0.50	0.50	0.25	NA	NA	NA	NA	0.00
Shrike-Flycatchers, Helmetshrikes, Vangas, Butcherbirds, Woodswallows,	1.00	161.00	4090.00	7428.00	9024.00	8592.00	6016.00	3456.00	2816.00	1280.00	512.00	NA	NA	NA	4.00

Allies														
Cisticolas, Allies	1.00	1.98	2.83	3.03	2.74	1.96	1.21	0.78	NA	NA	NA	NA	NA	NA	0.00
Mockingbirds, Thrashers, Philippine Creepers, Starlings	1.00	3.76	9.34	14.73	17.22	18.28	12.86	8.64	4.99	1.54	1.54	NA	NA	NA	0.00
Gulls, Terns, Auks, Crab Plover	1.00	2456.00	9244.00	12408.00	10208.00	6400.00	3264.00	2048.00	1024.00	NA	NA	NA	NA	NA	3.00
Thrushes	1.00	21.97	84.87	188.93	306.93	407.47	436.27	401.07	247.47	145.07	51.20	34.13	NA	NA	6.00
Tanagers I, Allies	1.00	31.53	157.16	329.05	520.84	555.79	466.53	309.89	202.11	121.26	53.89	NA	NA	NA	5.00
Buntings, American Sparrows, Brush-Finches	1.00	2.72	6.08	9.73	12.18	11.06	10.23	9.40	7.19	3.32	4.42	4.42	NA	NA	0.00
Parrots II	1.00	4.67	13.85	23.10	27.86	25.63	21.83	14.64	6.51	3.25	NA	NA	NA	NA	4.00
Waxbills, Allies	1.00	420.67	2032.00	4330.67	6352.00	6410.67	5952.00	4309.33	2645.33	1877.33	1024.00	1365.33	NA	NA	5.00
Parrots III	1.00	7.91	18.52	25.34	24.50	17.40	10.75	4.81	1.28	NA	NA	NA	NA	NA	3.00
Pheasants, Quail, Guineafowl	1.00	3.61	7.40	9.95	10.95	8.32	5.20	3.71	1.78	0.59	2.38	NA	NA	NA	0.00
Honeyeaters	1.00	2.38	4.01	4.81	5.21	3.85	2.33	1.74	0.55	0.73	NA	NA	NA	NA	0.00
Owls	1.00	2.46	3.75	4.03	3.11	2.07	1.14	0.59	NA	NA	NA	NA	NA	NA	0.00
Finches, Allies	1.00	5.38	20.71	39.73	43.72	39.98	27.57	20.48	7.09	9.45	3.15	6.30	NA	NA	4.00
Tanagers II, Flowerpiercers, Conebills, Seedeaters, Warbling-finches, Allies	1.00	54.00	2100.00	7560.00	11904.00	13904.00	13216.00	9920.00	8320.00	2560.00	1536.00	2048.00	NA	NA	5.00

Antbirds	1.00	24.73	78.12	116.55	112.31	91.29	69.02	40.16	22.59	5.02	NA	NA	NA	NA	3.00
Woodpeckers	1.00	1.06	0.69	0.34	0.18	0.05	NA	NA	NA	NA	NA	NA	NA	NA	0.00
Hawks, Eagles, Secretarybird	1.00	1.47	1.58	1.39	0.97	0.44	0.33	0.15	NA	NA	NA	NA	NA	NA	0.00
Chats, Old World Flycatchers	1.00	216.40	828.40	1256.00	1284.80	988.80	723.20	396.80	179.20	204.80	NA	NA	NA	NA	4.00
Ovenbirds, Woodcreepers	1.00	4.98	13.57	22.43	30.35	34.58	32.07	21.48	16.04	11.45	2.29	9.16	NA	NA	5.00
Pigeons, Doves	1.00	1.76	2.24	1.89	1.49	1.03	0.51	0.18	0.24	NA	NA	NA	NA	NA	0.00
Hummingbirds	1.00	1.87	2.40	1.97	1.55	0.71	0.56	0.25	NA	NA	NA	NA	NA	NA	0.00
Shrikes, Monarchs, Drongos, Fantails, Birds Of Paradise, Crows, Jays, Allies	1.00	5.55	12.53	17.76	19.04	14.04	9.59	6.55	1.87	0.94	NA	NA	NA	NA	0.00
Whiteyes, Babblers I, Parrotbills	1.00	4.96	8.09	8.90	9.36	5.31	3.33	1.43	1.27	0.63	NA	NA	NA	NA	0.00
Tyrant-Flycatchers, Cotingas, Allies	1.00	47.73	143.45	238.59	271.37	222.12	148.08	75.29	50.20	40.16	20.08	NA	NA	NA	4.00

Figures S17: S104. Avian clades scaled by the rate of body mass evolution, as described by variable-rates approaches (a) BayesTraits, and (b) BAMM-flip. Branches are coloured by the rate of evolution (i.e. the proportion between the scaled branch length and the length of the same branch in the original input phylogeny). The colour scheme goes from blue (low rates) to red (high rates), and rates bigger than x10 are all represented in red regardless of their magnitude. Figures S17-S63 are groups where the single-process model (i.e. BM, OU and EB) with highest AICw is the BM method, followed by the OU (Figures S64-S86), and EB (Figures S87-S104) models. (c) Model inadequacy for single-process and variable-rates approaches represented by differences in the distributions of observed and simulated test statistics (significance at $P < 0.05$). Inadequacy measures model ability to account for total rate variation (p_cvar), and further, variation related to time (p_shgt), branch lengths (p_svar), and ancestral states (p_sasr). Positive or negative differences between test statistics are marked by (+) and (-), respectively. For single-process models, the AICw and parameter estimates for the rate of evolution (*sigma*), the OU constraint force (*alpha*) and the EB rate-decay (*r*) are also given.

Australasian Babblers, Logrunners

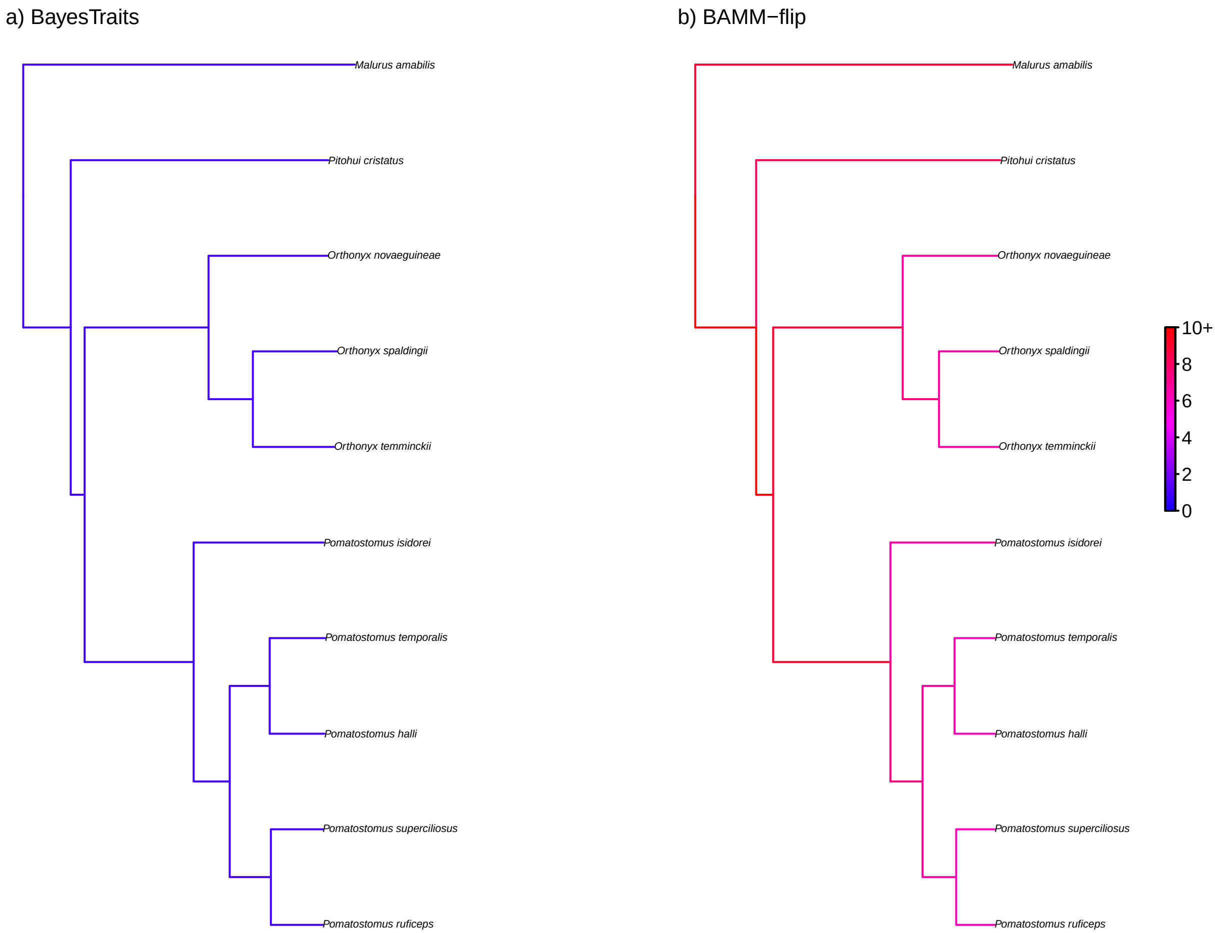

c)

	p_cvar	*p_shgt*	*p_svar*	*p_sasr*	*AICw*	*sigma*	*alpha*	*r*
BM	0.61 (+)	0.66 (+)	0.6 (+)	0.39 (−)	0.57	5.32		
OU	0.66 (+)	0.67 (−)	0.63 (−)	0.38 (+)	0.21	5.32	0	
EB	0.78 (+)	0.79 (+)	0.8 (+)	0.46 (−)	0.22	7.49		−3.95
BayesTraits	0.7 (+)	0.7 (+)	0.51 (+)	0.41 (−)				
BAMM−flip	0.81 (−)	0.78 (−)	0.48 (+)	0.92 (+)				

Figure S17

Owlet−Nightjars

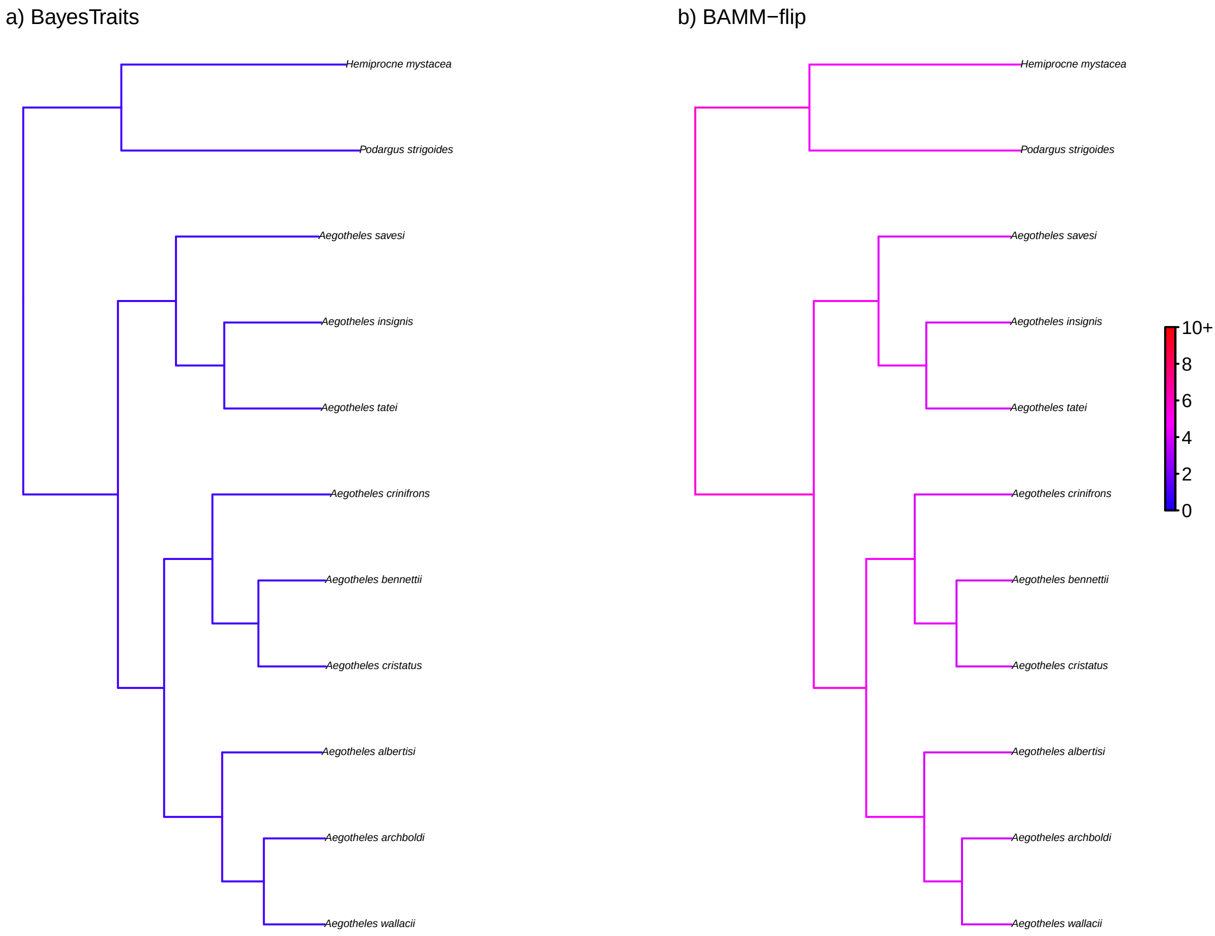

c)

	p_cvar	*p_shgt*	*p_svar*	*p_sasr*	*AICw*	*sigma*	*alpha*	*r*
BM	0.78 (−)	0.56 (+)	0.3 (+)	0.12 (+)	0.57	3.28		
OU	0.76 (−)	0.42 (+)	0.19 (+)	0.11 (−)	0.22	4.04	2.2	
EB	0.65 (−)	0.6 (+)	0.32 (+)	0.13 (+)	0.21	3.28		0
BayesTraits	0.73 (−)	0.61 (+)	0.27 (+)	0.14 (+)				
BAMM−flip	0.51 (−)	0.86 (+)	0.3 (+)	0.19 (+)				

Figure S18

Ducks I, Geese I, Other Waterfowl, Screamers I

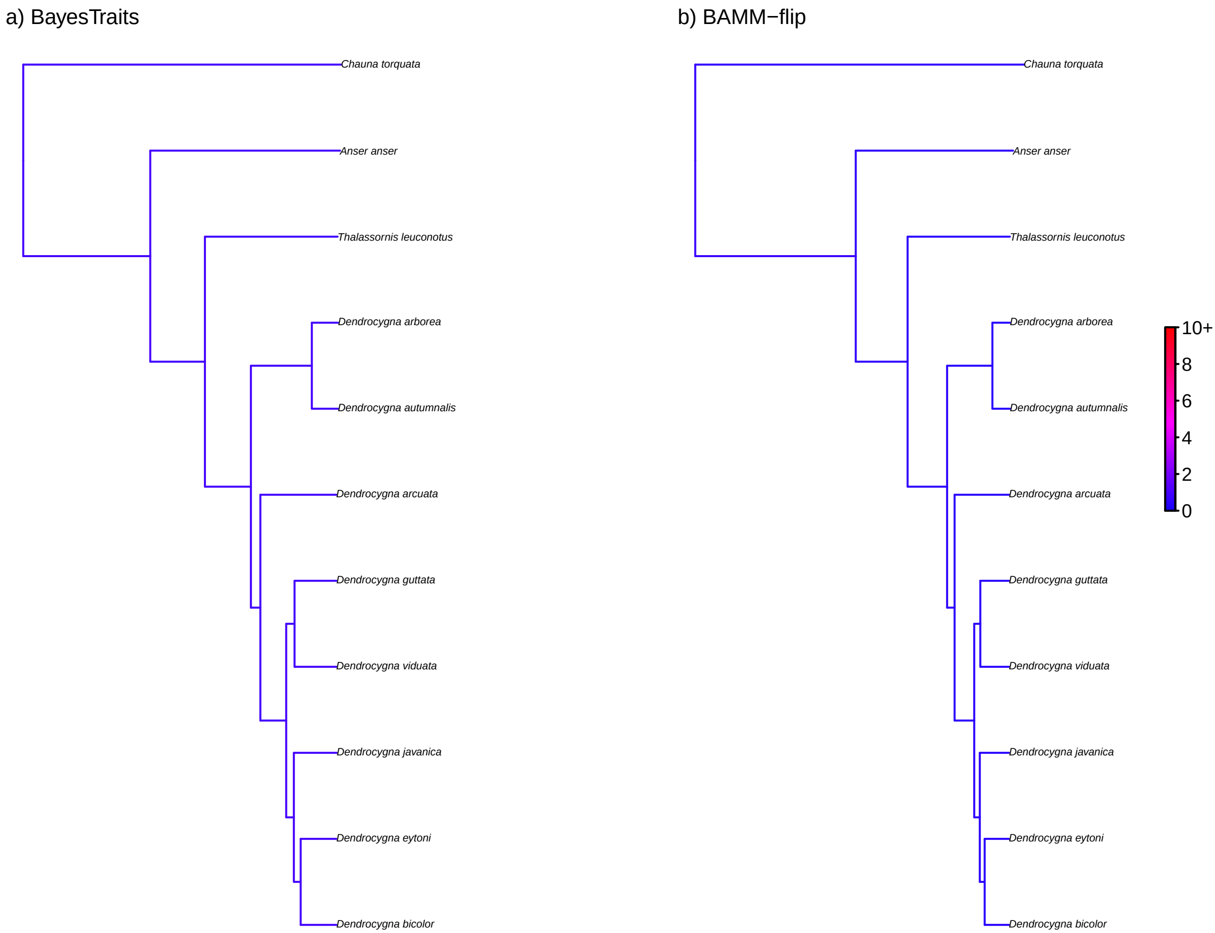

c)

	p_cvar	*p_shgt*	*p_svar*	*p_sasr*	*AICw*	*sigma*	*alpha*	*r*
BM	0.55 (+)	0.14 (+)	0.14 (+)	0.19 (+)	0.54	0.38		
OU	0.55 (−)	0.15 (+)	0.16 (+)	0.21 (+)	0.2	0.38	0	
EB	0.7 (+)	0.42 (+)	0.42 (+)	0.35 (+)	0.26	1.22		−1.19
BayesTraits	0.57 (+)	0.15 (+)	0.16 (+)	0.18 (+)				
BAMM−flip	0.62 (+)	0.17 (+)	0.16 (+)	0.16 (+)				

Figure S19

Waxwings, Allies

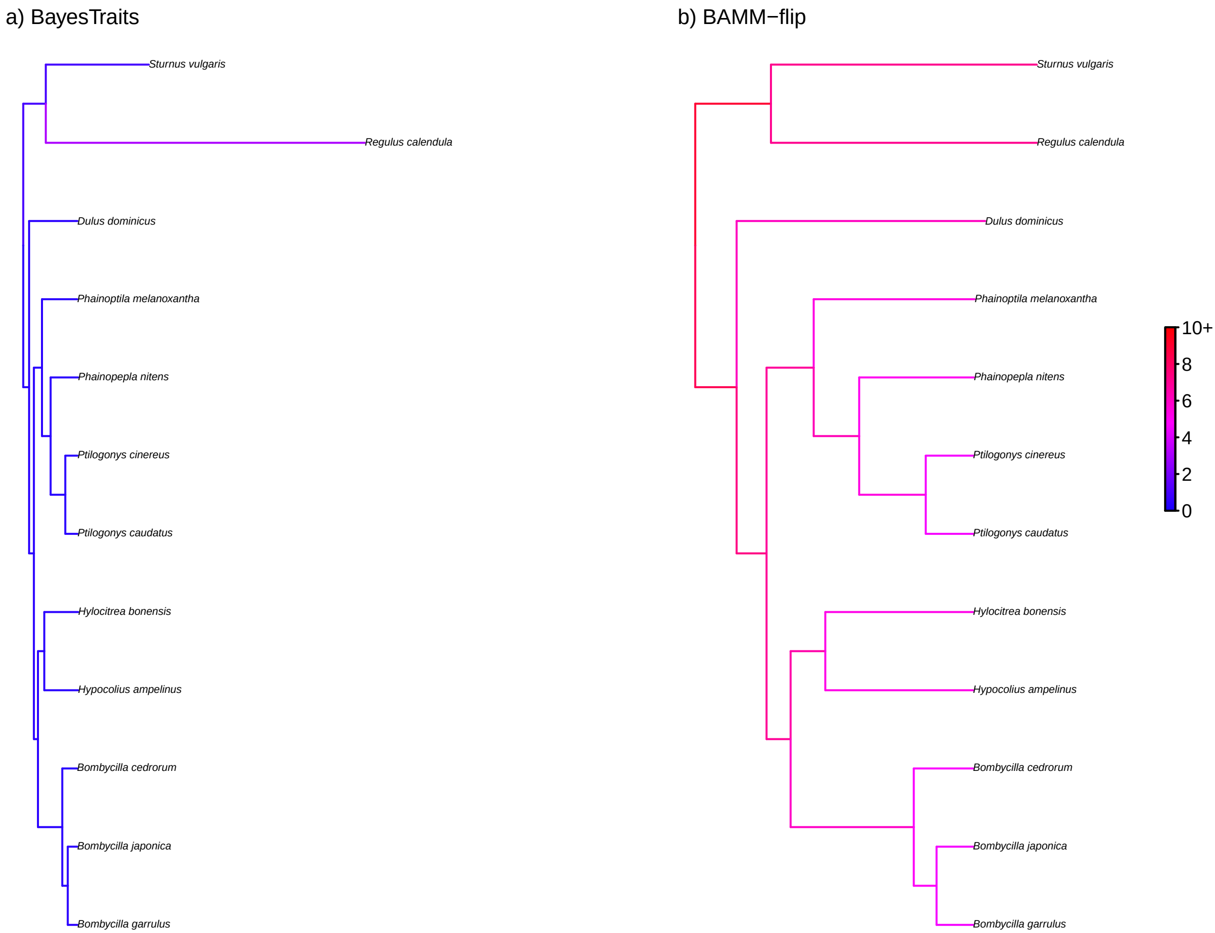

c)

	p_cvar	*p_shgt*	*p_svar*	*p_sasr*	*AICw*	*sigma*	*alpha*	*r*
BM	0.15 (+)	0.41 (+)	0.02 (+)	0 (−)	0.56	4.72		
OU	0.14 (+)	0.4 (+)	0.03 (+)	0 (+)	0.21	4.72	0	
EB	0.27 (+)	0.84 (+)	0.3 (+)	0.01 (−)	0.23	13.96		−17.31
BayesTraits	0.7 (+)	0.26 (−)	0.15 (+)	0.73 (+)				
BAMM−flip	0.82 (+)	0.36 (−)	0.11 (+)	0.27 (−)				

Figure S20

Cormorants, Anhingas, Gannets, Frigatebirds

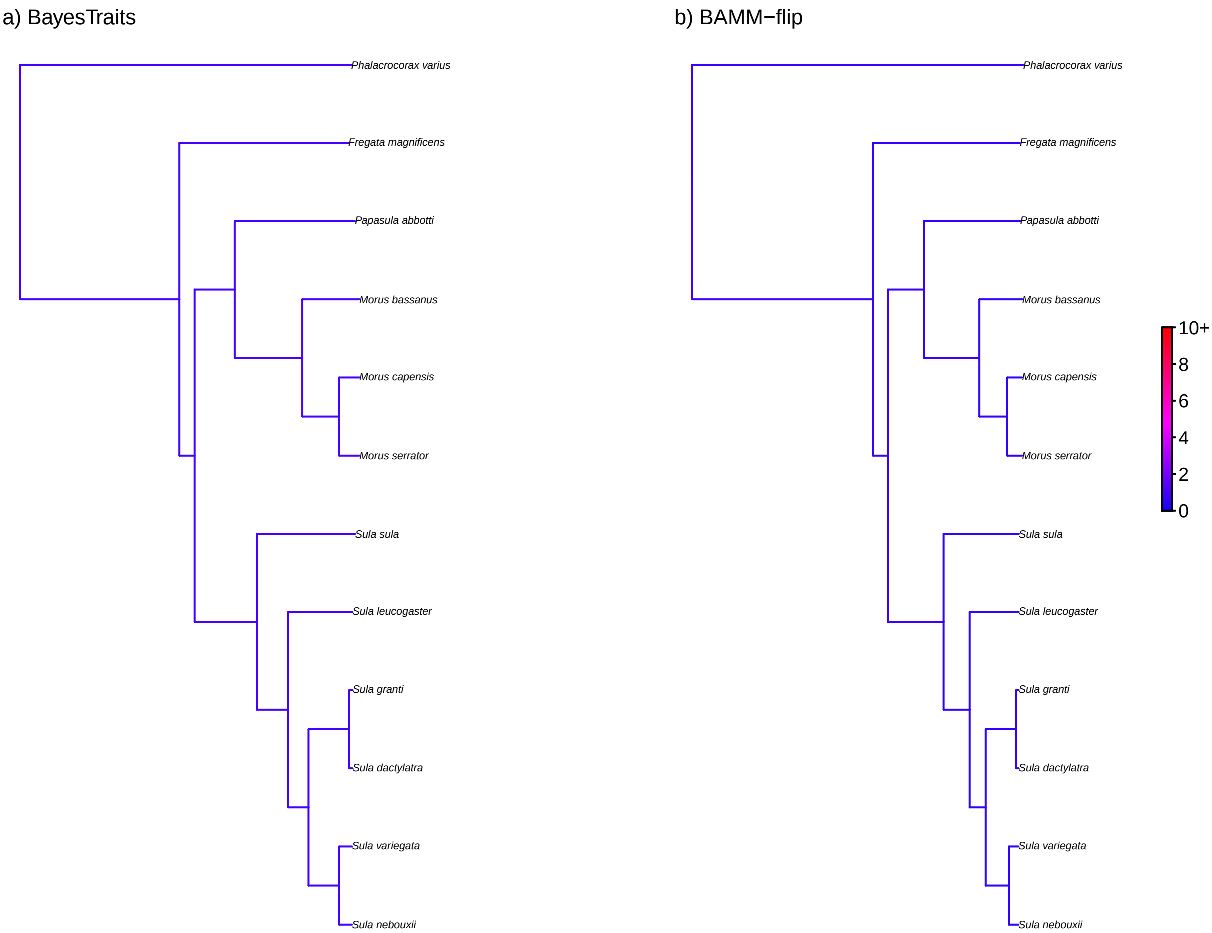

c)

	p_cvar	*p_shgt*	*p_svar*	*p_sasr*	*AICw*	*sigma*	*alpha*	*r*
BM	0.6 (−)	0.83 (+)	0.49 (+)	0.86 (+)	0.57	0.49		
OU	0.63 (−)	0.46 (+)	0.24 (+)	0.89 (−)	0.22	0.6	1.94	
EB	0.63 (−)	0.84 (+)	0.45 (+)	0.91 (+)	0.21	0.49		0
BayesTraits	0.59 (−)	0.88 (+)	0.52 (+)	0.93 (+)				
BAMM−flip	0.53 (−)	0.9 (+)	0.48 (+)	0.95 (+)				

Figure S21

Accentors, Olive Warbler

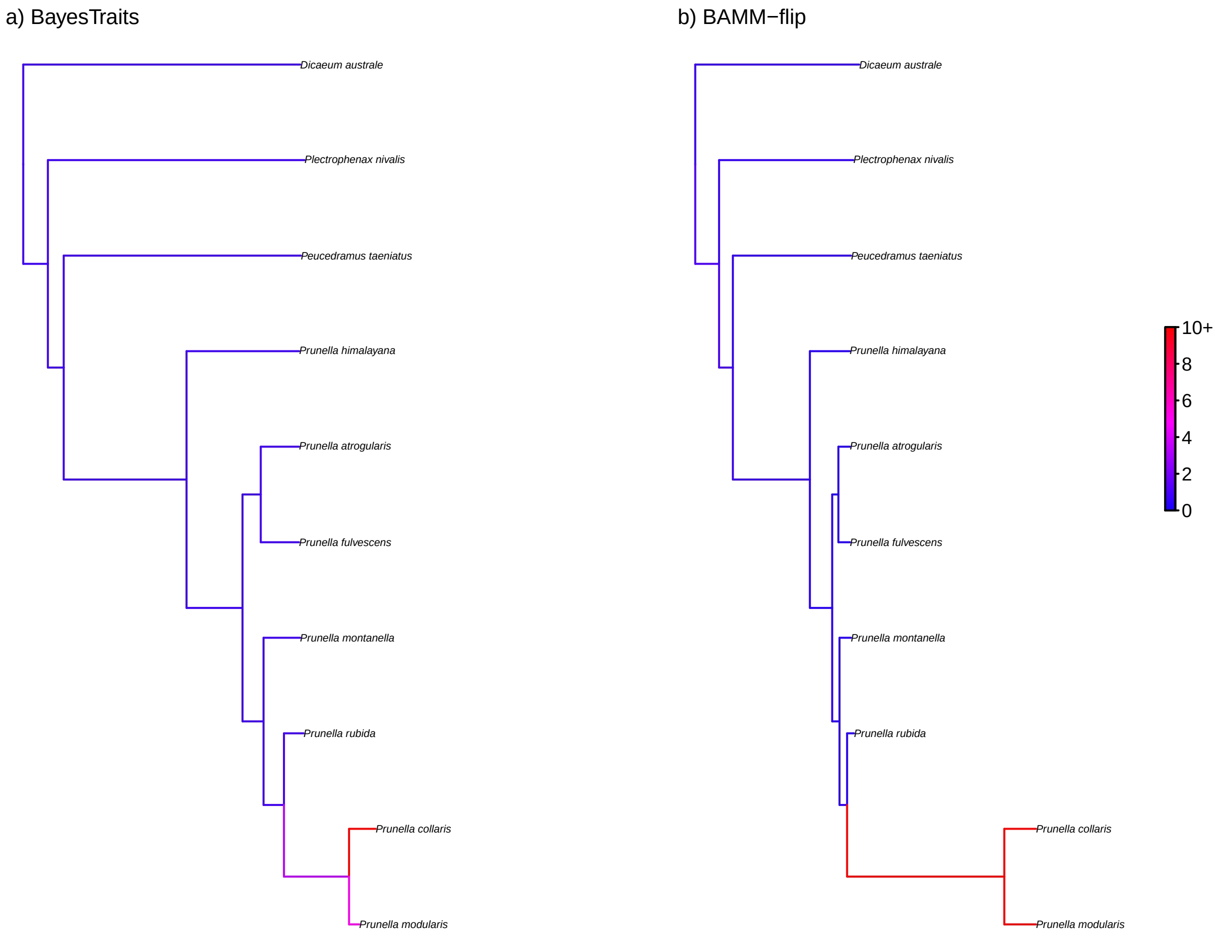

c)

	p_cvar	*p_shgt*	*p_svar*	*p_sasr*	*AICw*	*sigma*	*alpha*	*r*
BM	0 (+)	0.11 (−)	0.01 (−)	0 (+)	0.73	4.98		
OU	NA	NA	NA	NA	NA	NA	NA	NA
EB	0 (+)	0.13 (−)	0.04 (−)	0 (+)	0.27	4.98		0
BayesTraits	0.32 (+)	0.8 (−)	0.96 (+)	0 (+)				
BAMM−flip	0.91 (−)	0.59 (−)	0.08 (+)	0.11 (+)				

Figure S22

Cranes, Trumpeters, Limpkin

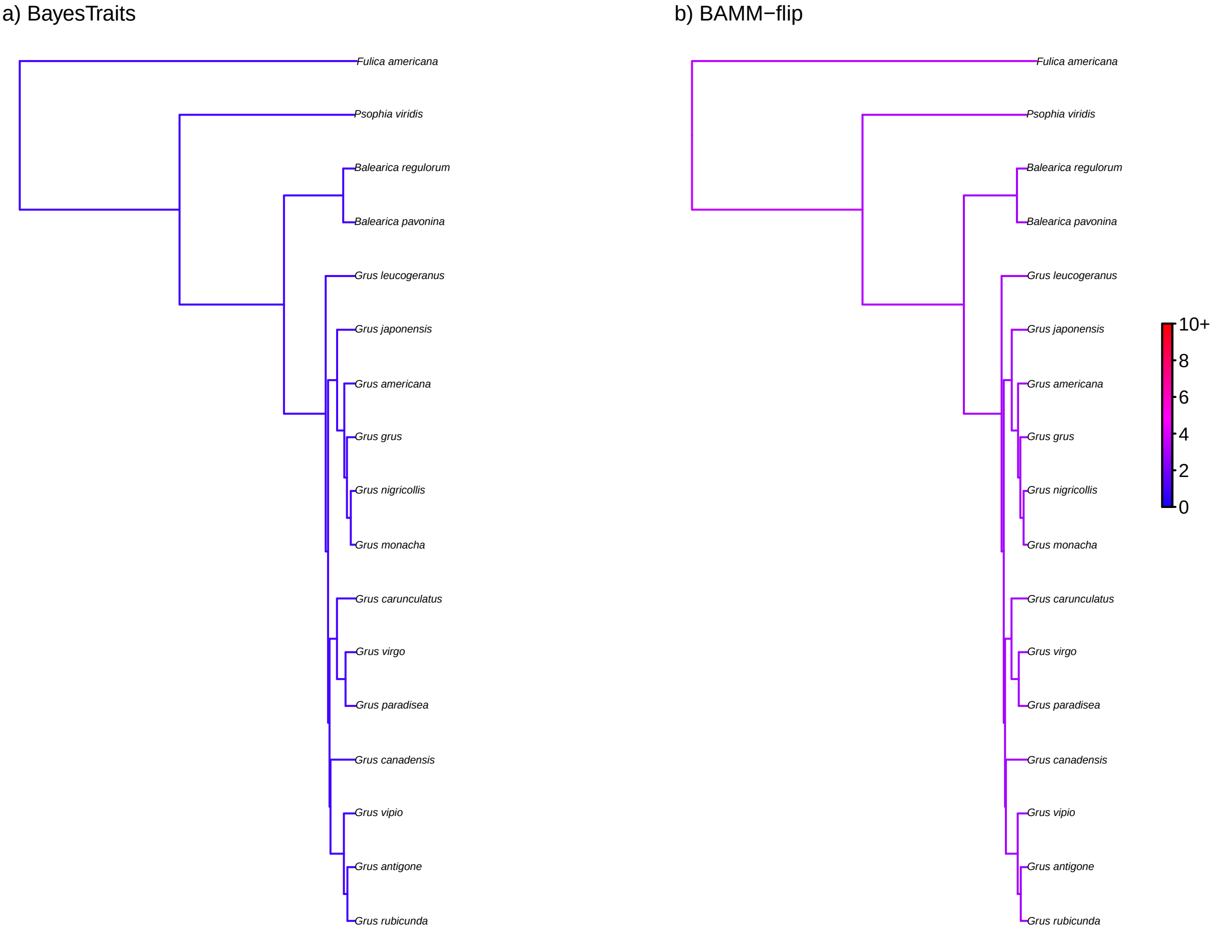

c)

	p_cvar	*p_shgt*	*p_svar*	*p_sasr*	*AICw*	*sigma*	*alpha*	*r*
BM	0.48 (+)	0.98 (−)	0.92 (−)	0.61 (−)	0.48	2.49		
OU	0.71 (+)	0.35 (+)	0.36 (+)	0.21 (+)	0.35	3.08	1.85	
EB	0.53 (+)	0.97 (−)	0.9 (−)	0.56 (−)	0.18	2.49		0
BayesTraits	0.54 (+)	0.94 (−)	0.97 (−)	0.58 (−)				
BAMM−flip	0.54 (+)	0.97 (−)	0.92 (−)	0.62 (−)				

Figure S23

Grosbeaks, Saltators, Allies

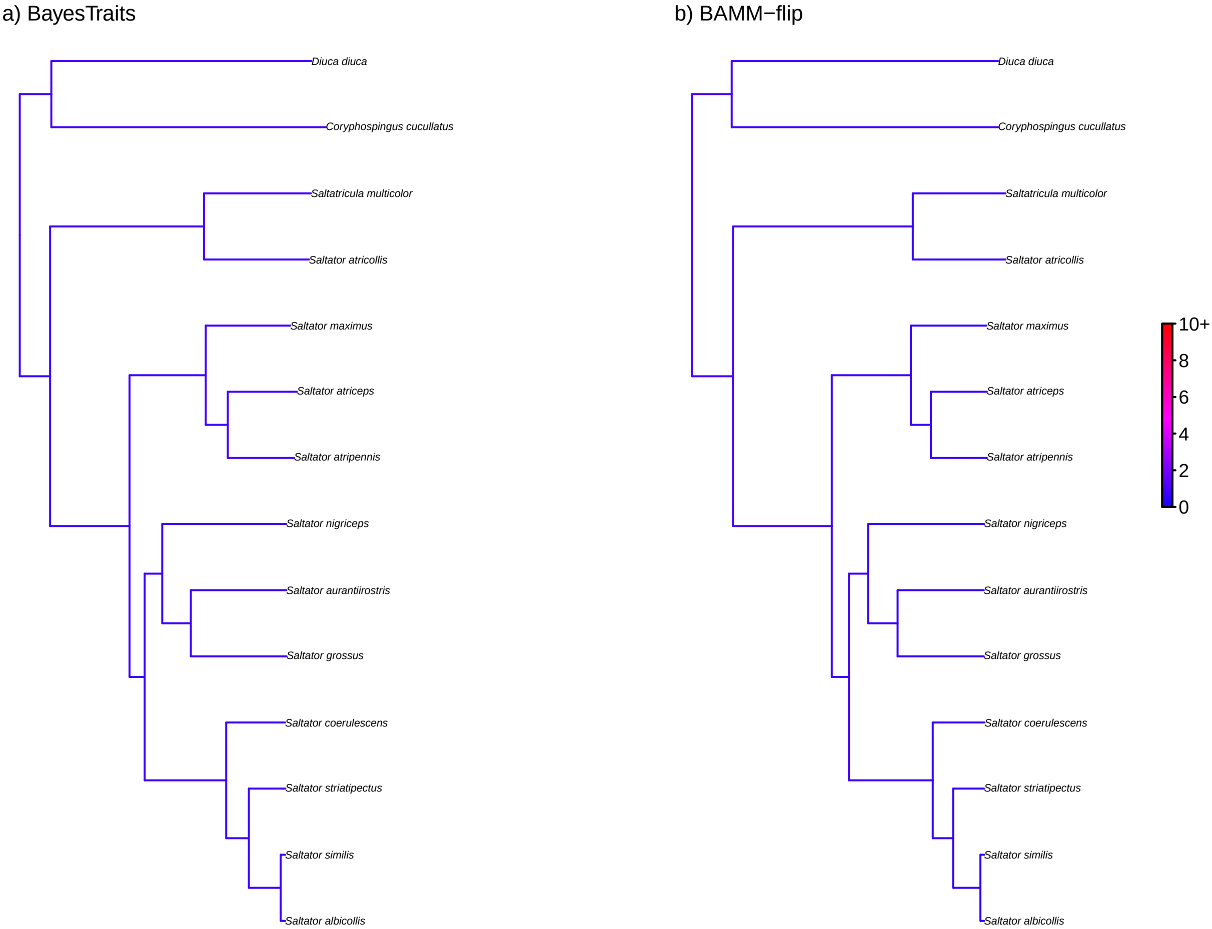

c)

	p_cvar	*p_shgt*	*p_svar*	*p_sasr*	*AICw*	*sigma*	*alpha*	*r*
BM	0.92 (−)	0.47 (+)	0.26 (+)	0.28 (−)	0.58	0.62		
OU	0.94 (+)	0.43 (+)	0.26 (+)	0.27 (+)	0.21	0.63	0.09	
EB	0.96 (−)	0.44 (+)	0.28 (+)	0.3 (−)	0.21	0.62		0
BayesTraits	0.82 (−)	0.53 (+)	0.21 (+)	0.29 (−)				
BAMM−flip	0.58 (−)	0.76 (+)	0.2 (+)	0.49 (−)				

Figure S24

Whipbirds, Quail−Thrushes, Allies

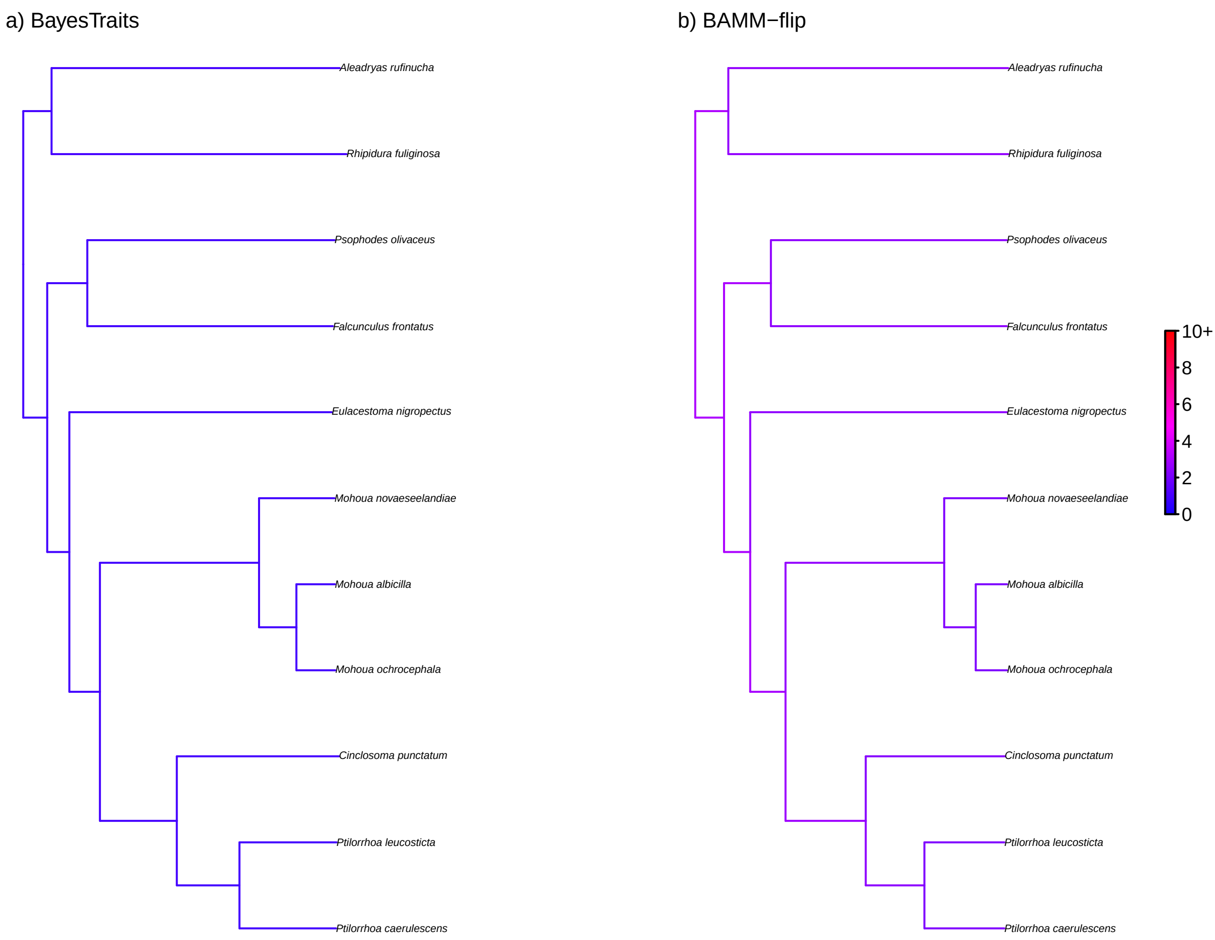

c)

	p_cvar	*p_shgt*	*p_svar*	*p_sasr*	*AICw*	*sigma*	*alpha*	*r*
BM	0.2 (−)	0.78 (+)	0.56 (+)	0.32 (−)	0.57	1.82		
OU	0.21 (−)	0.6 (+)	0.46 (+)	0.4 (−)	0.22	2.43	1.14	
EB	0.17 (−)	0.85 (+)	0.61 (+)	0.34 (−)	0.21	1.82		0
BayesTraits	0.19 (−)	0.81 (+)	0.61 (+)	0.33 (−)				
BAMM−flip	0.23 (−)	0.93 (+)	0.59 (+)	0.38 (−)				

Figure S25

African Warblers

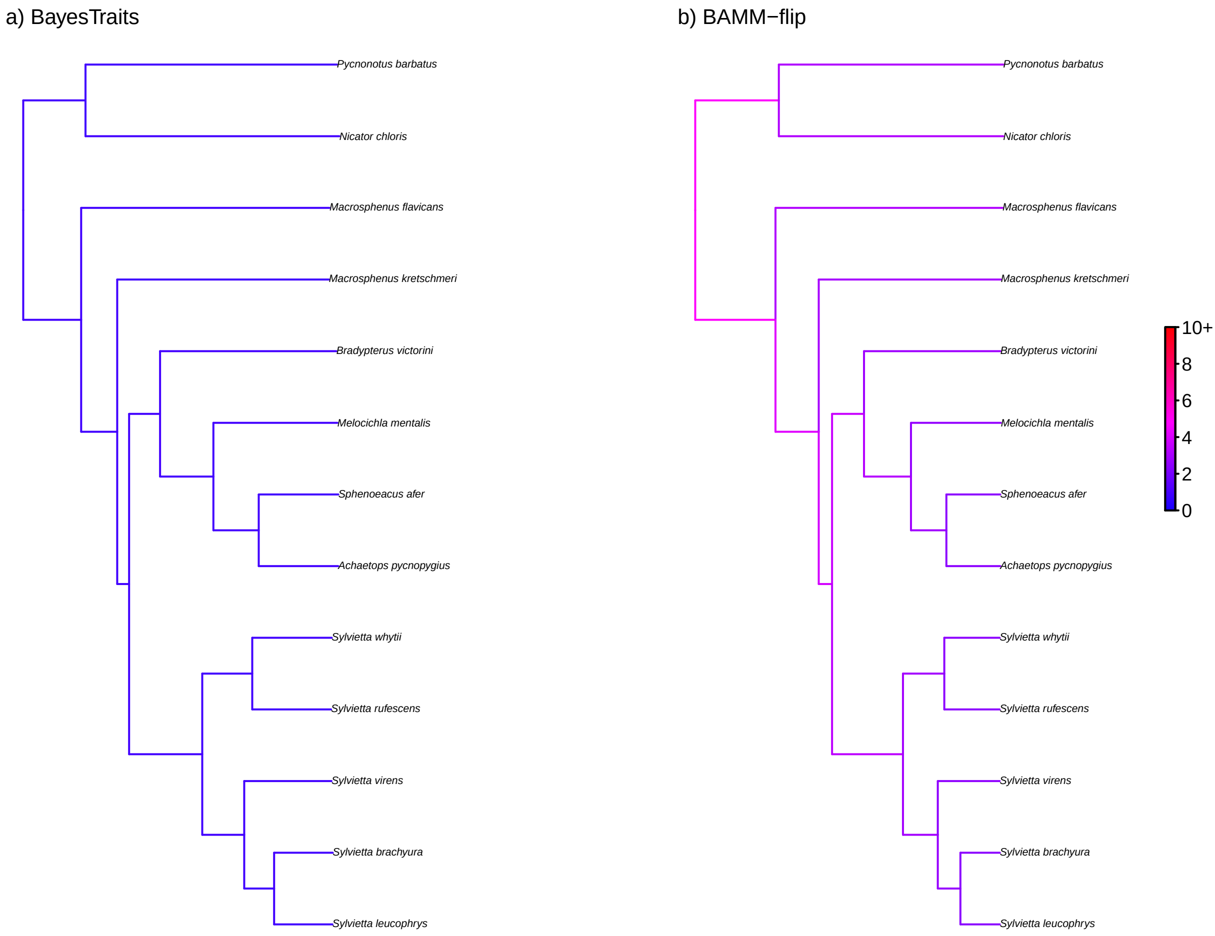

c)

	p_cvar	p_shgt	p_svar	p_sasr	AICw	sigma	alpha	r
BM	0.49 (+)	0.15 (+)	0.51 (+)	0.92 (+)	0.5	2.44		
OU	0.49 (−)	0.18 (+)	0.56 (+)	0.96 (+)	0.18	2.44	0	
EB	0.69 (+)	0.65 (+)	0.75 (+)	0.76 (−)	0.32	9.79		−19.02
BayesTraits	0.49 (+)	0.13 (+)	0.48 (+)	0.93 (+)				
BAMM−flip	0.48 (+)	0.18 (+)	0.58 (+)	0.98 (−)				

Figure S26

Australian Treecreepers, Bowerbirds

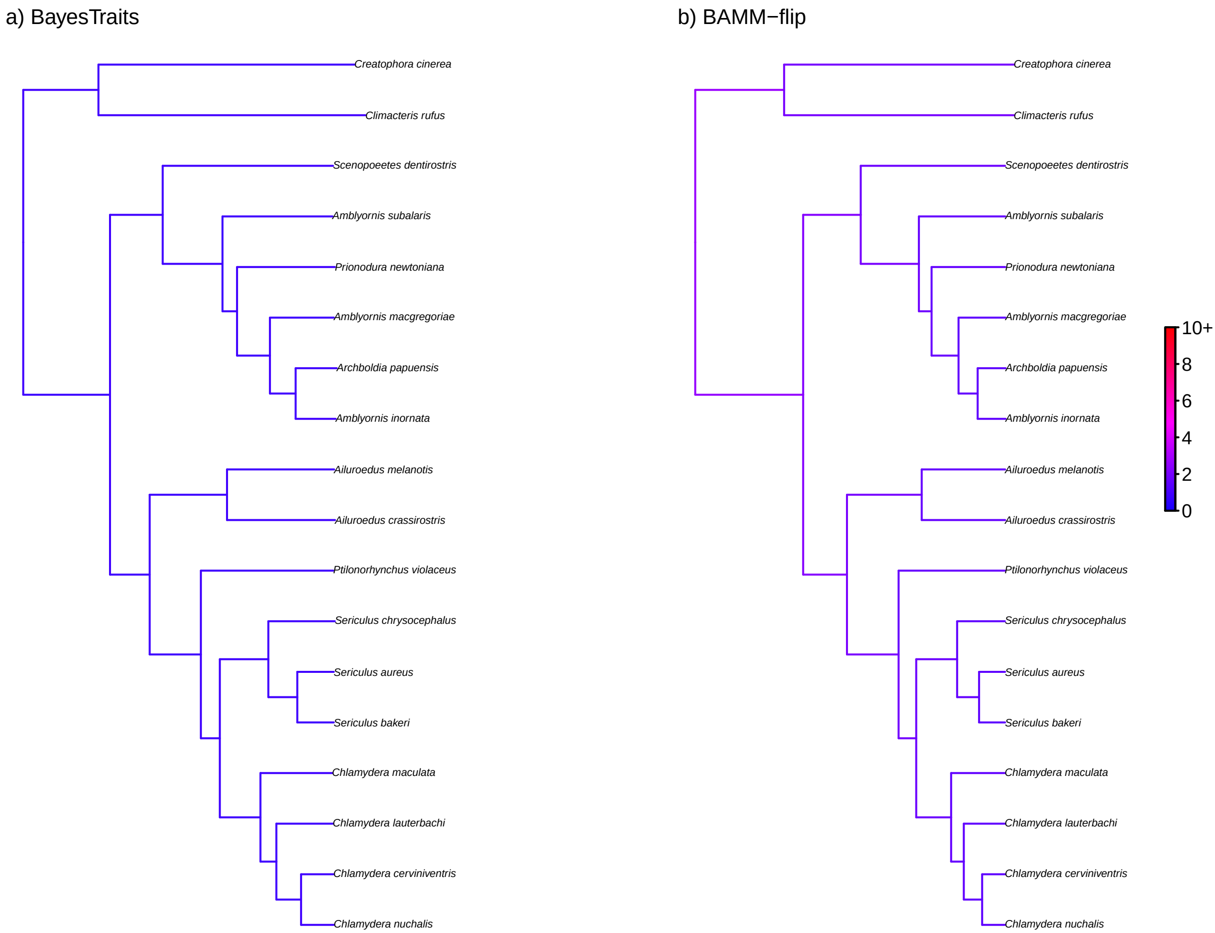

c)

	p_cvar	*p_shgt*	*p_svar*	*p_sasr*	*AICw*	*sigma*	*alpha*	*r*
BM	0.76 (−)	0.34 (+)	0.47 (+)	0.19 (−)	0.57	1.5		
OU	0.73 (+)	0.3 (+)	0.42 (+)	0.21 (+)	0.21	1.5	0	
EB	0.66 (−)	0.44 (+)	0.57 (+)	0.25 (−)	0.23	2.42		−3.37
BayesTraits	0.73 (−)	0.33 (+)	0.4 (+)	0.22 (−)				
BAMM−flip	0.64 (−)	0.42 (+)	0.49 (+)	0.29 (−)				

Figure S27

Parrots I

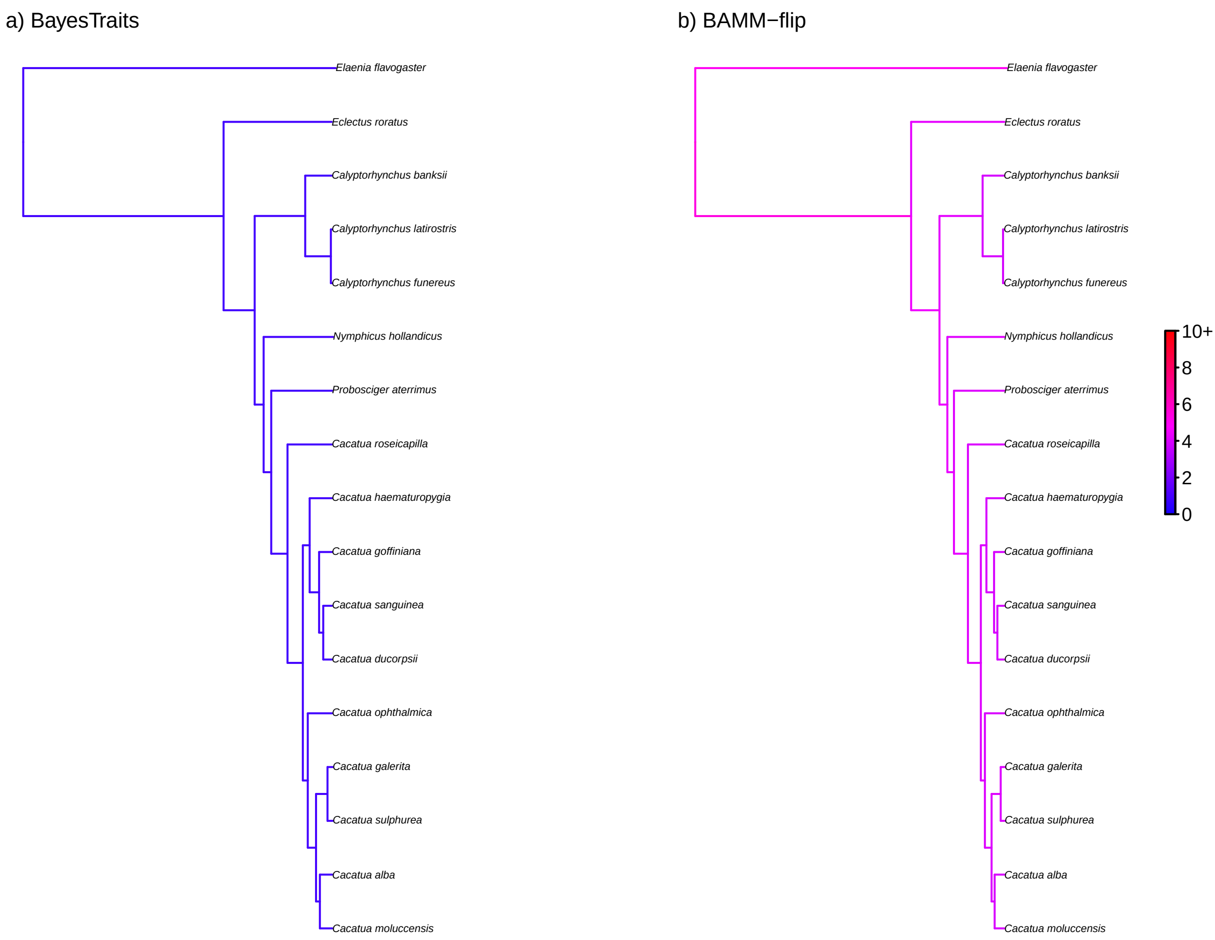

c)

	p_cvar	*p_shgt*	*p_svar*	*p_sasr*	*AICw*	*sigma*	*alpha*	*r*
BM	0.87 (+)	0.75 (+)	0.89 (−)	0.21 (−)	0.54	3.47		
OU	0.85 (+)	0.48 (+)	0.76 (+)	0.08 (+)	0.26	4.09	0.94	
EB	0.86 (+)	0.79 (+)	0.9 (−)	0.23 (−)	0.2	3.47		0
BayesTraits	0.94 (+)	0.72 (+)	0.98 (−)	0.23 (−)				
BAMM−flip	0.96 (−)	0.74 (+)	0.96 (−)	0.24 (−)				

Figure S28

Megapodes

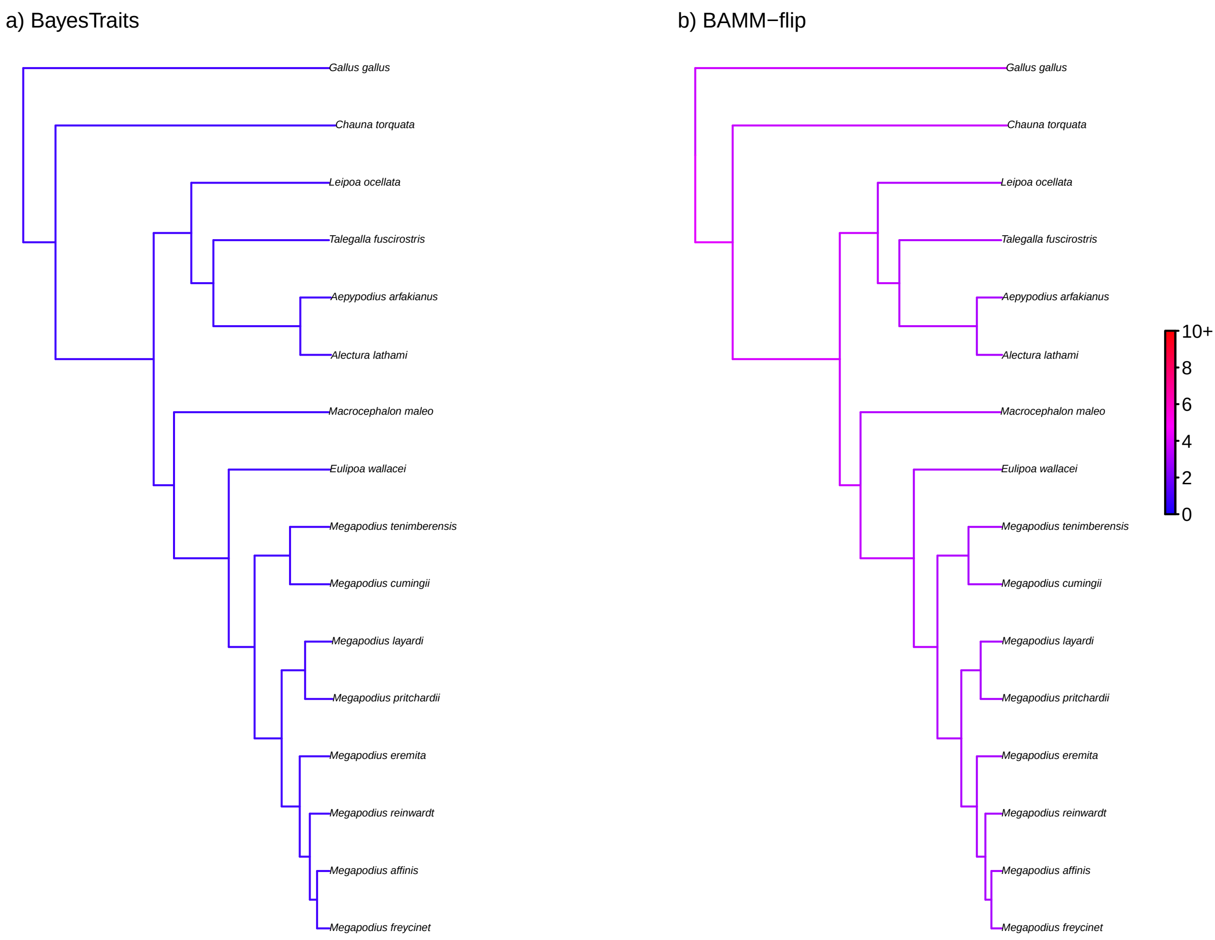

c)

	p_cvar	*p_shgt*	*p_svar*	*p_sasr*	*AICw*	*sigma*	*alpha*	*r*
BM	0.36 (−)	0.62 (+)	0.7 (+)	0.88 (−)	0.56	2.63		
OU	0.32 (+)	0.33 (+)	0.49 (+)	0.97 (−)	0.24	3.32	2.5	
EB	0.34 (−)	0.66 (+)	0.74 (+)	0.81 (−)	0.2	2.63		0
BayesTraits	0.29 (−)	0.6 (+)	0.7 (+)	0.88 (−)				
BAMM−flip	0.27 (−)	0.74 (+)	0.73 (+)	0.85 (−)				

Figure S29

Turacos

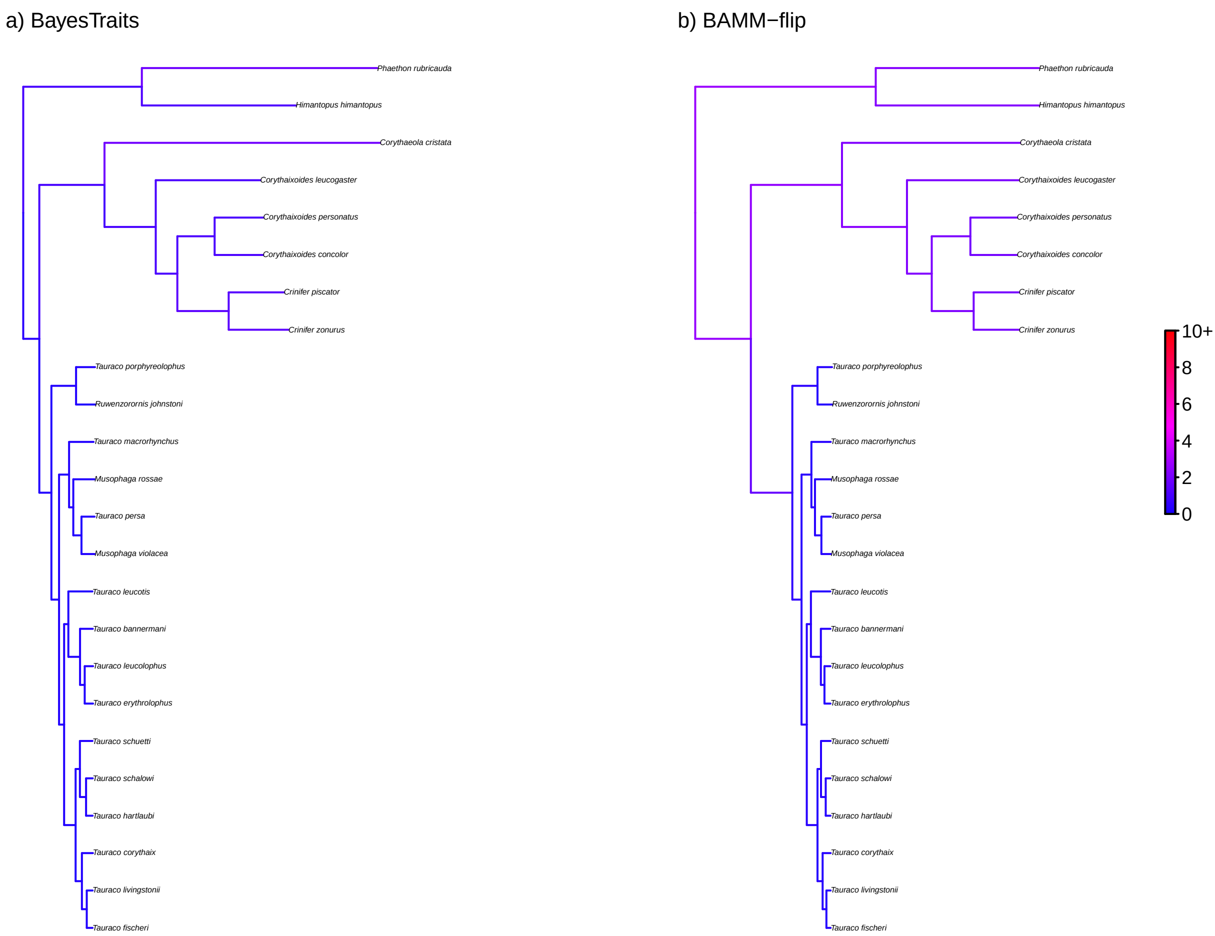

c)

	p_cvar	*p_shgt*	*p_svar*	*p_sasr*	*AICw*	*sigma*	*alpha*	*r*
BM	0.04 (+)	0.26 (+)	0.01 (+)	0 (+)	0.54	0.97		
OU	0.04 (−)	0.27 (+)	0.02 (+)	0 (+)	0.2	0.97	0	
EB	0.07 (+)	0.83 (−)	0.54 (+)	0 (+)	0.26	5.24		−8.96
BayesTraits	0.72 (−)	0.39 (−)	0.07 (+)	0.04 (+)				
BAMM−flip	0.47 (−)	0.59 (−)	0.31 (+)	0.05 (+)				

Figure S30

Ducks II, Geese II, Other Waterfowl, Screamers II

a) BayesTraits

b) BAMM−flip

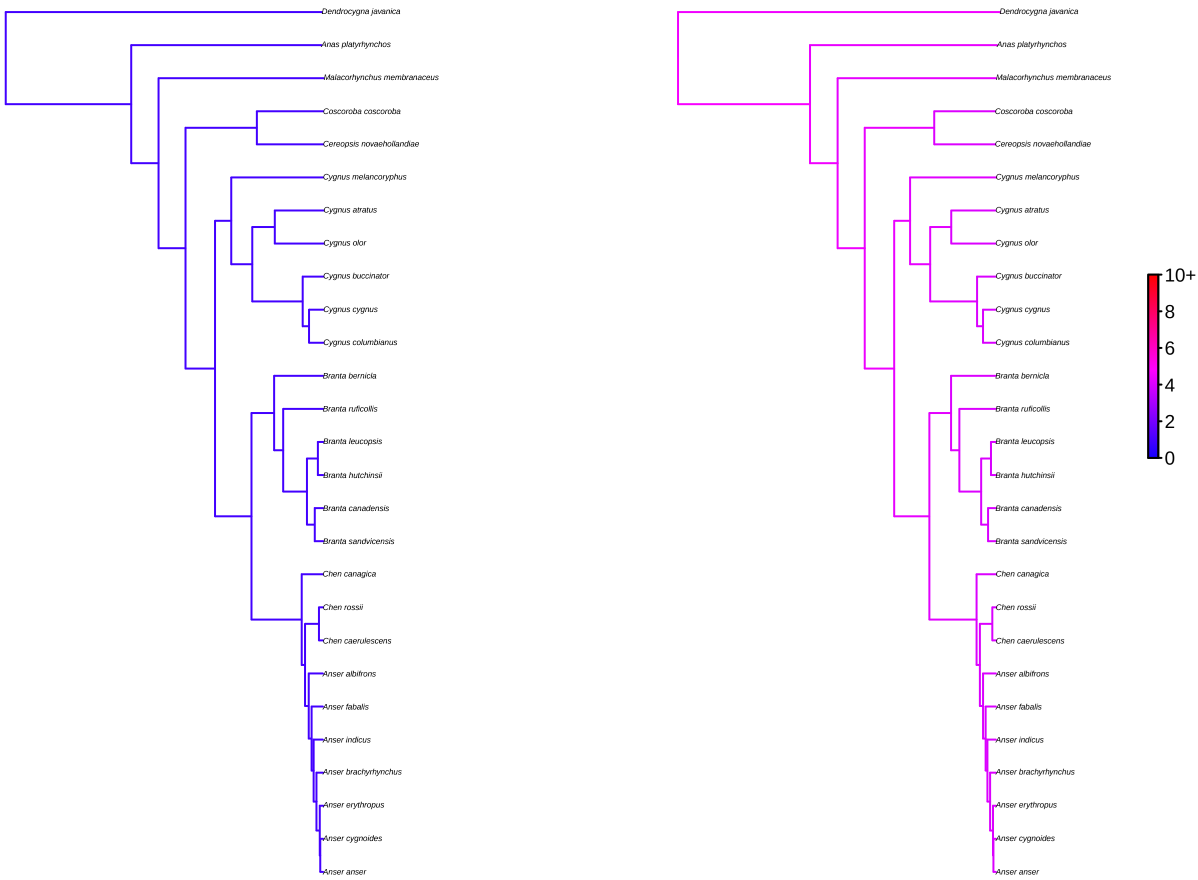

c)

	p_cvar	*p_shgt*	*p_svar*	*p_sasr*	*AICw*	*sigma*	*alpha*	*r*
BM	0.03 (+)	0.75 (−)	0.36 (−)	0.38 (−)	0.45	3.61		
OU	0.07 (+)	0.61 (+)	0.76 (+)	0.33 (+)	0.39	4.57	1.86	
EB	0.02 (+)	0.77 (−)	0.34 (−)	0.43 (−)	0.16	3.61		0
BayesTraits	0.02 (+)	0.72 (−)	0.35 (−)	0.5 (−)				
BAMM−flip	0.03 (+)	0.7 (−)	0.35 (−)	0.42 (−)				

Figure S31

Bustards

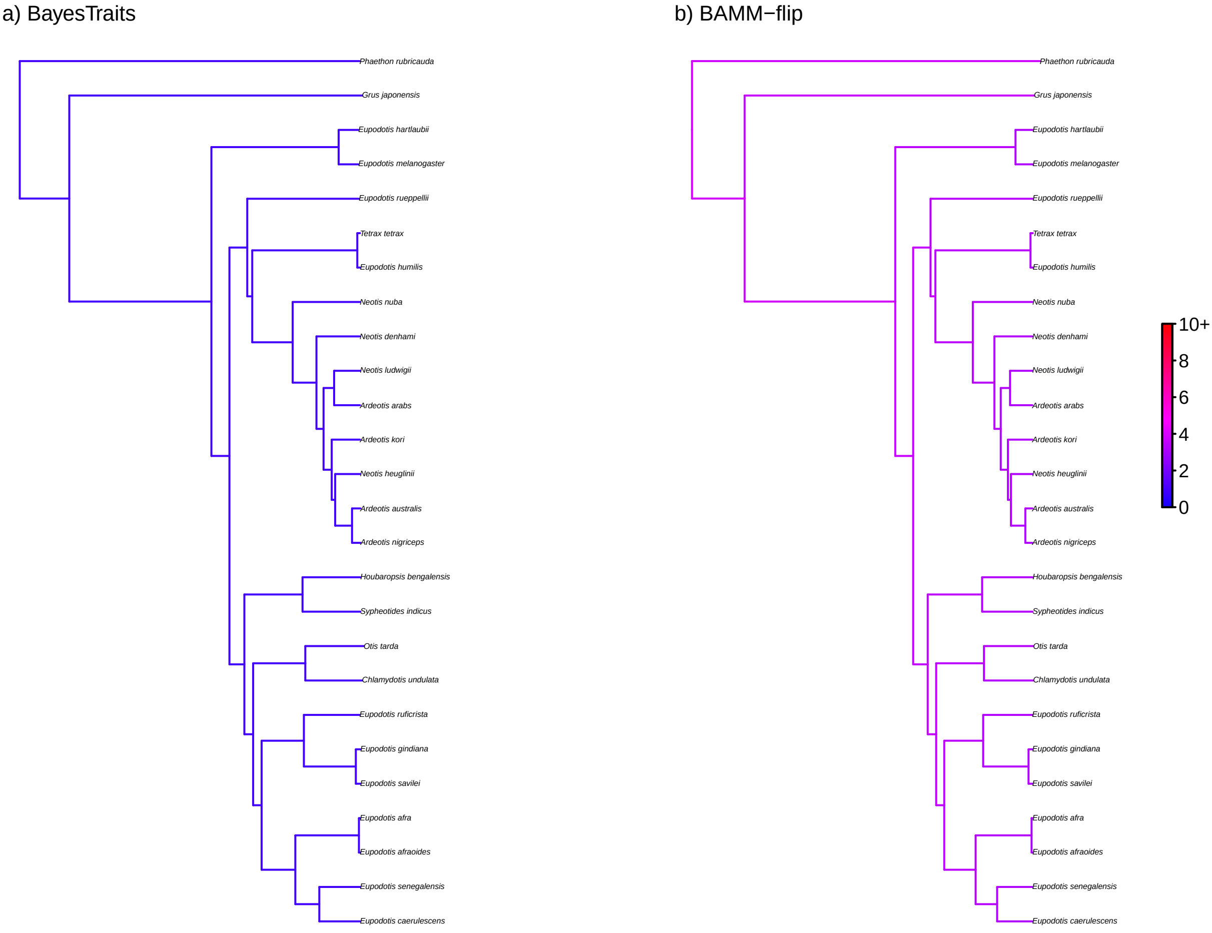

c)

	p_cvar	*p_shgt*	*p_svar*	*p_sasr*	*AICw*	*sigma*	*alpha*	*r*
BM	0.62 (−)	0.82 (−)	0.84 (−)	0.56 (+)	0.51	2.95		
OU	0.58 (+)	0.6 (+)	0.55 (−)	0.6 (−)	0.31	3.99	1.78	
EB	0.58 (−)	0.84 (−)	0.84 (−)	0.52 (+)	0.19	2.95		0
BayesTraits	0.56 (−)	0.84 (−)	0.89 (−)	0.53 (+)				
BAMM−flip	0.56 (−)	0.81 (−)	0.83 (−)	0.53 (+)				

Figure S32

Bee−eaters

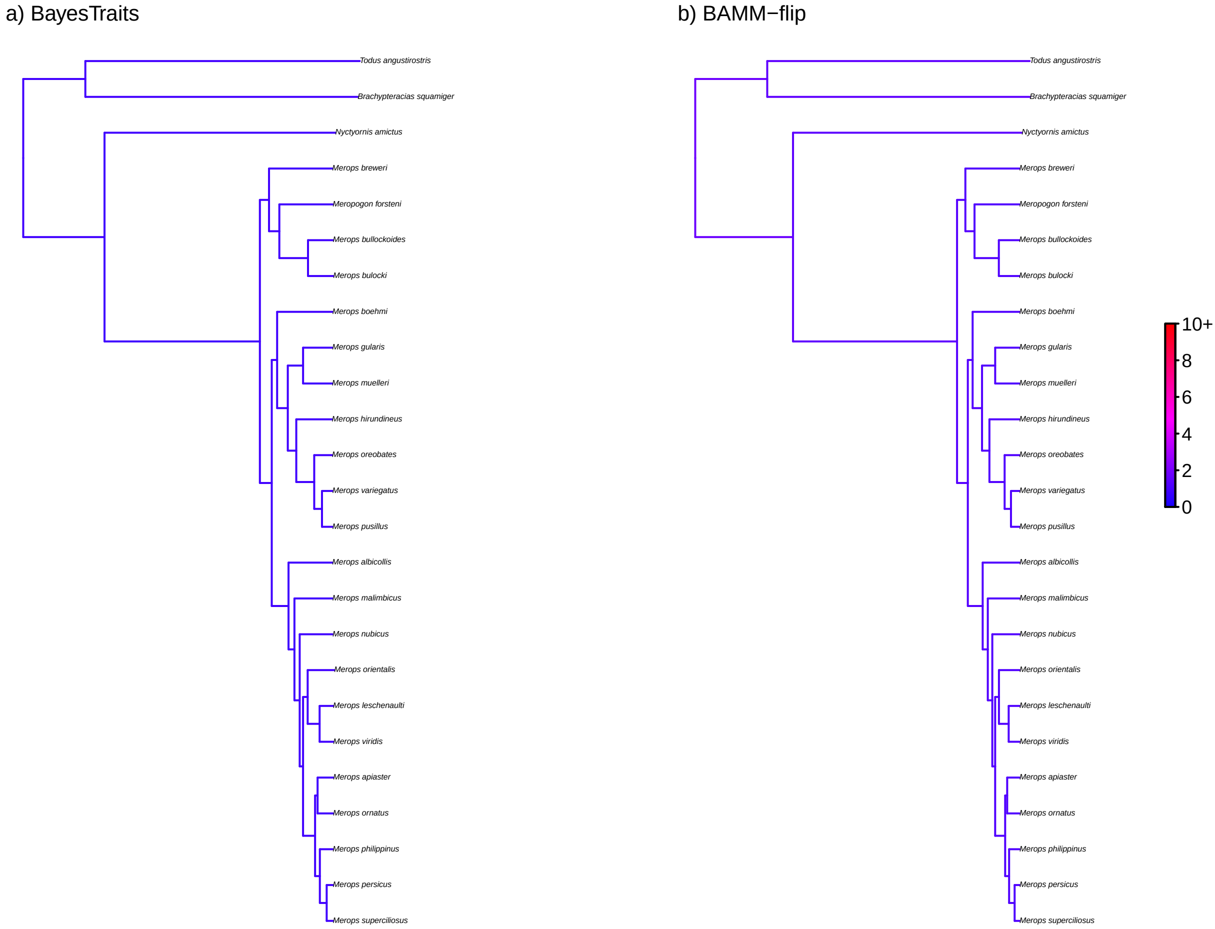

c)

	p_cvar	*p_shgt*	*p_svar*	*p_sasr*	*AICw*	*sigma*	*alpha*	*r*
BM	0.2 (−)	0.97 (−)	0.81 (+)	0.81 (+)	0.57	1.12		
OU	0.19 (−)	0.75 (+)	0.55 (+)	0.76 (+)	0.22	1.21	0.29	
EB	0.18 (−)	1 (−)	0.82 (+)	0.86 (+)	0.21	1.12		0
BayesTraits	0.13 (−)	0.94 (−)	0.86 (+)	0.81 (+)				
BAMM−flip	0.16 (−)	0.91 (−)	0.85 (+)	0.86 (+)				

Figure S33

Ibises

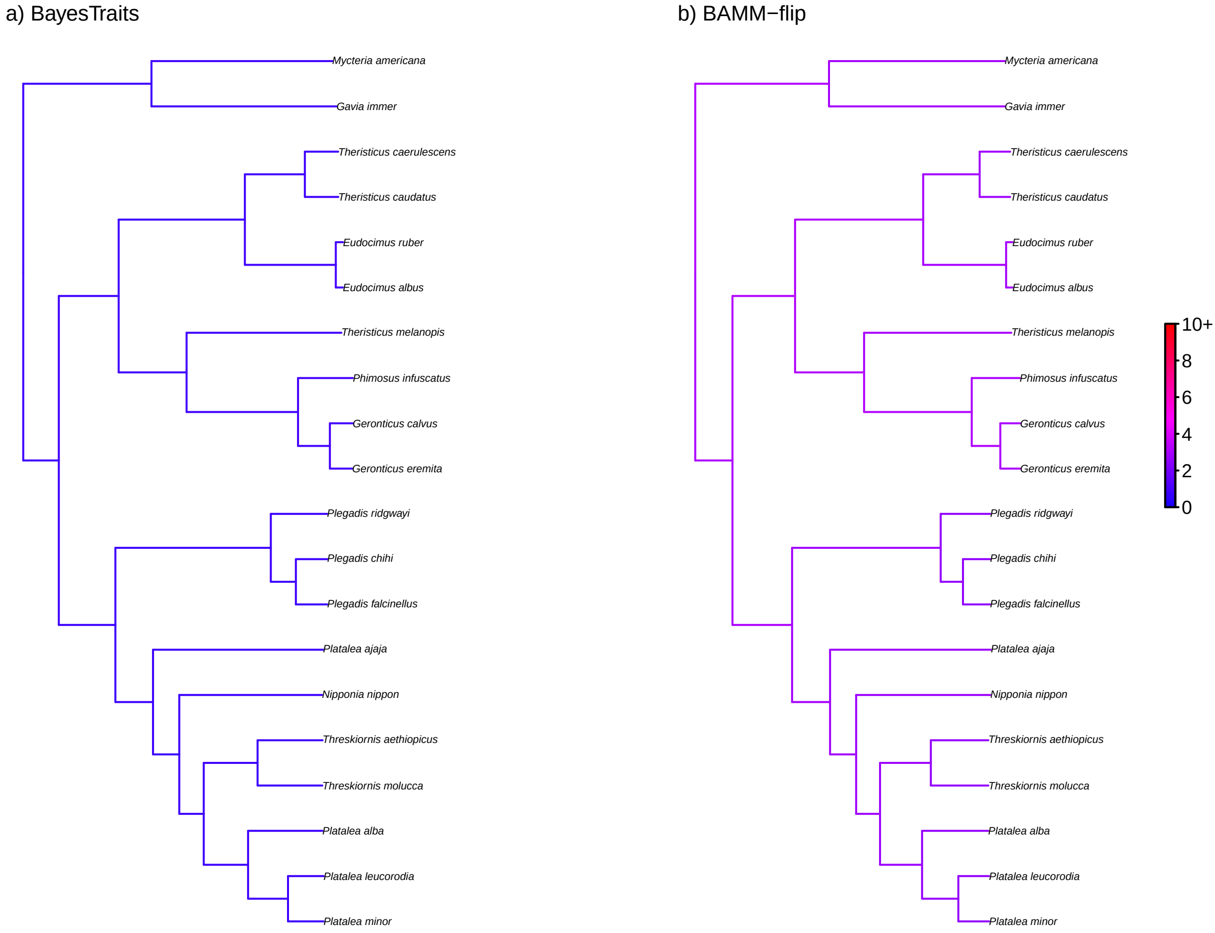

c)

	p_cvar	*p_shgt*	*p_svar*	*p_sasr*	*AICw*	*sigma*	*alpha*	*r*
BM	0.08 (+)	0.31 (−)	0.13 (−)	0.65 (−)	0.44	2.47		
OU	0.06 (+)	0.71 (−)	0.25 (−)	0.96 (−)	0.4	4	5.38	
EB	0.07 (+)	0.27 (−)	0.1 (−)	0.65 (−)	0.16	2.47		0
BayesTraits	0.13 (+)	0.24 (−)	0.19 (−)	0.73 (−)				
BAMM−flip	0.18 (+)	0.15 (−)	0.52 (−)	0.8 (−)				

Figure S34

Trogons

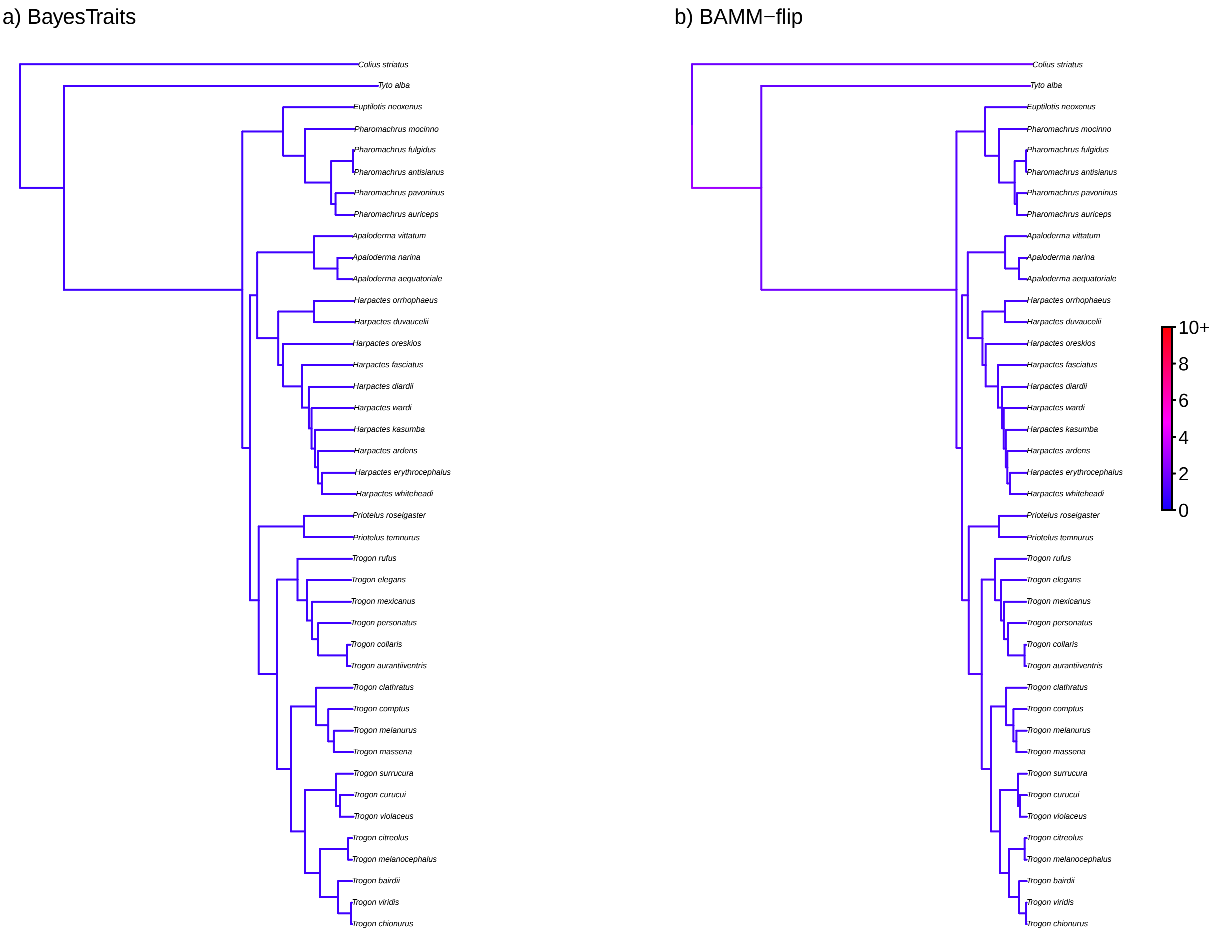

c)

	p_cvar	*p_shgt*	*p_svar*	*p_sasr*	*AICw*	*sigma*	*alpha*	*r*
BM	0.45 (−)	0.03 (+)	0.04 (+)	1 (+)	0.43	1.04		
OU	0.45 (−)	0.03 (+)	0.02 (+)	0.99 (−)	0.16	1.04	0	
EB	0.22 (+)	0.53 (−)	0.42 (−)	0.74 (+)	0.41	7.01		−4.85
BayesTraits	0.44 (−)	0.04 (+)	0.03 (+)	0.95 (−)				
BAMM−flip	0.39 (−)	0.04 (+)	0.04 (+)	0.85 (−)				

Figure S35

Whistlers, Allies

a) BayesTraits

b) BAMM−flip

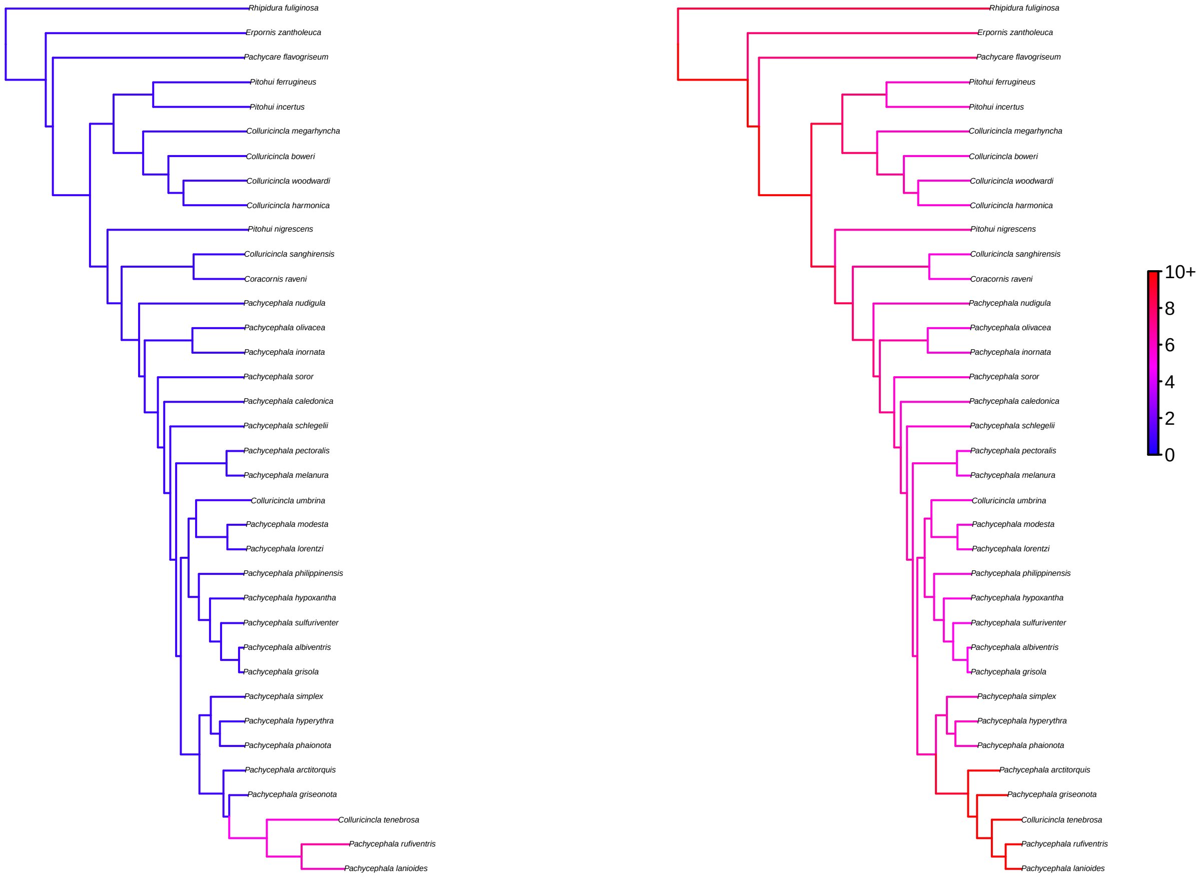

c)

	p_cvar	p_shgt	p_svar	p_sasr	AICw	sigma	alpha	r
BM	0.01 (+)	0.58 (+)	0.82 (−)	0.5 (+)	0.54	7.88		
OU	0.03 (+)	0.36 (+)	0.95 (−)	0.45 (+)	0.26	9.29	5.91	
EB	0.02 (−)	0.55 (+)	0.83 (+)	0.55 (−)	0.2	7.88		0
BayesTraits	0.23 (+)	0.04 (+)	0 (+)	0.53 (+)				
BAMM−flip	0.38 (+)	0.14 (+)	0.01 (+)	0.44 (+)				

Figure S36

Manakins

a) BayesTraits

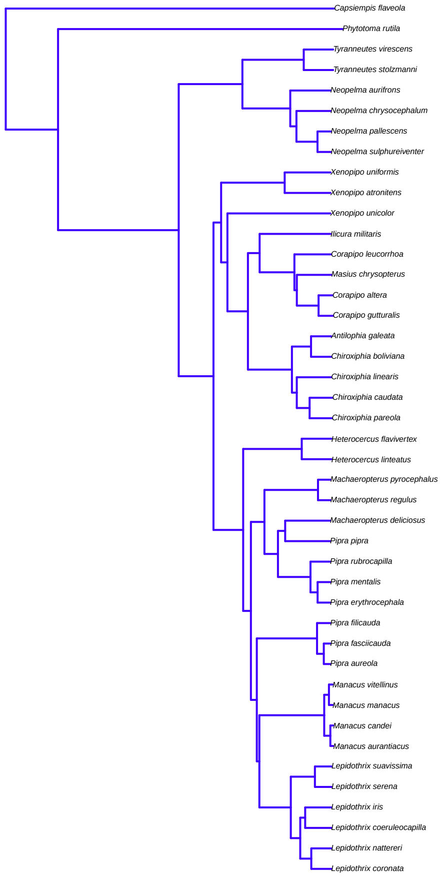

b) BAMM−flip

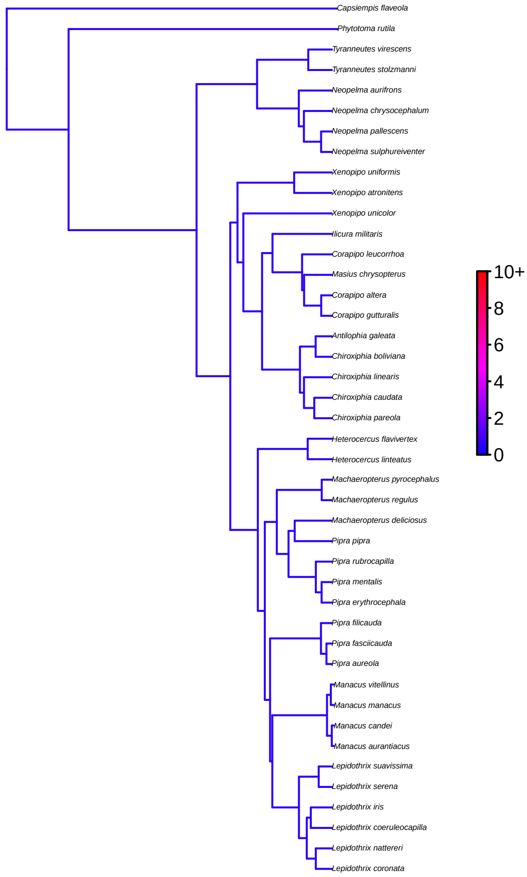

c)

	p_cvar	*p_shgt*	*p_svar*	*p_sasr*	*AICw*	*sigma*	*alpha*	*r*
BM	0.1 (+)	0.24 (+)	0.43 (+)	0.35 (+)	0.57	0.47		
OU	0.11 (+)	0.14 (+)	0.27 (+)	0.36 (+)	0.22	0.5	0.32	
EB	0.1 (+)	0.29 (+)	0.46 (+)	0.36 (−)	0.21	0.47		0
BayesTraits	0.12 (+)	0.24 (+)	0.32 (+)	0.4 (+)				
BAMM−flip	0.11 (+)	0.31 (+)	0.42 (+)	0.31 (+)				

Figure S37

Broadbills, Pittas, Asities, Allies

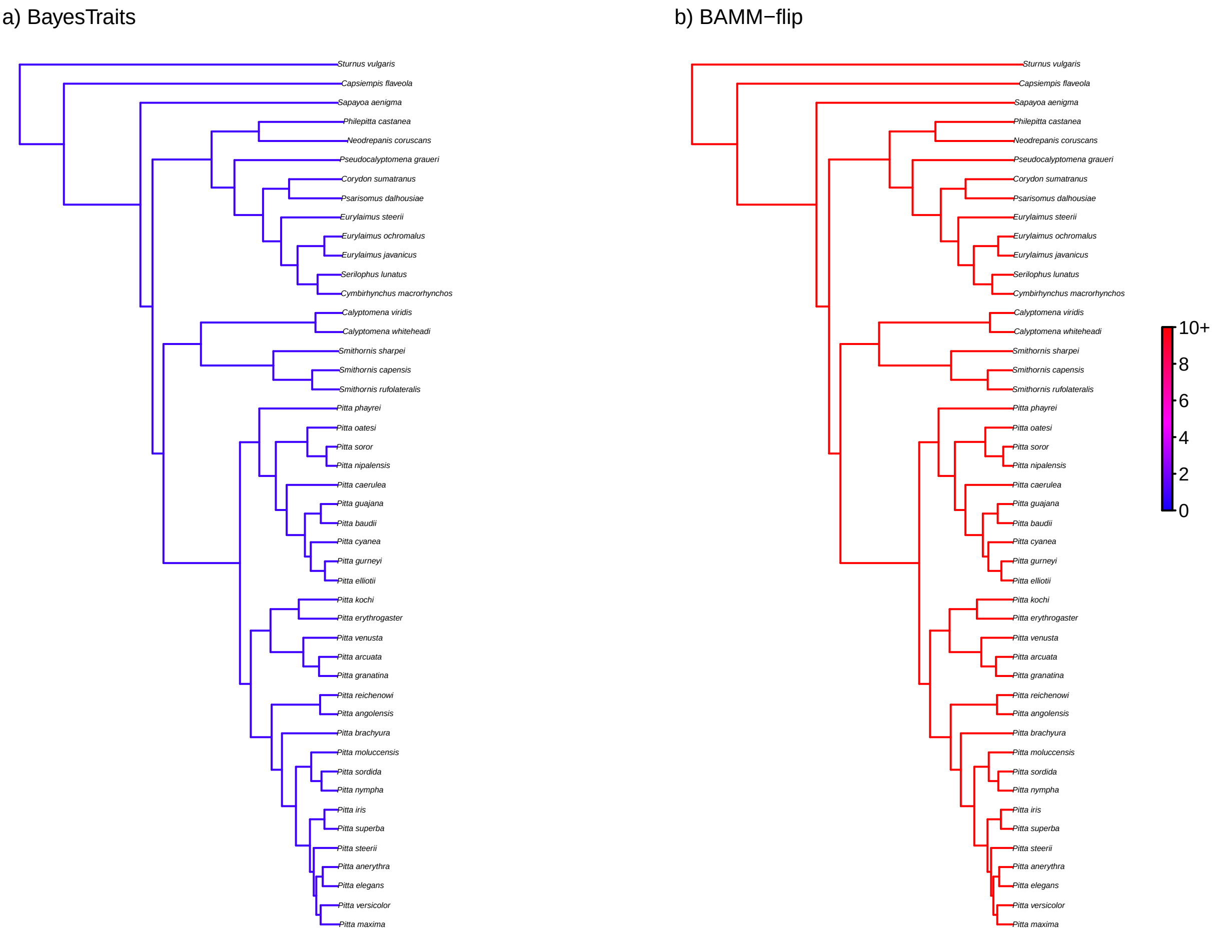

c)

	p_cvar	p_shgt	p_svar	p_sasr	AICw	sigma	alpha	r
BM	0.68 (−)	0.9 (+)	0.76 (+)	0.14 (−)	0.49	10.84		
OU	0.57 (−)	0.29 (+)	0.19 (+)	0.04 (−)	0.34	13.57	7.22	
EB	0.64 (+)	0.89 (+)	0.77 (+)	0.15 (+)	0.18	10.84		0
BayesTraits	0.61 (−)	0.92 (+)	0.66 (+)	0.13 (−)				
BAMM−flip	0.59 (−)	0.98 (−)	0.74 (+)	0.19 (−)				

Figure S38

Tapaculos

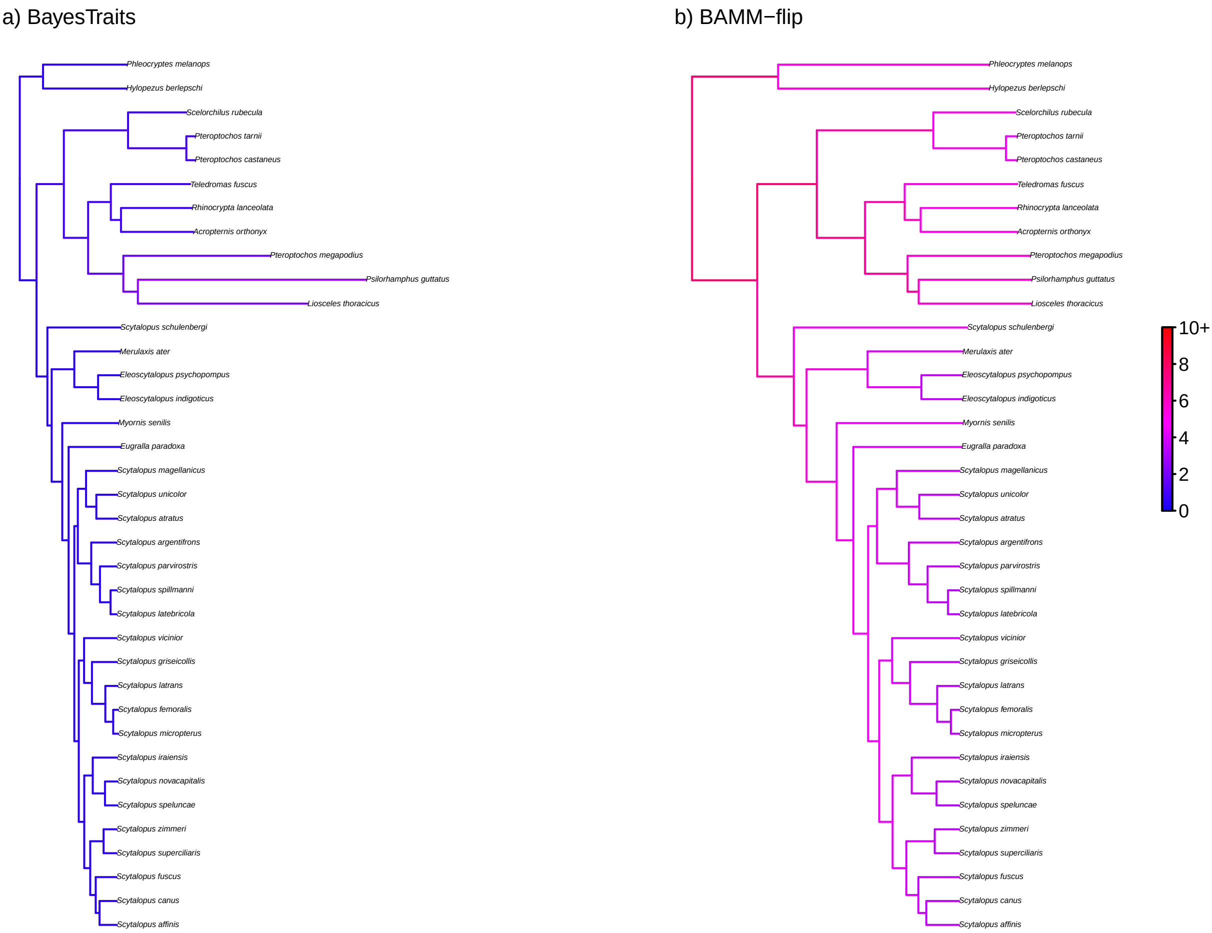

c)

	p_cvar	*p_shgt*	*p_svar*	*p_sasr*	*AICw*	*sigma*	*alpha*	*r*
BM	0.39 (+)	0.28 (+)	0.06 (+)	0.17 (+)	0.56	4.48		
OU	0.39 (+)	0.32 (+)	0.05 (+)	0.15 (+)	0.21	4.48	0	
EB	0.44 (−)	0.63 (+)	0.18 (+)	0.12 (−)	0.23	8.38		−7.38
BayesTraits	0.83 (−)	0.83 (−)	0.09 (+)	0.55 (+)				
BAMM−flip	0.68 (−)	0.62 (−)	0.11 (+)	0.68 (+)				

Figure S39

Hornbills

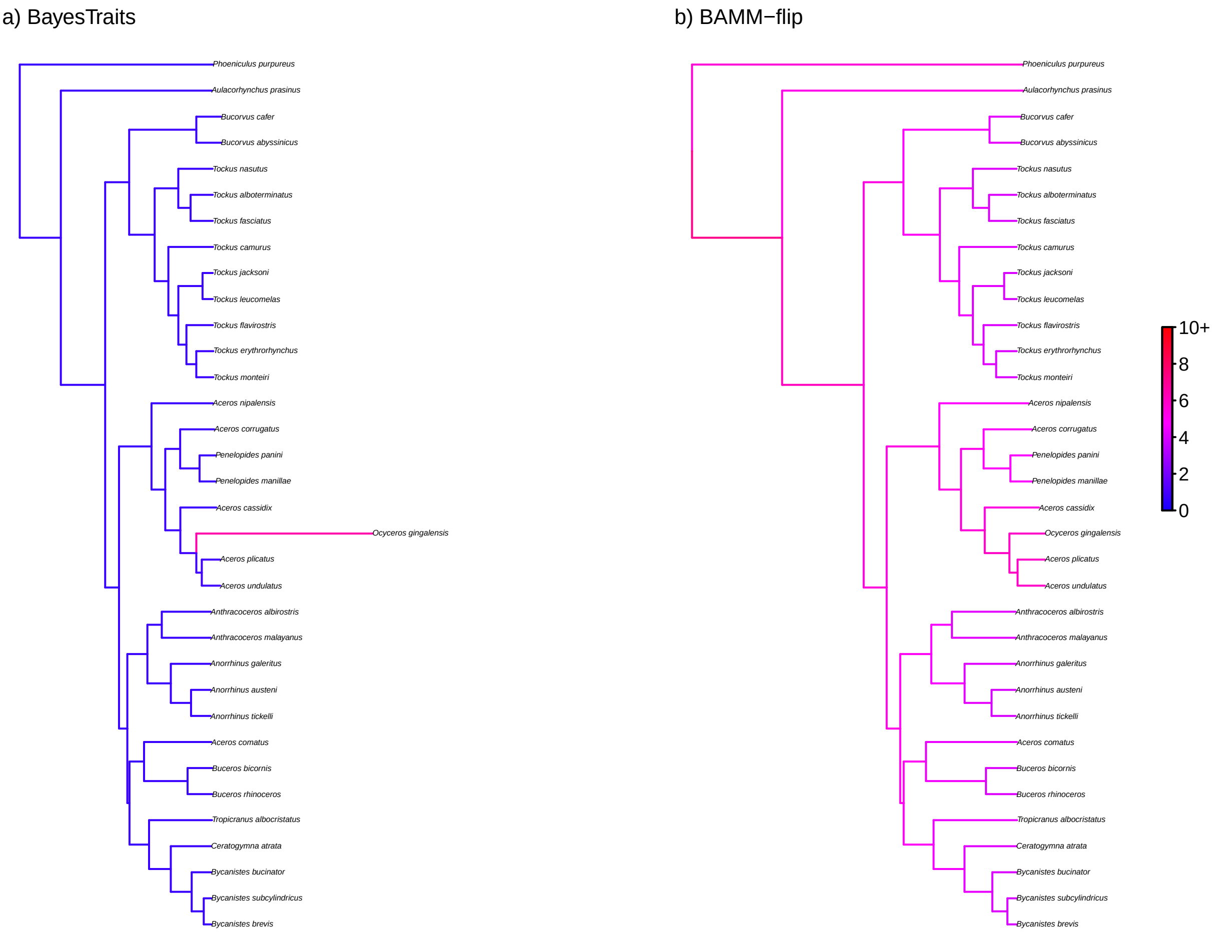

c)

	p_cvar	*p_shgt*	*p_svar*	*p_sasr*	*AICw*	*sigma*	*alpha*	*r*
BM	0 (+)	0.24 (+)	0.19 (+)	0.97 (−)	0.57	4.6		
OU	0 (+)	0.18 (+)	0.14 (+)	0.9 (−)	0.22	4.87	0.37	
EB	0 (+)	0.25 (+)	0.19 (+)	0.93 (+)	0.21	4.6		0
BayesTraits	0.09 (+)	0.04 (+)	0 (+)	0.81 (−)				
BAMM−flip	0.02 (+)	0.18 (+)	0.02 (+)	0.91 (−)				

Figure S40

Herons

a) BayesTraits b) BAMM−flip

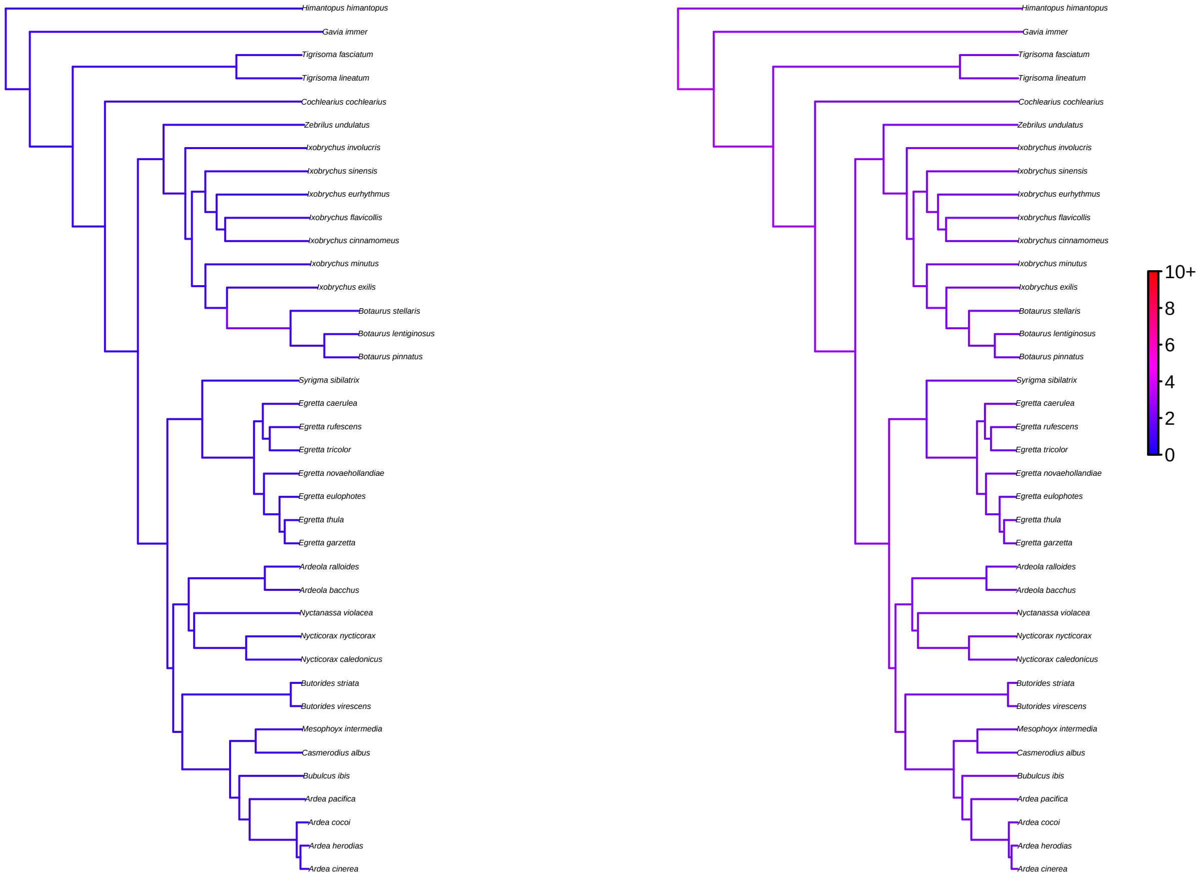

c)

	p_cvar	*p_shgt*	*p_svar*	*p_sasr*	*AICw*	*sigma*	*alpha*	*r*
BM	0.4 (+)	0.21 (+)	0.23 (+)	0.59 (+)	0.57	1.94		
OU	0.35 (+)	0.21 (+)	0.23 (+)	0.52 (+)	0.21	1.94	0	
EB	0.31 (+)	0.27 (−)	0.28 (−)	0.49 (+)	0.22	2.79		−0.56
BayesTraits	0.43 (+)	0.17 (+)	0.09 (+)	0.55 (+)				
BAMM−flip	0.42 (+)	0.25 (+)	0.24 (+)	0.55 (+)				

Figure S41

Thornbills, Gerygones

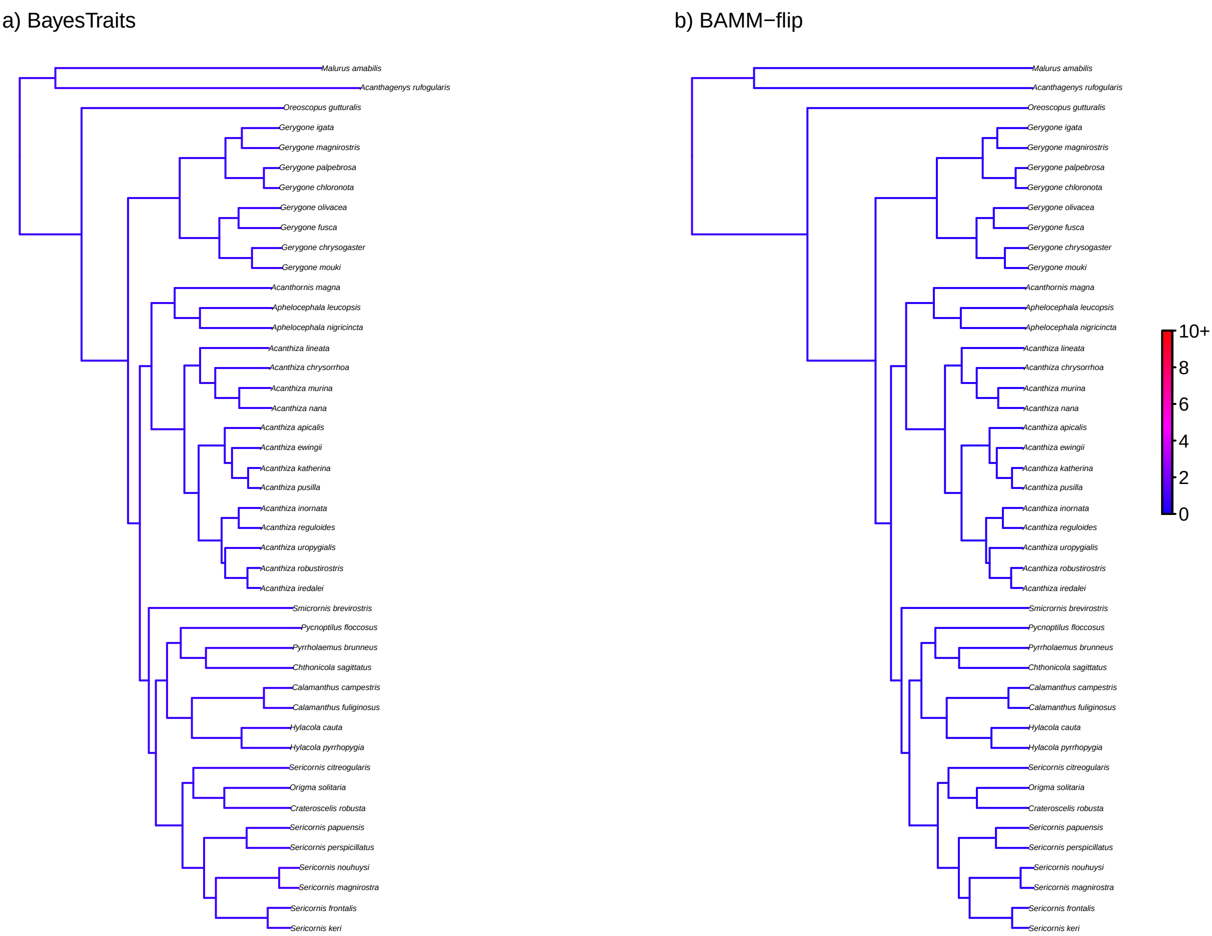

c)

	p_cvar	*p_shgt*	*p_svar*	*p_sasr*	*AICw*	*sigma*	*alpha*	*r*
BM	0.03 (+)	0.04 (+)	0.11 (+)	0.05 (+)	0.51	0.37		
OU	0.05 (+)	0.02 (+)	0.1 (+)	0.05 (+)	0.19	0.37	0	
EB	0.04 (+)	0.15 (+)	0.29 (+)	0.05 (+)	0.31	1		−1.49
BayesTraits	0.12 (+)	0.07 (+)	0.07 (+)	0.08 (+)				
BAMM−flip	0.11 (+)	0.06 (+)	0.09 (+)	0.07 (+)				

Figure S42

Falcons, Caracaras

a) BayesTraits

b) BAMM−flip

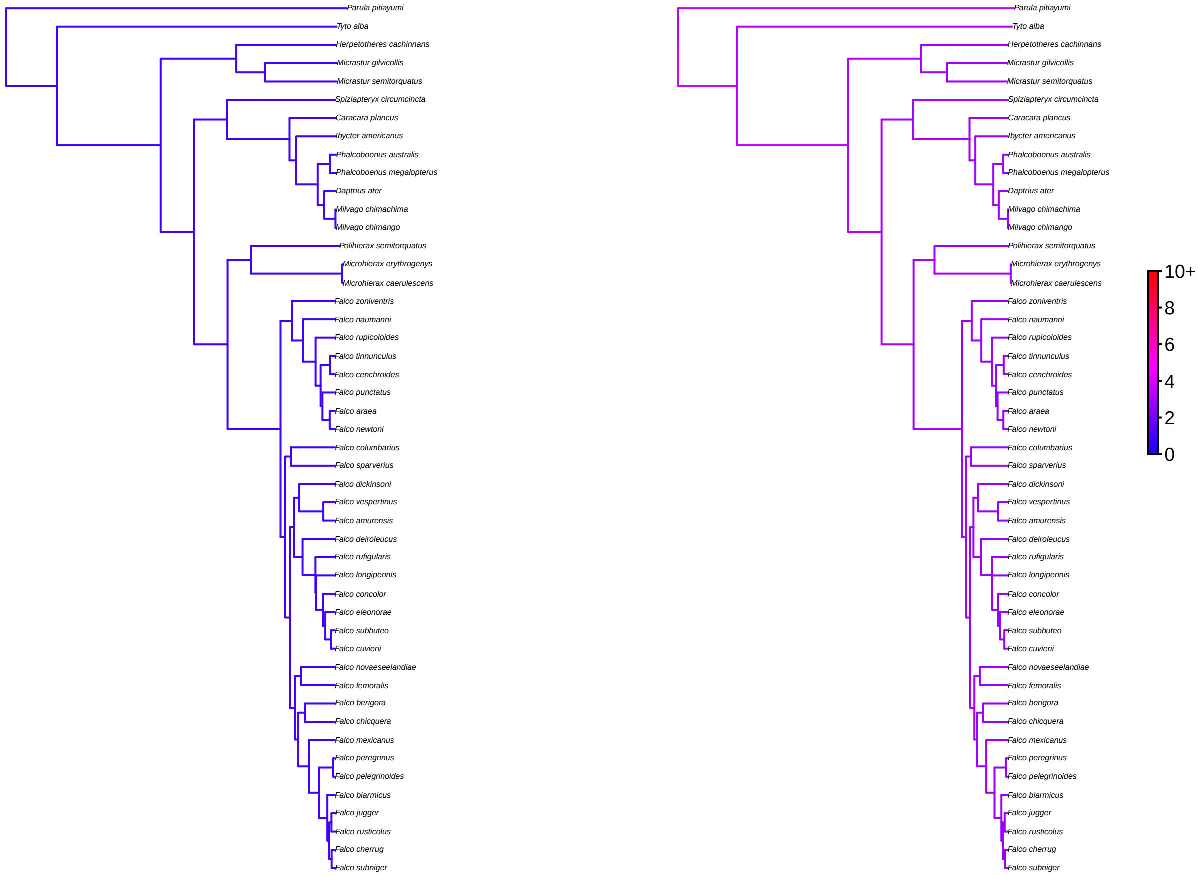

c)

	p_cvar	p_shgt	p_svar	p_sasr	AICw	sigma	alpha	r
BM	0.93 (+)	0.84 (+)	0.48 (−)	0.47 (−)	0.56	2.61		
OU	0.9 (+)	0.61 (+)	0.64 (−)	0.43 (−)	0.24	2.8	0.32	
EB	0.87 (+)	0.87 (+)	0.48 (+)	0.52 (+)	0.21	2.61		0
BayesTraits	0.97 (−)	0.79 (+)	0.59 (−)	0.56 (−)				
BAMM−flip	1 (−)	0.88 (+)	0.5 (−)	0.62 (−)				

Figure S43

Cotingas

a) BayesTraits

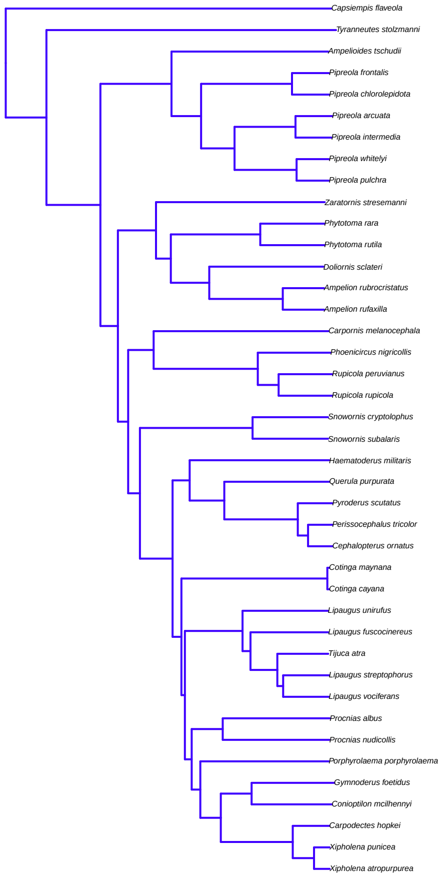

b) BAMM−flip

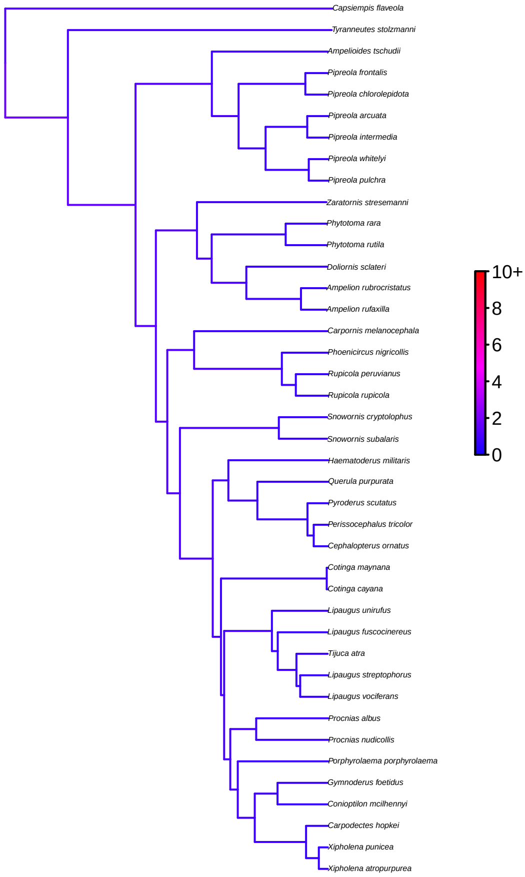

c)

	p_cvar	*p_shgt*	*p_svar*	*p_sasr*	*AICw*	*sigma*	*alpha*	*r*
BM	0.97 (+)	0.09 (+)	0.22 (+)	0.49 (−)	0.45	0.8		
OU	0.95 (−)	0.1 (+)	0.25 (+)	0.51 (−)	0.16	0.8	0	
EB	0.8 (+)	0.46 (+)	0.64 (−)	0.69 (−)	0.39	2.43		−1.51
BayesTraits	1 (−)	0.06 (+)	0.17 (+)	0.46 (−)				
BAMM−flip	0.93 (−)	0.12 (+)	0.21 (+)	0.54 (−)				

Figure S44

Swallows, Martins

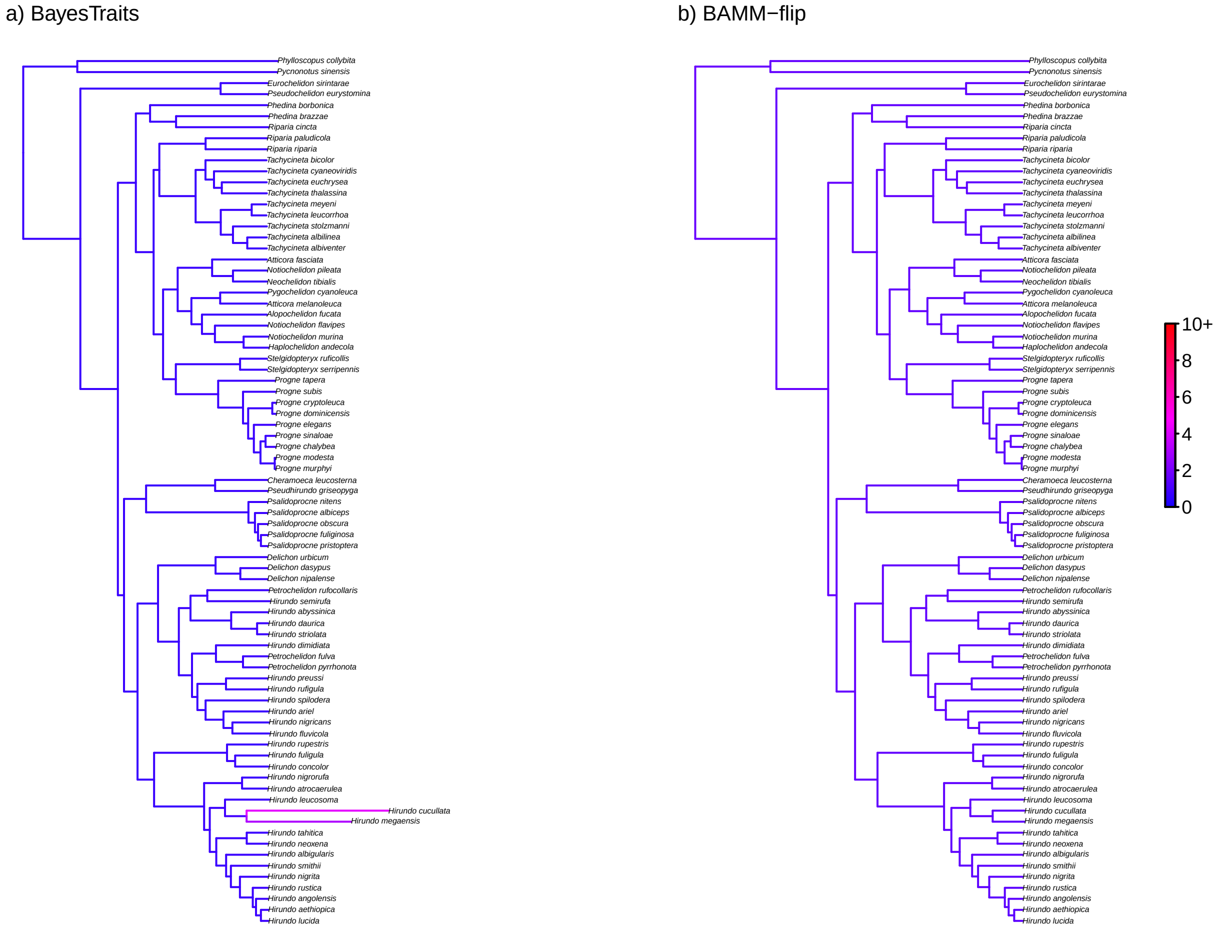

c)

	p_cvar	*p_shgt*	*p_svar*	*p_sasr*	*AICw*	*sigma*	*alpha*	*r*
BM	0.05 (+)	0.83 (−)	0.59 (+)	0.76 (+)	0.53	1.45		
OU	0.03 (+)	0.61 (+)	0.2 (+)	0.87 (+)	0.27	1.7	2.64	
EB	0.04 (+)	0.79 (+)	0.61 (−)	0.84 (+)	0.2	1.45		0
BayesTraits	0.36 (+)	0.98 (−)	0.26 (+)	0.81 (+)				
BAMM−flip	0.07 (+)	0.83 (−)	0.51 (+)	0.8 (+)				

Figure S45

Nightjars

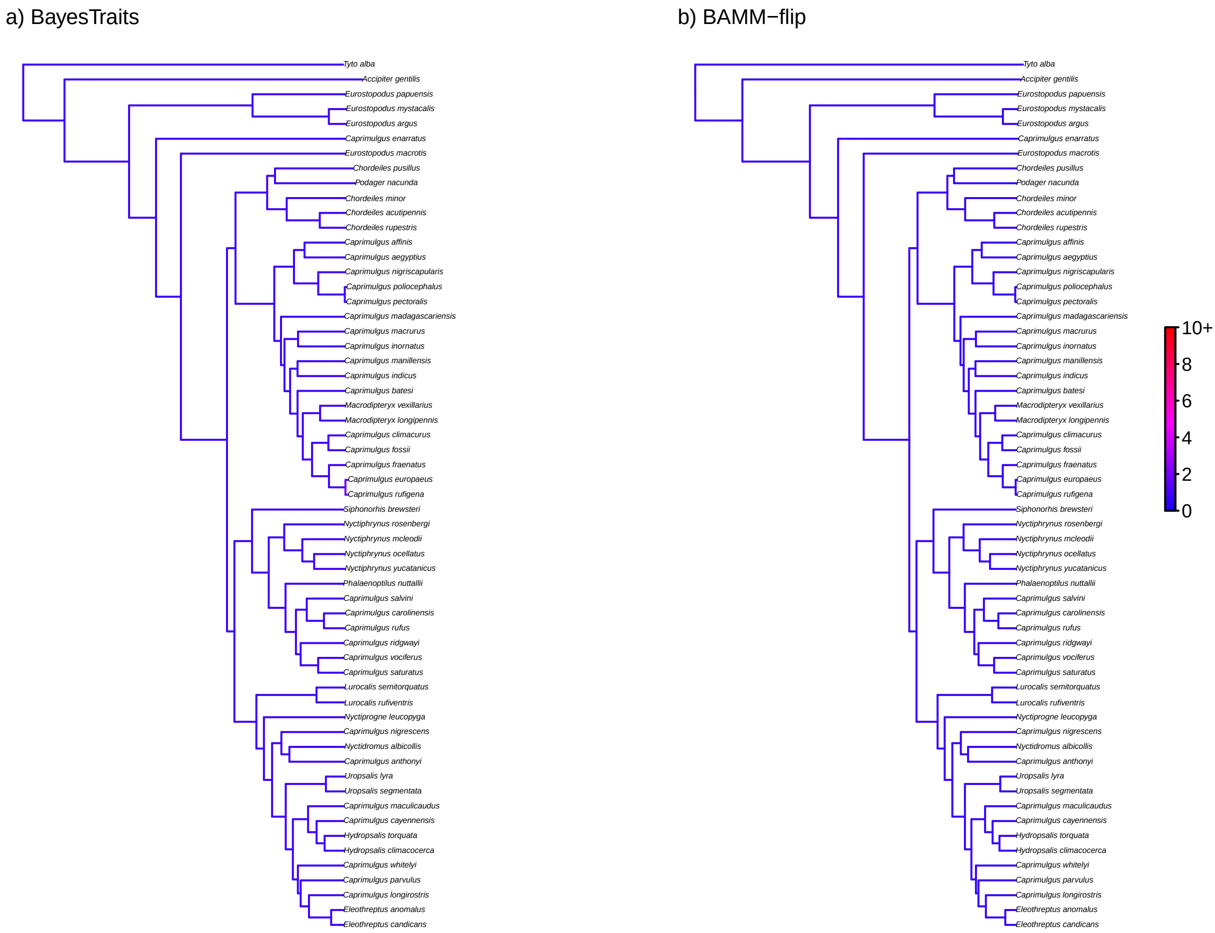

c)

	p_cvar	p_shgt	p_svar	p_sasr	AICw	sigma	alpha	r
BM	0.13 (+)	0.74 (+)	0.7 (−)	0.06 (+)	0.57	0.65		
OU	0.11 (+)	0.62 (+)	0.87 (−)	0.03 (+)	0.22	0.67	0.12	
EB	0.1 (+)	0.76 (−)	0.73 (+)	0.05 (+)	0.21	0.65		0
BayesTraits	0.47 (+)	0.54 (+)	0.7 (+)	0.08 (+)				
BAMM−flip	0.15 (+)	0.74 (+)	0.73 (−)	0.04 (+)				

Figure S46

Waders, Allies

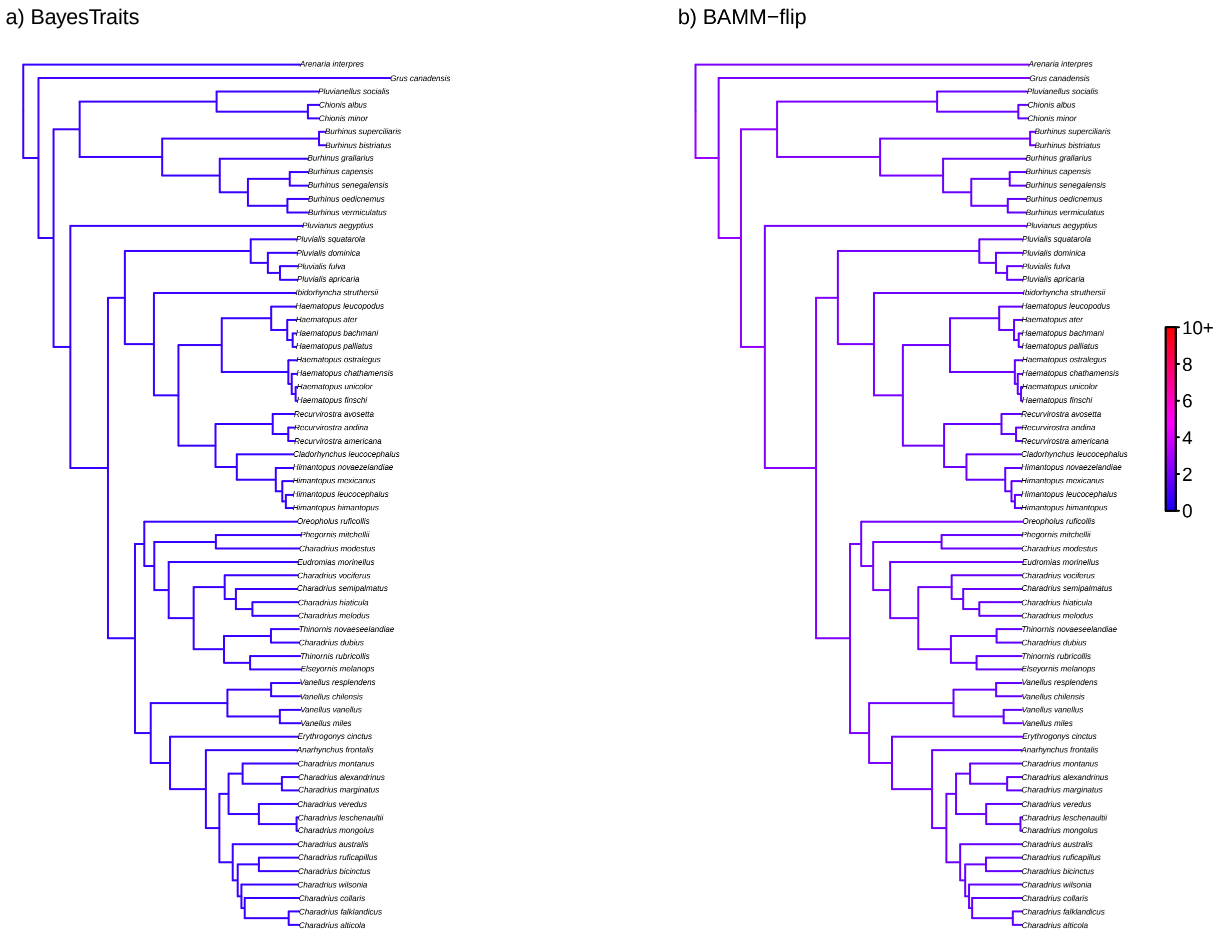

c)

	p_cvar	*p_shgt*	*p_svar*	*p_sasr*	*AICw*	*sigma*	*alpha*	*r*
BM	0.19 (+)	0.33 (+)	0.87 (−)	0.52 (+)	0.57	1.65		
OU	0.19 (+)	0.37 (+)	0.88 (−)	0.46 (+)	0.21	1.65	0	
EB	0.2 (−)	0.48 (+)	0.75 (+)	0.45 (+)	0.23	2.22		−0.52
BayesTraits	0.37 (+)	0.51 (+)	0.89 (+)	0.6 (+)				
BAMM−flip	0.28 (+)	0.52 (+)	0.95 (−)	0.57 (+)				

Figure S47

New World Blackbirds

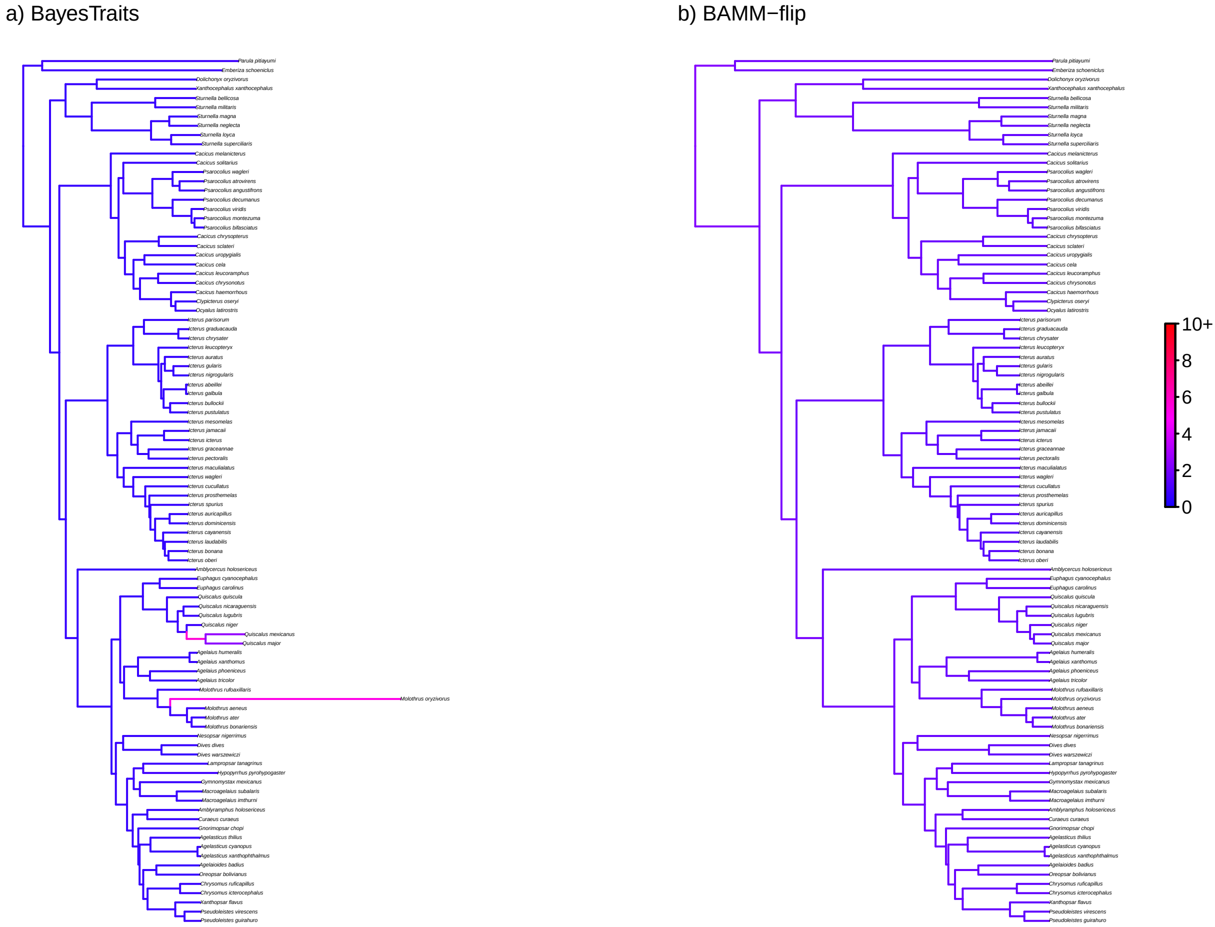

c)

	p_cvar	*p_shgt*	*p_svar*	*p_sasr*	*AICw*	*sigma*	*alpha*	*r*
BM	0.04 (+)	0.41 (+)	0.89 (−)	0.07 (+)	0.57	1.5		
OU	0.06 (+)	0.42 (+)	0.94 (−)	0.06 (+)	0.21	1.5	0	
EB	0.06 (+)	0.5 (+)	0.91 (−)	0.06 (+)	0.21	1.72		−0.44
BayesTraits	0.83 (+)	0.24 (+)	0.19 (+)	0.18 (+)				
BAMM−flip	0.17 (+)	0.96 (+)	0.68 (+)	0.12 (+)				

Figure S48

Swifts, Treeswifts

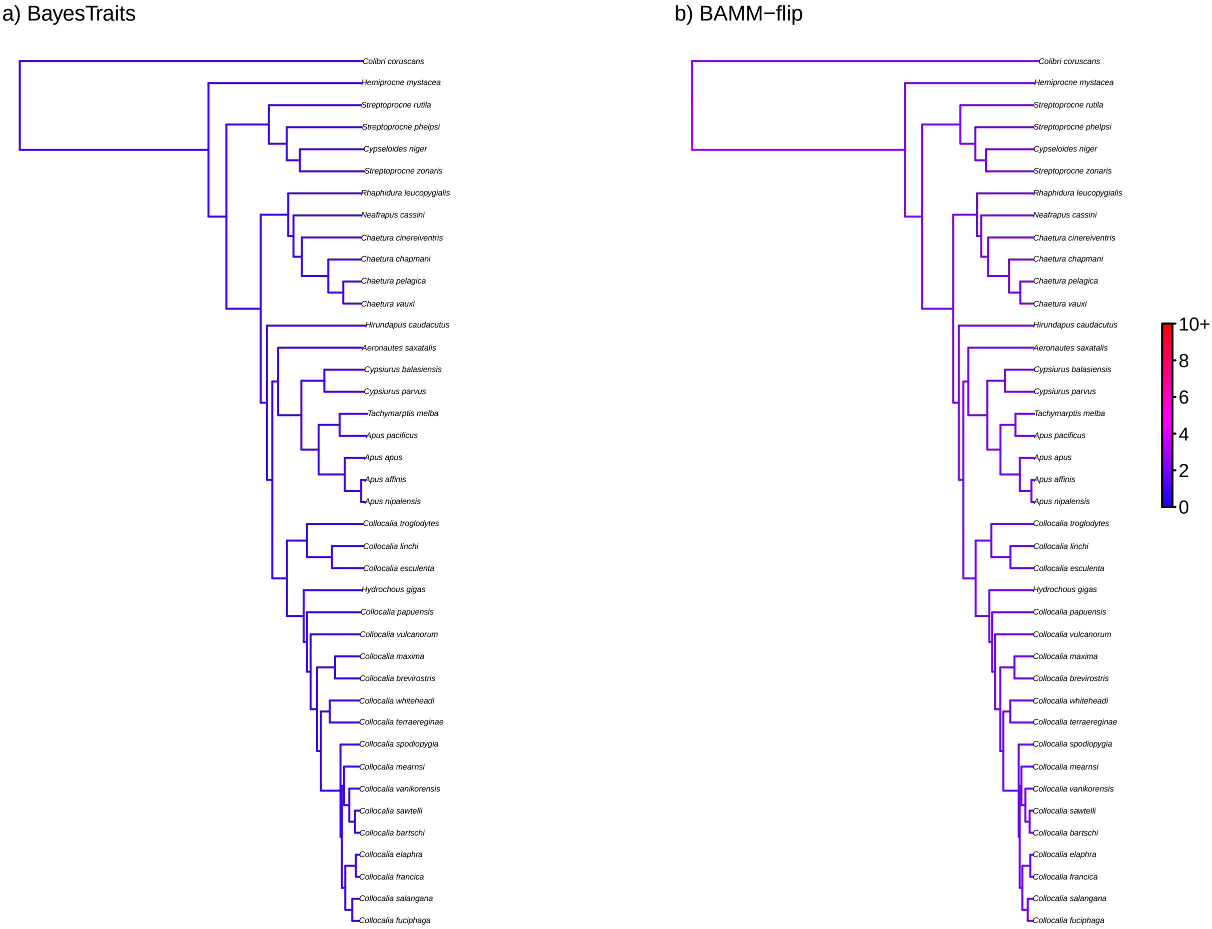

c)

	p_cvar	*p_shgt*	*p_svar*	*p_sasr*	*AICw*	*sigma*	*alpha*	*r*
BM	0.36 (−)	0.4 (+)	0.48 (+)	0.05 (+)	0.58	1.8		
OU	0.37 (−)	0.38 (+)	0.41 (+)	0.04 (+)	0.21	1.81	0.02	
EB	0.37 (+)	0.44 (+)	0.53 (−)	0.05 (+)	0.21	1.8		0
BayesTraits	0.29 (−)	0.44 (+)	0.43 (+)	0.05 (+)				
BAMM−flip	0.33 (−)	0.43 (+)	0.46 (+)	0.04 (+)				

Figure S49

Toucans, Barbets

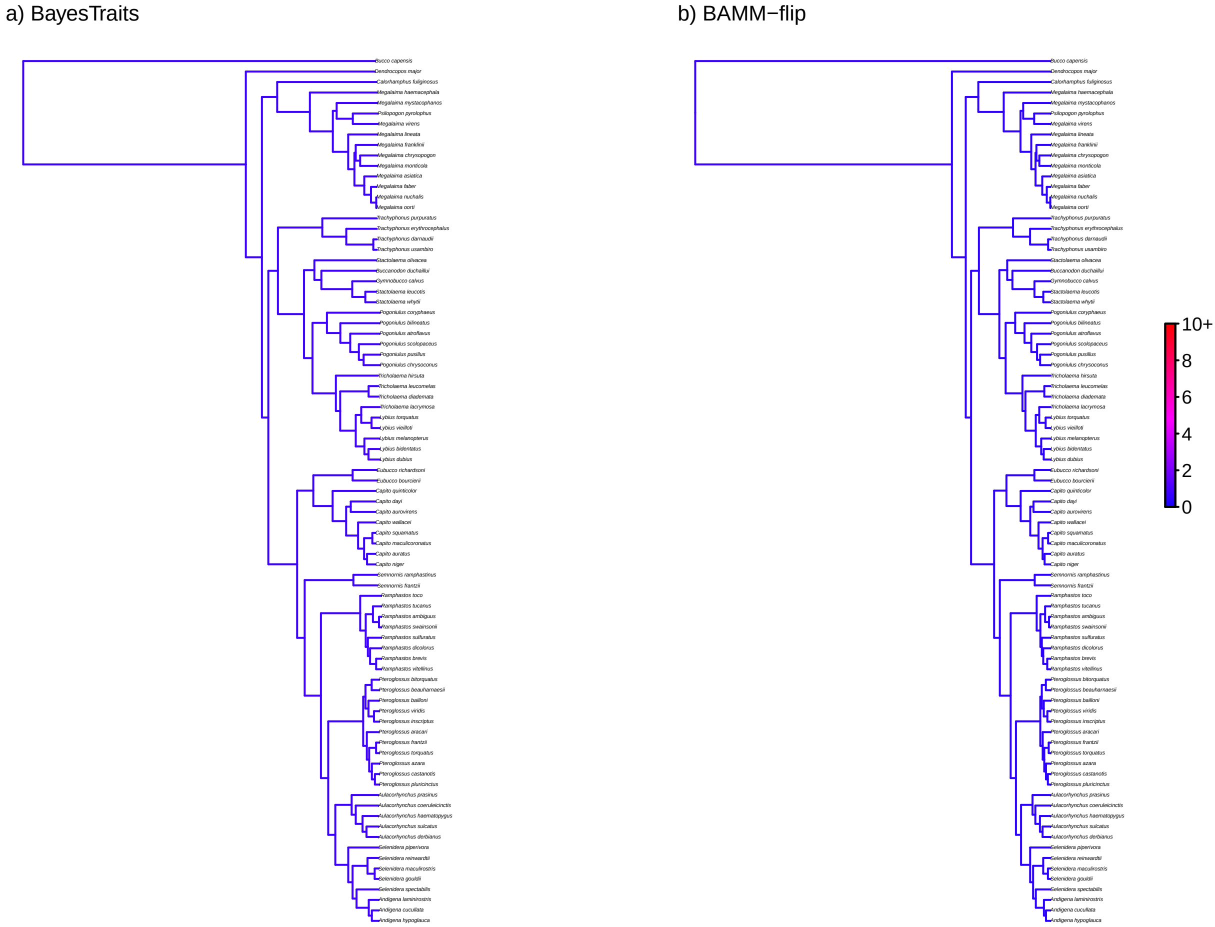

c)

	p_cvar	p_shgt	p_svar	p_sasr	AICw	sigma	alpha	r
BM	0.01 (+)	0.58 (+)	0.48 (+)	0.99 (+)	0.56	0.5		
OU	0 (+)	0.58 (+)	0.49 (+)	0.99 (−)	0.21	0.5	0	
EB	0.01 (+)	0.91 (−)	0.82 (−)	1 (+)	0.24	1.46		−0.37
BayesTraits	0.01 (+)	0.63 (+)	0.45 (+)	0.98 (−)				
BAMM−flip	0.01 (+)	0.69 (+)	0.55 (+)	0.94 (−)				

Figure S50

Wrens, Gnatcatchers, Nuthatches, Wallcreeper, Treecreepers

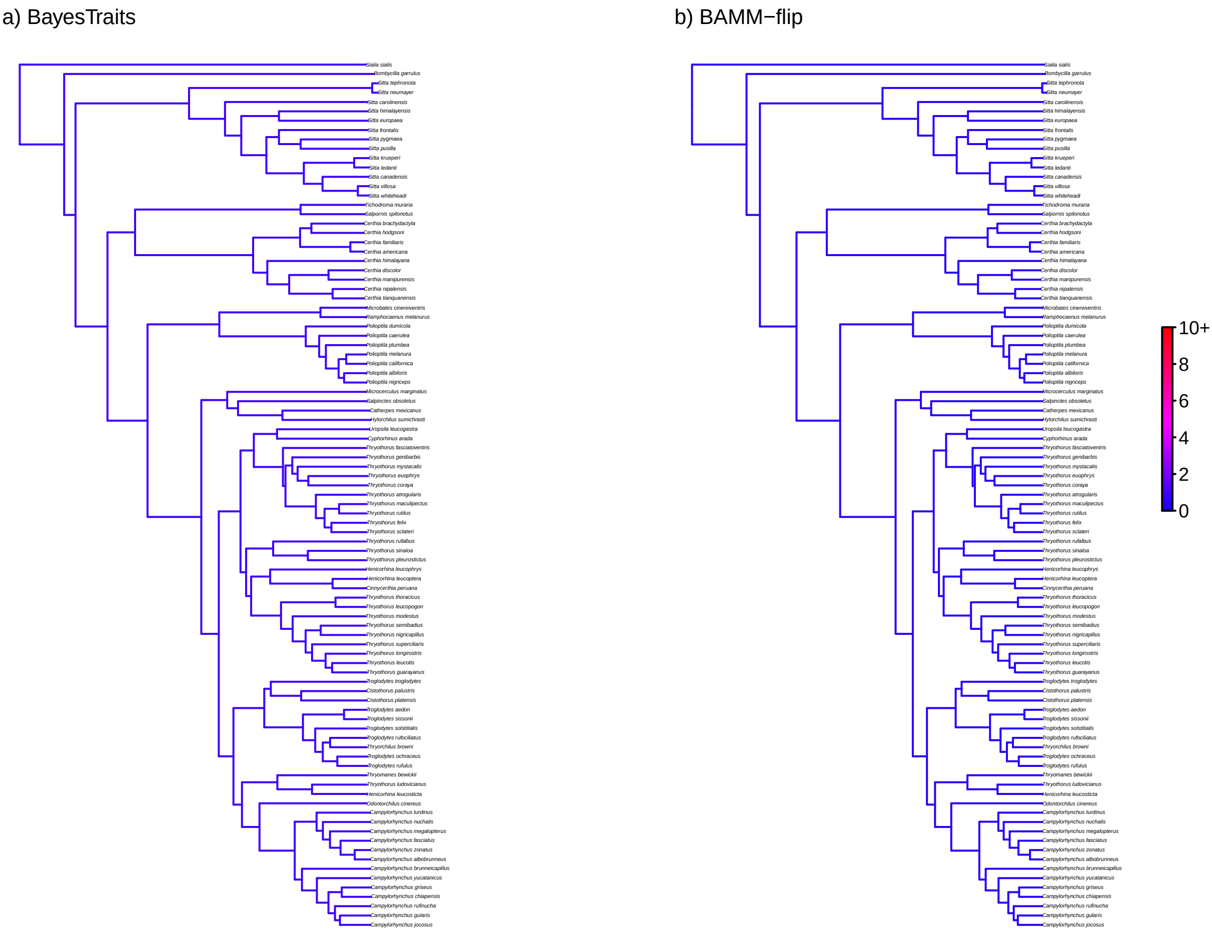

c)

	p_cvar	p_shgt	p_svar	p_sasr	AICw	sigma	alpha	r
BM	0.18 (+)	0.56 (+)	0.66 (+)	0.18 (+)	0.58	0.35		
OU	0.16 (+)	0.54 (+)	0.62 (+)	0.17 (+)	0.21	0.35	0	
EB	0.15 (+)	0.67 (−)	0.74 (−)	0.18 (+)	0.21	0.39		−0.11
BayesTraits	0.21 (+)	0.58 (+)	0.52 (+)	0.21 (+)				
BAMM−flip	0.17 (+)	0.59 (+)	0.6 (+)	0.16 (+)				

Figure S51

Rails, Finfoots

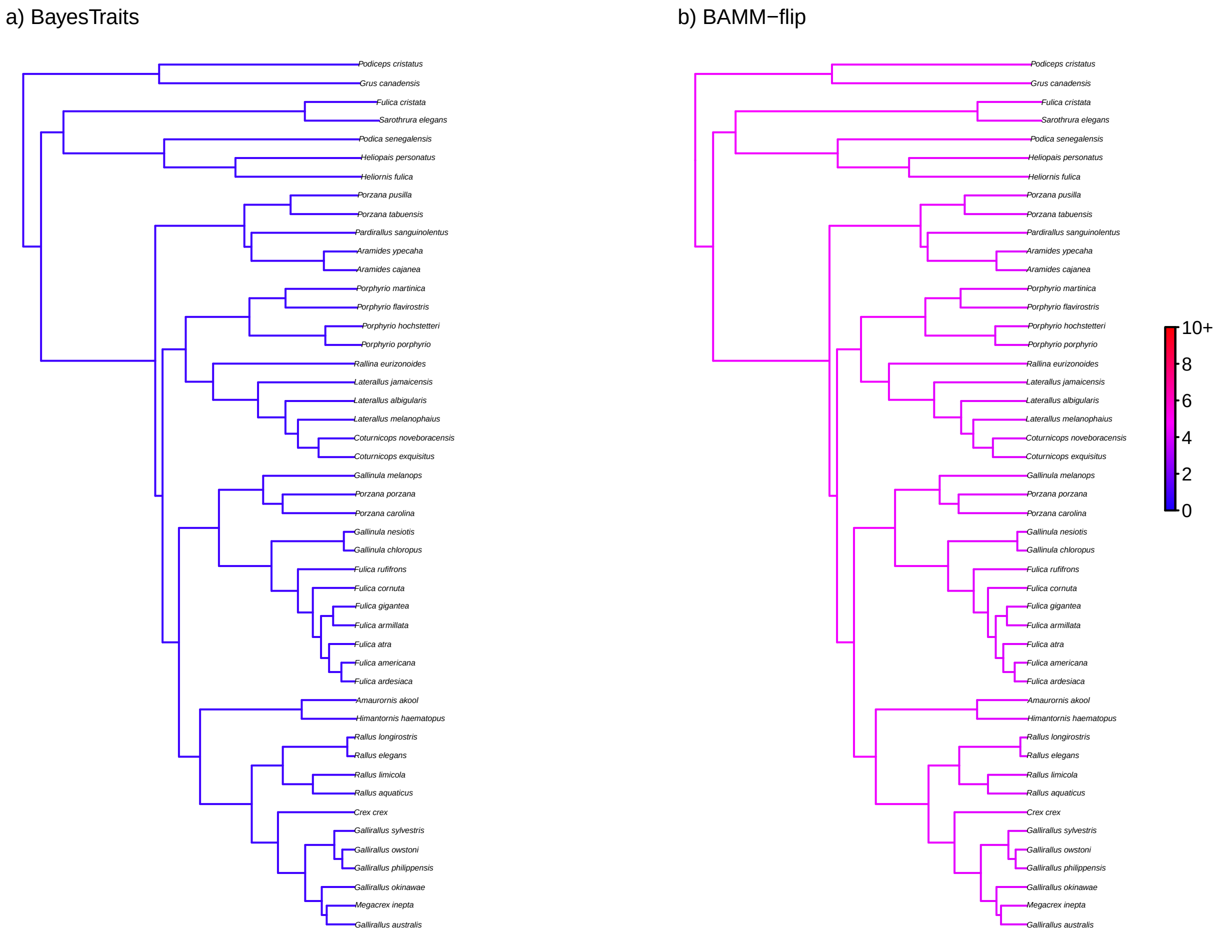

c)

	p_cvar	*p_shgt*	*p_svar*	*p_sasr*	*AICw*	*sigma*	*alpha*	*r*
BM	0.45 (−)	0.45 (−)	0.6 (−)	0.05 (+)	0.5	3.98		
OU	0.49 (−)	0.82 (+)	0.72 (+)	0.1 (+)	0.31	5.11	1.06	
EB	0.52 (+)	0.48 (+)	0.66 (+)	0.05 (+)	0.18	3.98		0
BayesTraits	0.46 (−)	0.39 (−)	0.7 (−)	0.05 (+)				
BAMM−flip	0.44 (−)	0.37 (−)	0.62 (−)	0.03 (+)				

Figure S52

Cuckoos

a) BayesTraits

b) BAMM−flip

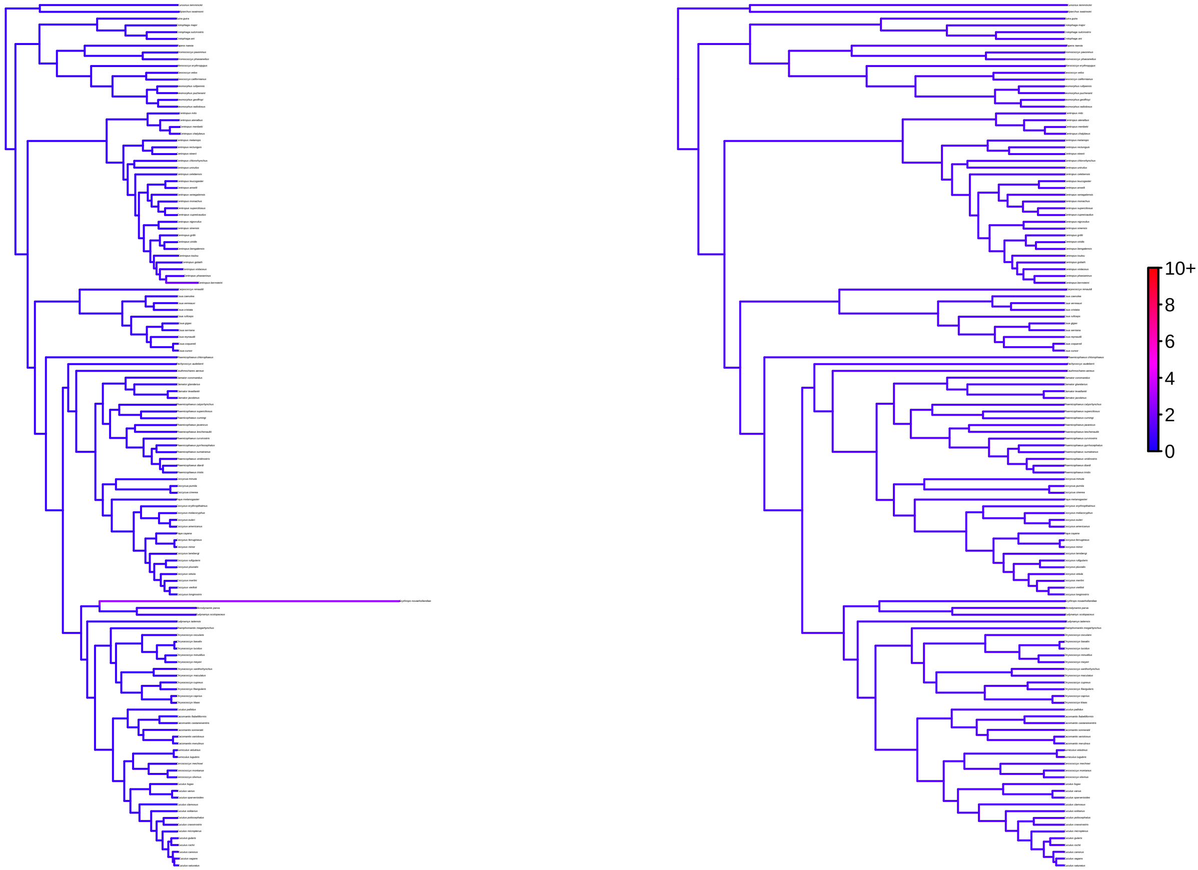

c)

	p_cvar	p_shgt	p_svar	p_sasr	AICw	sigma	alpha	r
BM	0.44 (+)	0.58 (−)	0.12 (−)	0.15 (+)	0.54	1.17		
OU	0.44 (+)	0.95 (+)	0.2 (−)	0.17 (+)	0.27	1.28	0.4	
EB	0.44 (−)	0.59 (−)	0.11 (−)	0.15 (+)	0.2	1.17		0
BayesTraits	1 (−)	0.54 (−)	0.29 (−)	0.28 (+)				
BAMM−flip	0.46 (+)	0.56 (−)	0.1 (−)	0.11 (+)				

Figure S53

Cisticolas, Allies

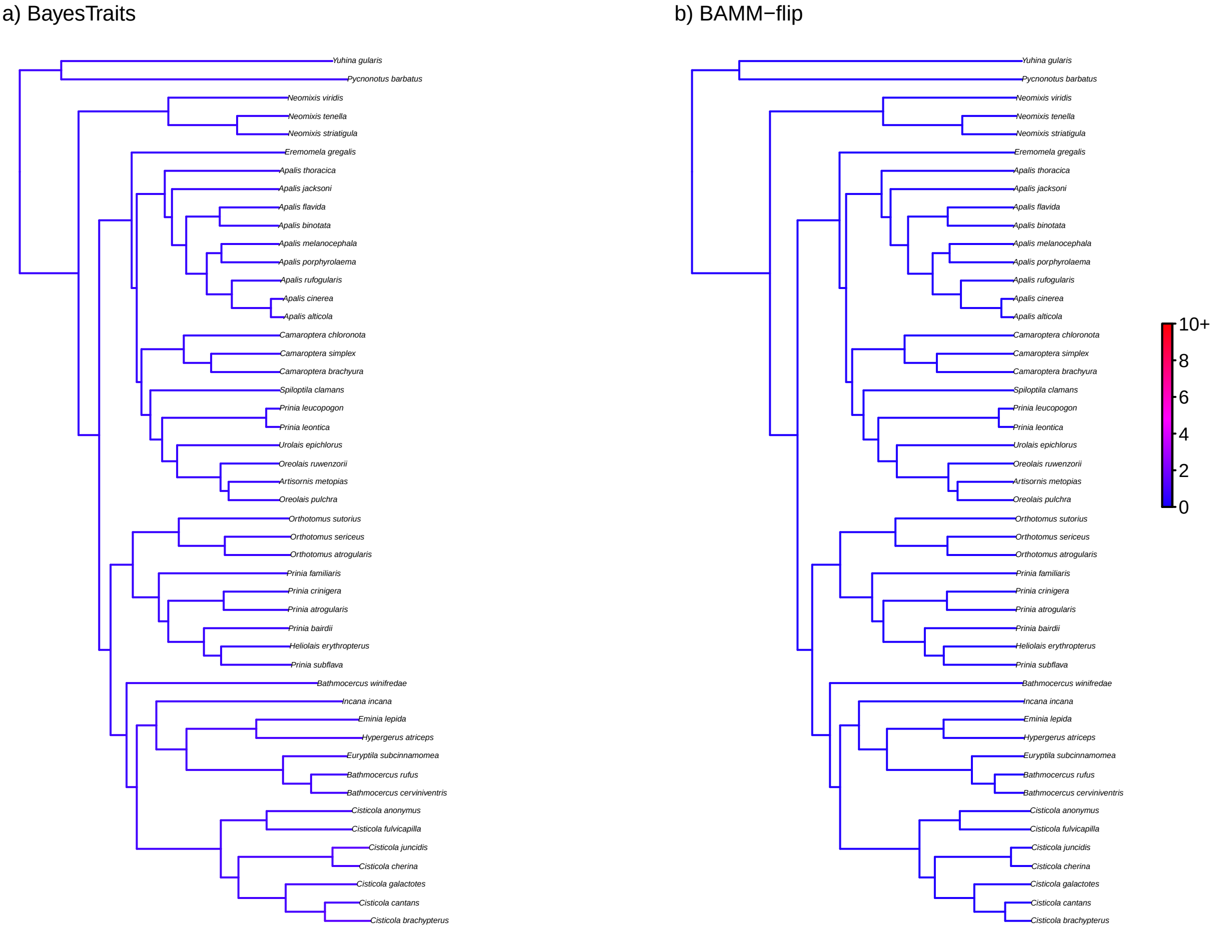

c)

	p_cvar	p_shgt	p_svar	p_sasr	AICw	sigma	alpha	r
BM	0.54 (−)	0.81 (+)	0.35 (−)	0.14 (+)	0.57	0.22		
OU	0.59 (−)	0.7 (+)	0.36 (−)	0.12 (+)	0.22	0.23	0.15	
EB	0.55 (+)	0.76 (+)	0.36 (+)	0.13 (+)	0.21	0.22		0
BayesTraits	0.2 (−)	0.86 (−)	0.6 (+)	0.17 (+)				
BAMM−flip	0.46 (−)	0.93 (+)	0.53 (−)	0.11 (+)				

Figure S54

Mockingbirds, Thrashers, Philippine Creepers, Starlings

a) BayesTraits

b) BAMM−flip

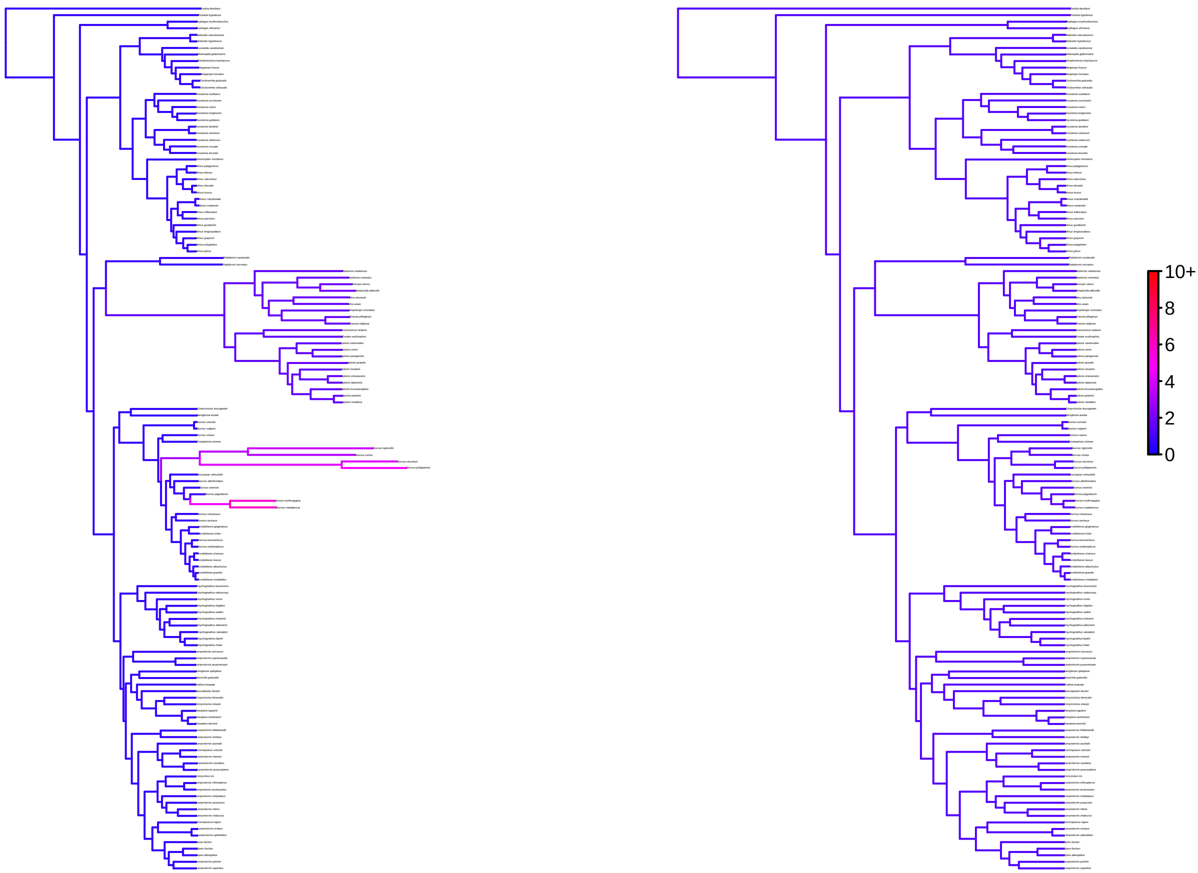

c)

	p_cvar	p_shgt	p_svar	p_sasr	AICw	sigma	alpha	r
BM	0 (+)	0.31 (−)	0.08 (−)	0.7 (+)	0.46	1.3		
OU	0 (+)	0.64 (−)	0.24 (−)	0.99 (+)	0.38	1.47	1.3	
EB	0 (−)	0.27 (+)	0.07 (−)	0.73 (+)	0.17	1.3		0
BayesTraits	0.65 (+)	0.52 (−)	0.34 (+)	0.55 (−)				
BAMM−flip	0.01 (+)	0.24 (−)	0.26 (−)	0.63 (+)				

Figure S55

Thrushes

a) BayesTraits

b) BAMM−flip

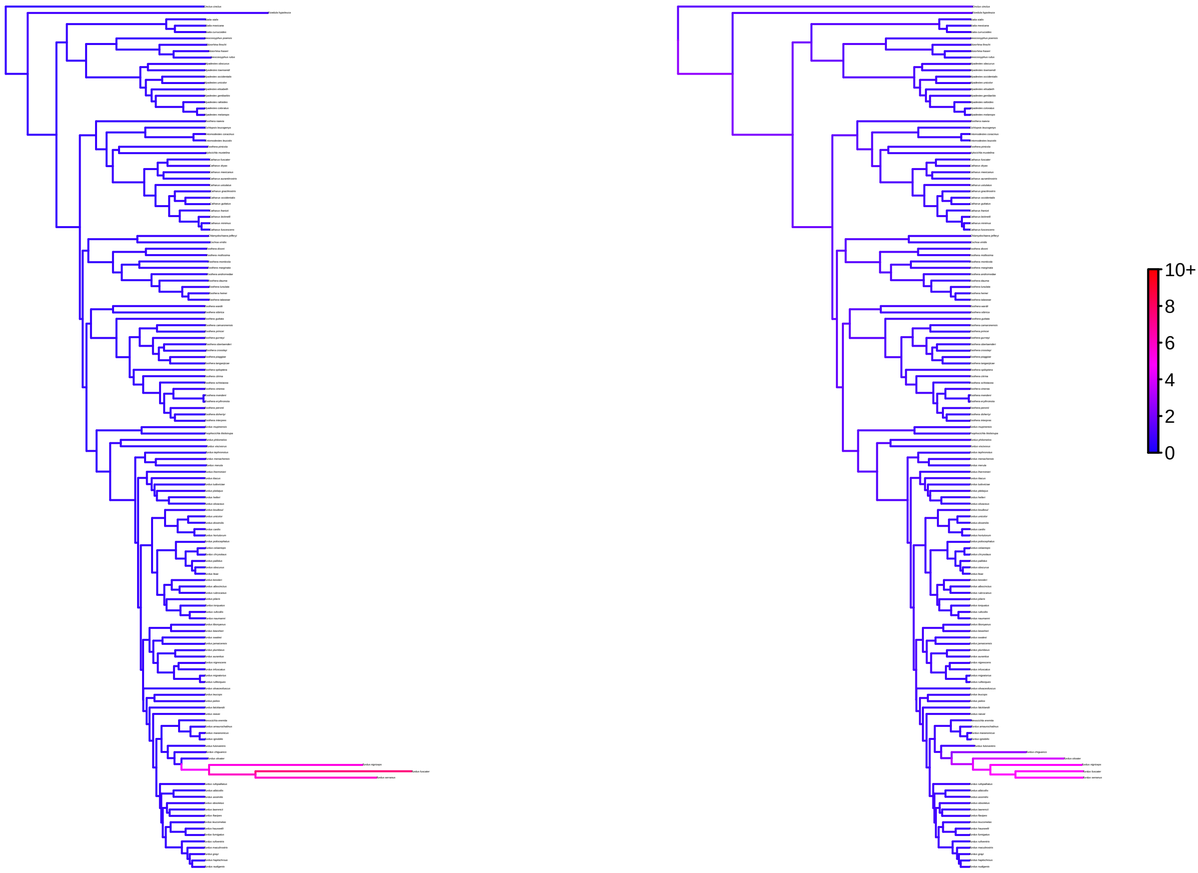

c)

	p_cvar	*p_shgt*	*p_svar*	*p_sasr*	*AICw*	*sigma*	*alpha*	*r*
BM	0.05 (+)	0.13 (+)	0.42 (+)	0.31 (+)	0.54	1.05		
OU	0.04 (+)	0.14 (+)	0.42 (+)	0.31 (+)	0.2	1.05	0	
EB	0.03 (+)	0.27 (−)	0.55 (−)	0.26 (+)	0.26	1.73		−2.89
BayesTraits	0.8 (+)	0.06 (+)	0.05 (+)	0.64 (+)				
BAMM−flip	0.58 (+)	0.13 (+)	0.12 (+)	0.53 (+)				

Figure S56

Buntings, American Sparrows, Brush−Finches

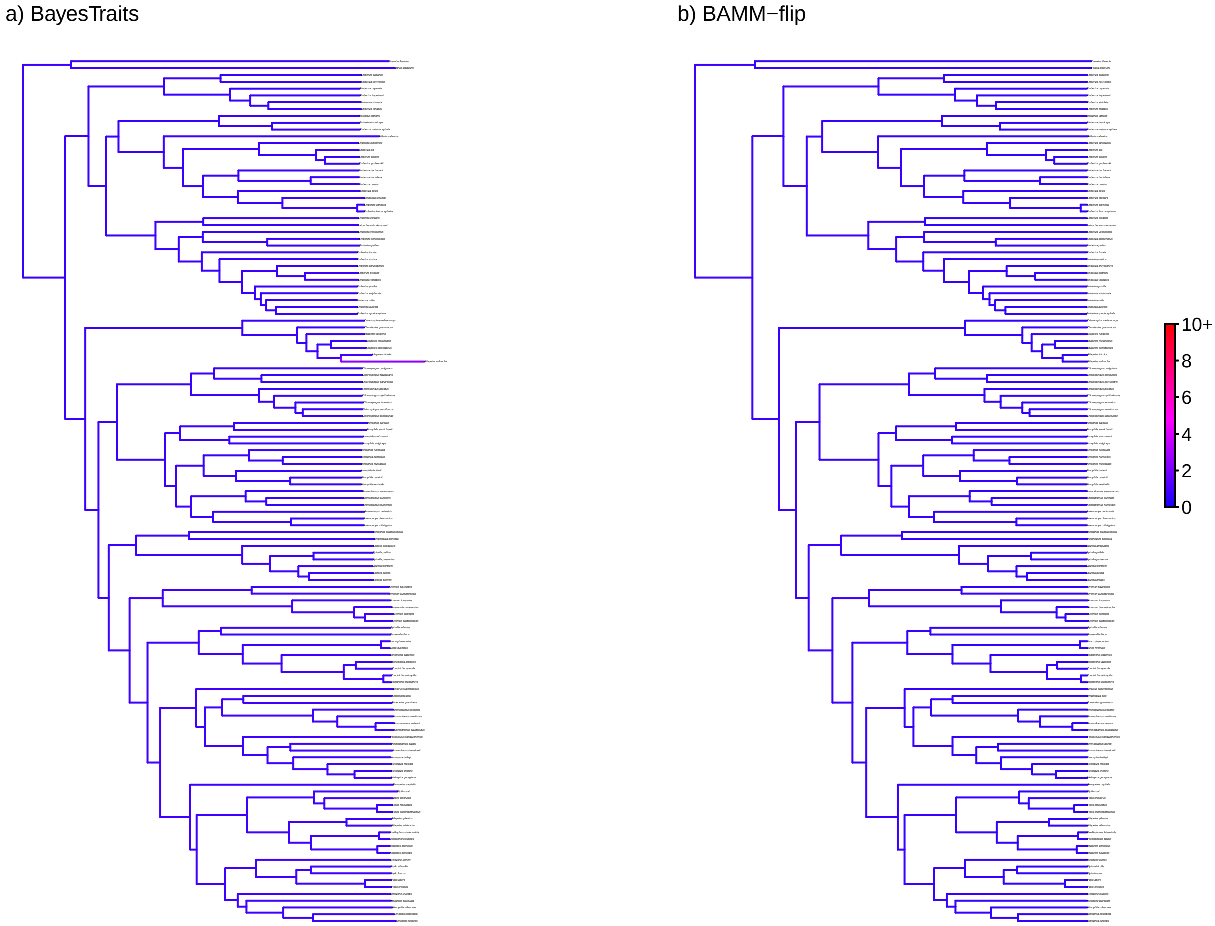

c)

	p_cvar	p_shgt	p_svar	p_sasr	AICw	sigma	alpha	r
BM	0.03 (+)	0.6 (+)	0.62 (−)	0.06 (+)	0.57	0.63		
OU	0.02 (+)	0.64 (+)	0.59 (−)	0.05 (+)	0.21	0.63	0	
EB	0.03 (+)	0.77 (−)	0.52 (−)	0.06 (−)	0.22	0.76		−0.83
BayesTraits	0.06 (+)	0.58 (+)	0.58 (+)	0.09 (+)				
BAMM−flip	0.01 (+)	0.67 (+)	0.65 (−)	0.06 (+)				

Figure S57

Waxbills, Allies

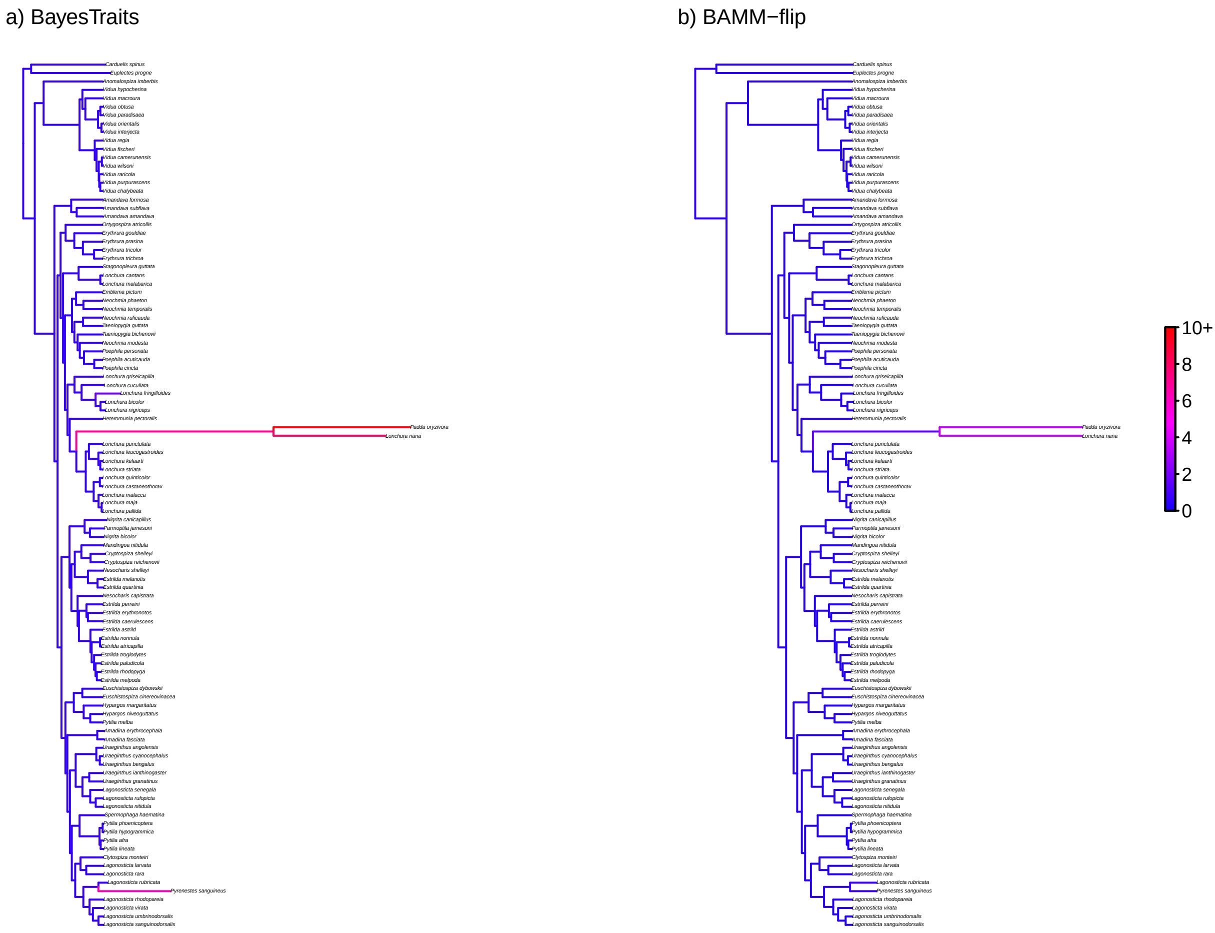

c)

	p_cvar	p_shgt	p_svar	p_sasr	AICw	sigma	alpha	r
BM	0 (+)	0.33 (+)	0.22 (+)	0 (+)	0.46	0.43		
OU	0 (+)	0.05 (+)	0.07 (+)	0 (+)	0.37	0.52	1.42	
EB	0 (+)	0.31 (+)	0.21 (+)	0 (+)	0.17	0.43		0
BayesTraits	0.16 (+)	0.83 (+)	0.02 (+)	0 (+)				
BAMM−flip	0.05 (+)	0.39 (+)	0.03 (+)	0 (+)				

Figure S58

Owls

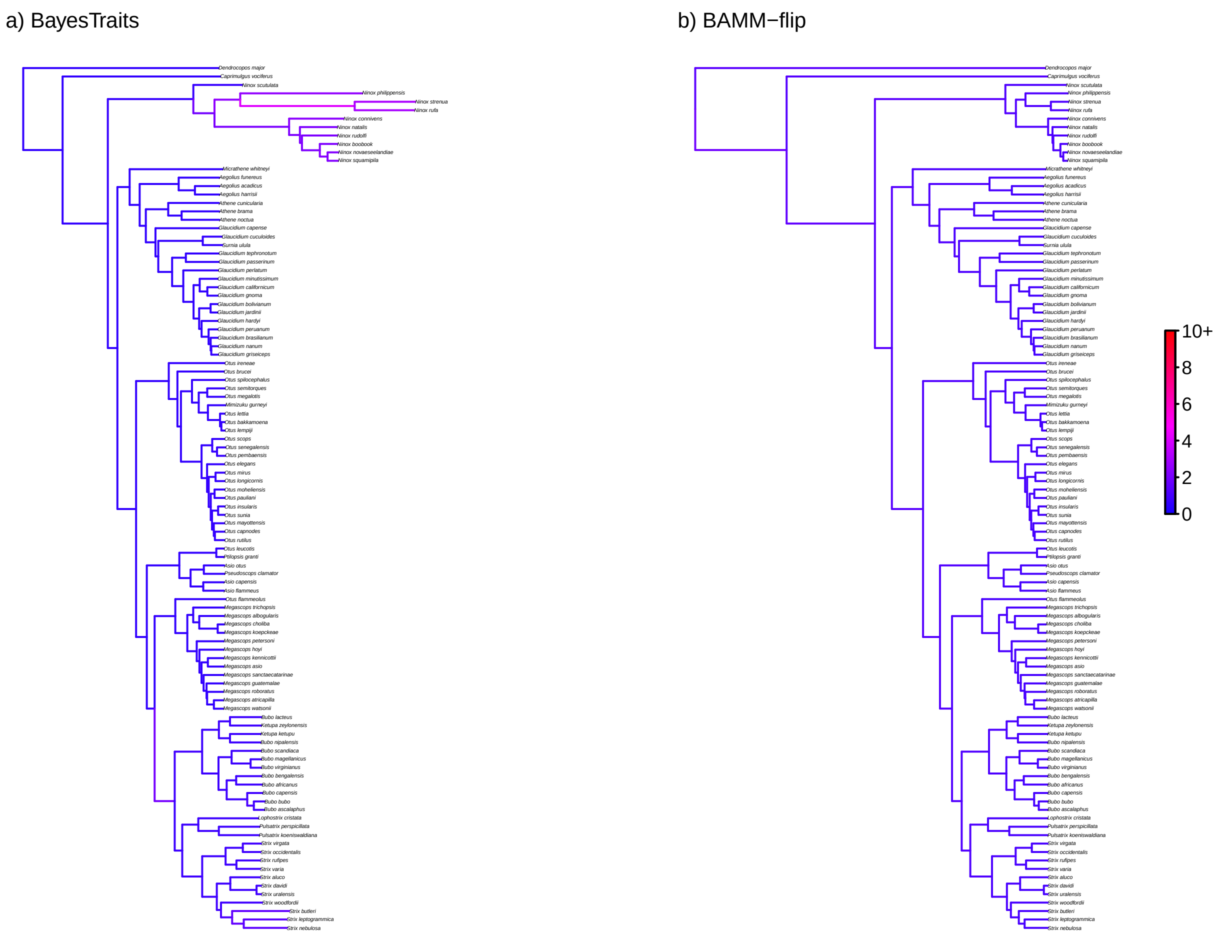

c)

	p_cvar	*p_shgt*	*p_svar*	*p_sasr*	*AICw*	*sigma*	*alpha*	*r*
BM	0.01 (+)	0.68 (+)	0.99 (−)	0.09 (+)	0.57	1.07		
OU	0.01 (+)	0.72 (+)	0.95 (−)	0.09 (+)	0.21	1.07	0	
EB	0.02 (+)	0.97 (+)	0.79 (+)	0.08 (+)	0.22	1.68		−0.36
BayesTraits	0.28 (+)	0.81 (−)	0.21 (+)	0.38 (+)				
BAMM−flip	0.03 (+)	0.86 (−)	0.96 (+)	0.1 (+)				

Figure S59

Woodpeckers

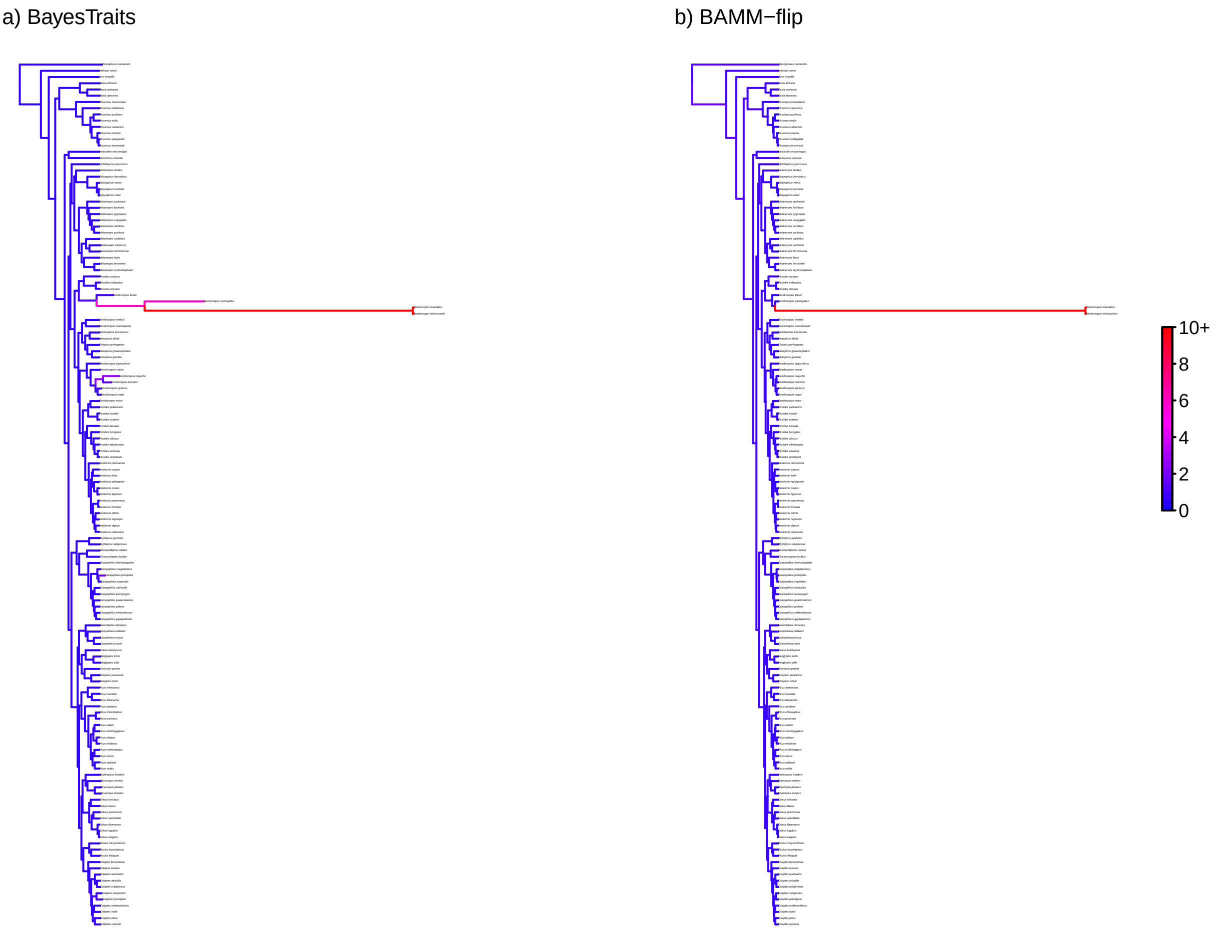

c)

	p_cvar	*p_shgt*	*p_svar*	*p_sasr*	*AICw*	*sigma*	*alpha*	*r*
BM	0 (+)	0.16 (−)	0 (−)	0 (−)	0.73	8.75		
OU	NA	NA	NA	NA	NA	NA	NA	NA
EB	0 (+)	0.11 (−)	0 (−)	0 (+)	0.27	8.75		0
BayesTraits	0.26 (+)	0.06 (−)	0.4 (−)	0.79 (+)				
BAMM−flip	0.09 (+)	0.02 (−)	0.07 (−)	0.31 (+)				

Figure S60

Pigeons, Doves

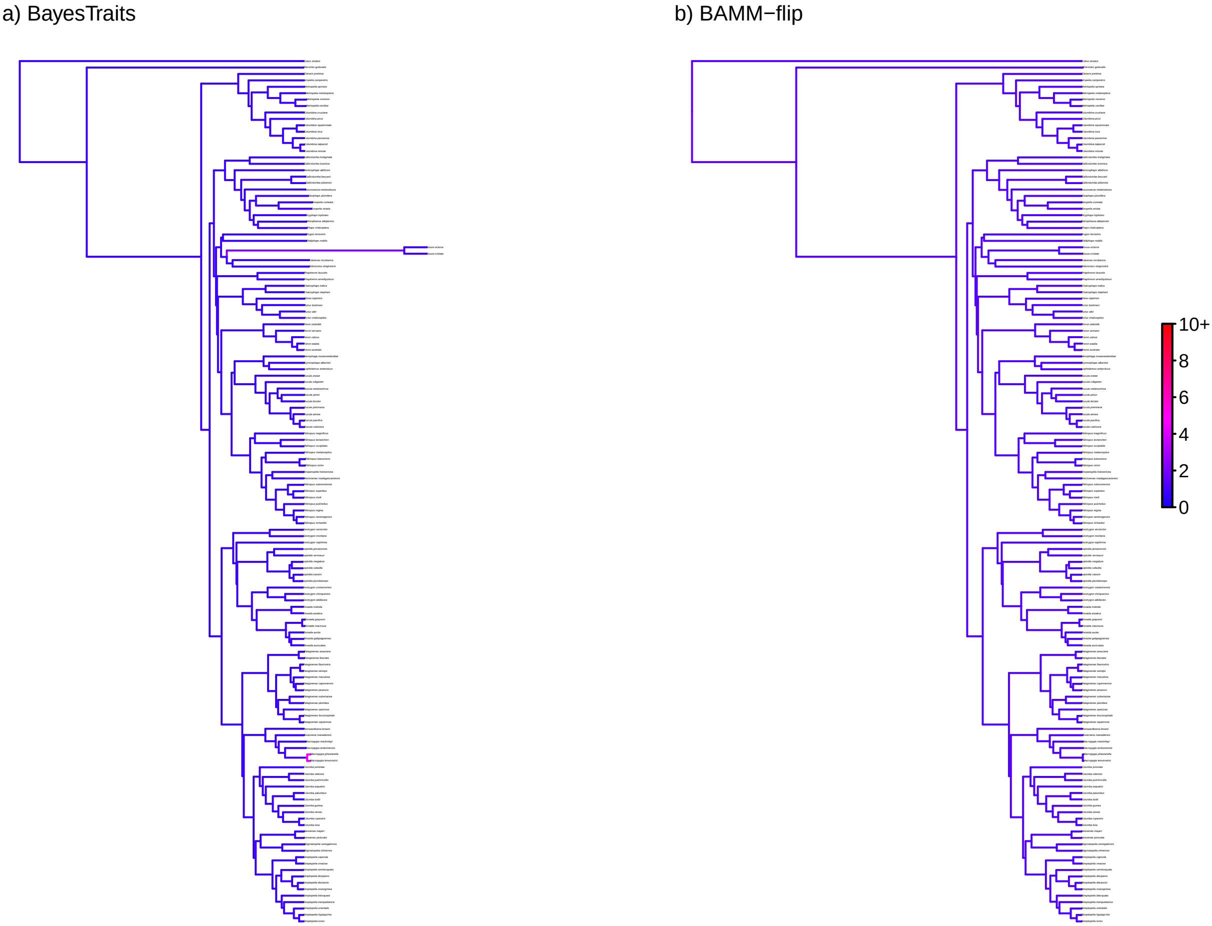

c)

	p_cvar	p_shgt	p_svar	p_sasr	AICw	sigma	alpha	r
BM	0.01 (+)	0.86 (+)	0.01 (−)	0.69 (−)	0.57	1.17		
OU	0 (+)	0.69 (+)	0.01 (−)	0.65 (−)	0.22	1.22	0.17	
EB	0 (+)	0.89 (−)	0.01 (−)	0.71 (+)	0.21	1.17		0
BayesTraits	0.07 (+)	0.83 (+)	0.11 (−)	0.43 (−)				
BAMM−flip	0 (+)	0.92 (+)	0.02 (−)	0.67 (−)				

Figure S61

Hummingbirds

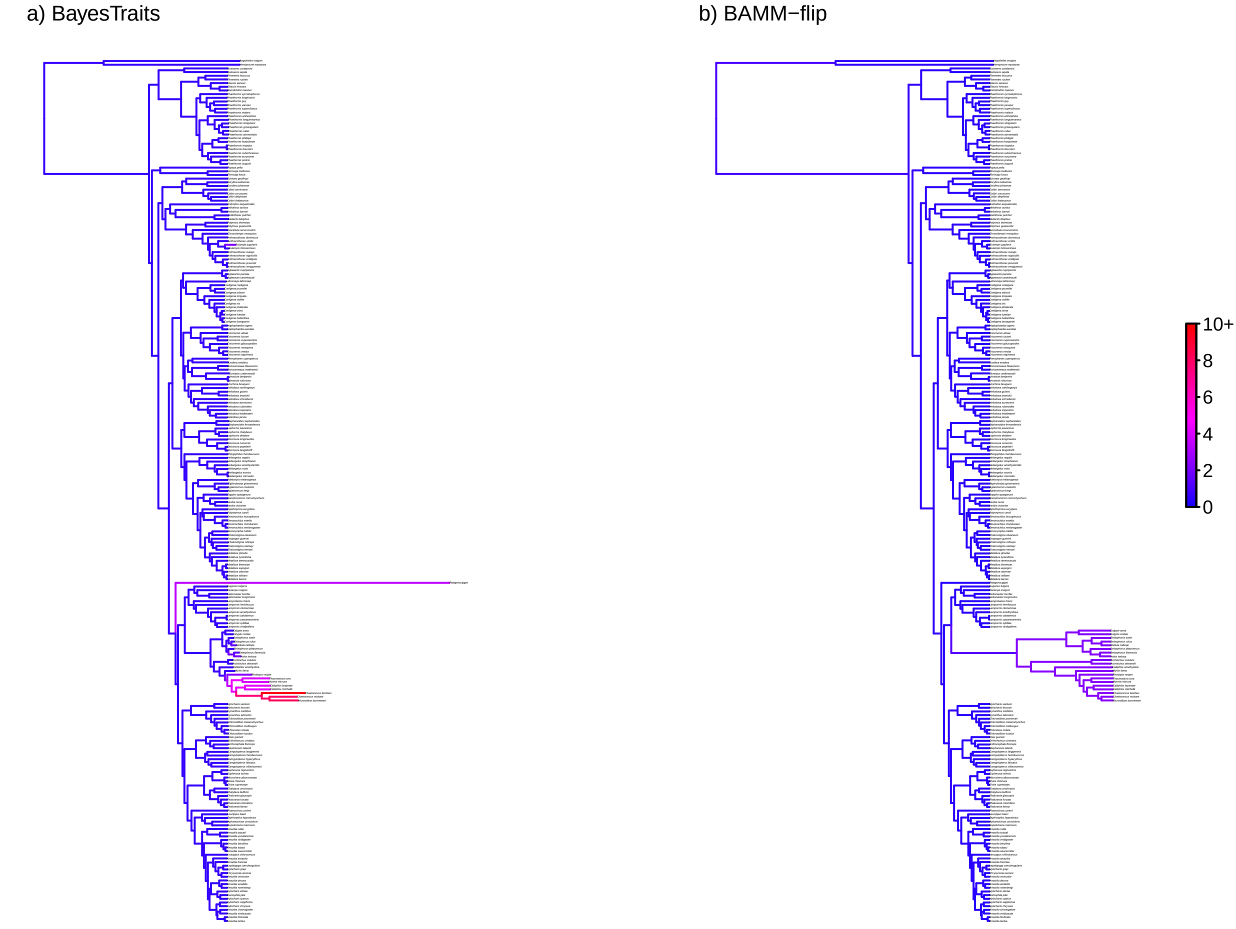

c)

	p_cvar	p_shgt	p_svar	p_sasr	AICw	sigma	alpha	r
BM	0 (+)	0.84 (+)	0.04 (−)	0.02 (−)	0.57	0.55		
OU	0 (+)	0.77 (+)	0.04 (−)	0.03 (−)	0.22	0.56	0.09	
EB	0 (+)	0.89 (+)	0.03 (−)	0.02 (−)	0.21	0.55		0
BayesTraits	0.1 (+)	0.24 (+)	0.39 (+)	0.38 (−)				
BAMM−flip	0 (+)	0.53 (+)	0.65 (−)	0.89 (−)				

Figure S62

Shrikes, Monarchs, Drongos, Fantails, Birds Of Paradise, Crows, Jays, Allies

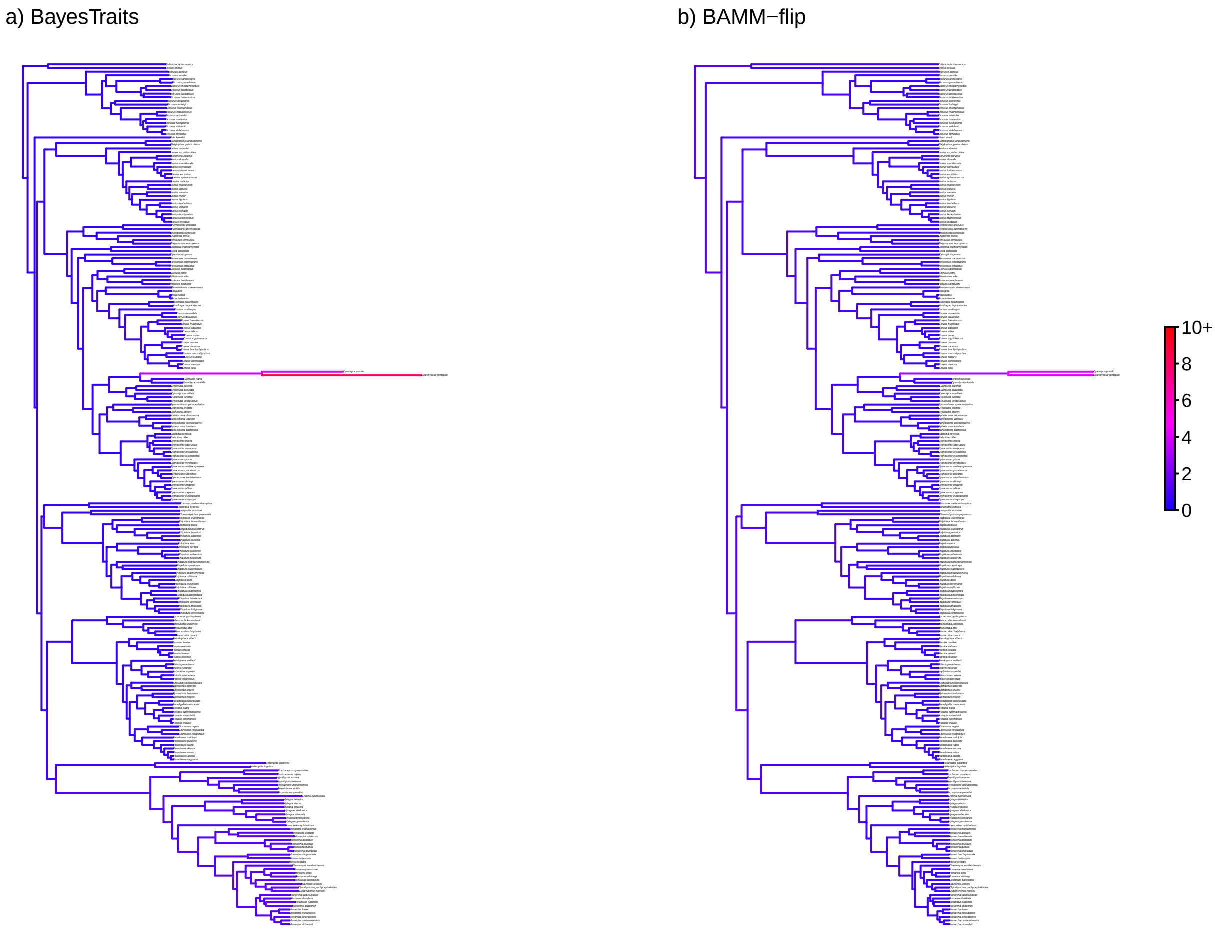

c)

	p_cvar	p_shgt	p_svar	p_sasr	AICw	sigma	alpha	r
BM	0.01 (+)	0.72 (+)	0.08 (−)	0.68 (+)	0.54	0.85		
OU	0.01 (+)	0.76 (+)	0.11 (−)	0.66 (+)	0.2	0.85	0	
EB	0.02 (+)	0.92 (+)	0.05 (−)	0.58 (−)	0.26	1.15		−0.47
BayesTraits	0.71 (+)	0.59 (+)	0.63 (+)	0.27 (+)				
BAMM−flip	0.15 (+)	0.96 (+)	0.27 (−)	0.68 (+)				

Figure S63

Penguins

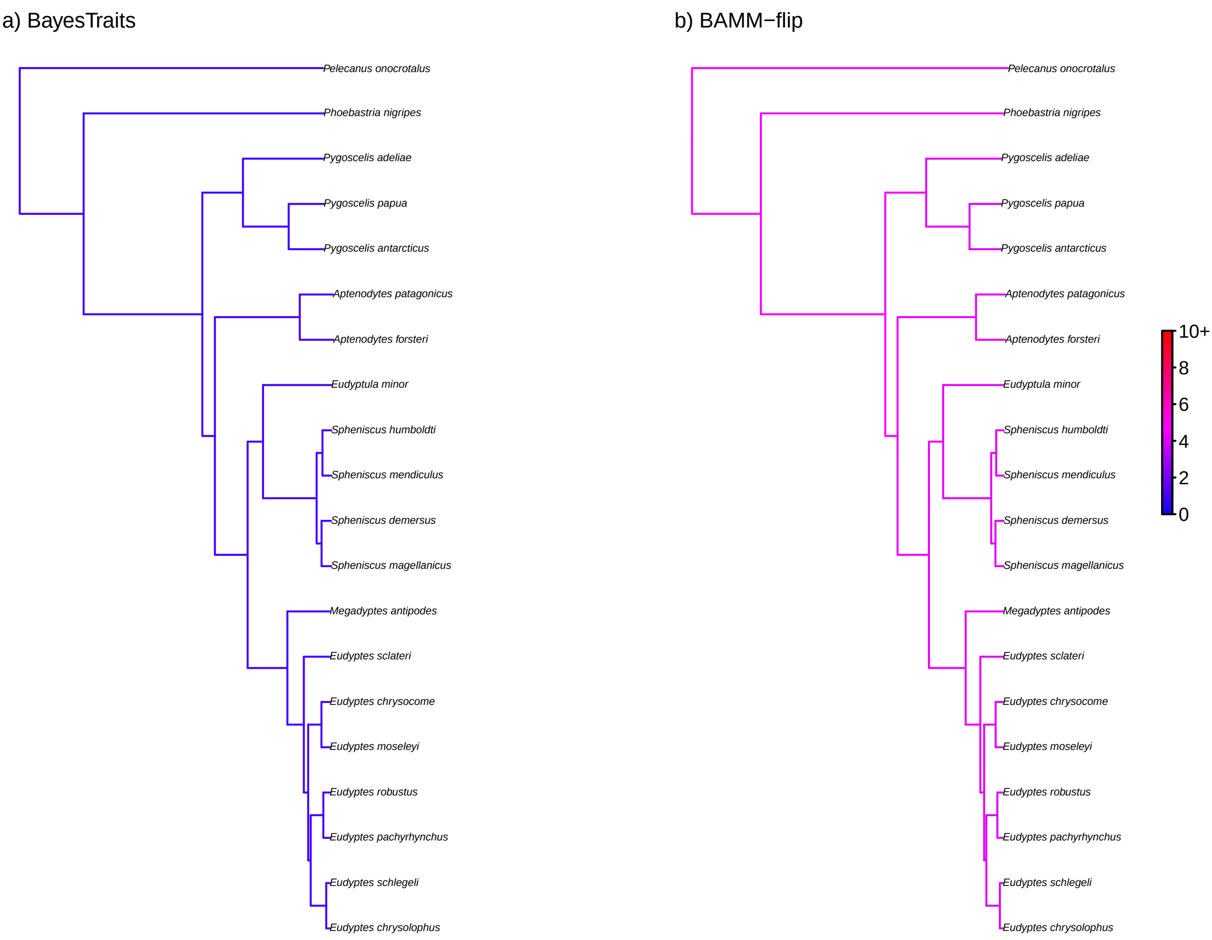

c)

	p_cvar	*p_shgt*	*p_svar*	*p_sasr*	*AICw*	*sigma*	*alpha*	*r*
BM	0.62 (+)	0.28 (−)	0.39 (−)	0.66 (+)	0.33	3.6		
OU	0.59 (+)	0.53 (+)	0.51 (+)	0.65 (+)	0.55	5.83	4.73	
EB	0.64 (+)	0.26 (−)	0.36 (−)	0.61 (+)	0.12	3.6		0
BayesTraits	0.71 (+)	0.33 (−)	0.49 (−)	0.6 (+)				
BAMM−flip	0.69 (+)	0.27 (−)	0.43 (−)	0.6 (+)				

Figure S64

Storks

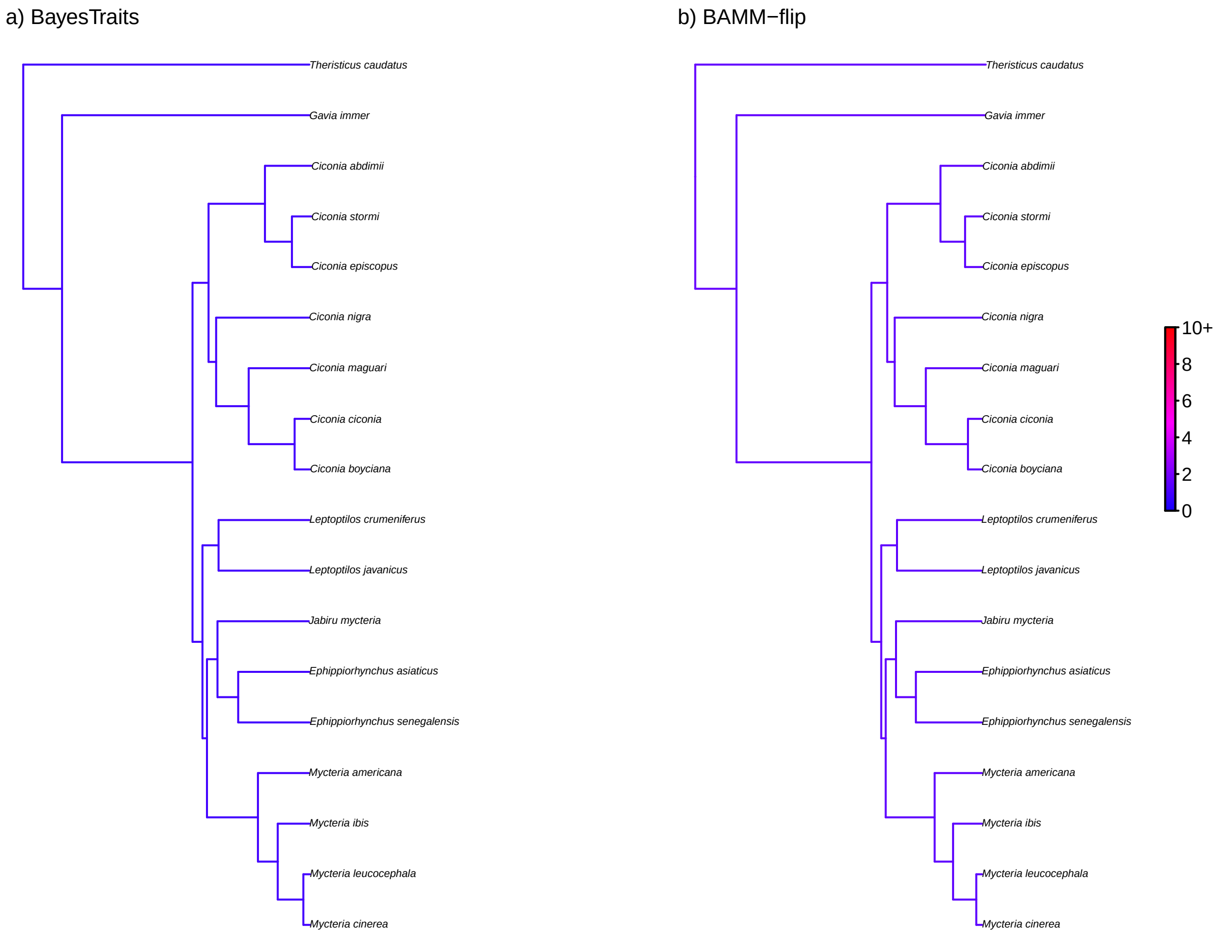

c)

	p_cvar	*p_shgt*	*p_svar*	*p_sasr*	*AICw*	*sigma*	*alpha*	*r*
BM	0.33 (−)	0.31 (−)	0.07 (−)	0.16 (−)	0.37	1.27		
OU	0.21 (−)	0.99 (+)	0.08 (+)	0.43 (−)	0.5	2.92	7.08	
EB	0.34 (−)	0.31 (−)	0.07 (−)	0.19 (−)	0.13	1.27		0
BayesTraits	0.33 (−)	0.33 (−)	0.09 (−)	0.18 (−)				
BAMM−flip	0.28 (−)	0.31 (−)	0.08 (−)	0.16 (−)				

Figure S65

Cormorants, Anhingas, Gannets, Frigatebirds

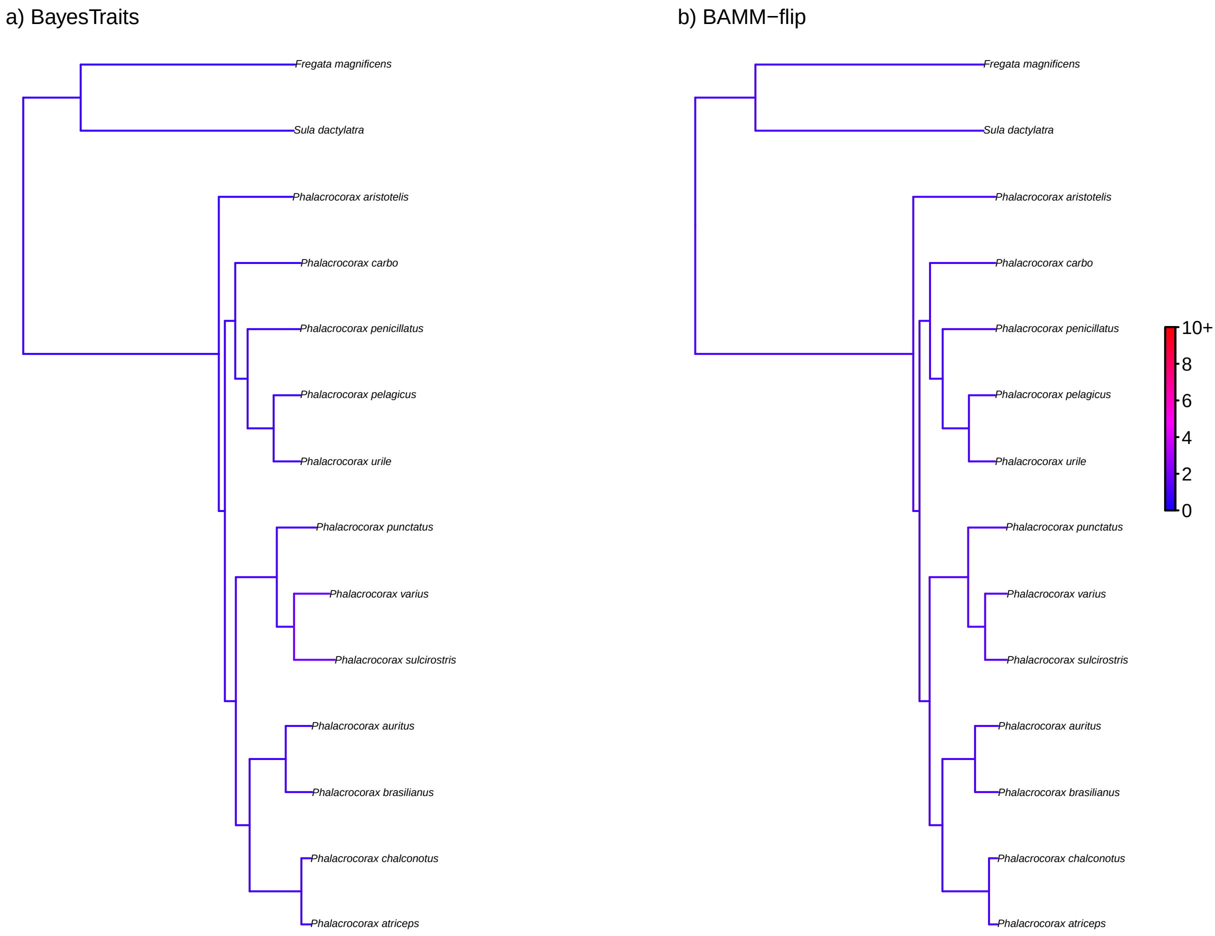

c)

	p_cvar	*p_shgt*	*p_svar*	*p_sasr*	*AICw*	*sigma*	*alpha*	*r*
BM	0.23 (+)	0.02 (−)	0.02 (−)	0.02 (−)	0.02	0.94		
OU	0.77 (+)	0.92 (+)	0.25 (+)	0.26 (+)	0.97	4.44	33.32	
EB	0.23 (+)	0.03 (−)	0.02 (−)	0.03 (−)	0.01	0.94		0
BayesTraits	0.94 (+)	0.02 (−)	0.07 (−)	0.06 (−)				
BAMM−flip	0.55 (+)	0.02 (−)	0.03 (−)	0.05 (−)				

Figure S66

Orioles, Allies

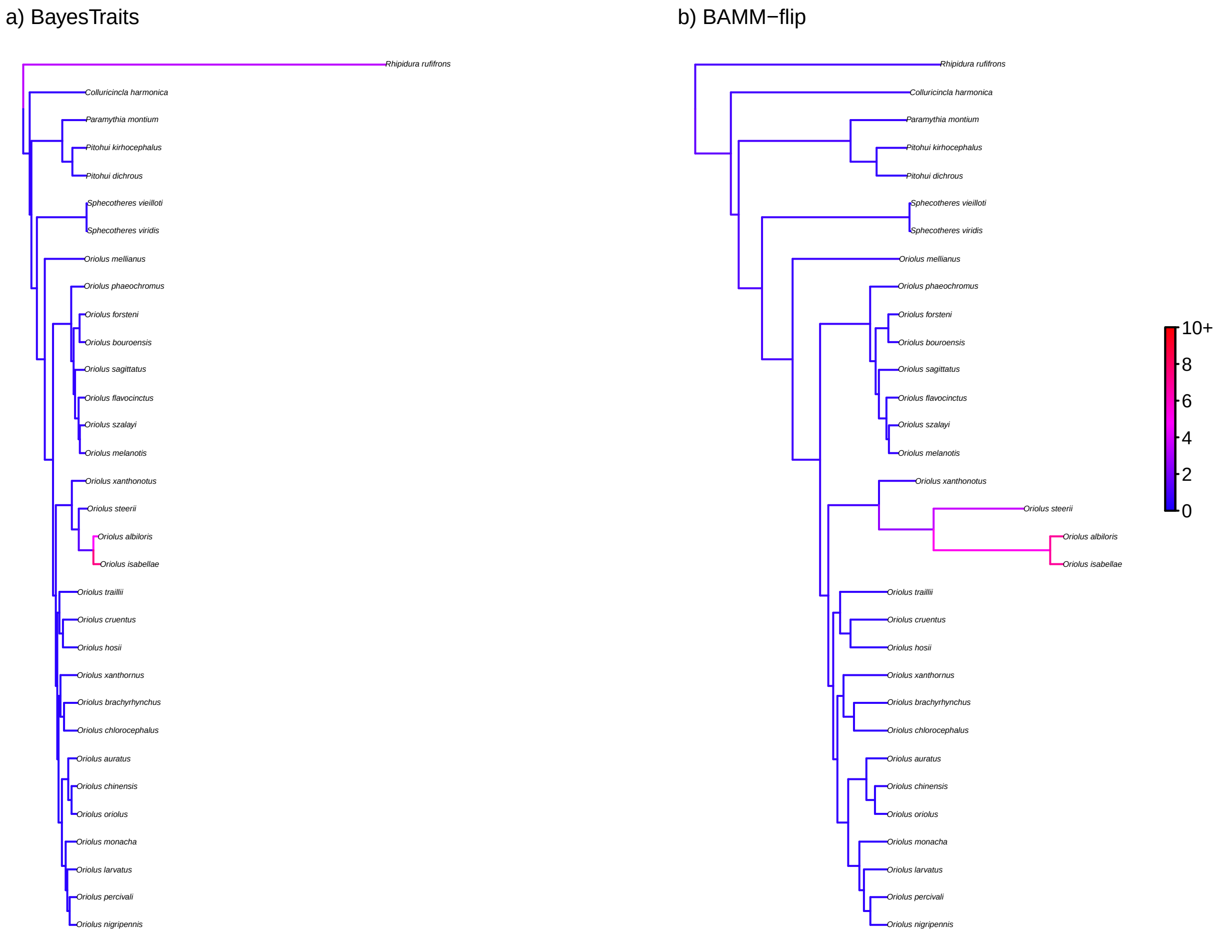

c)

	p_cvar	*p_shgt*	*p_svar*	*p_sasr*	*AICw*	*sigma*	*alpha*	*r*
BM	0 (+)	0.43 (−)	0.04 (−)	0.01 (−)	0.15	1.23		
OU	0 (+)	0.3 (+)	0.09 (−)	0.12 (−)	0.79	5.65	14.62	
EB	0 (+)	0.38 (−)	0.02 (−)	0.01 (−)	0.06	1.23		0
BayesTraits	0.56 (−)	0.13 (−)	0.6 (+)	0.21 (+)				
BAMM−flip	0.77 (−)	0.15 (−)	0.87 (+)	0.12 (+)				

Figure S67

Sparrows, Snowfinches, Allies

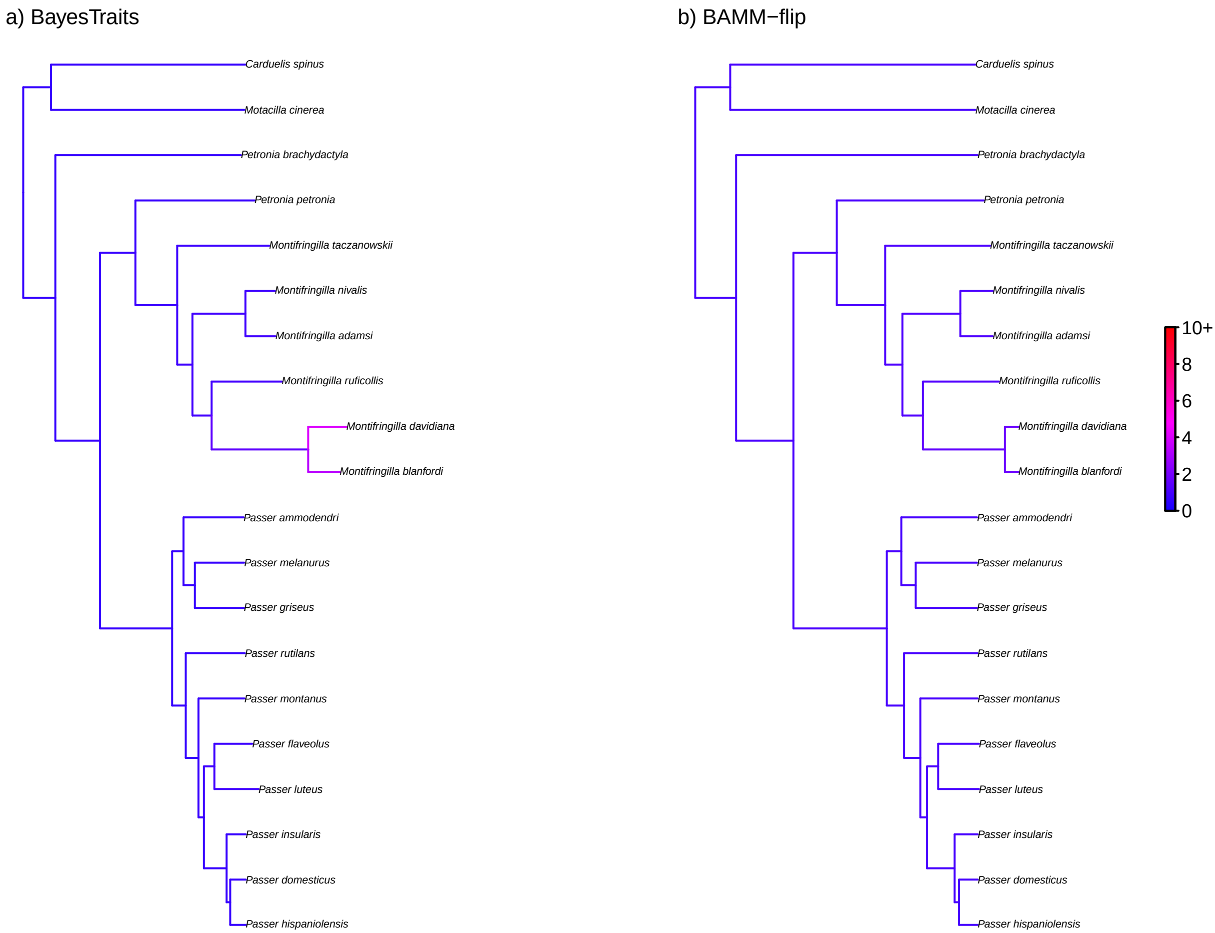

c)

	p_cvar	*p_shgt*	*p_svar*	*p_sasr*	*AICw*	*sigma*	*alpha*	*r*
BM	0.04 (+)	0.09 (−)	0.02 (−)	0.9 (+)	0.06	1.11		
OU	0.58 (+)	0.53 (−)	0 (−)	0.94 (−)	0.92	3.62	11.92	
EB	0.04 (+)	0.08 (−)	0.03 (−)	0.92 (+)	0.02	1.11		0
BayesTraits	0.96 (−)	0.19 (−)	0.33 (−)	0.78 (−)				
BAMM−flip	0.61 (+)	0.1 (−)	0.15 (−)	0.81 (−)				

Figure S68

Curassows, Chachalacas, Guans

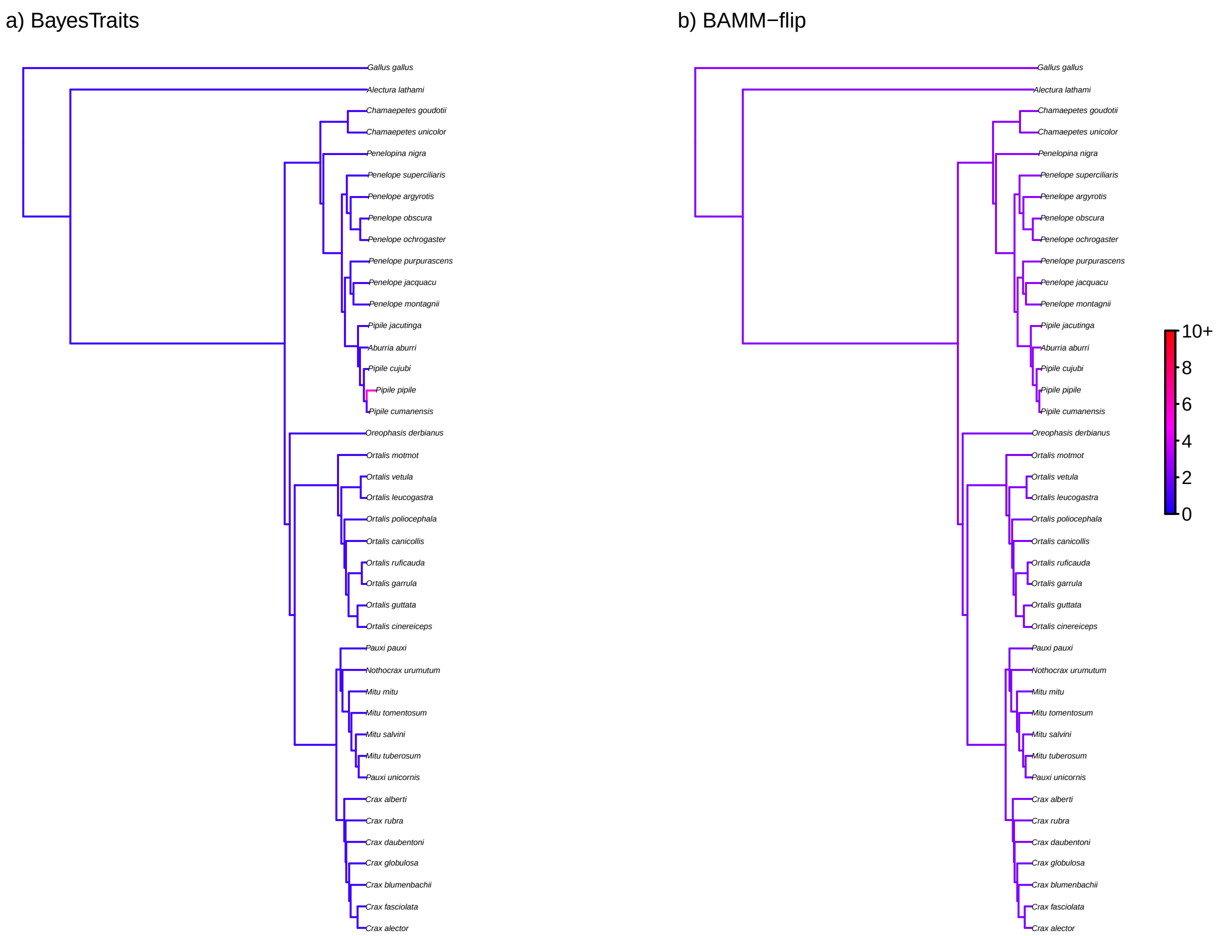

c)

	p_cvar	p_shgt	p_svar	p_sasr	AICw	sigma	alpha	r
BM	0.1 (+)	0.45 (−)	0.36 (−)	0.72 (+)	0.28	2.2		
OU	0.14 (+)	0.61 (+)	0.92 (+)	0.76 (+)	0.61	2.85	3.74	
EB	0.11 (−)	0.44 (−)	0.34 (+)	0.69 (+)	0.1	2.2		0
BayesTraits	0.74 (+)	0.6 (−)	0.89 (−)	0.71 (+)				
BAMM−flip	0.18 (+)	0.33 (−)	0.44 (−)	0.66 (+)				

Figure S69

Wagtails, Pipits

a) BayesTraits

b) BAMM−flip

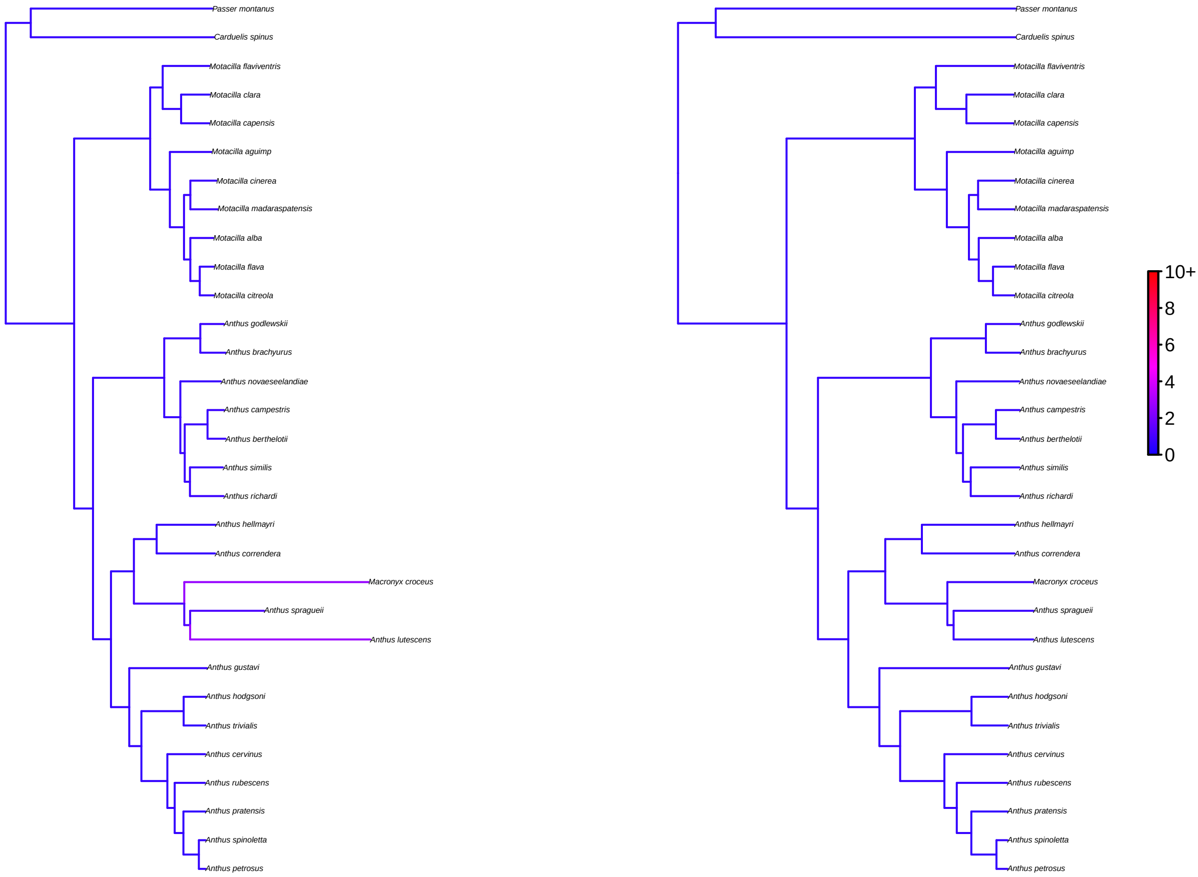

c)

	p_cvar	*p_shgt*	*p_svar*	*p_sasr*	*AICw*	*sigma*	*alpha*	*r*
BM	0.02 (+)	0.05 (−)	0.12 (−)	0.15 (+)	0	0.61		
OU	0.14 (+)	0.31 (+)	0.15 (+)	0.85 (+)	1	5.95	45.99	
EB	0.03 (−)	0.03 (+)	0.09 (+)	0.22 (+)	0	0.61		0
BayesTraits	0.21 (+)	0.01 (−)	0.72 (−)	0.2 (+)				
BAMM−flip	0.05 (+)	0.01 (−)	0.37 (−)	0.23 (+)				

Figure S70

Babblers II, Old World Warblers II, Allies

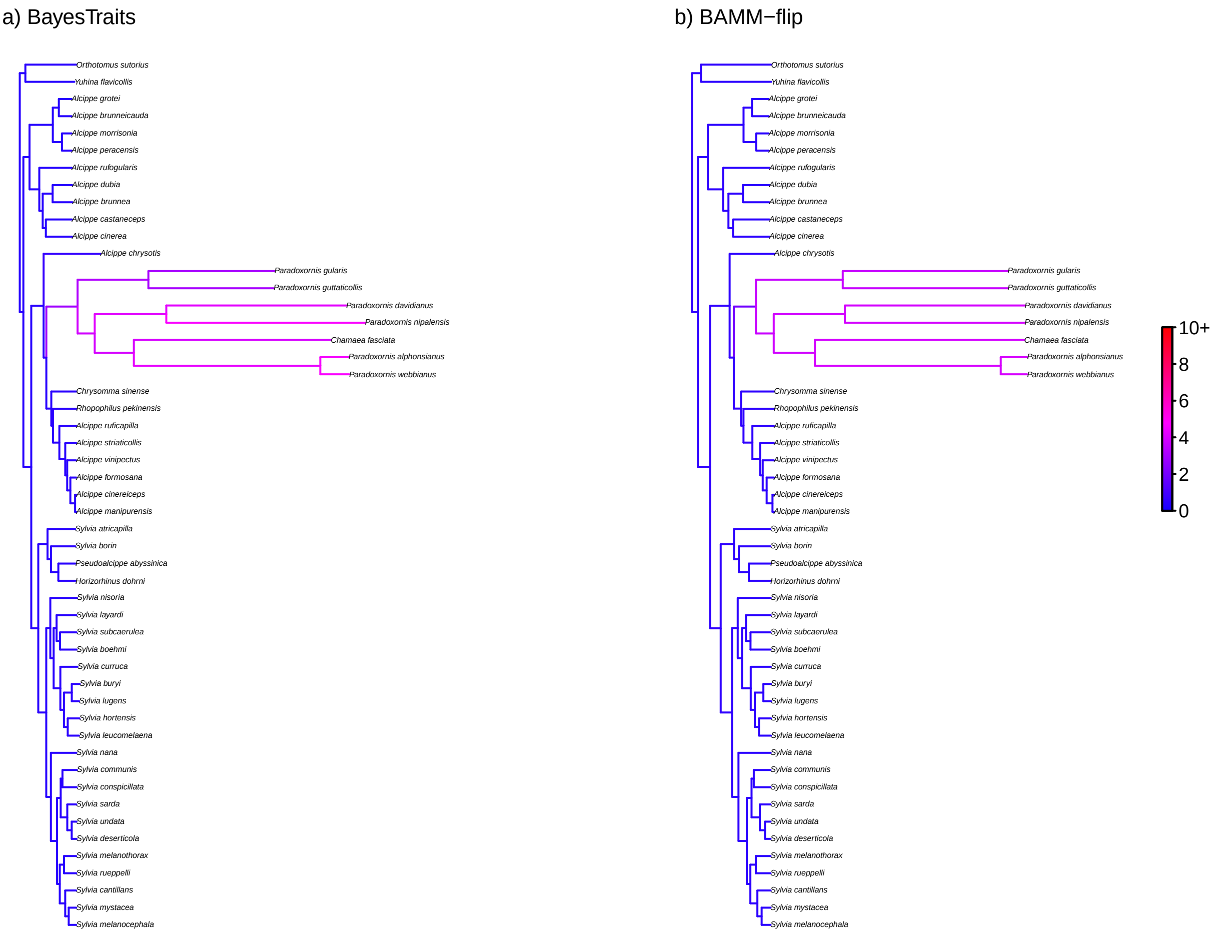

c)

	p_cvar	p_shgt	p_svar	p_sasr	AICw	sigma	alpha	r
BM	0 (+)	0.25 (−)	0.17 (−)	0.84 (−)	0.01	0.87		
OU	0 (+)	0.81 (+)	0.41 (−)	0.97 (−)	0.98	2.53	10.2	
EB	0 (−)	0.23 (+)	0.18 (+)	0.93 (−)	0.01	0.87		0
BayesTraits	0.06 (+)	0.25 (−)	0.97 (−)	0.46 (−)				
BAMM−flip	0.05 (+)	0.19 (−)	0.88 (−)	0.6 (−)				

Figure S71

Larks

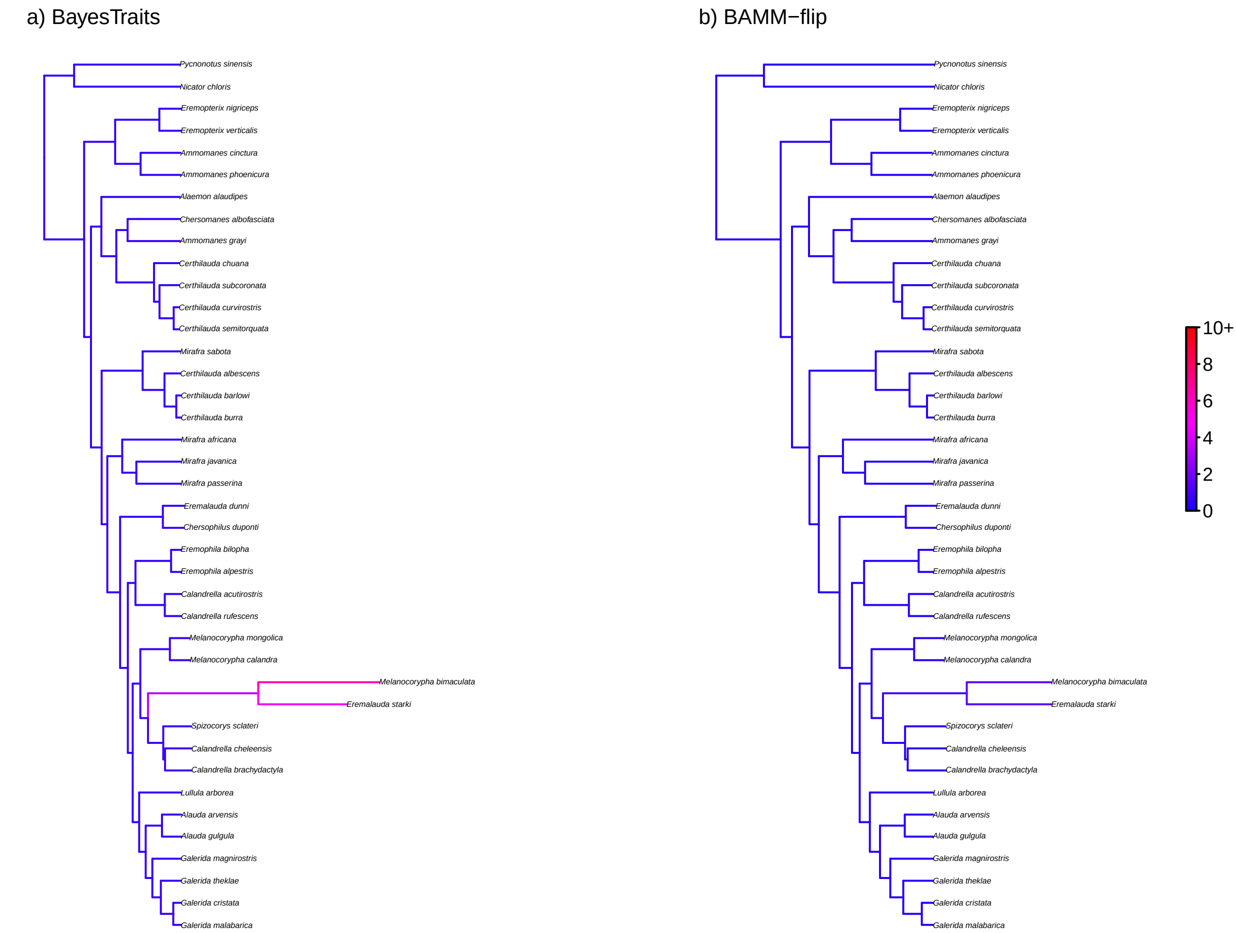

c)

	p_cvar	*p_shgt*	*p_svar*	*p_sasr*	*AICw*	*sigma*	*alpha*	*r*
BM	0 (+)	0.21 (−)	0.28 (−)	0.51 (−)	0.05	0.41		
OU	0.06 (+)	0.48 (+)	0.44 (−)	0.53 (−)	0.94	0.96	3.54	
EB	0 (+)	0.25 (+)	0.27 (−)	0.51 (+)	0.02	0.41		0
BayesTraits	0.18 (+)	0.29 (−)	0.57 (+)	0.49 (−)				
BAMM−flip	0.14 (+)	0.24 (−)	0.36 (+)	0.52 (−)				

Figure S72

Old World Warblers I

a) BayesTraits

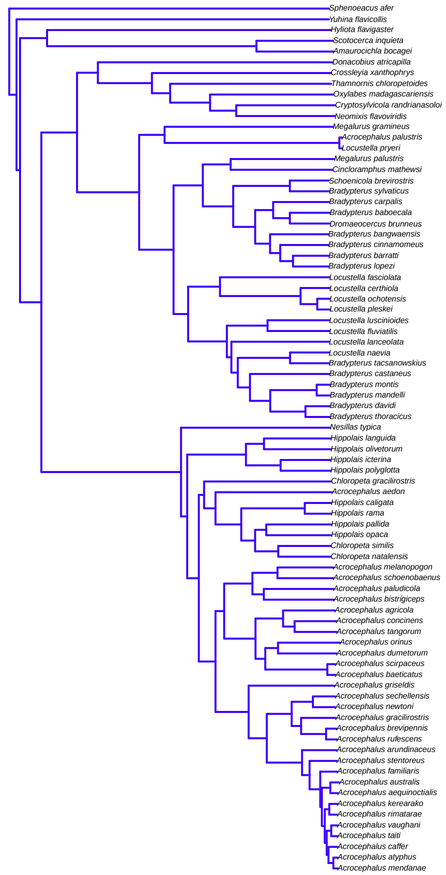

b) BAMM−flip

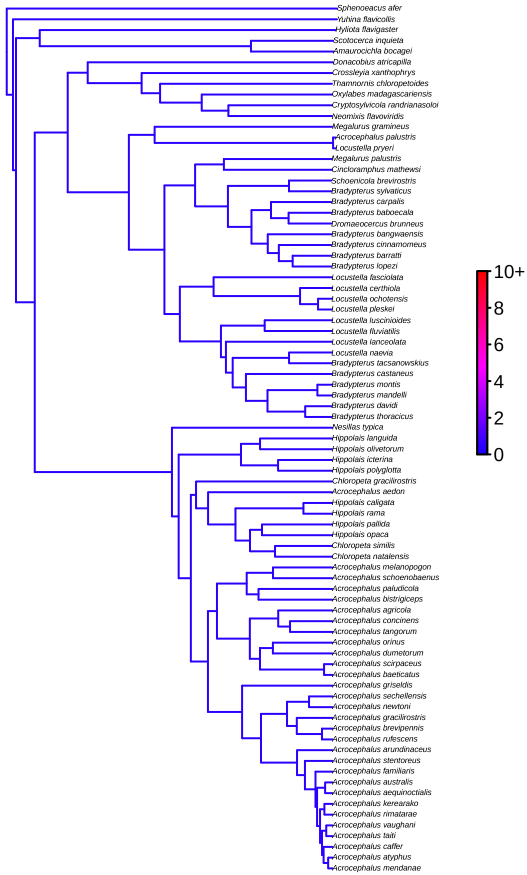

c)

	p_cvar	*p_shgt*	*p_svar*	*p_sasr*	*AICw*	*sigma*	*alpha*	*r*
BM	0.14 (+)	0.26 (−)	0.04 (−)	0.03 (+)	0.1	0.44		
OU	0.38 (+)	0.59 (+)	0.19 (−)	0.19 (+)	0.87	0.67	1.79	
EB	0.17 (+)	0.24 (−)	0.02 (−)	0.03 (+)	0.04	0.44		0
BayesTraits	0.26 (+)	0.3 (−)	0.09 (−)	0.03 (+)				
BAMM−flip	0.16 (+)	0.25 (−)	0.04 (−)	0.02 (+)				

Figure S73

Ducks III, Geese II, Other Waterfowl, Screamers III

a) BayesTraits

b) BAMM−flip

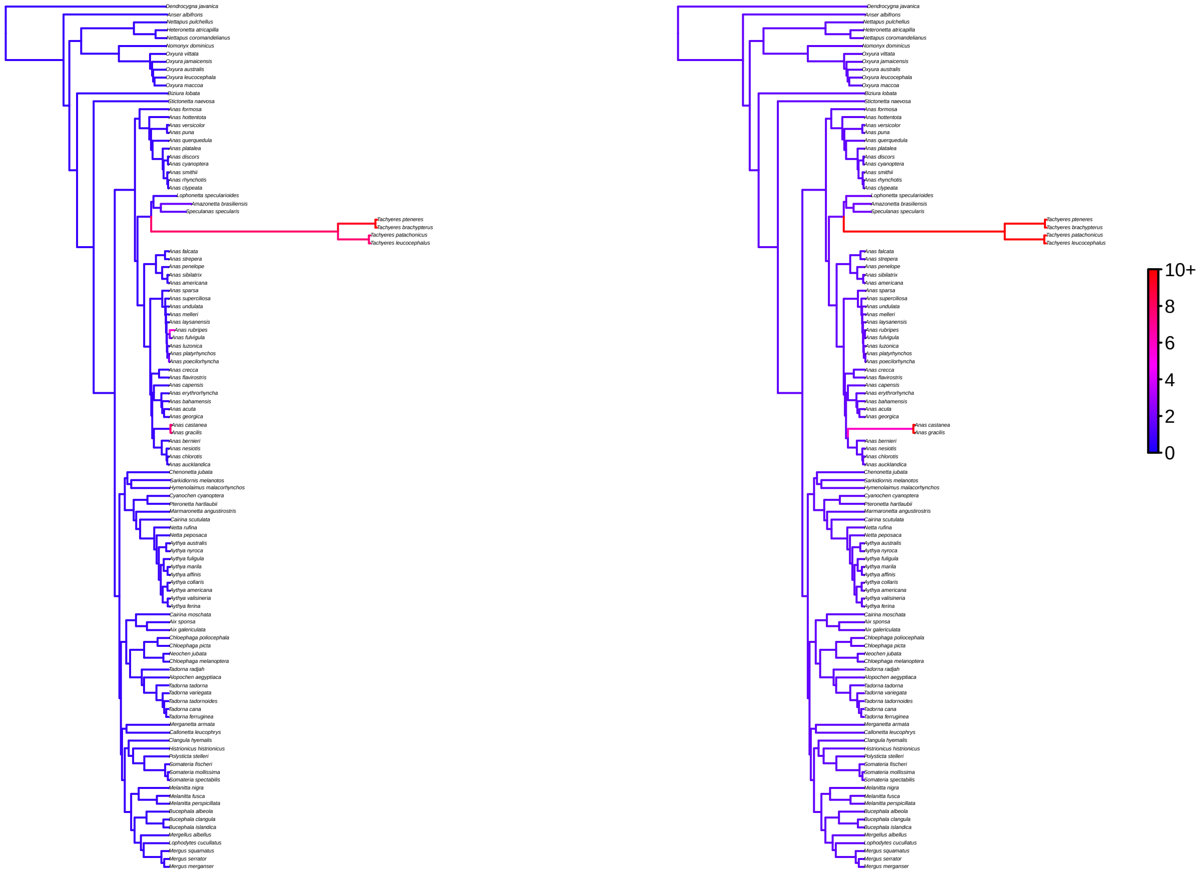

c)

	p_cvar	p_shgt	p_svar	p_sasr	AICw	sigma	alpha	r
BM	0 (+)	0.06 (−)	0 (−)	0 (+)	0	2.64		
OU	0 (+)	0.38 (+)	0.03 (−)	0.01 (+)	1	4.44	5.85	
EB	0 (+)	0.04 (−)	0 (−)	0 (+)	0	2.64		0
BayesTraits	0.47 (−)	0.45 (−)	0.69 (−)	0.07 (+)				
BAMM−flip	0.53 (+)	0.22 (−)	0.12 (−)	0.01 (+)				

Figure S74

New World Warblers

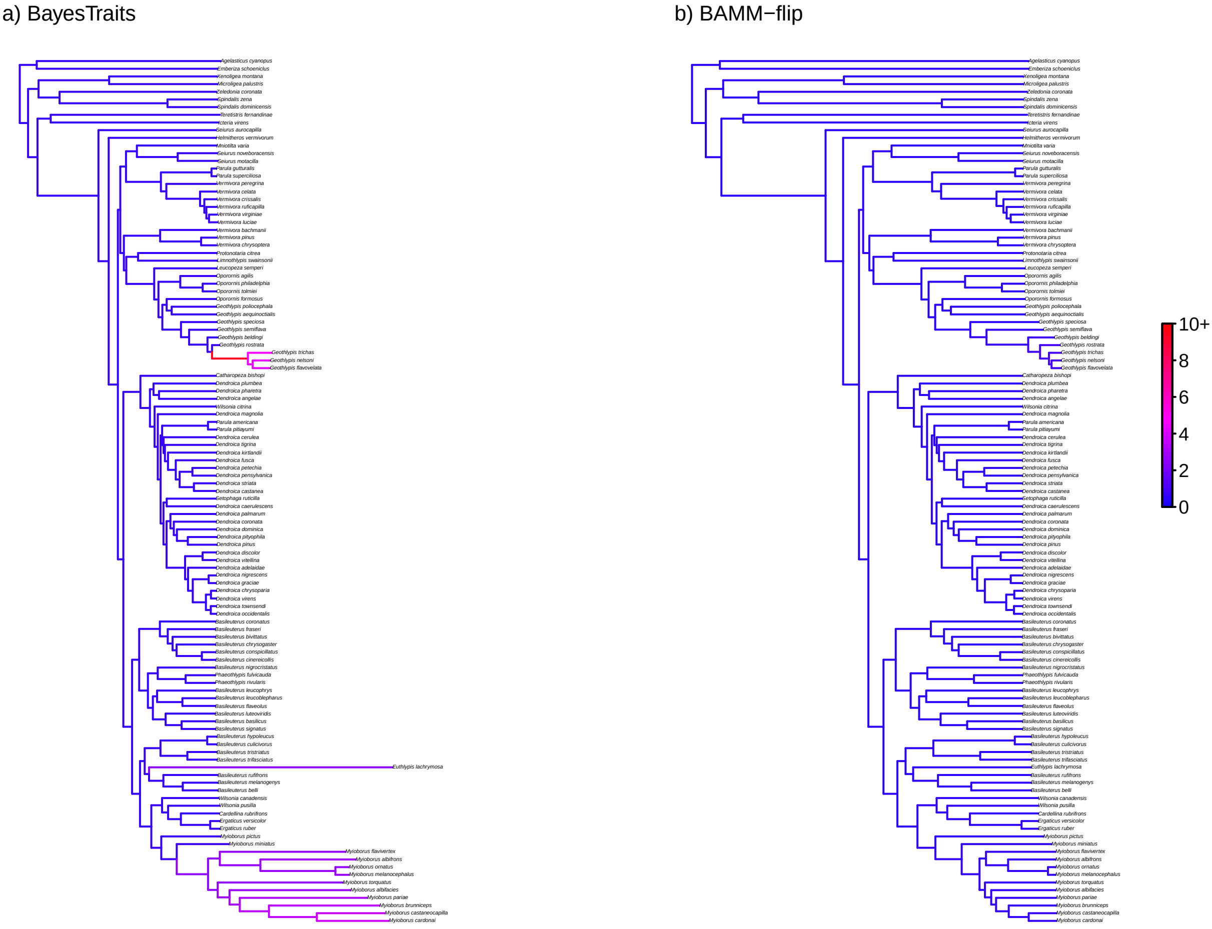

c)

	p_cvar	p_shgt	p_svar	p_sasr	AICw	sigma	alpha	r
BM	0.35 (+)	0.42 (−)	0.18 (−)	0.26 (+)	0.28	0.56		
OU	0.72 (+)	0.75 (+)	0.51 (−)	0.09 (+)	0.61	0.71	2.03	
EB	0.37 (+)	0.42 (−)	0.22 (+)	0.28 (+)	0.1	0.56		0
BayesTraits	0.08 (−)	0.47 (+)	0.17 (+)	0.85 (+)				
BAMM−flip	0.12 (−)	0.85 (−)	0.36 (+)	0.57 (+)				

Figure S75

Bulbuls, Allies

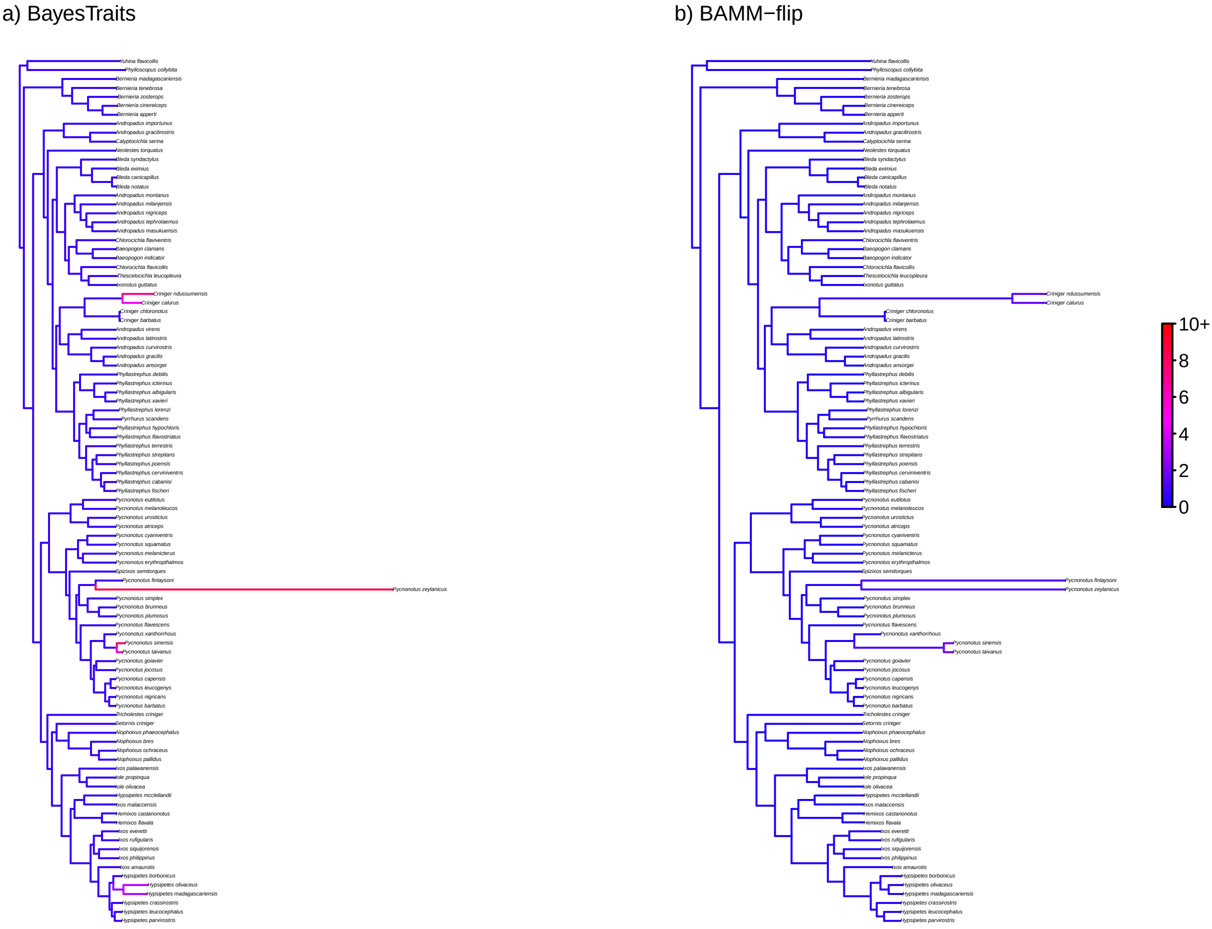

c)

	p_cvar	p_shgt	p_svar	p_sasr	AICw	sigma	alpha	r
BM	0 (+)	0.03 (−)	0 (−)	0.32 (+)	0.02	0.32		
OU	0 (+)	0.36 (−)	0 (−)	0.91 (+)	0.98	0.51	1.49	
EB	0 (+)	0.05 (+)	0 (+)	0.3 (+)	0.01	0.32		0
BayesTraits	0.67 (+)	0.77 (−)	0.69 (+)	0.77 (−)				
BAMM−flip	0.1 (+)	0.11 (−)	0.34 (−)	0.79 (+)				

Figure S76

Gulls, Terns, Auks, Crab Plover

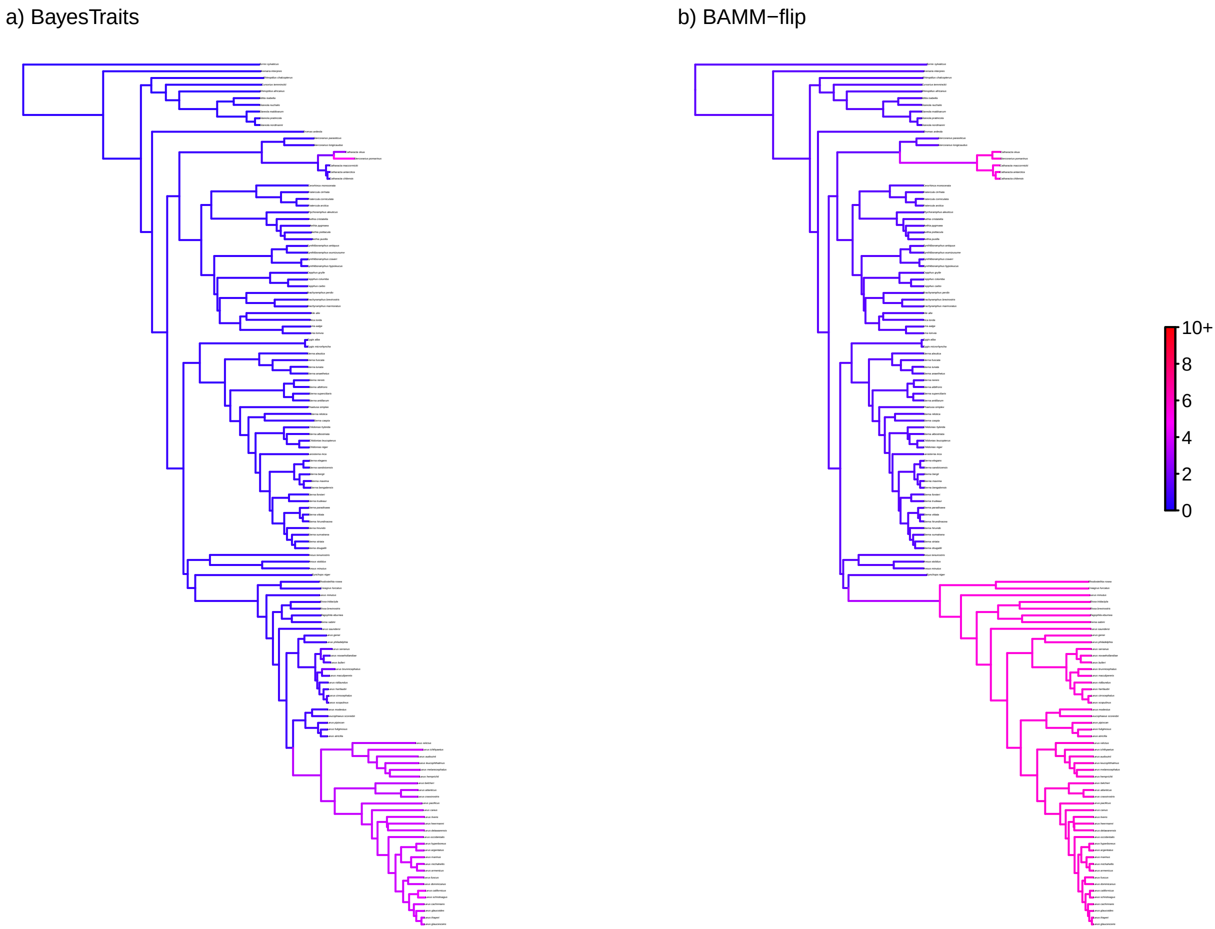

c)

	p_cvar	p_shgt	p_svar	p_sasr	AICw	sigma	alpha	r
BM	0 (+)	0 (−)	0 (−)	0 (+)	0	3.04		
OU	0.02 (+)	0.26 (−)	0 (−)	0 (+)	1	4.2	3.06	
EB	0 (+)	0 (−)	0 (−)	0 (+)	0	3.04		0
BayesTraits	0.82 (+)	0.21 (−)	0.22 (−)	0.1 (+)				
BAMM−flip	0.12 (+)	0.18 (−)	0.02 (−)	0.03 (+)				

Figure S77

Tanagers I, Allies

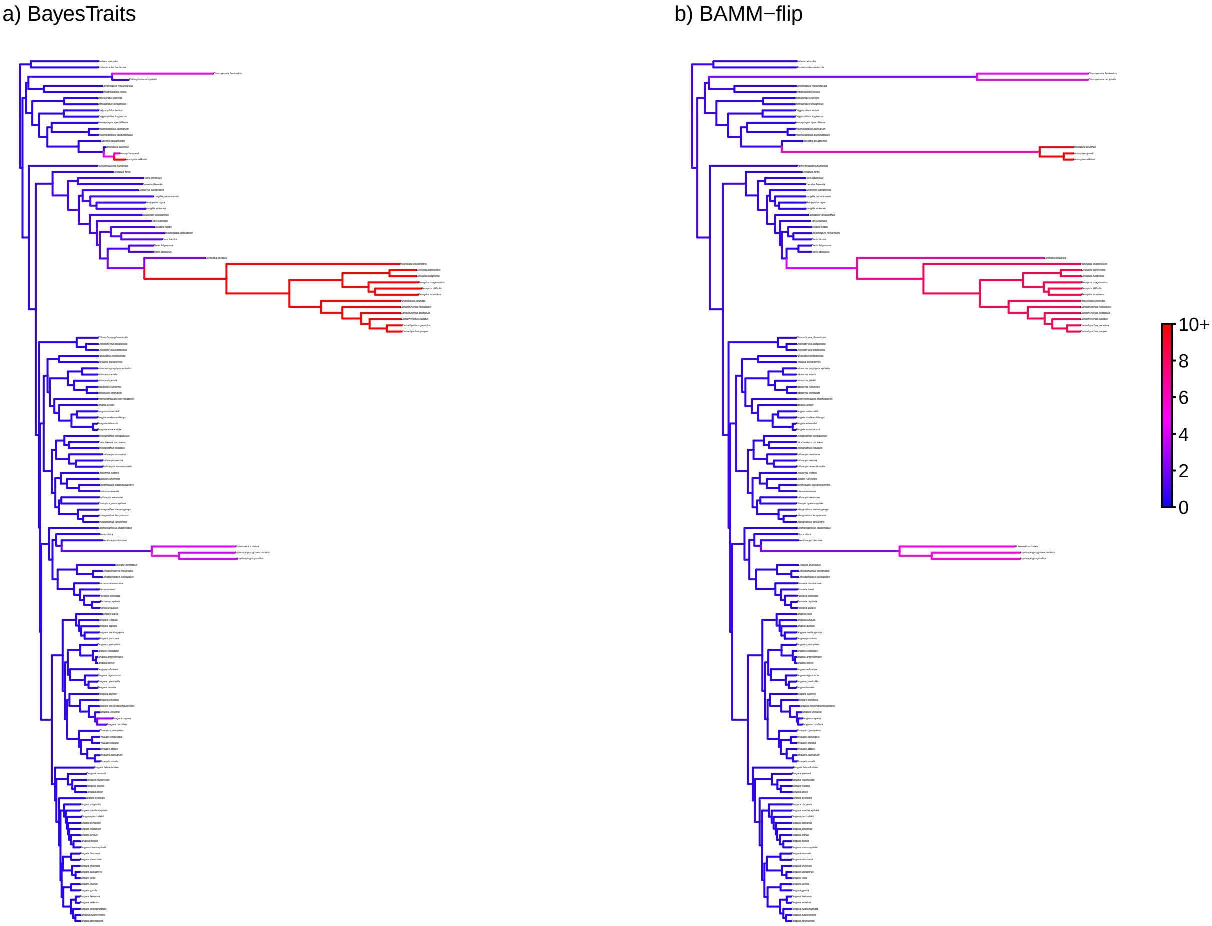

c)

	p_cvar	p_shgt	p_svar	p_sasr	AICw	sigma	alpha	r
BM	0 (+)	0 (−)	0 (−)	0.03 (−)	0	1.61		
OU	0 (+)	0.01 (−)	0 (−)	0.96 (−)	1	2.73	5.22	
EB	0 (+)	0 (+)	0 (+)	0.03 (+)	0	1.61		0
BayesTraits	0.6 (−)	0.61 (−)	0.41 (+)	0.38 (+)				
BAMM−flip	0.34 (+)	0.32 (−)	0.92 (−)	0.82 (+)				

Figure S78

Parrots III

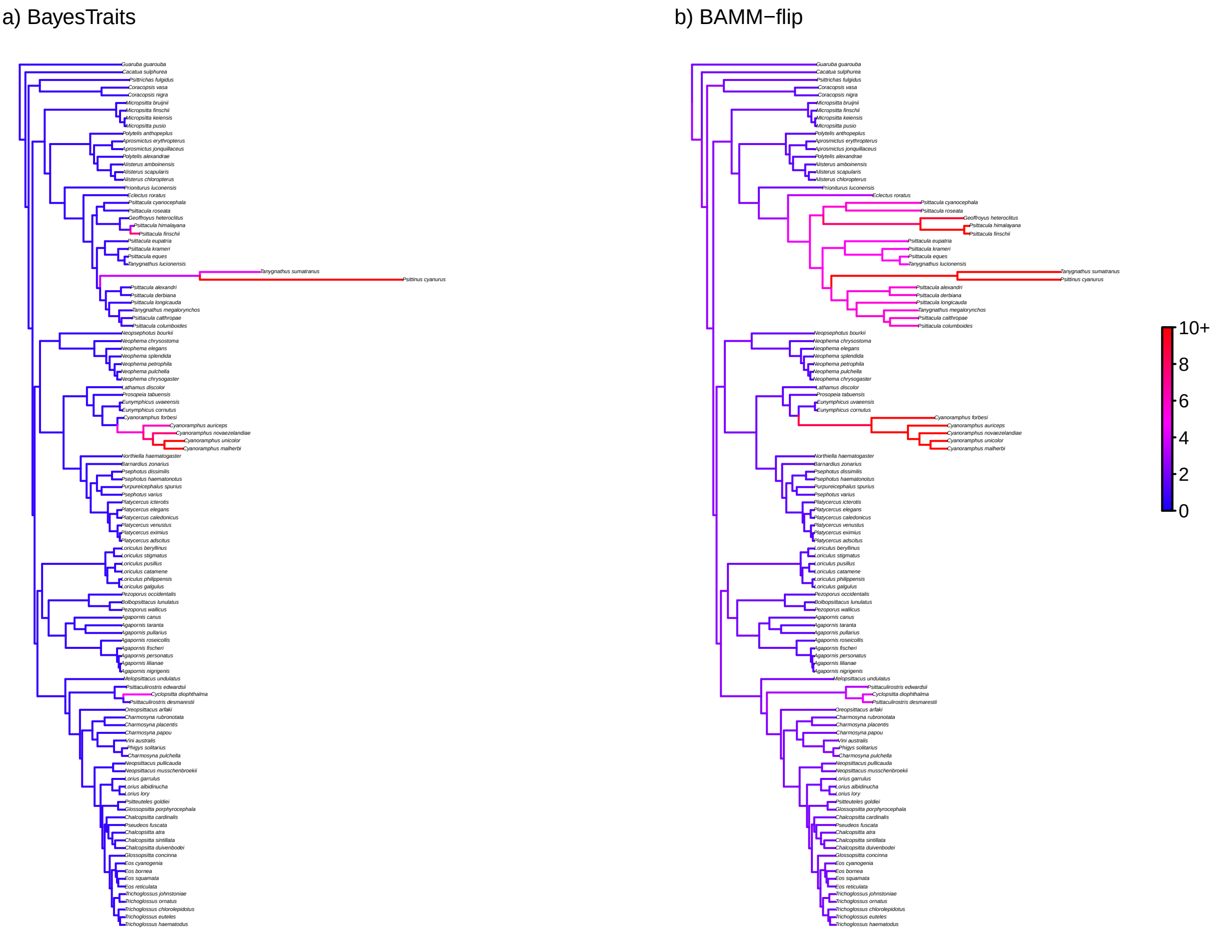

c)

	p_cvar	p_shgt	p_svar	p_sasr	AICw	sigma	alpha	r
BM	0 (+)	0.06 (−)	0 (−)	0.07 (+)	0	4.02		
OU	0 (+)	0.63 (+)	0.02 (−)	0.22 (+)	1	6.52	4.26	
EB	0 (+)	0.05 (+)	0 (+)	0.06 (+)	0	4.02		0
BayesTraits	0.32 (+)	0.42 (−)	0.53 (+)	0.02 (+)				
BAMM−flip	0.09 (+)	0.08 (−)	0.85 (−)	0.1 (+)				

Figure S79

Finches, Allies

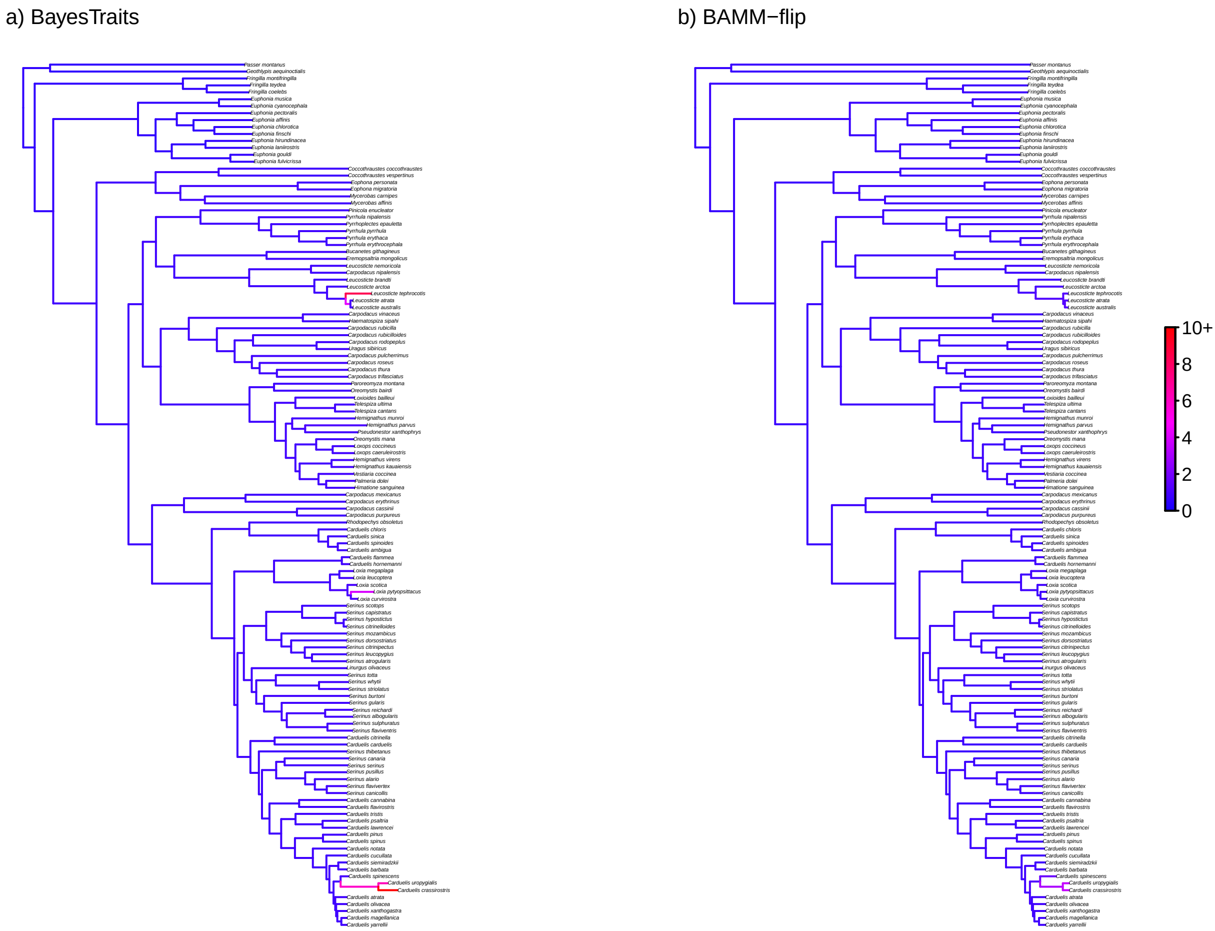

c)

	p_cvar	p_shgt	p_svar	p_sasr	AICw	sigma	alpha	r
BM	0 (+)	0.01 (−)	0 (−)	0.11 (+)	0.01	0.96		
OU	0 (+)	0.24 (−)	0 (−)	0.15 (+)	0.98	1.29	2.19	
EB	0 (+)	0.01 (−)	0 (−)	0.12 (+)	0.01	0.96		0
BayesTraits	0.79 (+)	0.06 (−)	0.1 (−)	0.4 (+)				
BAMM−flip	0.05 (+)	0.04 (−)	0.01 (−)	0.12 (+)				

Figure S80

Tanagers II, Flowerpiercers, Conebills, Seedeaters, Warbling−finches, Allies

a) BayesTraits

b) BAMM−flip

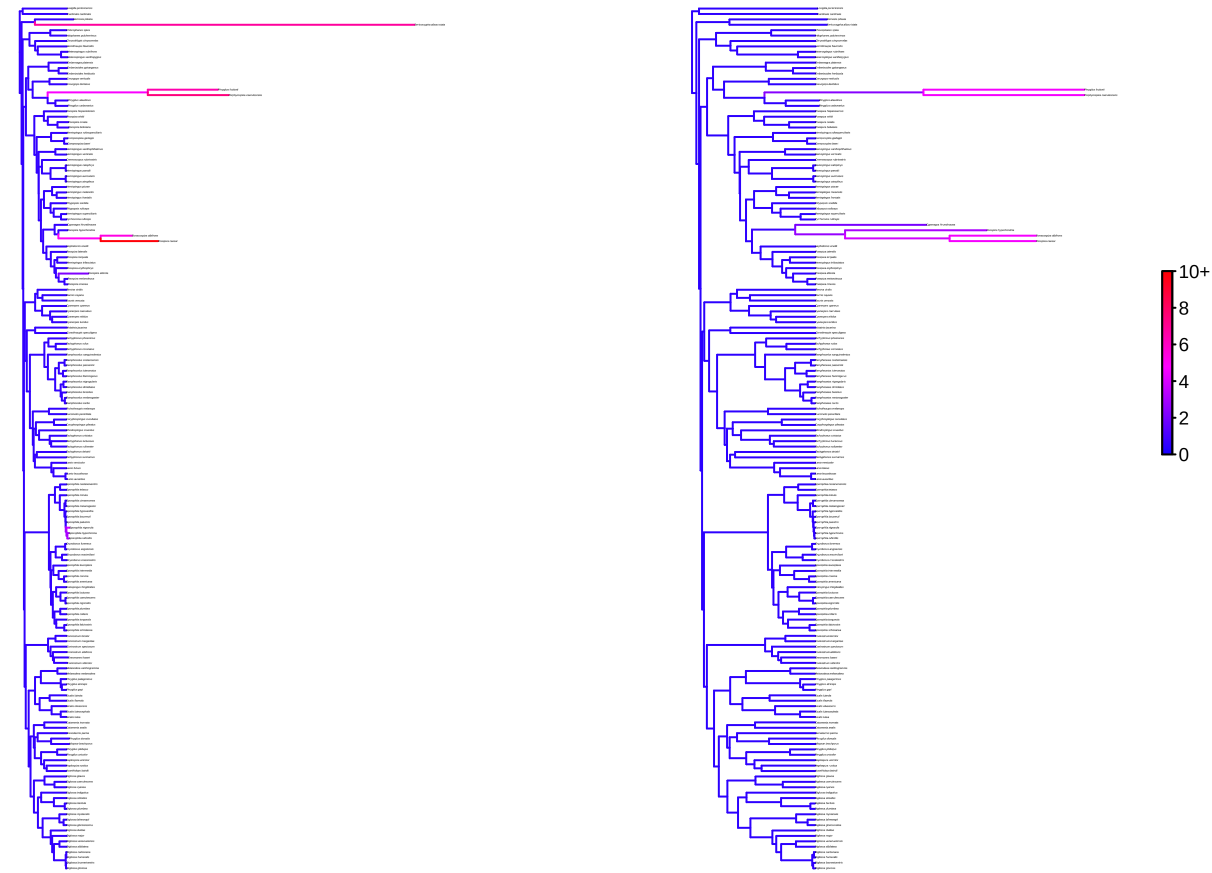

c)

	p_cvar	p_shgt	p_svar	p_sasr	AICw	sigma	alpha	r
BM	0 (+)	0.49 (−)	0.21 (−)	0.08 (+)	0.06	0.67		
OU	0 (+)	0.2 (+)	0.56 (−)	0.05 (+)	0.92	0.95	1.84	
EB	0 (+)	0.51 (−)	0.2 (−)	0.08 (+)	0.02	0.67		0
BayesTraits	0.99 (−)	0.41 (−)	0.37 (+)	0.41 (+)				
BAMM−flip	0.21 (+)	0.43 (−)	0.75 (−)	0.53 (+)				

Figure S81

Hawks, Eagles, Secretarybird

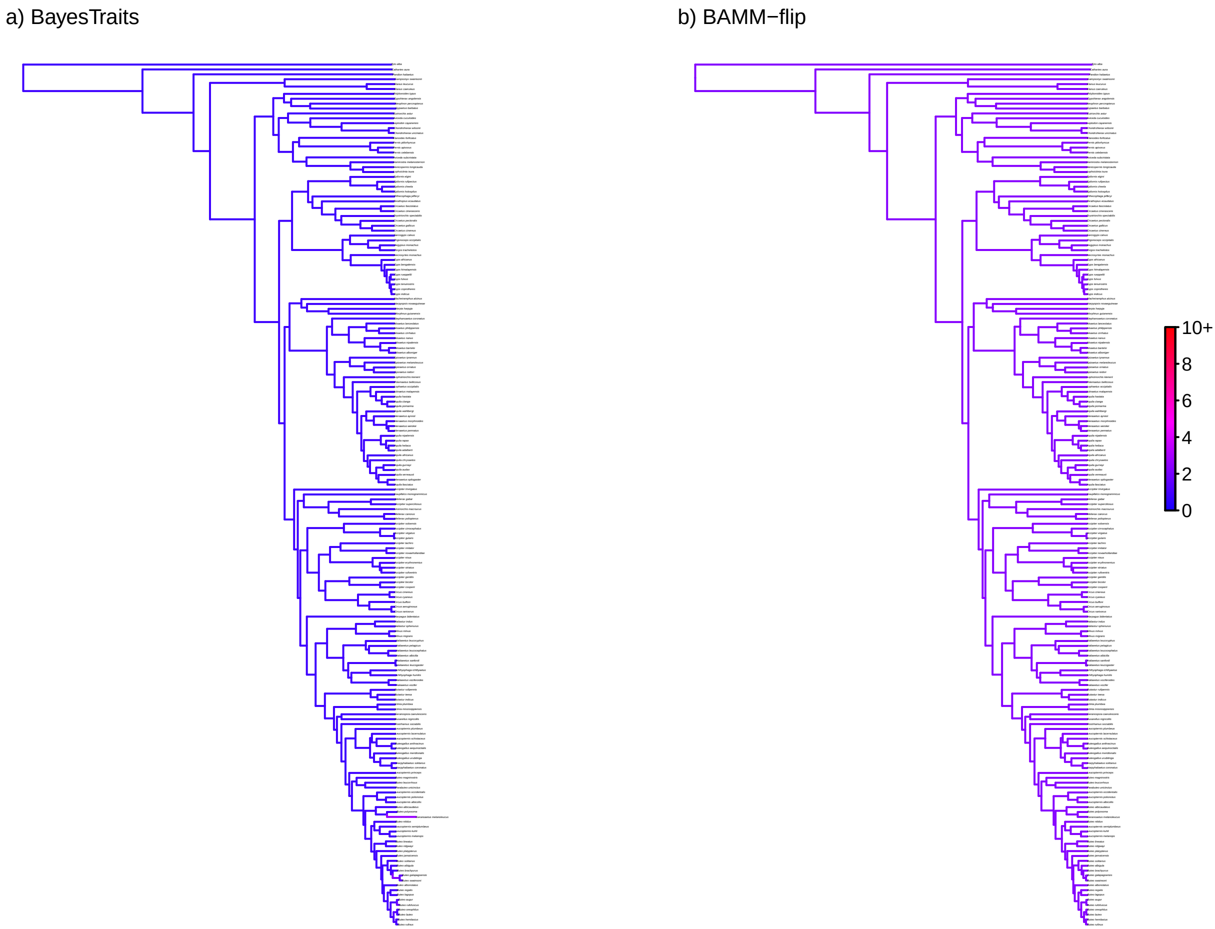

c)

	p_cvar	p_shgt	p_svar	p_sasr	AICw	sigma	alpha	r
BM	0.96 (−)	0.37 (−)	0.04 (−)	0.08 (+)	0.4	2.26		
OU	0.87 (−)	0.69 (+)	0.16 (−)	0.11 (+)	0.45	2.58	0.69	
EB	0.98 (+)	0.38 (−)	0.04 (−)	0.08 (−)	0.15	2.26		0
BayesTraits	0.59 (−)	0.63 (−)	0.17 (−)	0.04 (+)				
BAMM−flip	0.99 (+)	0.33 (−)	0.04 (−)	0.06 (+)				

Figure S82

Chats, Old World Flycatchers

a) BayesTraits

b) BAMM−flip

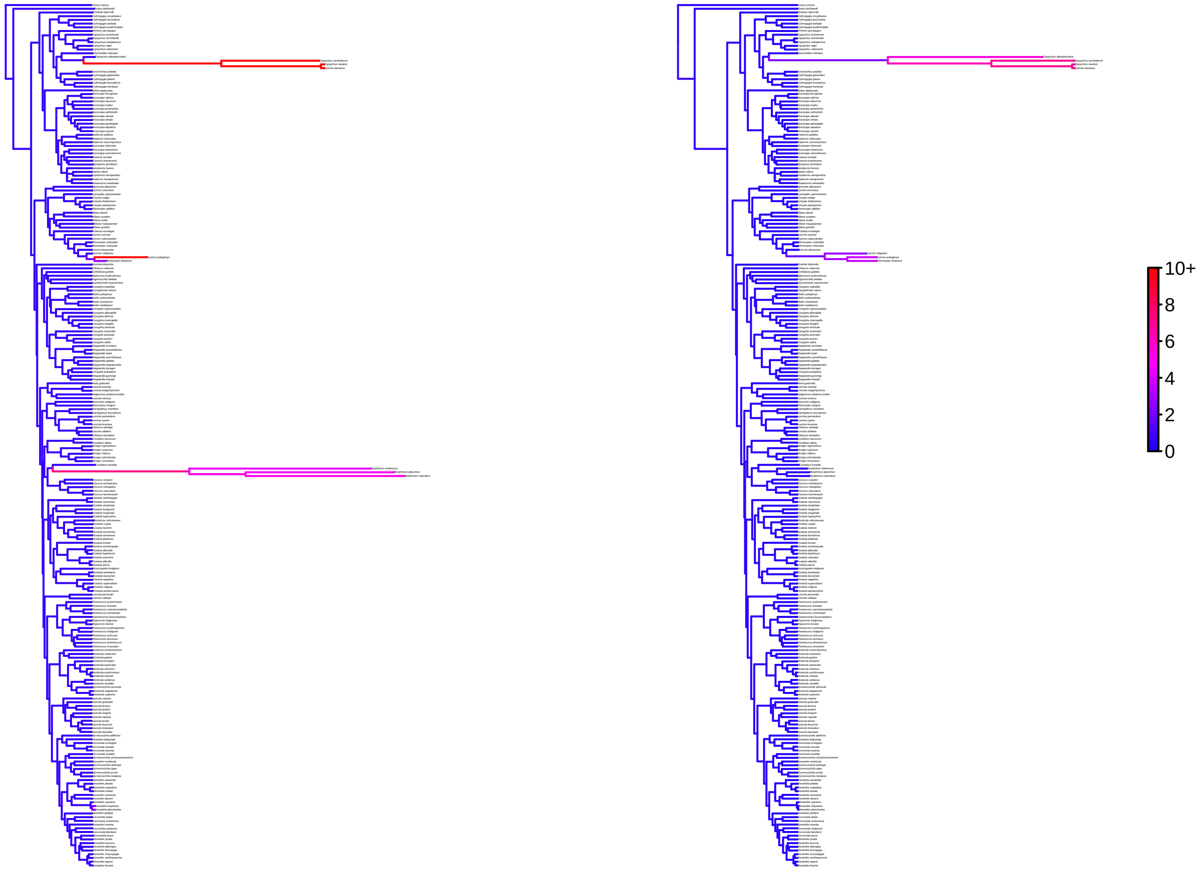

c)

	p_cvar	*p_shgt*	*p_svar*	*p_sasr*	*AICw*	*sigma*	*alpha*	*r*
BM	0 (+)	0.15 (−)	0 (−)	0 (+)	0.01	0.59		
OU	0 (+)	0.86 (−)	0 (−)	0 (+)	0.99	0.81	1.31	
EB	0 (−)	0.16 (−)	0 (−)	0 (+)	0	0.59		0
BayesTraits	0.17 (+)	0.6 (−)	0.45 (−)	0.11 (+)				
BAMM−flip	0.04 (+)	0.19 (−)	0.11 (−)	0.01 (+)				

Figure S83

Ovenbirds, Woodcreepers

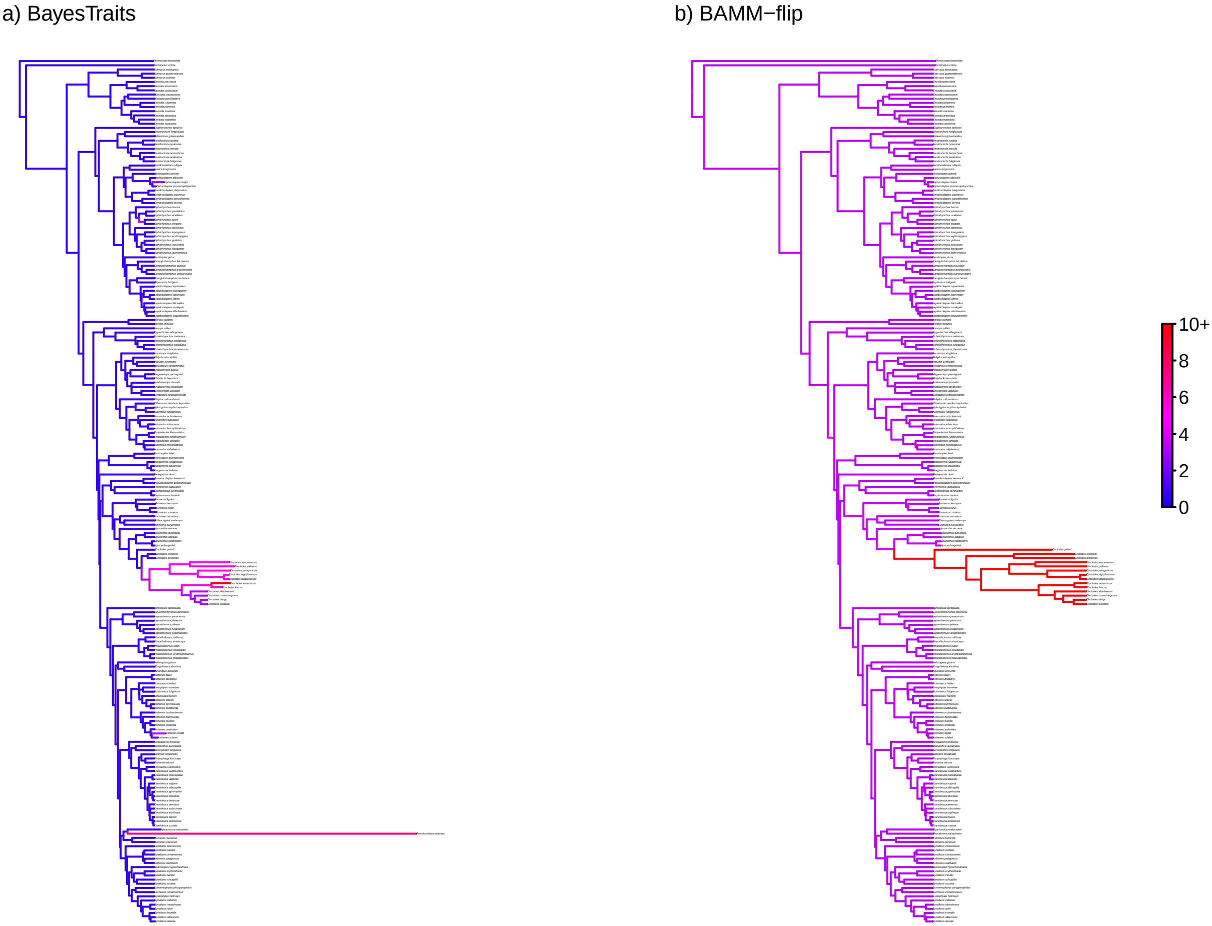

c)

	p_cvar	p_shgt	p_svar	p_sasr	AICw	sigma	alpha	r
BM	0 (+)	0.47 (−)	0.07 (−)	0.05 (+)	0.05	4.35		
OU	0 (+)	0.27 (+)	0.3 (−)	0.13 (+)	0.93	5.55	7.86	
EB	0 (+)	0.44 (−)	0.06 (−)	0.04 (+)	0.02	4.35		0
BayesTraits	0.13 (+)	0.56 (+)	0.25 (+)	0.69 (+)				
BAMM−flip	0 (+)	0.99 (+)	0.8 (−)	0.55 (+)				

Figure S84

Whiteyes, Babblers I, Parrotbills

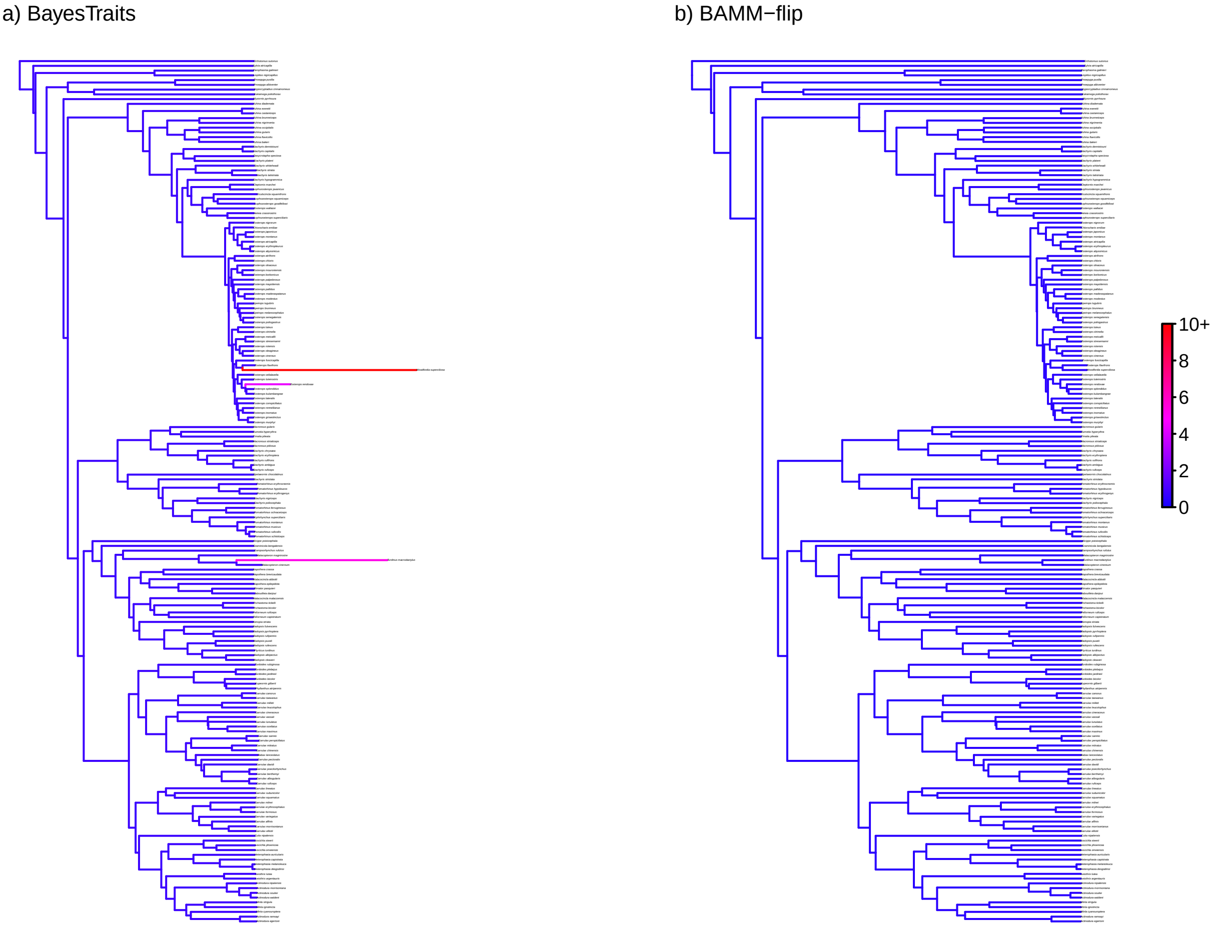

c)

	p_cvar	p_shgt	p_svar	p_sasr	AICw	sigma	alpha	r
BM	0.02 (+)	0.2 (−)	0.04 (−)	0.96 (+)	0.31	0.45		
OU	0.04 (+)	0.97 (+)	0.22 (−)	0.89 (+)	0.57	0.52	0.49	
EB	0.04 (+)	0.21 (−)	0.05 (−)	0.94 (+)	0.11	0.45		0
BayesTraits	0.32 (+)	0.94 (−)	0.84 (+)	0.57 (+)				
BAMM−flip	0.12 (+)	0.48 (−)	0.33 (−)	0.68 (+)				

Figure S85

Tyrant−Flycatchers, Cotingas, Allies

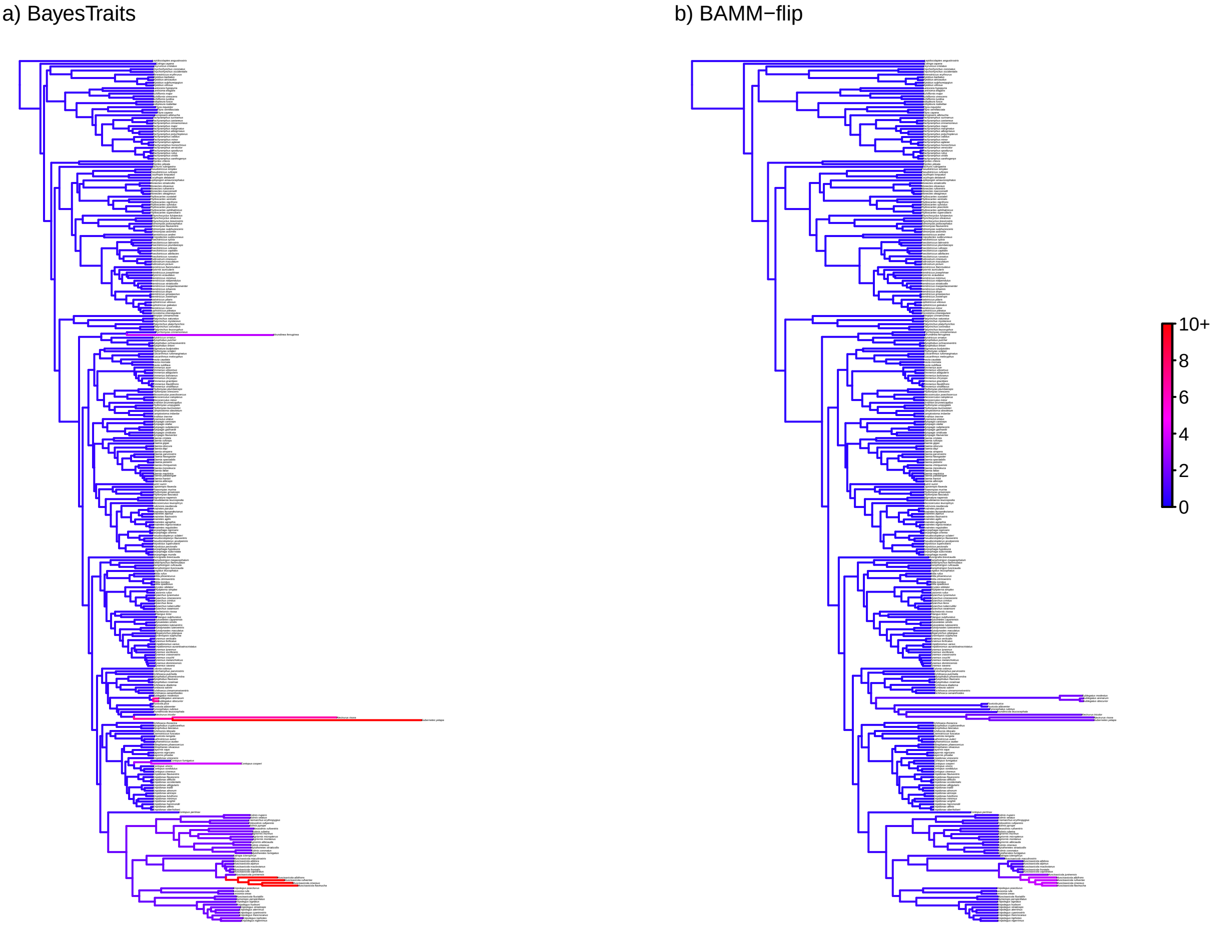

c)

	p_cvar	p_shgt	p_svar	p_sasr	AICw	sigma	alpha	r
BM	0 (+)	0.33 (−)	0 (−)	0 (+)	0.19	0.57		
OU	0 (+)	0.74 (+)	0 (−)	0 (+)	0.74	0.65	0.65	
EB	0 (+)	0.33 (−)	0 (−)	0 (+)	0.07	0.57		0
BayesTraits	0.17 (+)	0.4 (+)	0.91 (+)	0.09 (+)				
BAMM−flip	0.02 (+)	0.71 (−)	0.3 (−)	0.03 (+)				

Figure S86

Gnateaters, Allies

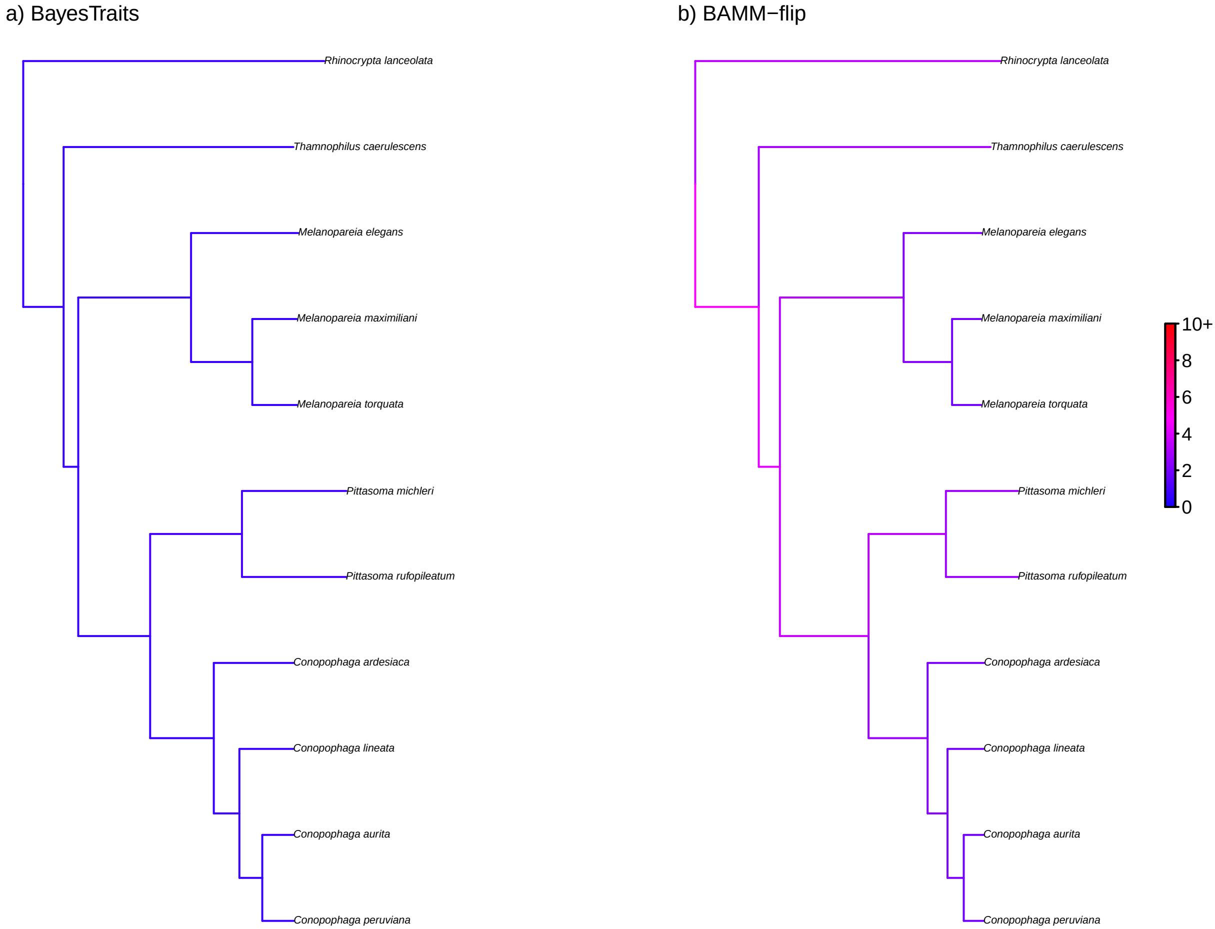

c)

	p_cvar	*p_shgt*	*p_svar*	*p_sasr*	*AICw*	*sigma*	*alpha*	*r*
BM	0.02 (+)	0.04 (+)	0.04 (+)	0.48 (+)	0.33	2.36		
OU	0.03 (−)	0.04 (+)	0.05 (+)	0.45 (−)	0.12	2.36	0	
EB	0.06 (+)	0.81 (−)	0.92 (+)	0.27 (+)	0.54	51.9		−29.88
BayesTraits	0.03 (+)	0.04 (+)	0.04 (+)	0.5 (+)				
BAMM−flip	0.1 (+)	0.09 (+)	0.12 (+)	0.96 (−)				

Figure S87

Berrypeckers, Satinbirds, Allies

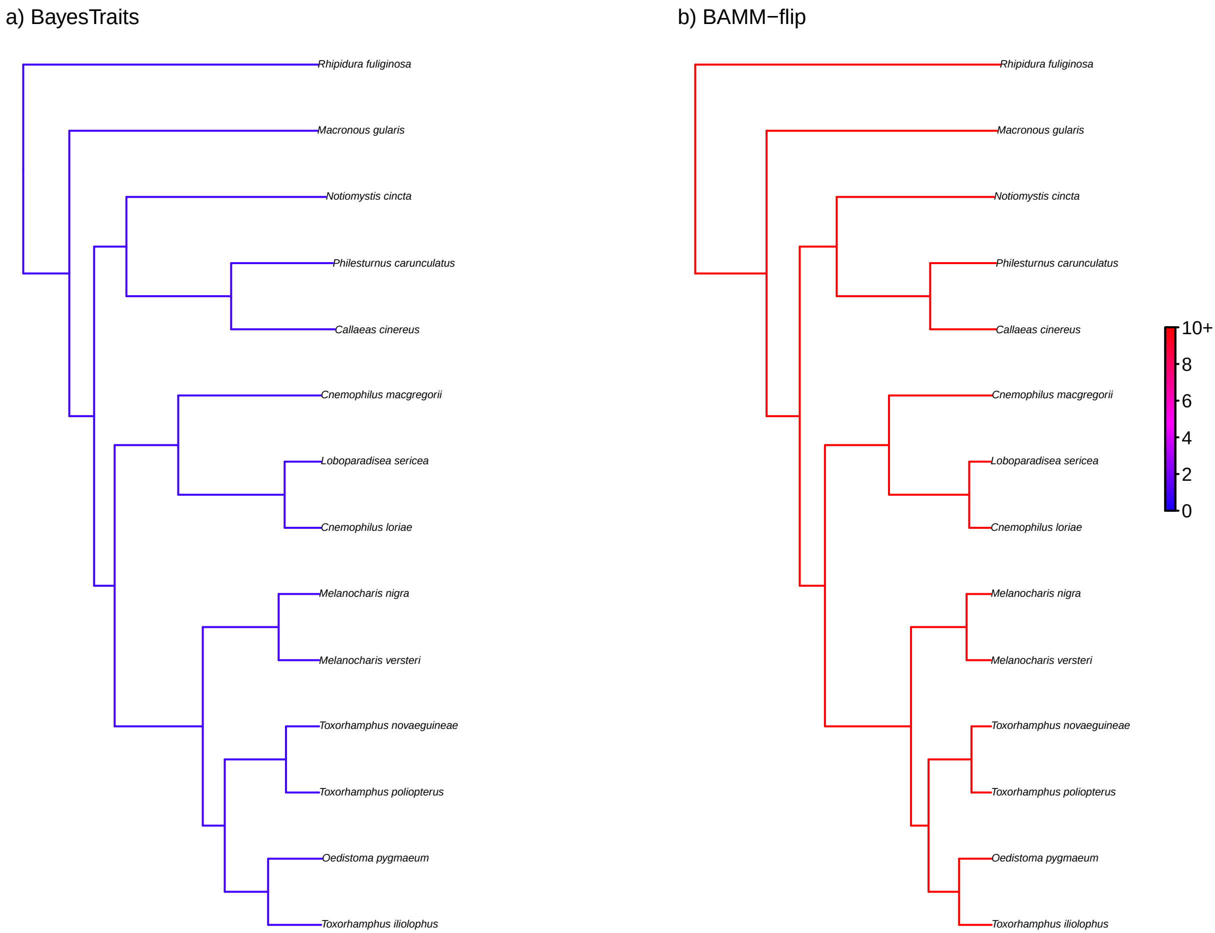

c)

	p_cvar	*p_shgt*	*p_svar*	*p_sasr*	*AICw*	*sigma*	*alpha*	*r*
BM	0.72 (+)	0.04 (+)	0.09 (+)	0.77 (+)	0.36	11.77		
OU	0.77 (+)	0.02 (+)	0.09 (+)	0.81 (−)	0.13	11.77	0	
EB	0.83 (−)	0.56 (+)	0.5 (+)	0.8 (+)	0.5	76.67		−32.56
BayesTraits	0.8 (+)	0.03 (+)	0.07 (+)	0.76 (+)				
BAMM−flip	0.81 (+)	0.06 (+)	0.1 (+)	0.91 (+)				

Figure S88

Grebes

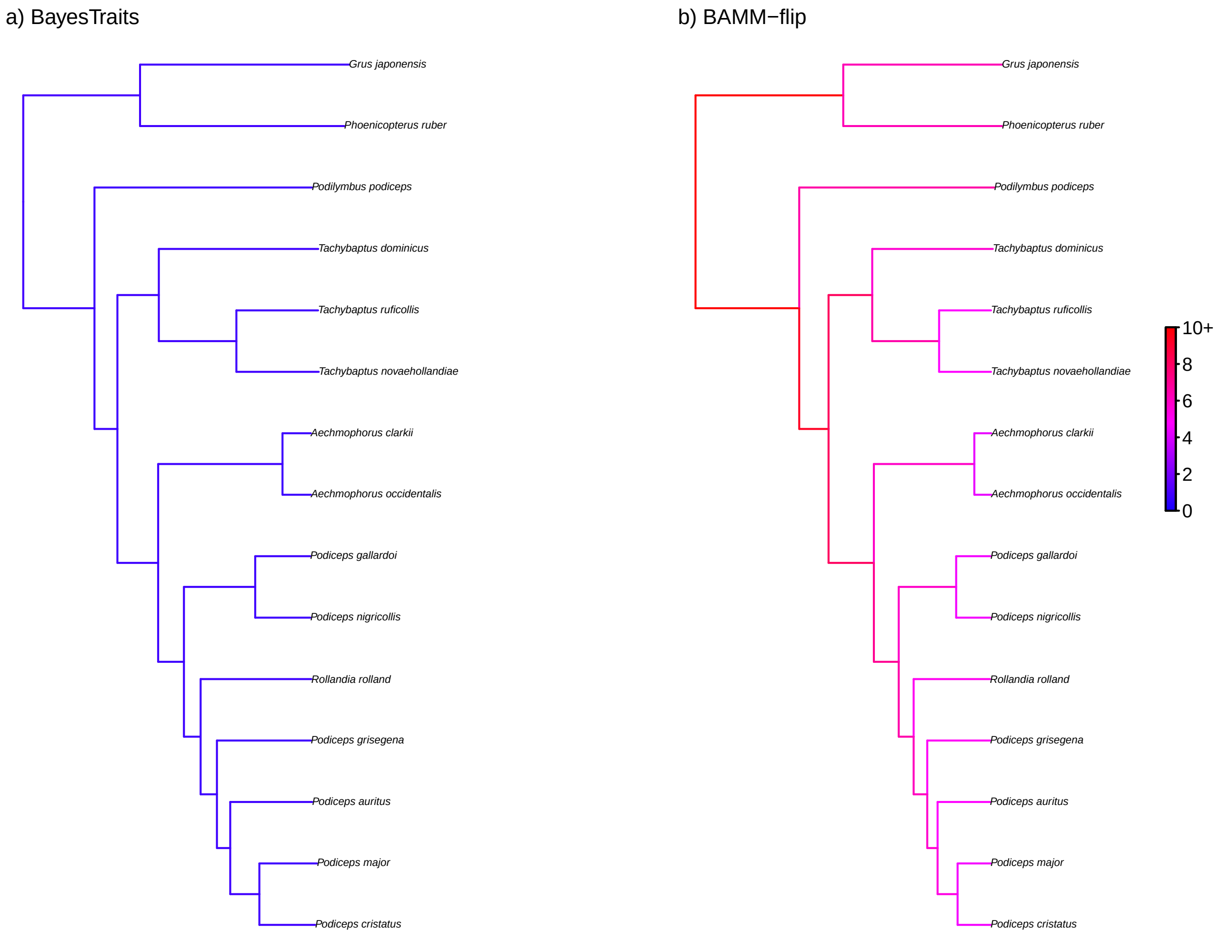

c)

	p_cvar	*p_shgt*	*p_svar*	*p_sasr*	*AICw*	*sigma*	*alpha*	*r*
BM	0.65 (+)	0.06 (+)	0.3 (+)	0.5 (+)	0.41	5.15		
OU	0.64 (−)	0.07 (+)	0.29 (−)	0.52 (−)	0.15	5.15	0	
EB	0.71 (−)	0.5 (+)	0.63 (+)	0.42 (+)	0.44	23.06		−10.89
BayesTraits	0.7 (+)	0.07 (+)	0.24 (+)	0.6 (+)				
BAMM−flip	0.78 (+)	0.1 (+)	0.34 (+)	0.62 (+)				

Figure S89

Australasian Wrens

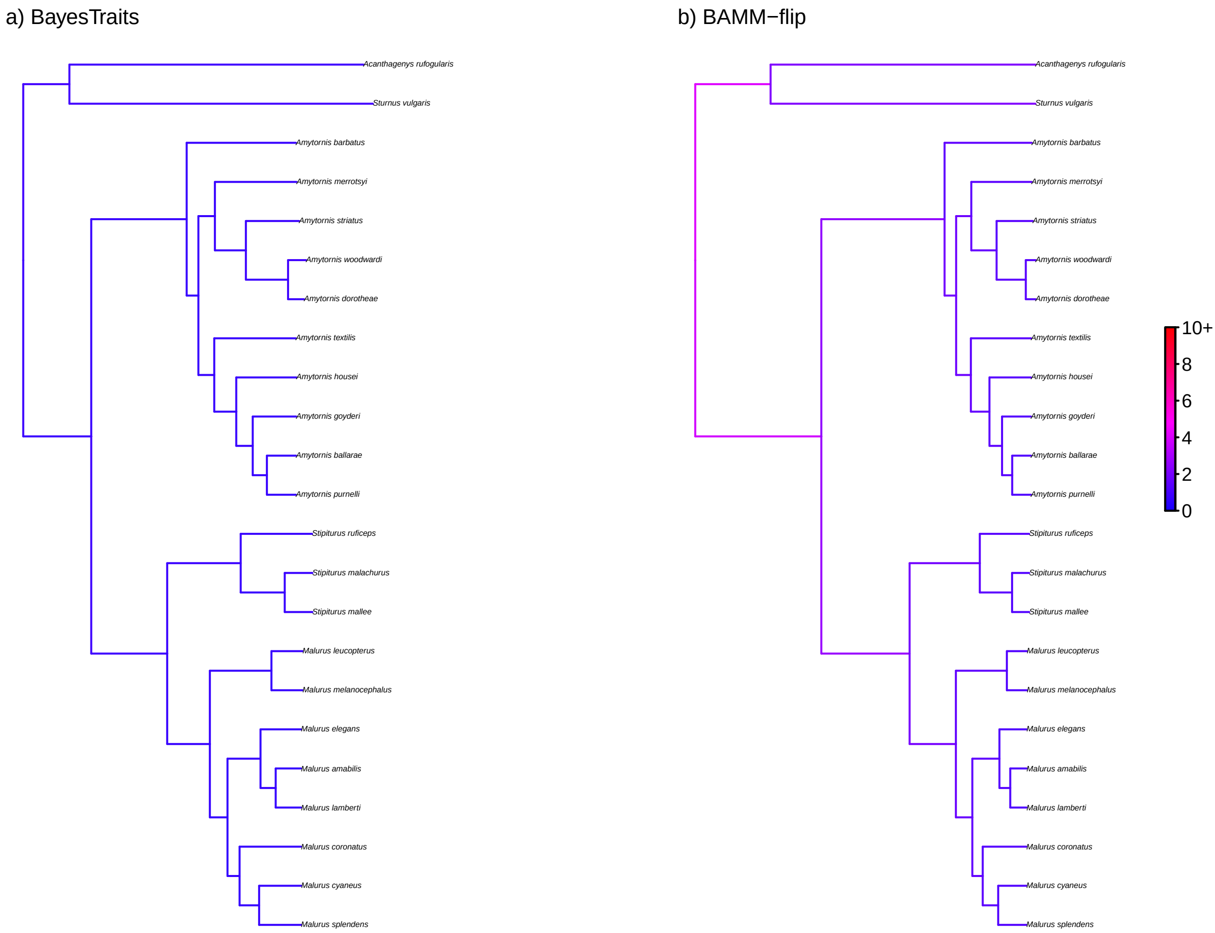

c)

	p_cvar	*p_shgt*	*p_svar*	*p_sasr*	*AICw*	*sigma*	*alpha*	*r*
BM	0.16 (+)	0.02 (+)	0.2 (+)	0.52 (+)	0.31	1.56		
OU	0.14 (−)	0.02 (+)	0.22 (+)	0.51 (+)	0.12	1.56	0	
EB	0.38 (+)	0.21 (+)	0.5 (+)	0.49 (+)	0.57	6.55		−8.99
BayesTraits	0.24 (+)	0.03 (+)	0.17 (+)	0.63 (+)				
BAMM−flip	0.57 (+)	0.06 (+)	0.23 (+)	0.83 (+)				

Figure S90

Pheasants, Quail, Guineafowl

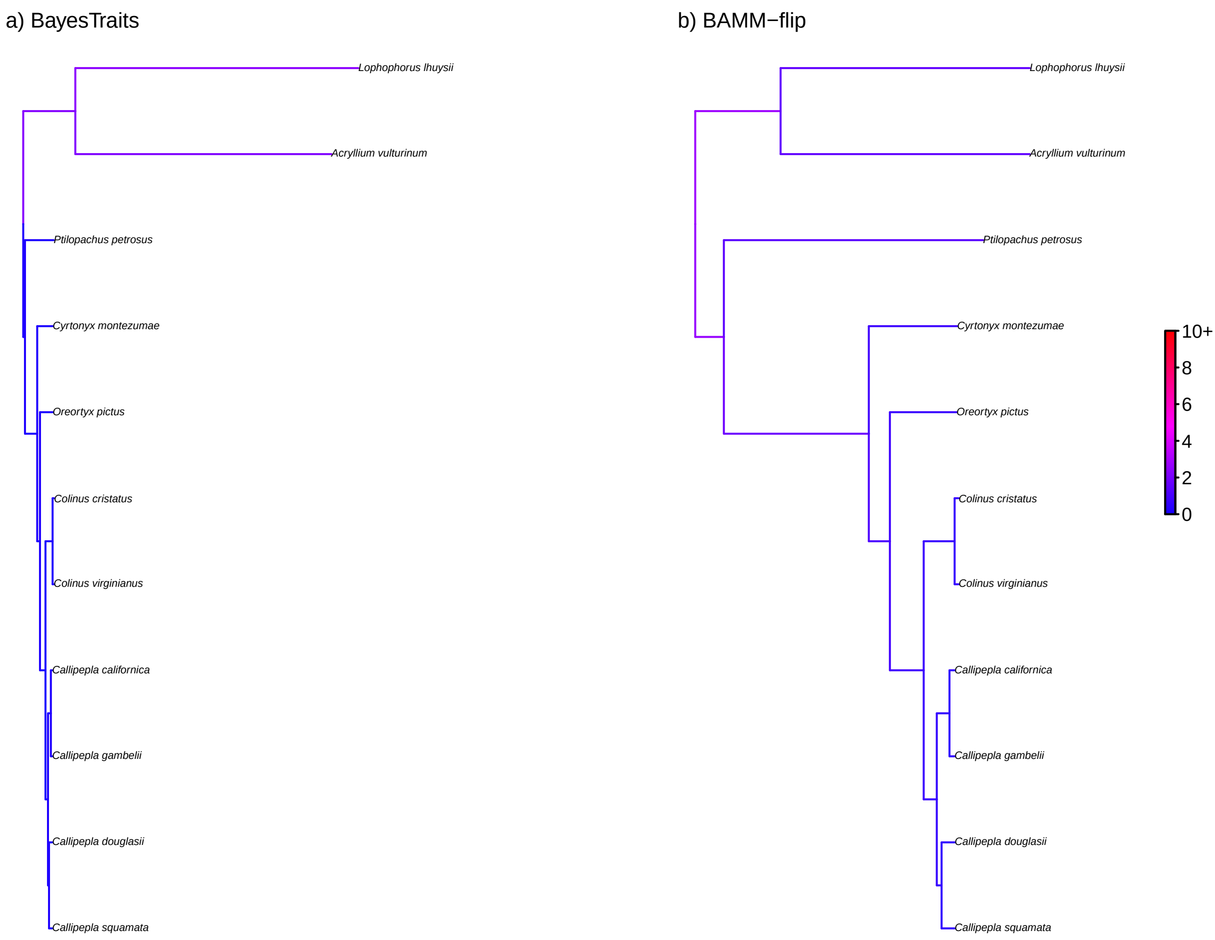

c)

	p_cvar	*p_shgt*	*p_svar*	*p_sasr*	*AICw*	*sigma*	*alpha*	*r*
BM	0 (+)	0.06 (+)	0.33 (+)	0.4 (+)	0.19	1.05		
OU	0 (+)	0.06 (+)	0.4 (+)	0.39 (+)	0.07	1.05	0	
EB	0.01 (+)	0.34 (+)	0.82 (+)	0.67 (+)	0.73	5.39		−4.81
BayesTraits	0.05 (+)	0.44 (+)	0.4 (+)	0.89 (−)				
BAMM−flip	0.02 (+)	0.2 (+)	0.38 (+)	0.97 (+)				

Figure S91

Australasian Robins

a) BayesTraits

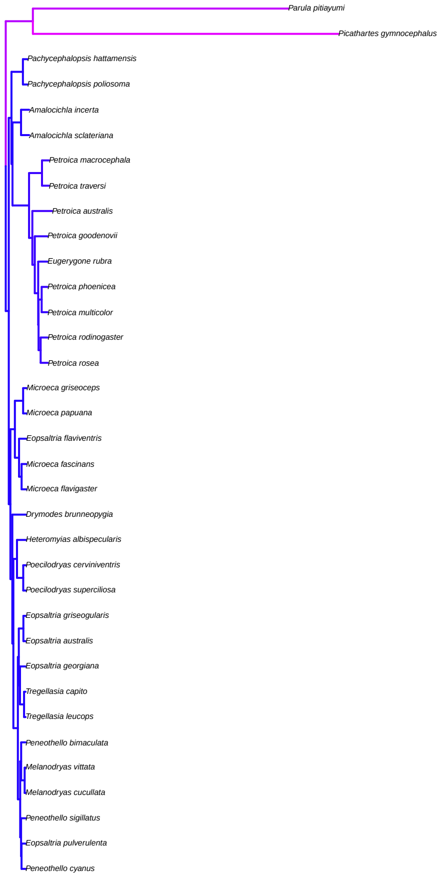

b) BAMM−flip

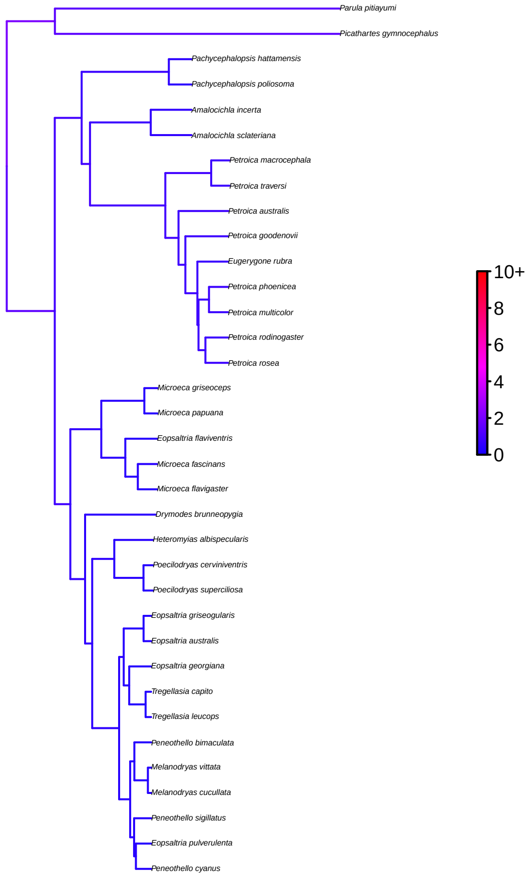

c)

	p_cvar	*p_shgt*	*p_svar*	*p_sasr*	*AICw*	*sigma*	*alpha*	*r*
BM	0 (+)	0.05 (+)	0.01 (+)	0.23 (+)	0.41	0.64		
OU	0 (+)	0.05 (+)	0.02 (+)	0.23 (+)	0.15	0.64	0	
EB	0 (+)	0.33 (+)	0.23 (−)	0.88 (+)	0.44	2.53		−2.37
BayesTraits	0.67 (+)	0.64 (−)	0.29 (+)	0.75 (+)				
BAMM−flip	0.25 (+)	0.94 (+)	0.06 (+)	0.88 (+)				

Figure S92

Antpittas

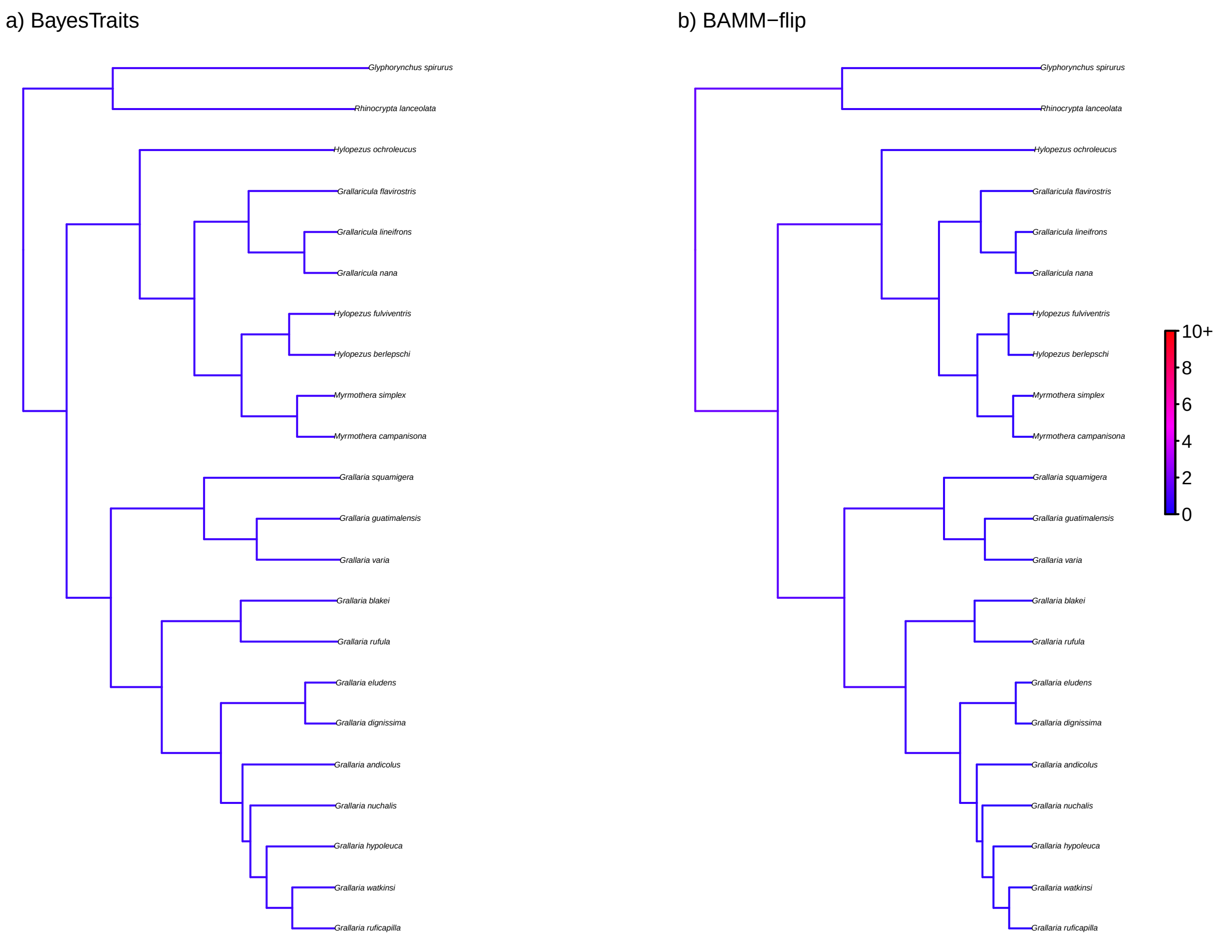

c)

	p_cvar	*p_shgt*	*p_svar*	*p_sasr*	*AICw*	*sigma*	*alpha*	*r*
BM	0.72 (+)	0 (+)	0.01 (+)	0.9 (−)	0.19	0.65		
OU	0.71 (+)	0.02 (+)	0.03 (+)	0.91 (−)	0.07	0.65	0	
EB	0.52 (+)	0.93 (+)	0.78 (+)	0.7 (+)	0.74	9.63		−7.44
BayesTraits	0.72 (+)	0.01 (+)	0.01 (+)	0.91 (−)				
BAMM−flip	0.87 (+)	0.02 (+)	0.01 (+)	0.88 (−)				

Figure S93

Cardinals, Allies

a) BayesTraits

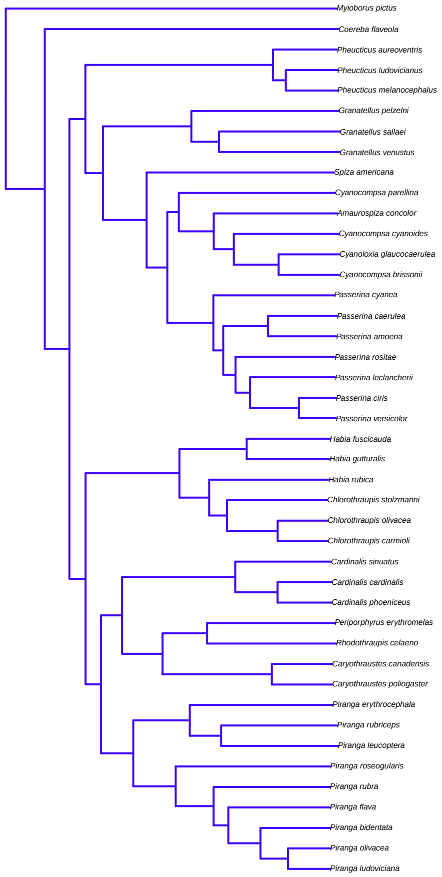

b) BAMM−flip

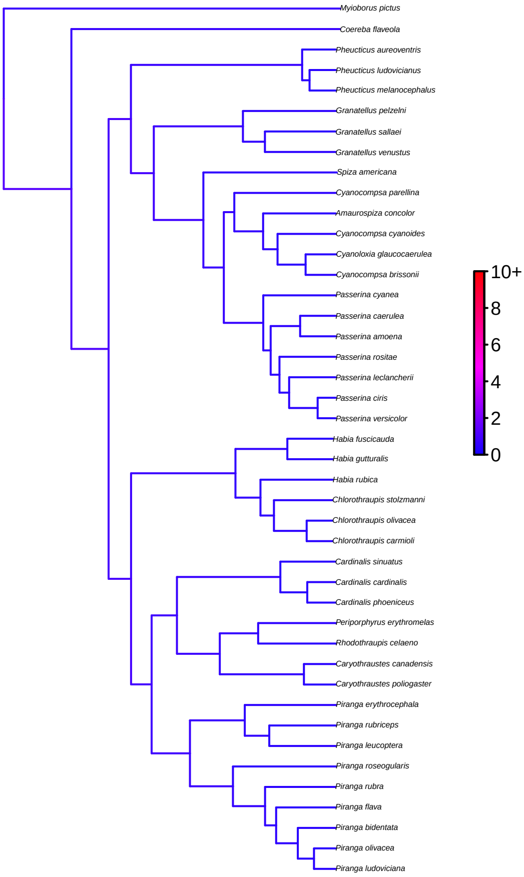

c)

	p_cvar	*p_shgt*	*p_svar*	*p_sasr*	*AICw*	*sigma*	*alpha*	*r*
BM	0.42 (+)	0.02 (+)	0.03 (+)	0.21 (−)	0.24	0.51		
OU	0.4 (+)	0 (+)	0.03 (+)	0.25 (−)	0.09	0.51	0	
EB	0.61 (+)	0.26 (−)	0.29 (−)	0.19 (+)	0.67	2.44		−4.94
BayesTraits	0.49 (+)	0 (+)	0.02 (+)	0.25 (−)				
BAMM−flip	0.59 (+)	0.02 (+)	0.02 (+)	0.23 (−)				

Figure S94

Vireos, Allies

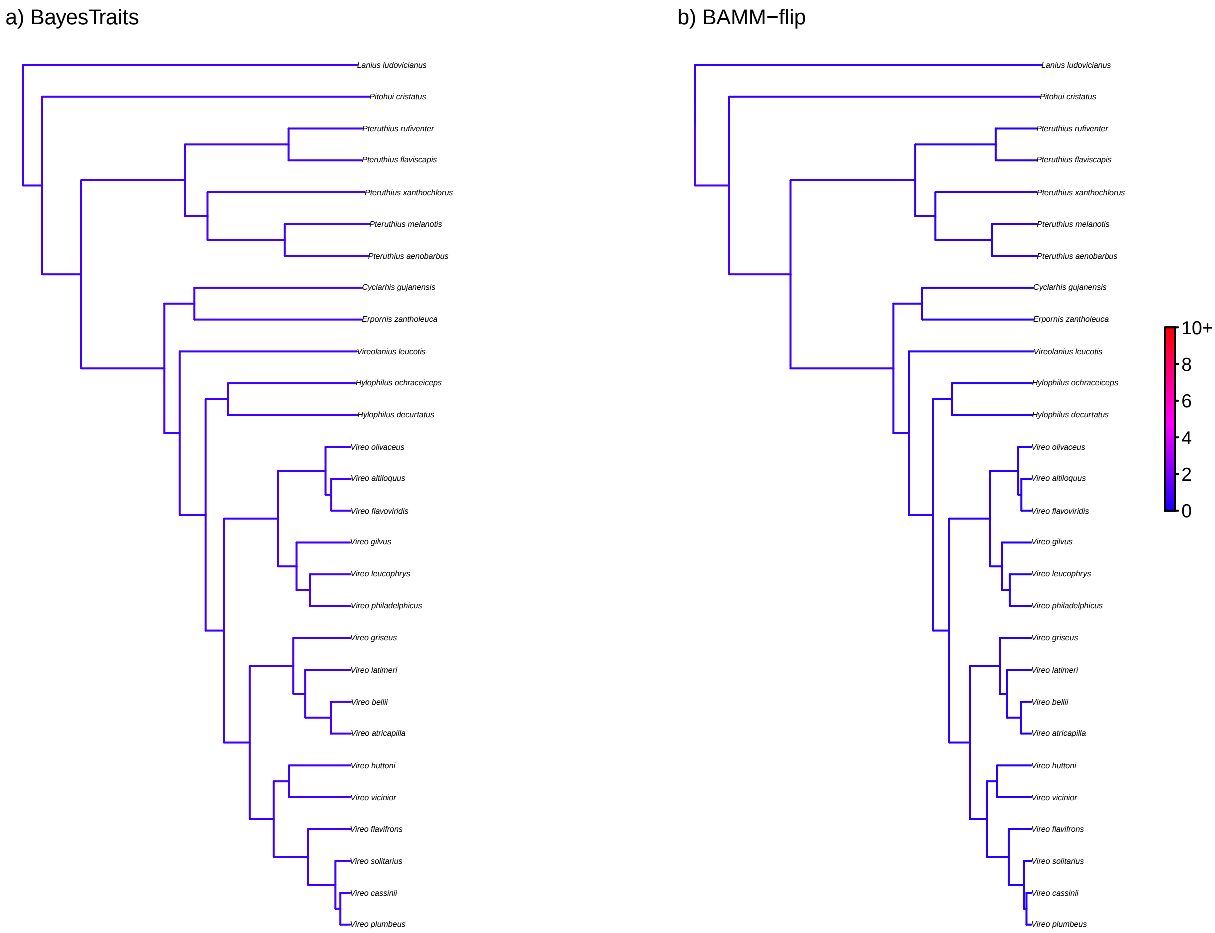

c)

	p_cvar	*p_shgt*	*p_svar*	*p_sasr*	*AICw*	*sigma*	*alpha*	*r*
BM	0.64 (−)	0.02 (+)	0.01 (+)	0.19 (+)	0.29	0.4		
OU	0.59 (−)	0.02 (+)	0.01 (+)	0.2 (+)	0.11	0.4	0	
EB	0.34 (+)	0.83 (+)	0.51 (+)	0.51 (−)	0.6	2.75		−3.39
BayesTraits	0.53 (−)	0.01 (+)	0 (+)	0.2 (+)				
BAMM−flip	0.38 (−)	0.04 (+)	0.02 (+)	0.23 (+)				

Figure S95

Tits, Chickadees, Penduline−Tits, Allies

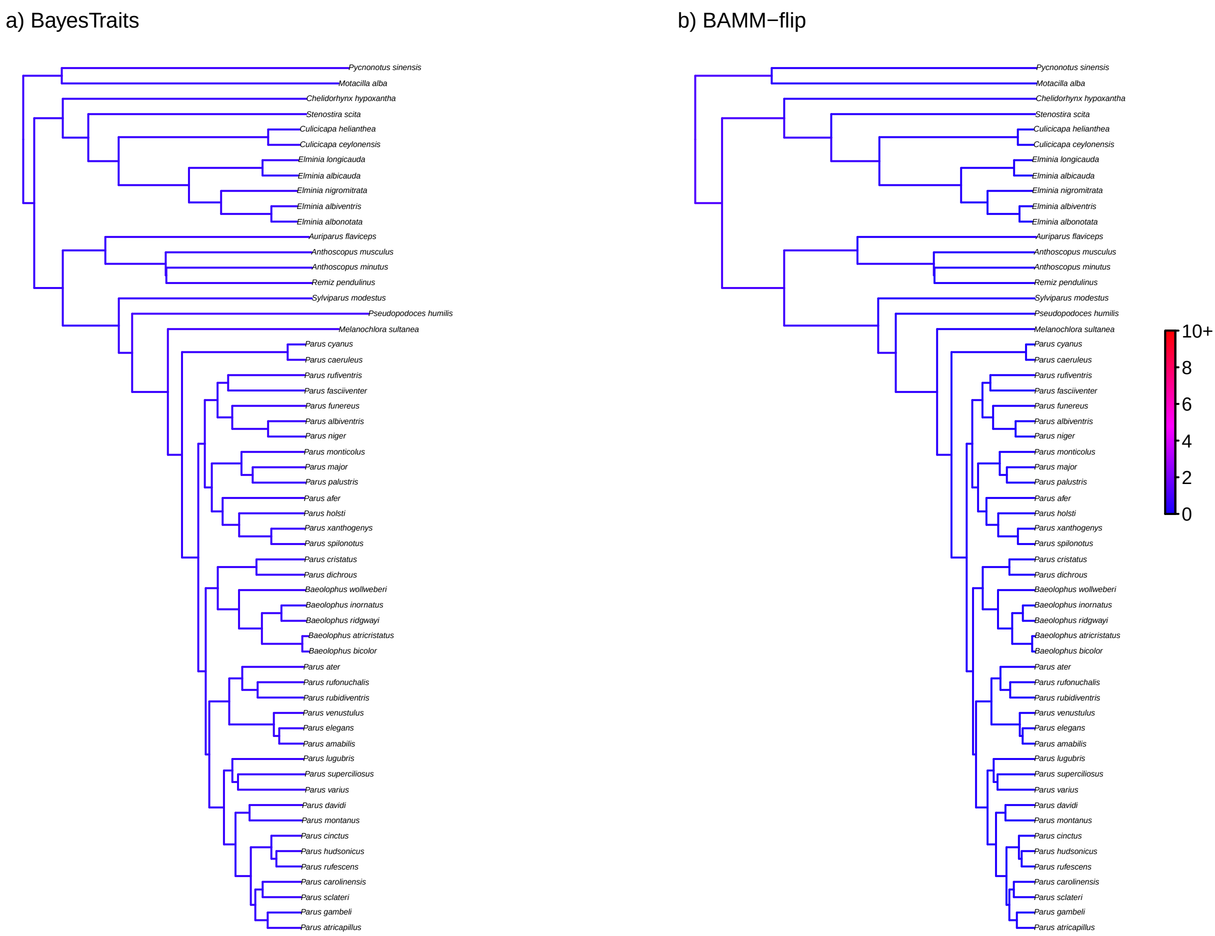

c)

	p_cvar	p_shgt	p_svar	p_sasr	AICw	sigma	alpha	r
BM	0.02 (+)	0 (+)	0.14 (+)	0.03 (+)	0.09	0.33		
OU	0.02 (+)	0 (+)	0.14 (+)	0.03 (+)	0.03	0.33	0	
EB	0.08 (+)	0.53 (−)	0.82 (−)	0.01 (−)	0.88	2.2		−3.45
BayesTraits	0.04 (+)	0.01 (+)	0.09 (+)	0.05 (+)				
BAMM−flip	0.09 (+)	0.09 (+)	0.25 (+)	0.01 (+)				

Figure S96

Cuckoo−Shrikes

a) BayesTraits

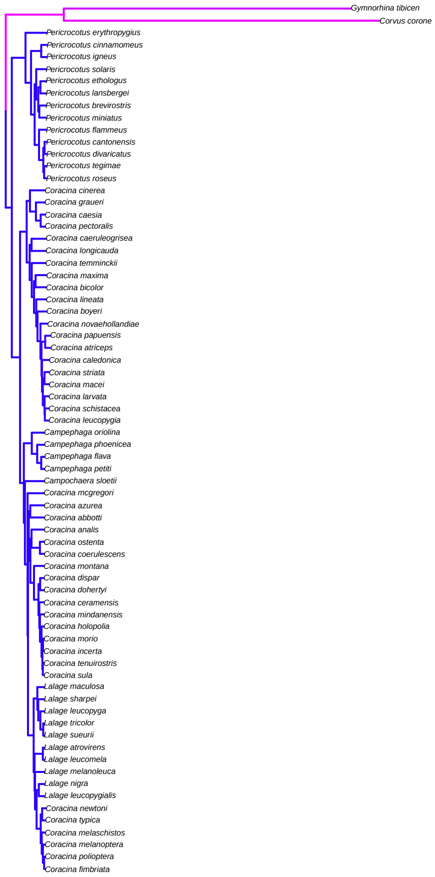

b) BAMM−flip

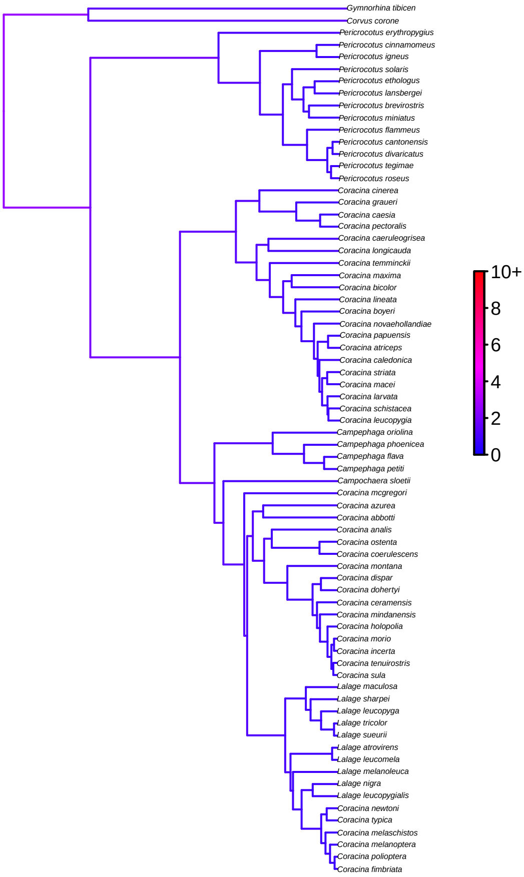

c)

	p_cvar	p_shgt	p_svar	p_sasr	AICw	sigma	alpha	r
BM	0.03 (+)	0.02 (+)	0.66 (+)	0.33 (+)	0.21	0.96		
OU	0.03 (+)	0.01 (+)	0.61 (+)	0.34 (+)	0.08	0.96	0	
EB	0.1 (+)	0.2 (+)	0.94 (+)	0.34 (+)	0.72	3.64		−2.85
BayesTraits	0.85 (+)	0.08 (+)	0.86 (+)	0.94 (+)				
BAMM−flip	0.11 (+)	0.06 (+)	0.64 (+)	0.45 (+)				

Figure S97

Kingfishers, Motmots, Todies

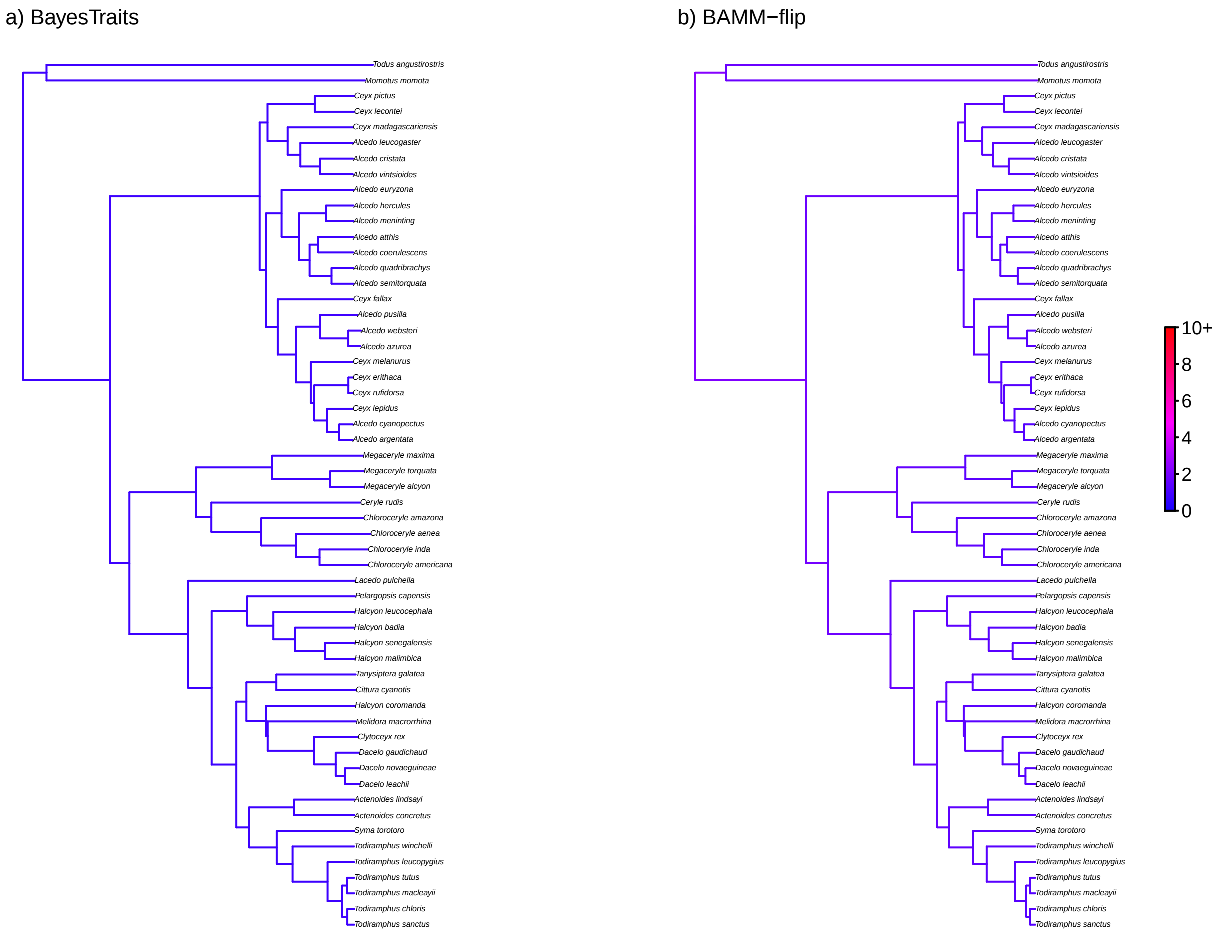

c)

	p_cvar	p_shgt	p_svar	p_sasr	AICw	sigma	alpha	r
BM	0.91 (−)	0.26 (+)	0.21 (+)	0.28 (+)	0.57	1.36		
OU	0.91 (−)	0.26 (+)	0.22 (+)	0.27 (+)	0.21	1.36	0	
EB	0.95 (+)	0.3 (−)	0.22 (−)	0.27 (−)	0.21	1.61		−0.22
BayesTraits	0.77 (−)	0.28 (+)	0.15 (+)	0.22 (+)				
BAMM−flip	0.93 (−)	0.28 (+)	0.2 (+)	0.25 (+)				

Figure S98

Albatrosses, Shearwaters, Petrels

a) BayesTraits

b) BAMM−flip

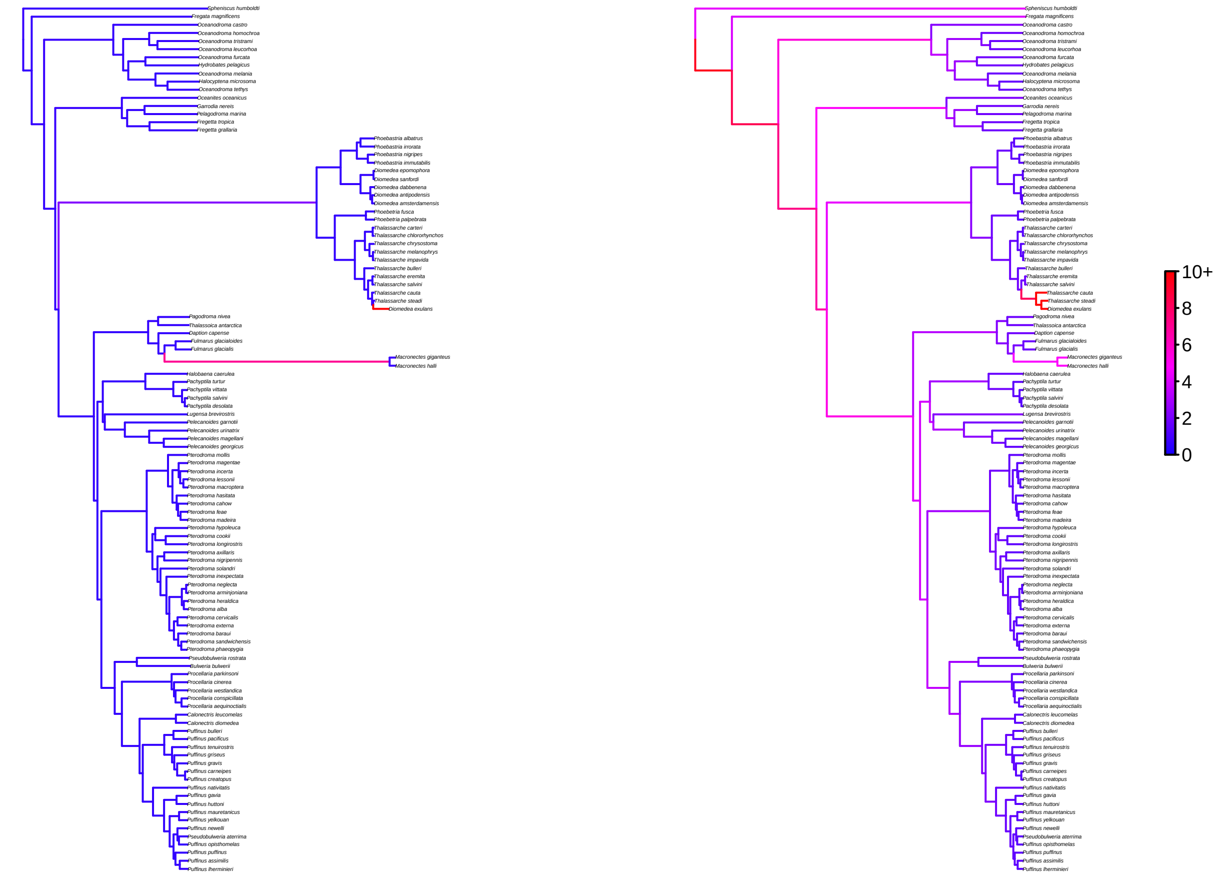

c)

	p_cvar	p_shgt	p_svar	p_sasr	AICw	sigma	alpha	r
BM	0 (+)	0 (+)	0.13 (+)	0.48 (+)	0.24	2.5		
OU	0 (+)	0 (+)	0.09 (+)	0.45 (+)	0.09	2.5	0	
EB	0 (+)	0.06 (−)	0.38 (−)	0.24 (+)	0.67	8.35		−2.23
BayesTraits	0.07 (+)	0 (+)	0 (+)	0.55 (−)				
BAMM−flip	0.1 (+)	0.05 (+)	0.08 (+)	0.95 (+)				

Figure S99

Shrike−Flycatchers, Helmetshrikes, Vangas, Butcherbirds, Woodswallows, Allies

a) BayesTraits

b) BAMM−flip

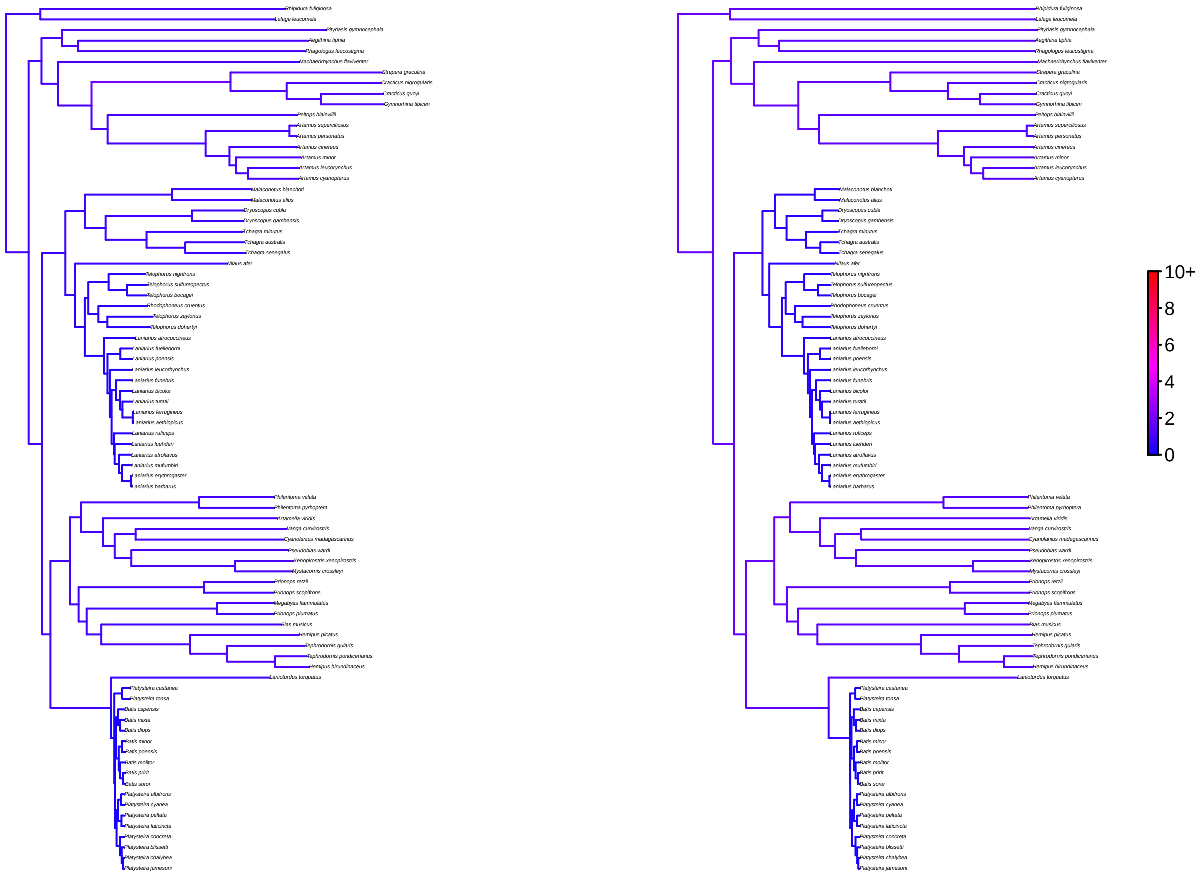

c)

	p_cvar	*p_shgt*	*p_svar*	*p_sasr*	*AICw*	*sigma*	*alpha*	*r*
BM	0.01 (+)	0 (+)	0 (+)	0.11 (+)	0.2	0.71		
OU	0 (+)	0 (+)	0 (+)	0.12 (+)	0.07	0.71	0	
EB	0.01 (+)	0.15 (−)	0.02 (−)	0.08 (+)	0.73	2.58		−2.61
BayesTraits	1 (−)	0.92 (−)	0.02 (+)	0.97 (−)				
BAMM−flip	0.87 (−)	0.8 (+)	0.14 (+)	0.71 (+)				

Figure S100

Parrots II

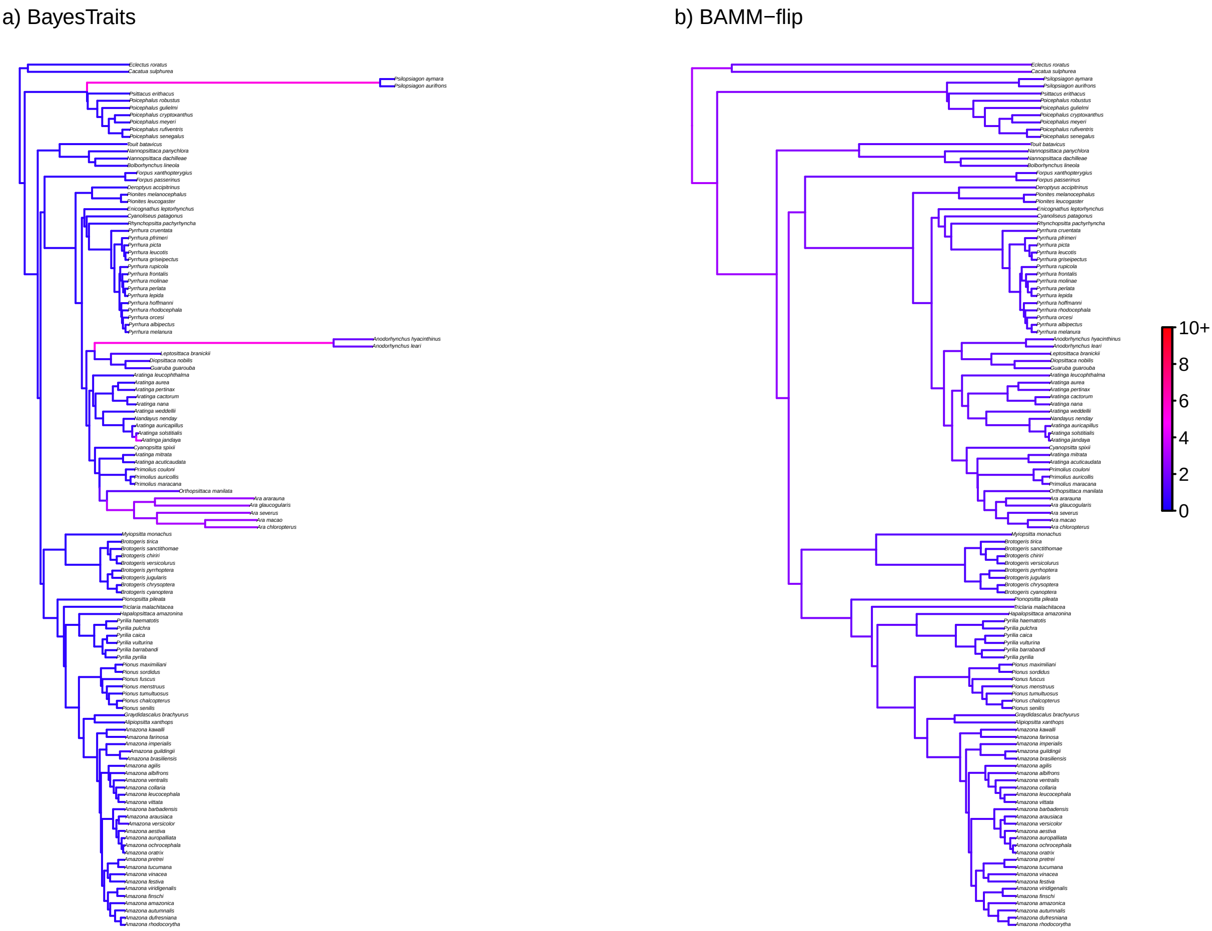

c)

	p_cvar	*p_shgt*	*p_svar*	*p_sasr*	*AICw*	*sigma*	*alpha*	*r*
BM	0 (+)	0.03 (+)	0.06 (+)	0.01 (+)	0.4	1.32		
OU	0 (+)	0.03 (+)	0.06 (+)	0.02 (+)	0.15	1.32	0	
EB	0 (+)	0.43 (+)	0.41 (−)	0.01 (+)	0.45	4.23		−2.12
BayesTraits	0.01 (+)	0.01 (+)	0 (+)	0.18 (+)				
BAMM−flip	0 (+)	0.17 (+)	0.02 (+)	0.01 (+)				

Figure S101

Pheasants, Quail, Guineafowl

a) BayesTraits

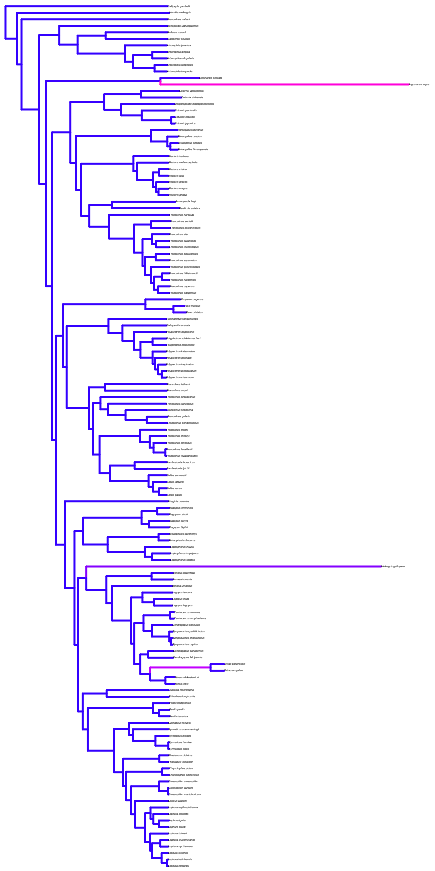

b) BAMM−flip

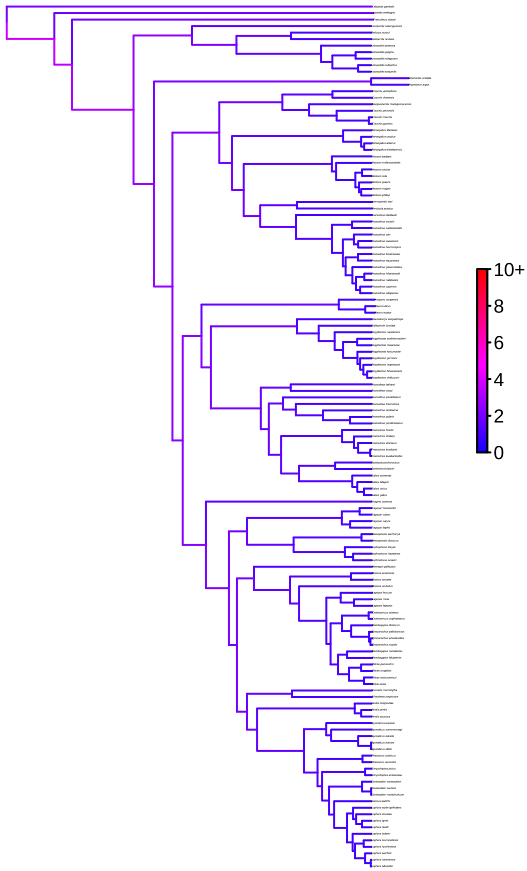

c)

	p_cvar	*p_shgt*	*p_svar*	*p_sasr*	*AICw*	*sigma*	*alpha*	*r*
BM	0.01 (+)	0.01 (+)	0.01 (+)	0.81 (+)	0.24	1.17		
OU	0.01 (+)	0.01 (+)	0.01 (+)	0.73 (+)	0.09	1.17	0	
EB	0.01 (+)	0.53 (−)	0.41 (−)	0.35 (+)	0.68	4.88		−2.21
BayesTraits	0.28 (+)	0.01 (+)	0 (+)	0.68 (−)				
BAMM−flip	0.02 (+)	0.25 (+)	0.1 (+)	0.59 (+)				

Figure S102

Honeyeaters

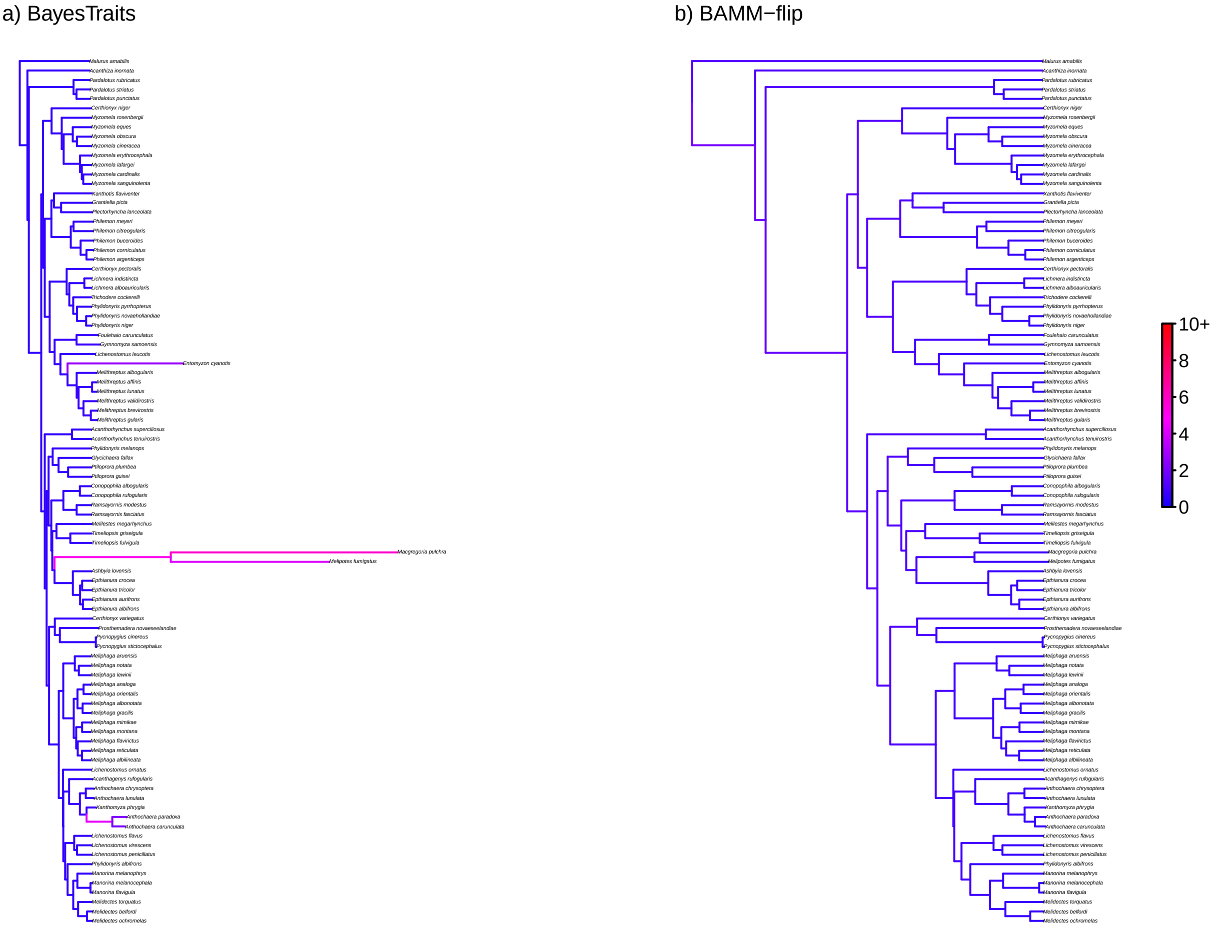

c)

	p_cvar	*p_shgt*	*p_svar*	*p_sasr*	*AICw*	*sigma*	*alpha*	*r*
BM	0.21 (+)	0.03 (+)	0.2 (+)	0.02 (+)	0.41	0.81		
OU	0.24 (+)	0.03 (+)	0.23 (+)	0.01 (+)	0.15	0.81	0	
EB	0.3 (+)	0.54 (+)	0.65 (+)	0 (+)	0.44	2.65		−1.64
BayesTraits	0.12 (−)	0.06 (+)	0.07 (+)	0.15 (+)				
BAMM−flip	0.45 (+)	0.12 (+)	0.25 (+)	0.01 (+)				

Figure S103

Antbirds

a) BayesTraits

b) BAMM−flip

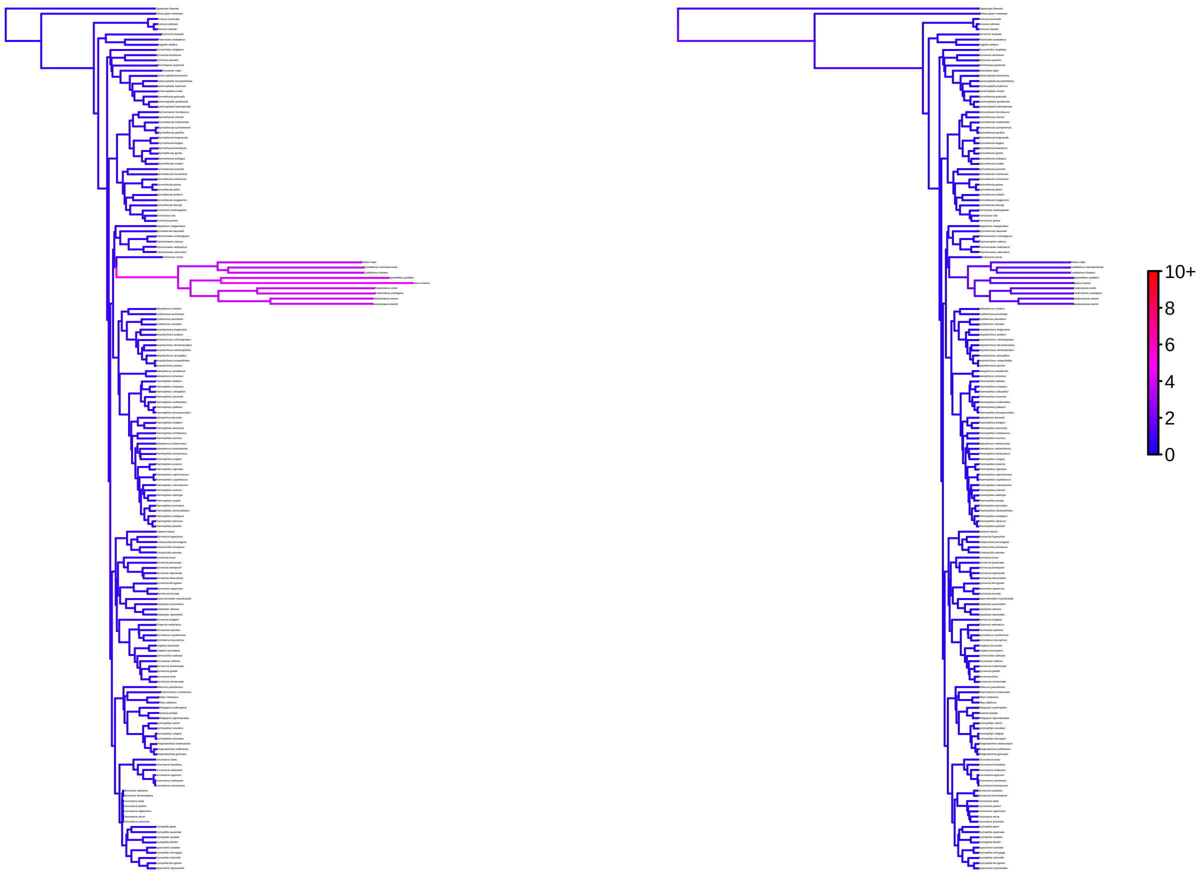

c)

	p_cvar	*p_shgt*	*p_svar*	*p_sasr*	*AICw*	*sigma*	*alpha*	*r*
BM	0 (+)	0 (+)	0.19 (+)	0.05 (+)	0	0.39		
OU	0 (+)	0 (+)	0.15 (+)	0.03 (+)	0	0.39	0	
EB	0.04 (+)	0.09 (−)	0.16 (−)	0.01 (+)	1	114.28		−4.99
BayesTraits	0.04 (+)	0 (+)	0.67 (+)	0.41 (+)				
BAMM−flip	0.05 (+)	0.48 (+)	0.55 (+)	0.19 (+)				

Figure S104
